# Amino acid composition drives aggregation during peptide synthesis

**DOI:** 10.1038/s41557-026-02090-0

**Published:** 2026-03-20

**Authors:** Bálint Tamás, Marvin Alberts, Teodoro Laino, Nina Hartrampf

**Affiliations:** 1 Department of Chemistry, University of Zürich, Zurich, Switzerland; 2 IBM Research Europe, Rüschlikon, Switzerland; 3 NCCR Catalysis, Zurich, Switzerland

**Keywords:** Flow chemistry, Solid-phase synthesis, Automation, Cheminformatics

## Abstract

Peptide aggregation is a long-standing challenge in chemical peptide synthesis, limiting its efficiency and reliability. Although data-driven methods have enhanced our understanding of many sequence-based phenomena, no comprehensive approach addresses so-called non-random difficult couplings (generally linked to aggregation) during solid-phase peptide synthesis. Here we leverage existing peptide synthesis datasets, supplemented with further experimental data, to build a predictive model that deciphers the role of individual amino acids in triggering aggregation. We first identified and experimentally validated composition-dependent aggregation as a stronger predictor than sequence-based patterns. This insight enabled the development of a composition vector representation, allowing insights into the aggregation propensities of individual amino acids. Applying an ensemble of trained models, we predicted the aggregation properties of peptides and recommended the optimized use of aggregation-reducing tools. By elucidating each individual amino acid’s influence, this method holds the potential to accelerate synthesis optimization through existing data, offering a robust framework for understanding and controlling peptide aggregation.

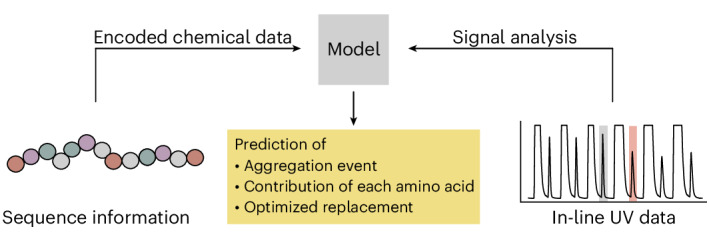

## Abstract (web summary)

Aggregation during solid-phase peptide synthesis is a bottleneck, often rendering peptide synthesis costly and inefficient. Now the impacts of individual amino acids on aggregation have been characterized through machine learning and analysis of composition patterns. The generated models predict problematic peptides and optimize synthesis conditions, enabling chemists to use aggregation-avoiding routes.

## Main

Peptides and proteins play diverse biological roles, functioning as hormones, enzymes and signalling molecules, which are critical for maintaining physiological processes. Their versatility and specificity have made them valuable therapeutic agents, driving innovations in the pharmaceutical industry^1^. Understanding their structures has been a long-standing challenge in biochemistry^2,3^. Despite key advances, human intuition alone has proven insufficient for a systematic understanding of the structure of proteins based on their primary sequence, leading to the widely known ‘protein folding problem’^4^. With decades of accumulated data, computational methods have emerged as essential tools to predict the structure of proteins^5–7^. This evolution in methodology culminated in the development of AlphaFold and RoseTTAFold, effectively solving the problem of accurately predicting a protein’s structure from its sequence^8,9^.

Although these developments have greatly enhanced our understanding of peptide and protein folding under physiological conditions, folding properties during solid-phase peptide synthesis (SPPS) remain comparatively unexplored. This gap stems from the vastly different conditions used: (1) the C terminus of the peptide is anchored to the solid support, affecting its flexibility and polarity; (2) 12 out of the 20 amino acids feature side-chain protecting groups, drastically altering their steric and electronic properties; and (3) instead of the aqueous solvents used for native peptides, the growing peptide chain is usually solvated in *N*,*N*-dimethylformamide (DMF) or similar aprotic solvents and surrounded by polymeric supports such as polystyrene or polyethylene glycol, resulting in a much less polar environment overall. During SPPS, the aggregation of resin- and linker-bound peptides often induces peptide folding, which can hinder synthetic efficiency and render certain sequences inaccessible. Aggregation is thought to originate from the undesired formation of β-sheet structures on the solid support^10–13^. This causes both truncations and deletions of the peptide sequence, often making it challenging, if not impossible, to isolate the desired peptide. Notably, even additional coupling or deprotection cycles and a large excess of amino acid do not lead to full conversion post-aggregation. Aggregation depends on several factors, such as synthesis temperature, loading of the solid support and—most importantly—the peptide sequence and its amino acid side-chain protecting groups. It has been shown that aggregation often occurs within 5–15 amino acids from the anchoring point to the resin^14,15^. Consequently, C-terminal amino acids exert the greatest influence on aggregation, with current literature suggesting that β-branched amino acids aggravate this effect^14^. Despite multiple attempts to understand the sequence dependence of aggregation experimentally^16–18^ and with advanced data analytics on UV data obtained from flow-SPPS^19,20^, a robust method to predict aggregation and to propose an alternative synthesis strategy remains elusive.

**Fig. 1:**
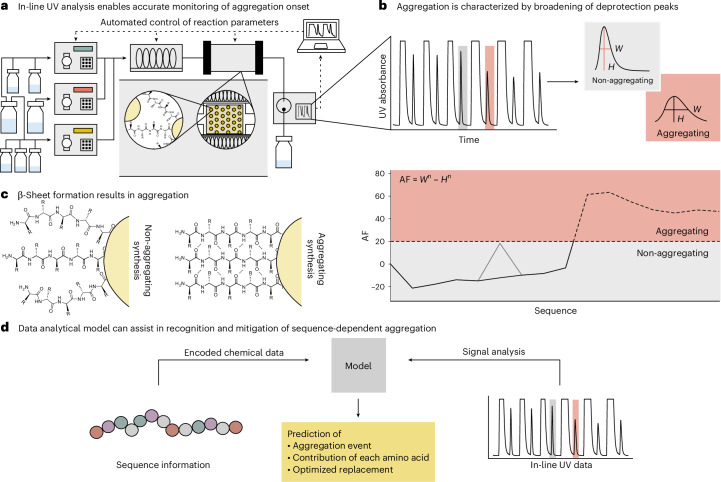
Analytical data collected with an in-line UV module enables data-driven methods for synthesis analysis. **a**, AFPS enables the precise monitoring of reaction kinetics, which corresponds to the aggregation of the sequences. **b**, Aggregation in the in-line UV traces is characterized as the broadening of the deprotection peak. Aggregation is quantified by an aggregation factor (AF), calculated using the following formula: AF = *W*^n ^− *H*^n^, where *W*^n^ is half of the maximum height, normalized to the first peak, and *H*^n^ is the peak height normalized to the first peak. If AF >20, the sequence is considered aggregating. **c**, Aggregation is driven by β-sheet formation between the growing peptide chains. **d**, In-line UV data collected during synthesis were leveraged to predict the occurrence of aggregation and the contribution of individual amino acids.

In this study, we use machine learning on deprotection peak data collected from the in-line UV–visible (UV–vis) of an automated fast-flow peptide synthesizer (AFPS)^21^. These data directly correlate to the aggregation state of the peptide being synthesized on the resin (Fig. 1b). We leverage the UV–vis data to gain insights into the factors contributing to peptide aggregation, including the influence of each individual amino acid. Through the shuffling of peptide sequences, it was found that the fractional composition (the percentage of amino acids present) of the peptide, rather than the specific sequence, largely determines the aggregation characteristics of a given peptide. We verify this claim experimentally and ultimately demonstrate how these findings can be used to avoid aggregation.

## Results and discussion

### Predicting aggregation during SPPS

Predicting peptide aggregation requires criteria to distinguish between aggregating and non-aggregating sequences. All data used in this study were collected on an AFPS platform equipped with an in-line UV–vis detector monitoring coupling and deprotection peaks during synthesis (Fig. 1a). Deprotection peaks, which result from 9-fluorenylmethyloxycarbonyl (Fmoc) removal, provide two crucial pieces of information: their area indicates the coupling/deprotection efficiency, while their shape reflects the aggregation state^16,20,22,23^. To ensure comparability, similarly to ref. ^19^, the traces were analysed using the aggregation factor (the difference of the normalized height and width of the peak) and we defined aggregation as the deprotection peak broadening by more than 20% relative to the first coupling. If any peak during synthesis exceeds this threshold, we classify the entire sequence as aggregating. In practice, this directly correlates to a decreased crude purity (Fig. 1b).

Next, we used machine learning to predict the aggregation characteristics of a given peptide using two datasets:one published by Mohapatra et al.^19^ and one internal dataset. Both were generated using similar AFPS platforms^21^ and synthesis conditions, ensuring minimal statistical deviation between the two. After curating and merging the two datasets (see ‘Extended computational and experimental methods’ in the Supplementary Information), the combined dataset comprised 539 peptide sequences. Of the total sequences, 420 were sourced from the Mohapatra dataset, with 48.8% showing aggregation, and an additional 119 sequences from our internal dataset, where 53.8% aggregated. This resulted in a nearly balanced combined dataset, with 49.9% of sequences exhibiting aggregation. As aggregation typically occurs 5–15 amino acids from the anchoring point to the resin, all peptides longer than 20 amino acids were truncated and those shorter than five amino acids were discarded (see Supplementary Fig. 1 for length distribution). Peptides containing non-canonical amino acids were also filtered out as only 49 sequences containing various non-canonical amino acids were available, making it very challenging for a machine learning model to learn patterns.

**Fig. 2:**
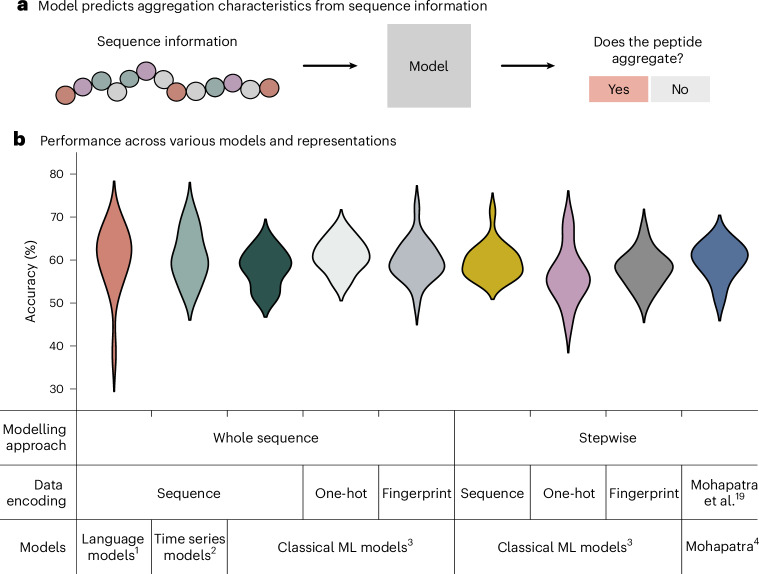
Prediction accuracy is independent of model or representation. **a**, A variety of different models ranging from language models to classical machine learning (ML) models were trained to predict whether a given peptide sequence aggregates. **b**, Consistent prediction accuracy scores are observed across all models and representations regardless of the model, chemical representation or if the sequence is fed stepwise or as a whole sequence. The violin plot shows the distribution of accuracies for the different modelling approaches and representations. Each distribution (or colour) illustrates the accuracies of a model family across the five test splits. Superscripts denote model groups: (1) language models (ESM 2.0, BERT); (2) time series models (HIVE-COTE 2.0, WEASEL, time forest); (3) classical ML models (XGBoost, random forest, KNN, Gaussian processes); and (4) Mohapatra et al.^19^ model variants (complete/minimal).

While extensive research has been conducted on identifying suitable statistical models and molecular representations for proteins, considerably less attention has been devoted to peptides. To address this gap, we explored a wide range of models and representations for peptide synthesis data. All models were trained with fivefold cross-validation (that is, the data are randomly split into five folds and training is repeated five times using a different fold as the test set) and the performance of each model was assessed using the accuracy. We average the accuracy over the five runs and report the mean accuracy and standard deviation. The accuracy distribution over the five trainings for different models and representations is illustrated in Fig. 2, with the mean and standard deviation reported in Supplementary Table 1. We framed the problem as a binary classification task: does a given peptide sequence aggregate or not? Our data were collected during synthesis, allowing for two distinct prediction approaches: either predicting the aggregation characteristics of the final synthesized peptide directly or leveraging the step-by-step nature of the synthesis process. During synthesis, the peptide is elongated amino acid by amino acid, with information on whether the peptide has aggregated available at each synthesis step. We explored both approaches for the predictions (Fig. 2): ‘whole sequence’ corresponds to predictions based on the final peptide sequence and ‘stepwise’ emphasizes the step-by-step nature of the syntheses. For the step-by-step approach, all peptides are labelled as non-aggregating for the first few couplings. Once an aggregation event (that is, broadening of the deprotection peak) occurs, all subsequent peptide couplings are labelled as aggregating.

To evaluate both approaches, we experimented with a range of models and representations. One highly successful approach for proteins treats the amino acid sequence as text and leverages language models to predict protein properties^24,25^. Inspired by this approach, we fine tuned a specialized protein language model (ESM 2.0^26^) as well as a generalist language model (BERT^27^) to classify whether a peptide aggregates. In addition to fine-tuning pretrained models, we also trained a BERT model from scratch (Fig. 2, Language models). Another common data type in machine learning are time series. A time series consists of a sequence of data points collected at regular time intervals. Following this definition, the stepwise synthesis of a peptide can be considered a time series with each addition of an amino acid corresponding to one time step. We trained three state-of-the-art time series classification models on this problem, representing each amino acid with a numerical token and padding to accommodate varying sequence lengths (Fig. 2, Time series models).

In addition to these models, we also explored the performance of classical machine learning models (for example, random forest^28^ and XGBoost^29^) on three different representations. These representations consist of a numerical token matching the approach for time series models, a one-hot encoding approach and a fingerprint-based method inspired by ref. ^19^ (for more details, see ‘Extended computational and experimental methods’ in the Supplementary Information). Last, we assessed the performance of the model by Mohapatra et al.^19^. Surprisingly, we observed similar performance across all representations, models or hyperparameter configurations (Fig. 2 and the accuracies for each combination are reported in Table 1).

**Table 1:** Accuracy scores: the table displays the accuracy obtained for the different representations and models. Each combination was trained with fivefold cross-validation, with the mean accuracy and standard deviation over five folds reported

Modelling approach	Data encoding	Model	Accuracy (%)
Whole sequence	Text	ESM 2.0 (35 M)	62.1 ± 6.1
		Bert (base, pretrained)	59.6 ± 4.3
		Bert (base, trained from scratch)	60.6 ± 11.3
	Sequence (time series)	HIVE-COTE 2.0	61.4 ± 6.9
		Time forest	60.7 ± 6.0
		WEASEL	60.6 ± 3.0
	Sequence	Random forest	59.9 ± 3.0
		XGBoost	58.0 ± 3.5
		KNN	57.3 ± 4.5
		Gaussian processor	56.5 ± 4.4
	One-hot encoding	Random forest	62.8 ± 2.7
		XGBoost	58.9 ± 2.6
		KNN	60.8 ± 3.5
		Gaussian processor	61.4 ± 4.2
	Fingerprint	Random forest	62.1 ± 5.7
		XGBoost	59.9 ± 3.4
		KNN	59.9 ± 1.8
		Gaussian processor	57.1 ± 4.4
Stepwise	Sequence	Random forest	59.9 ± 2.9
		XGBoost	60.1 ± 2.0
		KNN	58.2 ± 2.5
		Gaussian processor	60.1 ± 5.9
	One-hot encoding	Random forest	59.3 ± 4.5
		XGBoost	58.3 ± 6.4
		KNN	54.7 ± 2.0
		Gaussian processor	54.0 ± 7.9
	Fingerprint	Random forest	58.4 ± 4.6
		XGBoost	56.5 ± 4.7
		KNN	58.6 ± 3.7
		Gaussian processor	57.8 ± 1.7
	Mohapatra	Complete	61.8 ± 2.5
		Minimal	57.8 ± 4.1

The comparable performance of the Mohapatra model^19^ can be attributed to differences in the modelling approach: Mohapatra et al.^19^ aim to predict the characteristics of UV traces obtained during SPPS synthesis, whereas we aim to predict aggregation as a binary classification task. While Mohapatra et al.^19^ report relative root mean squared error values up to 5% for predicted UV traces, it is important to note that this error does not perfectly map to predicting aggregation outcomes. One reason for this is that the in-line UV traces contain many positions (for example, the first six to ten residues during synthesis or positions in non-aggregating sequences) where the traces are constant. These regions contribute to lowering the regression error for any model, even a null predictor, yet they do not improve discrimination between aggregating and non-aggregating sequences. As a result, regression metrics can be inflated by modelling these trivial regions rather than learning aggregation-specific patterns. As such, even a near-perfect predictor of the full UV trace will be limited in its aggregation classification performance, as the trace itself is only a partial proxy for aggregation. This explains why the Mohapatra model^19^, despite predicting accurate UV traces, achieves a classification accuracy of 60%. While this explains the performance of the Mohapatra model^19^, it does not diminish the observation that all tested configurations, including different model architectures, data representations and hyperparameter settings, yield very similar accuracies.

To further guide our modelling approach, we focused on labelling the most relevant segment of the sequence, leveraging the step-by-step nature of the synthesis process. We hypothesized that the sequence preceding the aggregation point (that is, the amino acid coupling at which aggregation occurs) is the most informative to distinguish between aggregating and non-aggregating sequences. By contrast, the remaining peptide sequence beyond the aggregation point contains little to no meaningful information. Therefore, we systematically investigated how many amino acids before and after the aggregation point are ideal to label as aggregating: we evaluated ranges up to ten amino acids before and after the point of aggregation (Supplementary Fig. 2). Across all screened hyperparameters, models or representations, the performance remained consistent provided the sequences were sufficiently long to form secondary structures (>6 amino acids)^30^. This suggests that either aggregation may be determined by factors other than peptide sequence or the models assessed were unable to effectively capture the aggregation signal from the data.

### Amino acid composition influences aggregation

The consistent results across different models and representations prompted us to question the quality and consistency of our dataset. As a validation experiment, we trained all models on a shuffled version of the peptide sequence. Assuming aggregation is highly sequence dependent, inconsistent performance with shuffled data would indicate that the models fail to capture a sequence-specific aggregation signal.

**Fig. 3:**
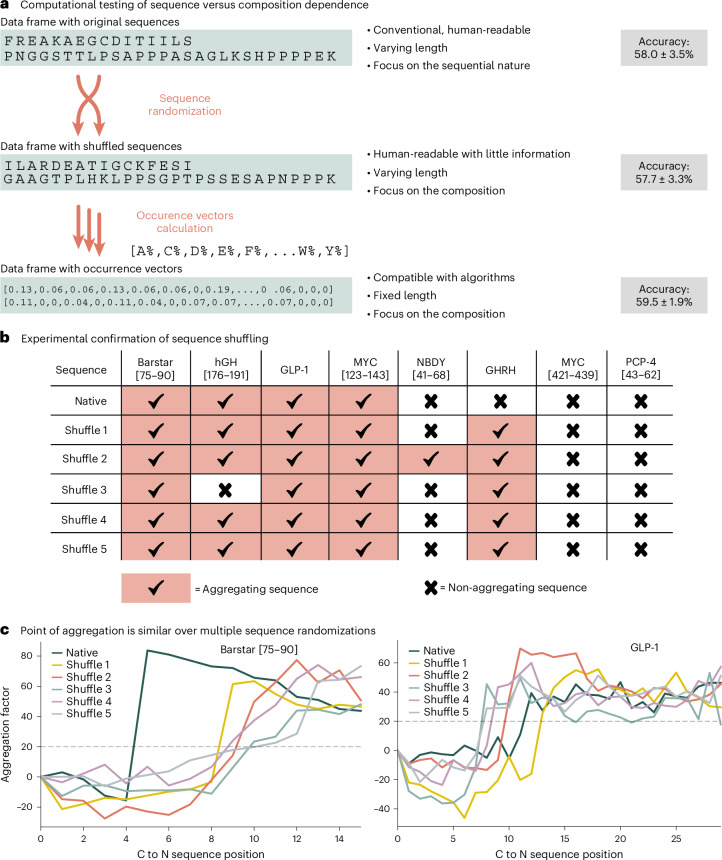
Computational and experimental investigation of sequence shuffling on aggregation behaviour. **a**, Training models on randomly shuffled sequences or only with a composition vector of the amino acids present in the peptide does not lead to a decrease in accuracy compared with training on the original sequences. **b**, To verify the computational results, four aggregating and four non-aggregating sequences were synthesized with five reproducible shuffles each. **c**, For aggregating test peptides, the point of aggregation remains consistent across the shuffled peptide sequences. Native UV–vis data for Barstar from ref. ^20^ and GLP-1 from ref. ^13^.

We trained XGBoost models using whole-sequence representation on both the original and a randomly shuffled dataset. No meaningful difference in accuracy was observed (58.0% ± 3.5% for the original sequences versus 57.7% ± 3.3% for the shuffled sequences). This result was consistent across all tested representations and models (Supplementary Information section 3). These findings challenge the widely accepted view of aggregation as a phenomenon that is highly dependent on peptide sequence^15^. To investigate this further, a simplified encoding method was developed, representing each sequence as a 20-dimensional vector corresponding to the normalized composition of amino acids. Using this minimal representation, the accuracy remains comparable (59.5% ± 1.9%), reinforcing the notion that amino acid composition might outweigh sequence order in influencing aggregation (Fig. 3a).

To experimentally test whether composition, rather than sequence, determines aggregation, we selected eight literature-known test peptides and synthesized five randomly shuffled variants of each peptide (Fig. 3b). Barstar [75–90]^20^, hGH [176–191] Y176F (abbreviated as hGH)^13^, GLP-1 (ref. ^13^) and MYC [123–243]^13^ were selected as aggregating sequences, and NBDY [53–68]^20^, GHRH^21^, MYC [421–439] and PCP-4 [43–62] as non-aggregating sequences. The shuffled sequences were generated through a reproducible randomization process to avoid selection bias. The peptides, ranging from 16 to 28 amino acids in length, were experimentally evaluated for aggregation behaviour during AFPS. In alignment with the in silico results, 19 out of 20 of the shuffled aggregating peptides retained their aggregation characteristics, while 14 out of 20 of the shuffled non-aggregating sequences also maintained their non-aggregating character (Fig. 3). The majority of peptides preserve their aggregation characteristics, regardless of amino acid order, as long as the overall composition remains unchanged. In addition, the aggregation point also remains similar for the shuffled sequences (Fig. 3c). This suggests that a factor beyond the sequence, that is, amino acid composition, play a prominent role in determining peptide aggregation rather than sequence information alone.

### Individual amino acid contribution to aggregation

To understand how individual amino acids influence aggregation, we analysed their contributions using Shapley Additive Explanations (SHAP)^31^. We trained an XGBoost classifier on 50 different random splits of the data to ensure robust SHAP value extraction. The composition vector was used to represent peptides and SHAP values were employed to interpret and quantify each amino acid’s contribution to aggregation. Positive SHAP values indicate amino acids that increase the likelihood of predicted aggregation, while negative values suggest the opposite. This approach established a direct relationship between amino acid composition and model predictions, providing insights into the molecular determinants of peptide aggregation.

**Fig. 4:**
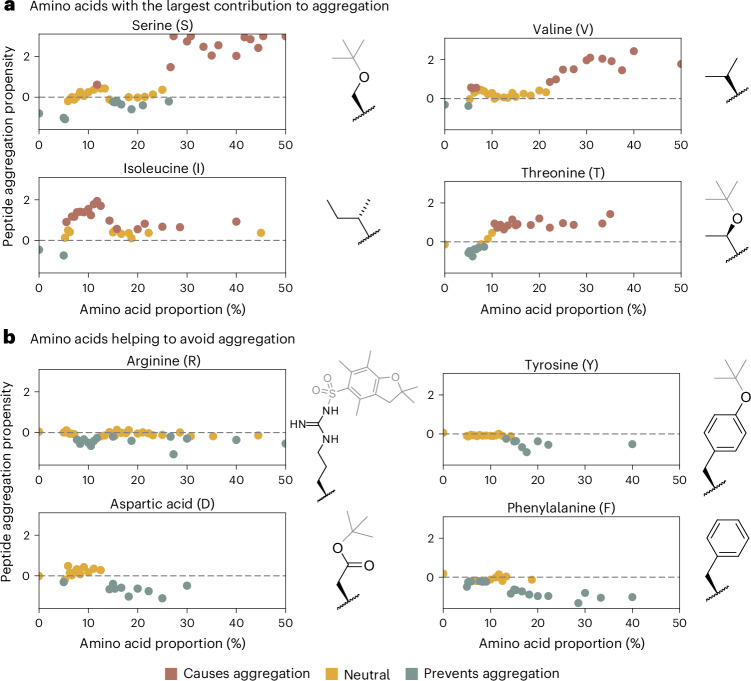
Analysis of amino acids influencing the XGBoost classifier’s decision making the most. The *x* axes represent the amino acid proportion in the sequences, with the *y* axes corresponding to the mean SHAP value. A positive value is associated with a higher likelihood of this model predicting aggregation and a negative one with a lower aggregation chance. **a**, Amino acids that contribute the most to aggregation: serine, valine, isoleucine and threonine. **b**, Amino acids that contribute the least to aggregation: arginine, tyrosine, aspartic acid and phenylalanine.

The analysis revealed distinct patterns in how different amino acids influence aggregation (Fig. 4). Amino acids such as Ser(*t*-Bu), Ile, Val and Thr(*t*-Bu) were found to increase the likelihood of aggregation the most when present in higher proportions, while Gln(Trt) and Leu are also major contributors. Conversely, the presence of Phe, Asp(*t*-Bu), Tyr(*t*-Bu) and Arg(Pbf) tended to reduce aggregation the most, followed by Cys(Trt), His(Trt) or Pro. The remaining amino acids appeared to contribute neutrally, without a strong positive or negative effect (Supplementary Information section 5). While our analysis revealed peptide composition to be predominantly driving aggregation, other factors influence aggregation as well. To this end, we investigated the effect of dipeptide motifs on aggregation, with Gly–Ser and Leu–Leu contributing most to aggregation (Supplementary Information section 4).

The aggregation-promoting amino acids generally have aliphatic, non-polar side chains, which seem to facilitate intermolecular interactions and packing between peptide strands. By contrast, amino acids that inhibit aggregation often have aromatic or polar side groups, which may increase spacing and disrupt aggregation-prone structures.

### Trained models suggest conditions for improved SPPS

**Fig. 5:**
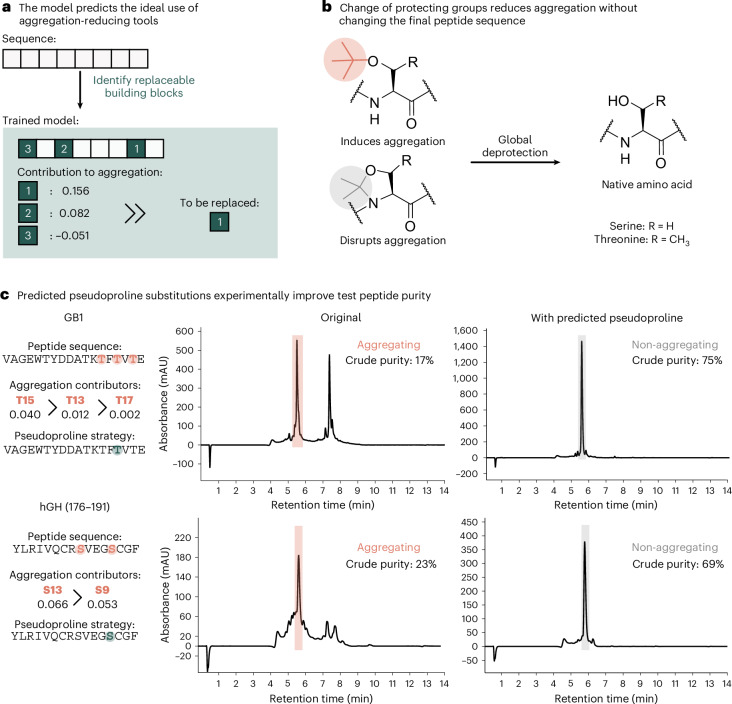
Leveraging the XGBoost ensemble for rational use of aggregation reduction tools to suggest improved synthesis conditions. **a**, With the user input sequence and replaceable amino acids, the trained model ensemble predicts and scores the aggregation property of the sequence and predicts the contribution of the present amino acids in the early fragment of the peptide (position 2–12). This enables more effective introduction of aggregation-suppressing moieties. **b**, Serine and threonine, two *t*-Bu-protected amino acids with substantial predicted contribution to aggregation, can also be introduced as pseudoprolines. The latter are established aggregation-reducing tools, which yield the native amino acid upon global deprotection. **c**, The potential of this model was tested in two known aggregating sequences: GB1 and hGH. The serines and threonines with the largest contribution were selected and replaced, resulting in a substantial crude purity increase of 58% for the GB1 fragment and 46% for the hGH fragment.

The optimization of peptide synthesis can be a tedious process: as aggregation is difficult to predict, the usual workflow requires repetitive synthesis with the trial-and-error use of known aggregation-reducing tools. Our trained XGBoost classifier not only enables the prediction of the aggregation propensity of a given peptide but also provides insights into how aggregation could be mitigated through strategic modifications. By understanding the contributions of specific amino acids, the most effective use of aggregation-reducing tools, such as different backbone and side-chain protecting groups, can be predicted. The algorithm we developed works as follows (Fig. 5a): 100 XGBoost classifiers were trained on varying splits of the data, forming an ensemble to avoid bias stemming from the relatively small size of the dataset. The user inputs the peptide sequence and the amino acids with available aggregation-reducing substitutions. The models then predict whether the given sequence is likely to aggregate. If the sequence is predicted to be aggregating, the key positions (2–12) are analysed to identify amino acids that could be substituted with their aggregation-reducing counterparts. These potential substitutions are then ranked in order of their relative contribution to aggregation, allowing the user to prioritize the most impactful changes.

To test this capability, we selected two aggregating sequences, hGH and GB1, and pseudoproline-protected amino acid building blocks as a widely used tool to mitigate aggregation^32^. Notably, pseudoprolines are aggregation-disrupting equivalents of the two protected amino acids with the highest contribution, Ser(*t*-Bu) and Thr(*t*-Bu) (Fig. 5b). For hGH, 74% of the ensemble of 100 XGBoost classifiers predicted aggregation, which increased to 90% for GB1. Next, the contribution of Ser(*t*-Bu) and Thr(*t*-Bu) in the 2–12 amino acids from the resin (C terminus) was assessed yielding three potential substitutions for GB1 and two for hGH, ranked by their potential to mitigate aggregation. For both peptides the native as well as all pseudoproline-substituted variants were synthesized with crude purities matching the ordering, as suggested by the model (Supplementary Information section 7) The incorporation of the ideal pseudoproline resulted in a crude purity increase from 23% to 69% for hGH and 17% to 75% for GB1 (Fig. 5c). In summary, the developed algorithm can use the trained models to predict the aggregation property and suggest the ideal position to incorporate aggregation-reducing tools to improve synthetic efficiency.

## Conclusion

In this study, machine learning was used as a discovery tool, uncovering a surprisingly strong composition dependence of peptide aggregation. This finding was validated experimentally by synthesizing 40 sequences (8 established sequences, each shuffled 5 times). In the process, we developed a simple composition vector as a peptide representation to investigate the aggregation character during SPPS. By leveraging the interpretability of this representation, we found that bulkier and more polar side chains or protecting groups have a tendency to reduce aggregation, while characteristically aliphatic side chains increase the likelihood of aggregation. We demonstrated the practical value of these findings by pinpointing the key amino acids contributing to aggregation in a given target peptide. By strategically introducing pseudoprolines at these positions, we observed a reduction in aggregation and an increase in the purity of two test sequences by 58% and 46%, respectively.

These findings question the understanding of aggregation as a mainly sequence-dependent event originating from intermolecular hydrogen bonding between backbones, resulting in β-sheet structures^10,11,13,15,30^. For biological systems, it has been established that amino acids with aliphatic side chains, such as valine or leucine, tend to be large contributors to β-sheet formation and aggregation^33,34^. Aromatic side chains also seem to have a major impact on the aggregation of native peptides and proteins under physiological conditions^34^. Our findings revealed that during SPPS, amino acids with aliphatic side chains, such as valine or isoleucine, predominantly contribute to aggregation. Protecting groups that mimic these structures, such as *t*-Bu-protected serine or threonine, exhibit similar behaviour during SPPS. In contrast to native peptides, amino acids with aromatic side chains or protecting groups, such as phenylalanine or tyrosine, tend to reduce aggregation occurrence. Furthermore, aggregation is widely considered sequence dependent, yet our results indicate that during SPPS, amino acid composition is more influential. This discovery led to the development of the composition vector, a simplified representation of peptides that allowed predicting the onset of aggregation while also recommending mitigation strategies.

Our machine learning-driven approach revealed previously undetected patterns in peptide aggregation. The strong correlation between peptide composition and aggregation emerged only through the use of computational analysis, highlighting how machine learning can discover complex relationships in chemical systems. This work demonstrates that the value of machine learning in chemistry extends beyond its common applications in property prediction and molecular generation: it serves as a powerful discovery tool that can challenge established models and uncover hidden patterns in molecular data.

## Methods

### Computational methods

#### Dataset curation

The data used in this study consist of the UV traces gathered during the SPPS of various peptides. We used the dataset published by Mohapatra et al. containing 769 unique syntheses (last accessed 10 February 2025) in addition to an internal dataset of 167 unique syntheses. Both datasets were combined and all syntheses containing non-canonical amino acids, steps not performed on an AFPS (for example, batch synthesis of a pre-chain) and synthesis of peptides with fewer than five amino acids were removed. As aggregation was reported to primarily occur between amino acids 5 and 15, only the synthesis steps of the first 20 amino acids were considered^15^. In addition, we filtered all duplicated sequences from the dataset. This reduced the size of the combined dataset to 539 unique syntheses. We defined aggregation as a broadening of the deprotection peak in excess of 20% compared with the first deprotection peak. During the synthesis, the addition of histidine and cysteine requires changes in the temperature of the reactor, causing a broadening of the deprotection peak. Following ref. ^20^, we ignored these peaks and interpolated with the previous and subsequent peaks for all histidine and cysteine additions.

#### Data processing

We used two processing strategies for the peptide sequences (1) Step-by-step: since SPPS builds the peptide sequence one amino acid at a time and aggregation information is available for each synthesis step, the problem can be framed as predicting whether a peptide sequence has aggregated at a given synthesis step. In theory, this approach has multiple advantages. It exposes the assessed models to a considerably larger amount of training data (a total of 7,000 synthesis steps in the dataset) and enables the practitioner to not only predict whether a peptide will aggregate, but also pinpoint where aggregation occurs. In total, this approach yielded 7,000 training samples. (2) Whole peptide: in this approach, we only considered the full peptide sequence and labelled it as aggregating or not aggregating. This yielded 539 training samples.

#### Peptide representation

Text: in this approach we leveraged pretrained Transformer models to predict whether a peptide aggregates. The peptide sequence is used as is and fed into the tokenizer of the Transformer model. ESM and BERT models were used.

Sequence: this representation converts a peptide into a vector by mapping each amino acid to a value between 1 and 20. We padded each sequence to the maximum sequence length (in this case 20) and fed this vector into the models. For this representation, we evaluated the performance of the time series models (HIVE-COTE 2.0, WEASEL and time forest) as well as the classical machine learning models (XGBoost, random forest, *K*-nearest neighbours (KNNs) and Gaussian processes).

One-hot encoding: this approach works similarly to sequence representation. Instead of mapping each amino acid to a numerical value, we one-hot encoded each amino acid and concatenated the vectors. In addition, we pad the resultant vector to match the maximum sequence length. Classical machine learning models were evaluated on this representation (XGBoost, random forest, KNNs and Gaussian processes).

Fingerprint: this approach is inspired by Mohapatra et al.^19^. Here, we used a Morgan Fingerprint^35^ with a radius of three and a bit size of 128 to represent each amino acid. We concatenated the fingerprint for each amino acid and padded the vector with zeros to a uniform length regardless of the sequence size. We trained classical machine learning models (XGBoost, random forest, KNNs and Gaussian processes) on this representation.

Composition vector: for a given peptide, we constructed a normalized vector where each index corresponds to a specific amino acid. This vector is built as follows: assign a fixed index to each of the 20 standard amino acids, creating a 20-dimensional vector followed by counting the number of occurrences of each amino acid and populating the corresponding vector indices. This vector is normalized by dividing by the total number of amino acids, ensuring that the vector represents the proportional composition of the peptide independent of its length.

#### Models

All models were trained with fivefold cross-validation using the same seed for each training ensuring consistency between runs. Fivefold cross-validation works by randomly splitting the data into five equal-sized subsets (folds), with each fold serving as a test set once while the remaining four folds are used for training. This yielded five accuracies, which we averaged to provide a more reliable assessment of the performance of the model.

Fine-tuning ESM 2.0 and BERT: for ESM 2.0 and BERT, the implementations provided on Huggingface were used. The problem is phrased as a sequence classification task for a given peptide sequence. The entire model is fine tuned. We used a standard Huggingface trainer with a learning rate of 2.5e-5, a batch size of 16 and a weight decay of 0.01. Adam is used as an optimizer with β_1 of 0.9 and β_2 of 0.99. We trained each model for 15 epochs and evaluated the model with the best validation loss. For ESM 2.0, we evaluated the sizes varying from 8 M, 35 M, 350 M to 650 M whereas for BERT we evaluated the base and large checkpoints. For ESM 2.0 we only used pretrained models, whereas for BERT we both fine tuned a pretrained model and trained a model for each size from scratch.

All time series models were used as implemented in the SKTIME library^36^ using the default parameters.

HIVE-COTE version 2: we used the implementation as provided by SKTIME with 500 estimators and a time limit of 10 min (ref. ^37^).

WEASEL: WEASEL was used with ANOVA and bigrams using ‘information-gain’ as the binning strategy^38^.

Time forest: the time series forest classifier was used with a minimum interval of three and 200 estimators^39^.

XGBoost: we used the implementation in the XGBoost library^29^ with the default settings.

Scikit-learn models: all scikit-learn models were used with the default hyperparameters. We evaluated the random forest-, Gaussian processes- and KNN-classifier^40^.

Models by Mohapatra et al.^19^: the implementation and hyperparameters provided in the GitHub page linked to the paper were used. Mohapaptra et al. provide two models: one using only peptide sequence as input and one supplemented with the synthesis conditions. Both models were evaluated.

#### Explainability

We used the SHAP library^31^ to explain the predictions of the models. Specifically, we leveraged the TreeExplainer and we trained a total of 50 models on random splits of the data to avoid noise in the explanations.

### Experimental

#### Reagents and solvents

Fmoc- and side-chain protected L-amino acids (Fmoc-Ala-OH, Fmoc-Arg(Pbf)-OH, Fmoc-Asn(Trt)-OH, Fmoc-Asp(O*t*-Bu)-OH, Fmoc-Cys(Trt)-OH, Fmoc-Gln(Trt)-OH, Fmoc-Glu(O*t*-Bu)-OH, Fmoc-Gly-OH, Fmoc-His(Trt)-OH, Fmoc-Ile-OH, Fmoc-Leu-OH, Fmoc-Lys(Boc)-OH, Fmoc-Met-OH, Fmoc-Phe-OH, Fmoc-Pro-OH, Fmoc-Ser(*t*-Bu)-OH, Fmoc-Thr(*t*-Bu)-OH, Fmoc-Trp(Boc)-OH, Fmoc-Tyr(*t*-Bu)-OH and Fmoc-Val-OH) and *N*′-tetramethyluronium hexafluorophosphate (HATU) were purchased from Bachem. *O*-(7-Azabenzotriazol-1-yl)-*N*,*N*,*N*′ and (7-azabenzotriazol-1-yloxy)tripyrrolidinophosphonium hexafluorophosphate (PyAOP) were purchased from Advanced ChemTech. *N*,*N*-diisopropylethylamine (*i*-Pr_2_NEt, DIPEA, 99.5%) was purchased from Sigma-Aldrich. Trifluoroacetic acid (for HPLC, ≥99.0%), triisopropylsilane (98%) and 3,6-dioxa-1,8-octane-dithiol (95%) were purchased from Sigma-Aldrich. DMF was purchased from the from VWR International GmbH. Dichloromethane (DCM, ≥99.8%) was purchased from Fisher Scientific Ltd. Diethyl ether was purchased from Honeywell Riedel-de Haën. Acetonitrile (MeCN, for HPLC gradient grade, ≥99.9%) was purchased from Sigma-Aldrich. NovaPEG Rink Amide resin (0.41 or 0.20 mmol g^−1^ loading) was purchased from the Novabiochem-line from Sigma-Aldrich. Piperidine (>99%, for synthesis) was purchased from Carl Roth GmbH. Formic acid (reagent grade, >95%) and AldraAmine trapping agent added to DMF were purchased from Sigma-Aldrich.

#### AFPS

Peptides were synthesized on an automated flow system built in the Hartrampf laboratory, which is similar to the published AFPS system. Capitalized letters refer to L-amino acids. For all synthesis (referred to as standard AFPS protocol) the following settings were used for peptide synthesis: a flow rate of 20 ml min^−1^ for coupling and deprotection steps and a temperature of 90 °C (loop) for all canonical amino acids, except histidine and cysteine, which were coupled at room temperature and 90 °C (reactor). The standard synthetic cycle involves a first step of prewashing the resin at 90 °C for 60 s at 40 ml min^−1^. During the coupling step, three HPLC pumps are used: a 50 ml min^−1^ pump head delivers the activating agent, a second 50 ml min^−1^ pump head delivers the amino acid and a 5.0 ml min^−1^ pump head delivers *i*-Pr_2_NEt (neat). The 50 ml min^−1^ pump head delivered 0.398679 ml of liquid per pump stroke, the 5.0 ml min^−1^ pump head delivered 3.9239 × 10^−2^ ml of liquid per pump stroke.

All peptides were prepared by AFPS on NovaPEG Rink Amide resin (0.41 mmol g^−1^) and standard Fmoc/*t*-Bu-protected amino acids (0.40 M in DMF) were coupled using HATU (0.38 M in DMF) or PyAOP (0.38 M in DMF) with DIPEA (neat, 3.0 ml min^−1^) at a total flow rate of 20 ml min^−1^. For amino acids D, E, F, G, I, K, L, M, P, S, W and Y, a total volume of 6.4 ml of the ‘coupling solution’ (that is, amino acid (0.20 M), HATU or PyAOP (0.19 M) and DIPEA in DMF) was applied for each coupling. For amino acids A, C, H, N, Q, R, S, T and V, a total of 10.4 ml of ‘coupling solution’, was applied for each coupling. All amino acids except C and H were preheated at 90 °C during the activation step with HATU or PyAOP, whereas C and H were preactivated with PyAOP at room temperature. The removal of the Nα-Fmoc group was achieved using 20% piperidine with 1% formic acid in DMF at a flow rate of 20 ml min^−1^ and a total volume of 6.4 ml at 90 °C. Between each coupling and deprotection step, the resin was washed with DMF (32 ml) at 90 °C with a flow rate of 40 ml min^−1^. After completion of the peptide sequence, the resins were manually washed with DCM (3× 5 ml) and dried under reduced pressure.

## Online content

Any methods, additional references, Nature Portfolio reporting summaries, source data, extended data, supplementary information, acknowledgements, peer review information; details of author contributions and competing interests; and statements of data and code availability are available at 10.1038/s41557-026-02090-0.

## Supplementary information

Supplementary Information Supplementary Figs. 1–171 and Tables 1–5 and extended computational and experimental methods.

## Data Availability

All data used in this work are available via Zenodo at https://zenodo.org/records/14824562 (ref. ^41^).

## Code availability

The code for generating the data and training the models is freely available via GitHub at https://github.com/rxn4chemistry/AI4Aggregation.
