# Supplementary material for: Amino acid composition drives aggregation during peptide synthesis
Source: Nat Chem. 2026 Mar 20;18(4):677–85. doi: 10.1038/s41557-026-02090-0 (PMC13061631; doi:10.1038/s41557-026-02090-0)
Supplement: Supplementary file 1 — Supplementary Figs. 1–171 and Tables 1–5 and extended computational and experimental methods. [file 41557_2026_2090_MOESM1_ESM.pdf]

nature chemistry

Supplementary information https://doi.org/10.1038/s41557-026-02090-0

Amino acid composition drives aggregation during peptide synthesis

In the format provided by the authors and unedited

Contents

1 Dataset statistics 4
1.1 Dataset diversity 6
1.1.1 Levenshtein Distance for Quantifying Pairwise Sequence Similarity 6
1.1.2 Shannon Entropy for Quantifying Positional Sequence Diversity 6
2 Raw performances across models and Representations 7
2.1 Similarity between the train and test set 7
2.2 Summary of Models used 8
2.3 Performance on the combined dataset 9
57.8 ± 4.1 10
2.4 Shuffled dataset 10
2.5 Performance with Composition vector 11
2.6 Atomistic Representations 11
3 Explainability 13
4 Dipeptide Sequences (Motifs) 16
5 Analytical methods 17
5.1 Liquid Chromatography with High-Resolution Electrospray Ionization Mass Spectrometry (LC–MS) 17
5.2 Analytical Ultra-High Performance Liquid Chromatography (UHPLC) 17
6 Screening of the effect of sequence randomization on aggregation property of peptides 18
6.1 Peptide randomization method 18
6.2 Barstar[75–90] shuffling 18
6.2.1 Shuffling 1 18
6.2.2 Shuffling 2 20
6.2.3 Shuffling 3 22
6.2.4 Shuffling 4 24
6.2.5 Shuffling 5 25
6.3 hGH[176–191]F176Y shuffling 27
6.3.1 Original sequence 27
6.3.2 Shuffling 1 28
6.3.3 Shuffling 2 30
6.3.4 Shuffling 3 31
6.3.5 Shuffling 4 33
6.3.6 Shuffling 5 34
6.4 GLP-1 shuffling 36

6.4.1 Shuffling 1 ... 36
6.4.2 Shuffling 2 ... 38
6.4.3 Shuffling 3 ... 39
6.4.4 Shuffling 4 ... 41
6.4.5 Shuffling 5 ... 42
6.5 MYC[123–143] shuffling ... 44
6.5.1 Shuffling 1 ... 44
6.5.2 Shuffling 2 ... 46
6.5.3 Shuffling 3 ... 47
6.5.4 Shuffling 4 ... 49
6.5.5 Shuffling 5 ... 50
6.6 NBDY [41–68] shuffling ... 52
6.6.1 Shuffling 1 ... 52
6.6.2 Shuffling 2 ... 54
6.6.3 Shuffling 3 ... 55
6.6.4 Shuffling 4 ... 57
6.6.5 Shuffling 5 ... 58
6.7 GHRH shuffling ... 60
6.7.1 Original sequence ... 60
6.7.2 Shuffling 1 ... 62
6.7.3 Shuffling 2 ... 63
6.7.4 Shuffling 3 ... 65
6.7.5 Shuffling 4 ... 66
6.7.6 Shuffling 5 ... 68
6.8 MYC[421–439] shuffling ... 70
6.8.1 Original sequence ... 70
6.8.2 Shuffling 1 ... 71
6.8.3 Shuffling 2 ... 73
6.8.4 Shuffling 3 ... 74
6.8.5 Shuffling 4 ... 76
6.8.6 Shuffling 5 ... 77
6.9 PCP-4[43–62] shuffling ... 80
6.9.1 Original sequence ... 80
6.9.2 Shuffling 1 ... 81
6.9.3 Shuffling 2 ... 83
6.9.4 Shuffling 3 ... 84

6.9.5 Shuffling 4 ... 86

6.9.6 Shuffling 5 ... 87

7 Rational incorporation of pseudoproline to reduce aggregation ... 90

7.1 Example 1: hGH[176–191] ... 90

7.1.1 Original sequence ... 90

7.1.2 Use of suggested pseudoproline ... 91

7.1.3 Use of the non-optimal pseudoproline ... 94

7.2 Example 2: GB1 ... 96

7.2.1 Original sequence ... 96

7.2.2 Use of suggested pseudoproline ... 97

7.2.3 Use of 2nd suggested pseudoproline ... 100

7.2.4 Use of 3rd suggested pseudoproline ... 102

1 Dataset statistics

The dataset used in this work contains a total of 539 peptide sequences. In total 420 are sourced from Mohapatra et. al.[20] and 119 stem from the internal UZH dataset. The average peptide length is 14.43 with a standard deviation of 5.20 amino acids. The peptide length distribution is shown in Figure 1A and the amino acid distribution across all peptides is displayed in Figure 1B.

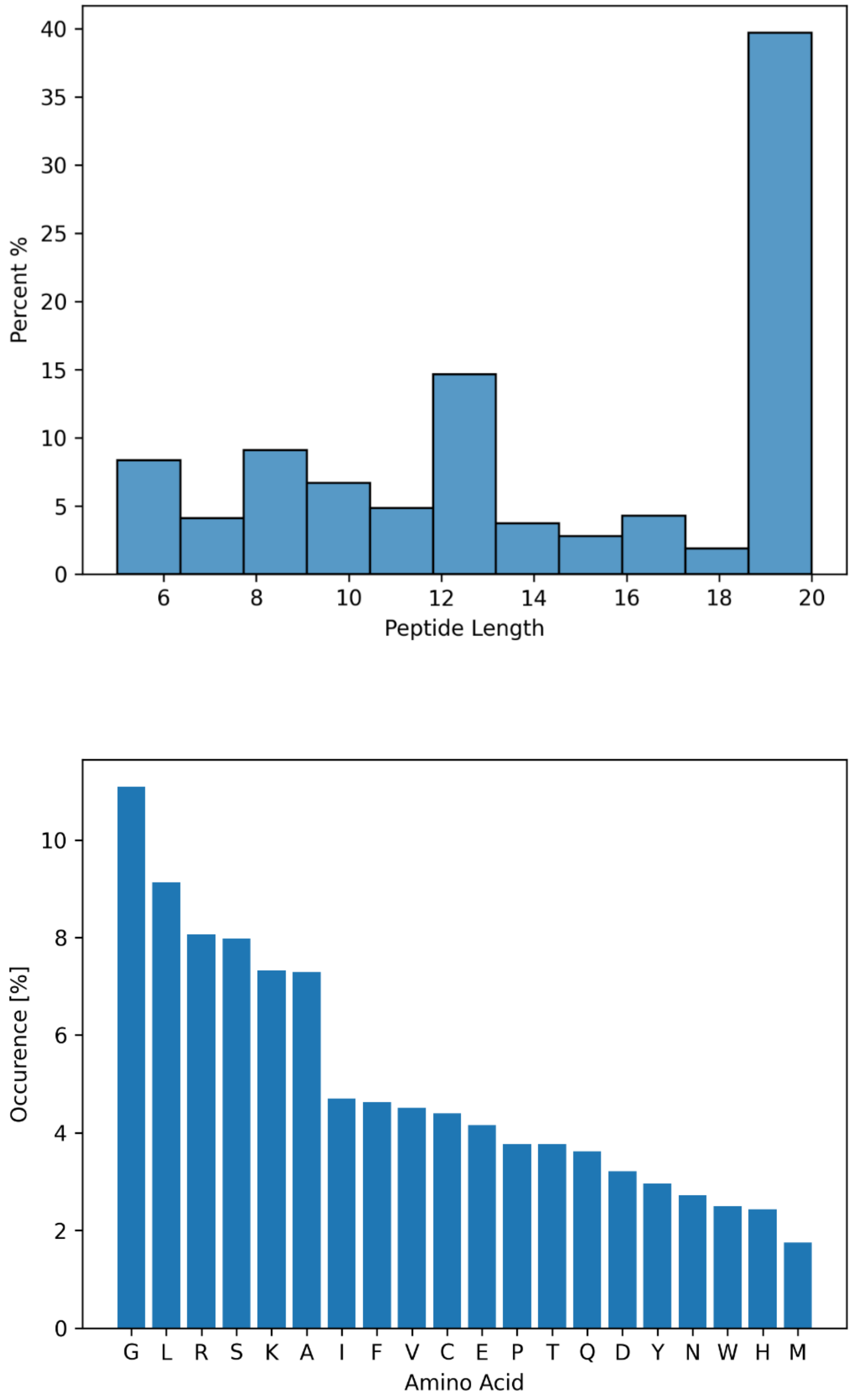

SI Figure 1. *Dataset statistics. Top: Distribution of peptide lengths in the dataset. Approximately 40% of peptides were longer than 20 amino acids and were shortened to 20 amino acids. Bottom: Amino acid distribution across all peptides.*

1.1 Dataset diversity

1.1.1 Levenshtein Distance for Quantifying Pairwise Sequence Similarity

To assess the dissimilarity between peptide sequences of varying lengths, we calculated the Levenshtein distance between all unique sequence pairs. The Levenshtein distance is a string metric that quantifies the minimum number of single-character edits, specifically, insertions, deletions, or substitutions, required to transform one sequence into another.

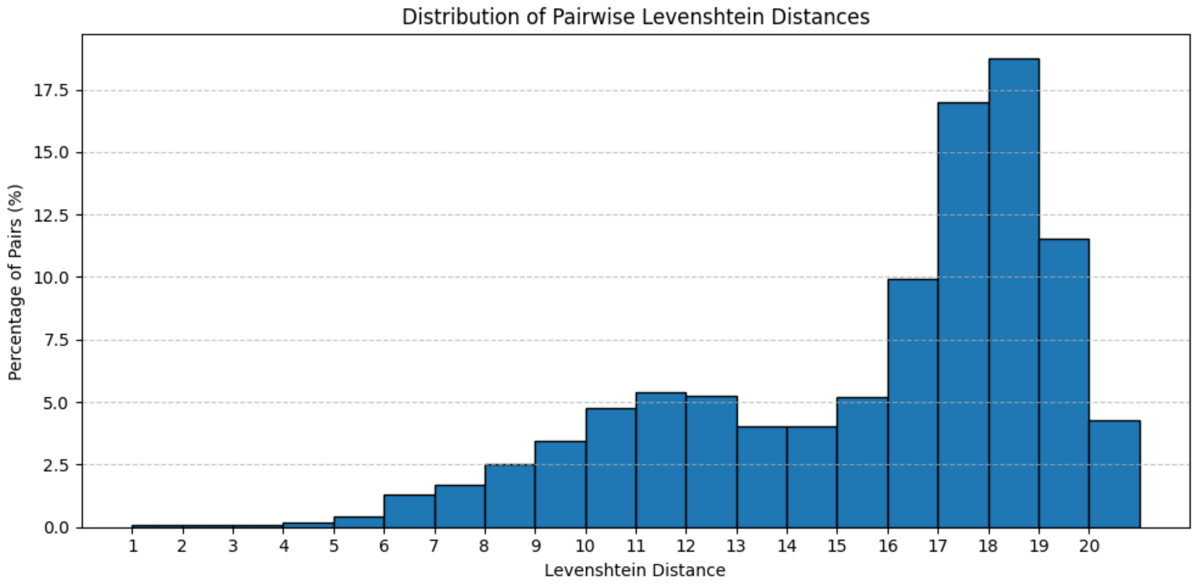

SI Figure 2 *Pairwise Levenshtein distances of our dataset. The median value is 17*

1.1.2 Shannon Entropy for Quantifying Positional Sequence Diversity

To quantify the positional variability of amino acids within our peptide dataset, we calculated the Shannon entropy at each residue position. This metric captures how evenly distributed amino acid identities are at a given position across the aligned sequences.

For a given position i, the Shannon entropy H_i is defined as:

$$H_i = -\sum_{\mathbf{a} \in \mathbf{A}} p_{i,a} \log_2 p_{i,a}$$

where:

- A is the set of 20 standard amino acids,
- $p_{i,a}$ is the frequency of amino acid a at position i in the sequence alignment.

Entropy values range from:

- 0, when a single amino acid dominates a position (no variability),
- to a maximum of $\log_2(20) \approx 4.32$, when all 20 amino acids are equally represented (maximum variability).

In this context, Shannon entropy provides a position-wise measure of sequence diversity, helping to identify conserved or variable regions across the peptide set.

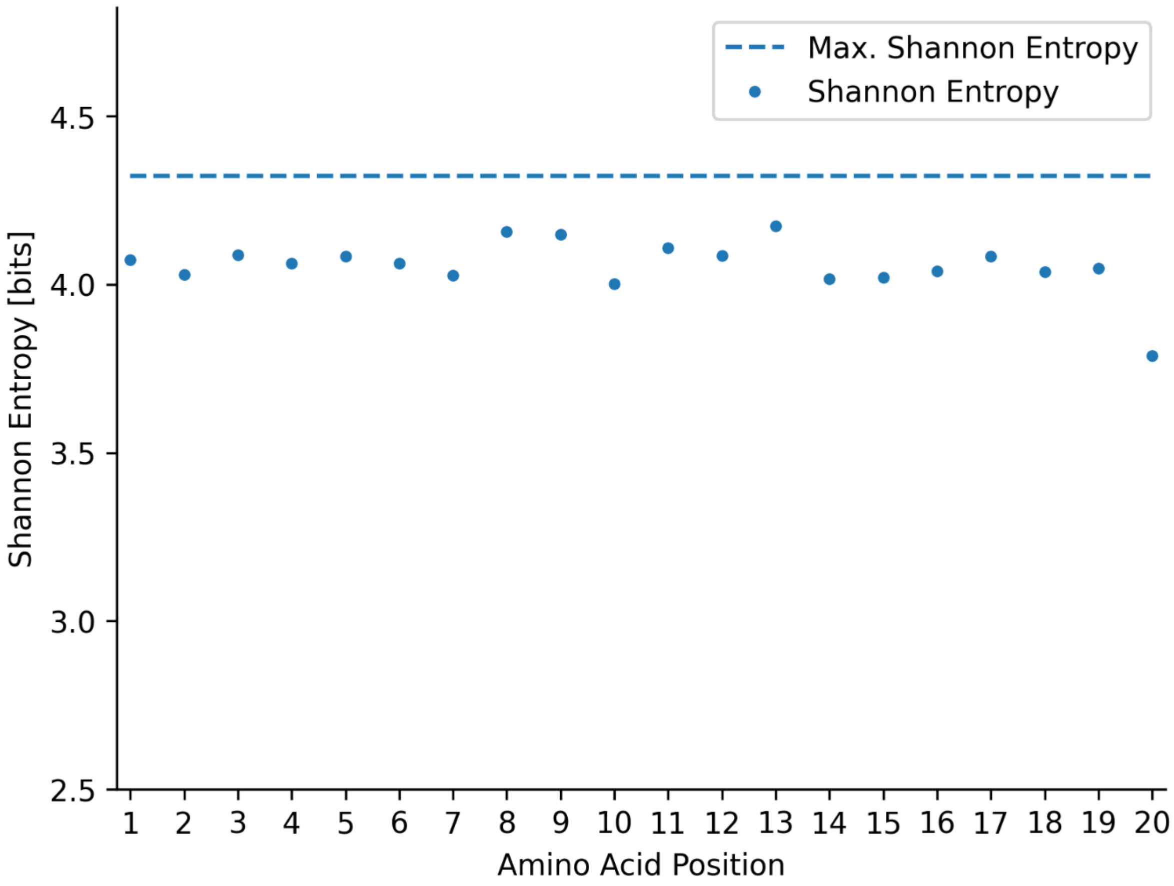

SI Figure 3 *Shannon entropy for our dataset.*

2 Raw performances across models and Representations

2.1 Similarity between the train and test set

We evaluated the similarity between the train and test sets by measuring the minimum Levenshtein distance for each peptide in the test compared to the peptides in the train set. The Levenshtein distance measures how many single amino acid operations (i.e. insertions, deletions, or substitutions) are required to transform one peptide sequence into another. Figure 2 shows the distribution of the minimum distances, illustrating the degree of sequence dissimilarity between the test samples and their closest counterparts in the training set. Most test sequences have a meaningful edit distance from any training sequence, with a median minimum Levenshtein distance of 4. As we limit the maximum peptide length to 20 amino acids this means that for at least half of the sequences in the test set more than 20% of amino acids are different and that our test set provides an evaluation of the model's generalisation capabilities rather than simply testing the memorisation capabilities of the models.

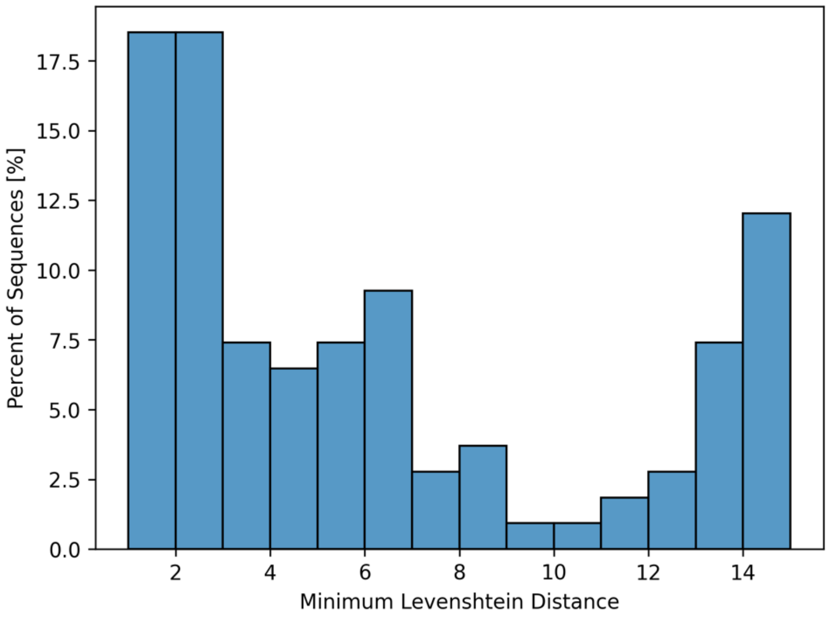

SI Figure 4. *Distribution of the Levenshtein distance of each peptide in the test set to the most similar peptide in the train set.*

2.2 Summary of Models used

In the following section we briefly describe the different models used in this study.

ESM 2.0 is a transformer-based protein language model able to learn representations of amino acid sequences through self-supervised pre-training on millions of protein sequences. This pretraining procedure enable the model to learn both evolutionary and structural information.

BERT is a transformer-based language model pretrained on masked word prediction tasks. This model has learned to capture semantic relationships in natural language and is commonly used for a variety of sequence classification tasks.

Hive Cote 2.0 is an ensemble time series classifier that combines multiple classifiers (including shapelet-based, dictionary-based, and frequency-based approaches) in a unified framework. One of the state-of-the-art models for time series classification.

Weasel is a time series classification algorithm that transforms time series into word-like patterns using symbolic representations and builds a bag-of-patterns model. Also, one of the state-of-the-art models for time series classification.

Time Forest is a time series classifier that extract features from random interval of the time series and builds a forest of decision trees (estimators) based on these features.

XGBoost is a gradient boosting framework that builds an ensemble of decision trees one after another, with each new tree correcting the errors made by the previous trees. Gradient descent is used to minimise the loss.

Random Forest is an ensemble method that builds multiple decision trees on random subsets of the data and features, then aggregates their predictions through voting.

Gaussian Processes is a probabilistic model that defines a distribution over functions, making predictions by conditioning this distribution on observed data points.

KNN is a simple learning algorithm that classifies new data points based on the majority class among their k nearest neighbours in the feature space.

2.3 Performance on the combined dataset

We assessed the performance of various representations and models on the peptide dataset. Each experiment was carried out with five-fold cross validation and reporting the mean and standard deviation of the accuracy. We chose accuracy for this problem as the dataset is balanced with 49.9% aggregating and 50.1% non-aggregating sequences. The raw data is shown in Table 1 below.

SI Table 1. *Raw performance data of the various representations and models.*

Problem Level	Representation	Model	Accuracy [%]
Whole Sequence	Text	ESM2.0 (35M)	62.1 ± 6.1
		Bert (Base, Pretrained)	59.6 ± 4.3
		Bert (Base, Trained from scratch)	60.6 ± 11.3
	Sequence (Timeseries)	HIVE-COTE-2.0	61.4 ± 6.9
		Time Forest	60.7 ± 6.0
		WEASEL	60.6 ± 3.0
	Sequence	Random Forest	59.9 ± 3.0
		XGBoost	58.0 ± 3.5
		KNN	57.3 ± 4.5
		Gaussian Processor	56.5 ± 4.4
	One-Hot Encoding	Random Forest	62.8 ± 2.7
		XGBoost	58.9 ± 2.6
		KNN	60.8 ± 3.5
		Gaussian Processor	61.4 ± 4.2
	Fingerprint	Random Forest	62.1 ± 5.7
		XGBoost	59.9 ± 3.4
		KNN	59.9 ± 1.8
		Gaussian Processor	57.1 ± 4.4
Step Wise	Sequence	Random Forest	59.9 ± 2.9
		XGBoost	60.1 ± 2.0
		KNN	58.2 ± 2.5
		Gaussian Processor	60.1 ± 5.9
	One-Hot Encoding	Random Forest	59.3 ± 4.5
		XGBoost	58.3 ± 6.4
		KNN	54.7 ± 2.0
		Gaussian Processor	54.0 ± 7.9
	Fingerprint	Random Forest	58.4 ± 4.6
		XGBoost	56.5 ± 4.7
		KNN	58.6 ± 3.7
		Gaussian Processor	57.8 ± 1.7

	Mohapatra	Complete	61.8 ± 2.5
		Minimal	57.8 ± 4.1

2.4 Shuffled dataset

We reused the same experimental setup as in section 2 and shuffled the order of the peptide sequences. To avoid bias we repeated each experiment 100 times with different shuffling and train/test splits. The accuracy is reported in Table 2.

SI Table 2. *Raw performance data of the various representations and models on shuffled data.*

Problem Level	Representation	Model	Accuracy [%]
Whole Sequence Shuffled	Sequence	Random Forest	59.6 ± 3.2
		XGBoost	57.7 ± 3.8
		KNN	57.7 ± 3.5
		Gaussian Processor	55.2 ± 4.0
	One-Hot Encoding	Random Forest	60.2 ± 3.5
		XGBoost	57.0 ± 3.9
		KNN	58.1 ± 3.8
		Gaussian Processor	61.3 ± 3.7
	Fingerprint	Random Forest	61.0 ± 3.1
		XGBoost	57.9 ± 3.6
		KNN	58.5 ± 3.6
		Gaussian Processor	57.1 ± 4.0

In addition, we also performed shuffling experiments by training models on non-shuffled data and subsequently evaluating on a shuffled training set. To obtain a statistically significant measure of the performance we evaluate each model with five-fold cross validation and for each trained model we shuffled the test set 10 times. Both performance on the standard as well as shuffled test set are reported below in Table 3. There is no significant difference when in terms of the performance of the model when evaluating on the standard or shuffled test set.

SI Table 3. *Raw performance data of different models when training on non-shuffled data and evaluating on both a shuffled and non-shuffled test set.*

Problem Level	Representation	Model	Test Set	Accuracy [%]
Whole Sequence	Sequence	Random Forest	Standard	59.9 ± 3.0
			Shuffled	59.3 ± 5.8
		XGBoost	Standard	58.0 ± 3.5
			Shuffled	55.4 ± 4.7
		KNN	Standard	57.3 ± 4.5
			Shuffled	58.0 ± 5.0
		Gaussian Processor	Standard	56.6 ± 4.5

			Shuffled	54.9 ± 5.1

2.5 Performance with Composition vector

We assessed the performance of various models using a simplified amino acid composition-based representation. To this end, we constructed a 20-dimensional vector for each peptide with each value corresponding to the percentage of a given amino acid in the peptide. Training the Scikit-Learn models on this representation yielded comparable performance to all other representations.

SI Table 4. *Raw performance data of the various representations and models on shuffled data.*

Problem Level	Representation	Model	Accuracy [%]
Whole Sequence	Composition vector	Random Forest	62.8 ± 2.3
		XGBoost	59.5 ± 1.9
		KNN	54.7 ± 5.1
		Gaussian Processor	52.1 ± 4.1

2.6 Atomistic Representations

We also assessed the performance of the model when using more atomistic representations. For language models we investigated using the SMILES representation of the peptide sequences and infusing information about the protecting groups and whether an amino acid is aliphatic or aromatic. When training on the SMILES of the peptide we finetuned the MolFormer model [cite: https://www.nature.com/articles/s42256-022-00580-7] to classify whether a peptide aggregates or not. The same parameters used for finetuning the other language models (ESM 2.0 and BERT) were reused here (see Methods section for more detail). To infuse more information into the coarse-grained description of the amino acids we added tokens describing the protecting group as well as whether a given protected amino acid is aliphatic or aromatic. "|" is used as a separating character. An example is given below:

Original Amino Acid Sequence:

PLERLY

Including protecting group information:

P | L | E tBu | R Pbf | L | Y tBu |

Including aliphatic/aromatic character:

P ali | L ali | E ali | R aro | L ali | Y aro |

Including both:

P ali | L ali | E tBu ali | R Pbf aro | L ali | Y tBu aro |

In addition, we investigated whether adding further information to the composition vector improves performance. Four different approaches were assessed: 1. Adding vector with percentage of amino acids in the peptide being protect with either Pbf, Trt, Ot-Bu, Boc, tBu or no protecting group. 2. The percentage of protected amino acids being aliphatic or aromatic. 3. The polar surface area of the peptide. 4. The number of hydrogen bond donors and acceptors. Lastly, we also evaluated the performance when combining all four approaches. For all representations we trained the classical machine learning models (XGBoost, Random Forest, KNN-Classifier and Gaussian Processes Classifier) on the same data with five-fold cross-validation as for all other experiments.

SI Table 5. *Raw performance data of the test of different atomistic representations*

Problem Level	Representation	Added Data	Model	Accuracy [%]
Whole Sequence	Text	SMILES	MolFormer (XL, Pretrained)	61.5 ± 4.9
		Protecting Groups	Bert (Base, Pretrained)	62.3 ± 3.5
		Aliphatic/Aromatic	Bert (Base, Pretrained)	64.9 ± 4.6
		Protecting Groups + Aliphatic/Aromatic	Bert (Base, Pretrained)	62.7 ± 3.2
	Composition Vector	Protecting Groups	Random Forest	60.7 ± 4.0
			XGBoost	59.0 ± 2.5
			KNN	53.8 ± 4.4
			Gaussian Processor	53.0 ± 4.2
		Aliphatic/Aromatic	Random Forest	63.3 ± 6.2
			XGBoost	59.0 ± 2.9
			KNN	53.2 ± 6.4
			Gaussian Processor	52.5 ± 4.4
		Polar Surface Area	Random Forest	64.0 ± 4.3
			XGBoost	62.1 ± 4.0
			KNN	49.0 ± 4.7
			Gaussian Processor	54.4 ± 3.1
		Hydrogen Bond Donor/Acceptor	Random Forest	60.9 ± 4.0
			XGBoost	58.1 ± 4.0
			KNN	52.3 ± 3.9
			Gaussian Processor	52.9 ± 4.4
		Protecting Groups + Aliphatic/Aromtic + Polar Surface Area + Hydrogen Bond Donor/Acceptor	Random Forest	61.8 ± 4.2
			XGBoost	61.6 ± 6.2
			KNN	51.9 ± 4.1
			Gaussian Processor	54.7 ± 5.5

2.5 Labelling experiments

When running experiments with the step wise modelling approach, we labelled all synthesis steps before the onset of aggregation (point of aggregation) as non-aggregating (False) and all steps after the onset as aggregating (True). However, the sequence before the onset of aggregation should be most informative for the model to decide whether aggregation occurs or not. We hypothesised that labelling the sequence before the onset of aggregation as aggregating (True), the models may be guided to focus only on the important part of the sequence. This hypothesis was systematically evaluated by labelling 0-10 amino acids before the point of aggregation as aggregating as well as 0-10 amino acids after the point of aggregation and training different models and representations for each combination. The results are shown below in SI Figure 3.

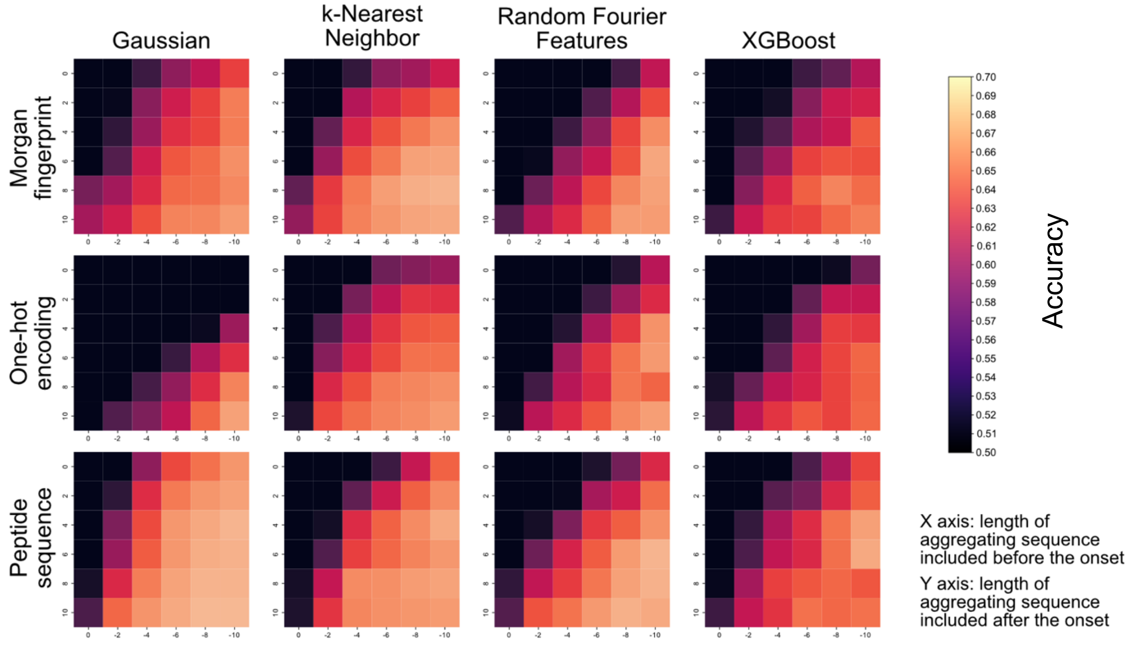

SI Figure 5. *Performance of the models with the stepwise representation, with a different window of the sequence labelled as aggregating. We labeled 0-10 amino acids before the point of aggregation (X-axis) and 0-10 amino acids after the point of aggregation (Y-axis) and investigated the accuracy of the models. We could observe that the accuracy mainly depended on the length of the sequence, and less on its position.*

3 Explainability

The impact of each amino acid on the prediction of the model was investigated. For these experiments, an XGBoost classifier was trained on 50 different splits of the data. Each peptide was represented with the composition vector and SHAP values were used to interpret the prediction of the model. SHAP values measure how much each input feature contributes to the prediction of the model. In our case, a positive SHAP value is associated with a higher likelihood that the model is going to predict the given peptide aggregates and the opposite is true for a negative SHAP value. For each amino acid, we plotted the amino acid occurrence in percent against the average SHAP value. The plots can be found in Figure 3 and 4.

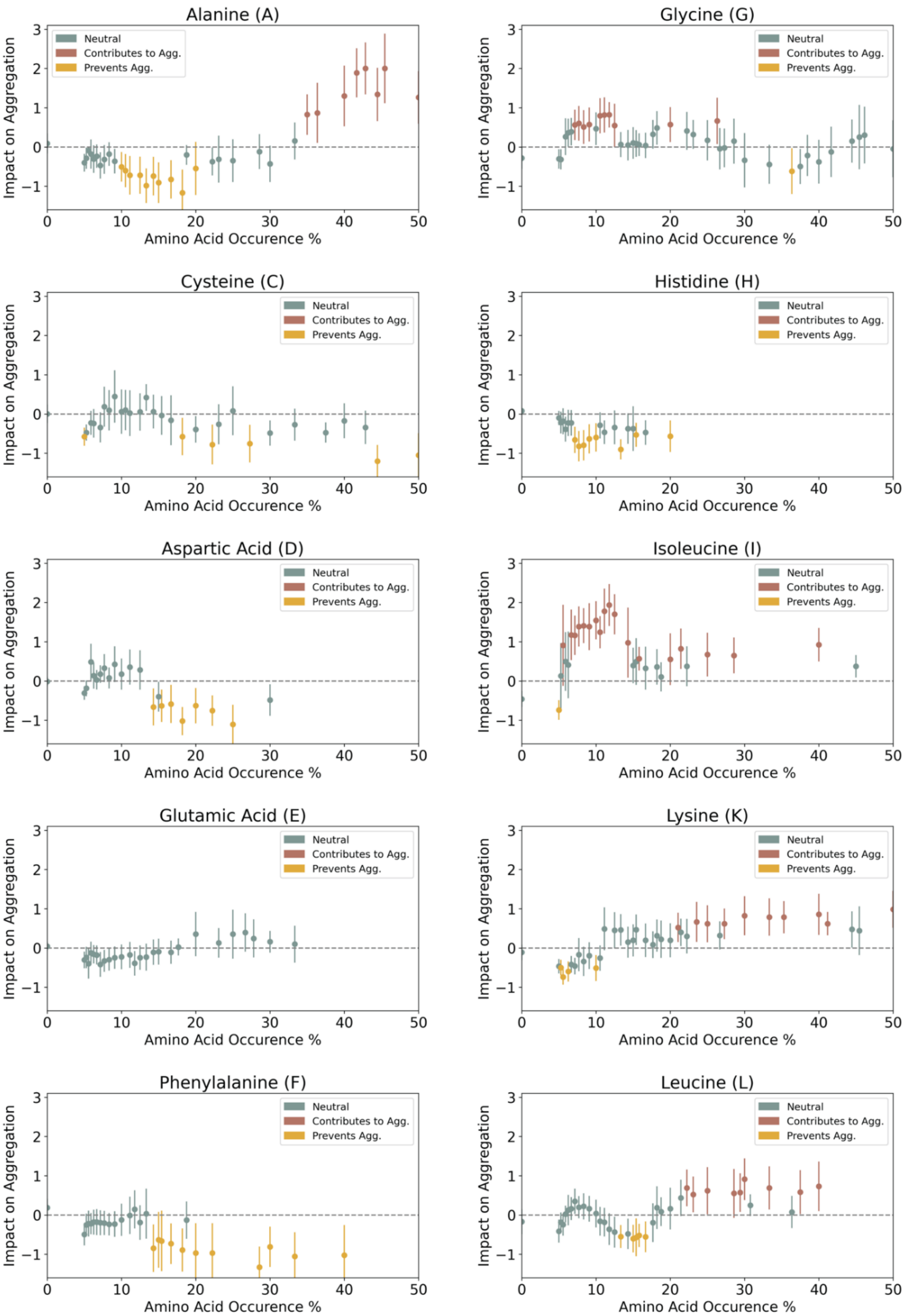

SI Figure 6. *Amino acid occurrence vs impact on the model's prediction for alanine, cysteine, aspartic acid, glutamic acid, phenylalanine, glycine, histidine, isoleucine, lysine, and leucine.*

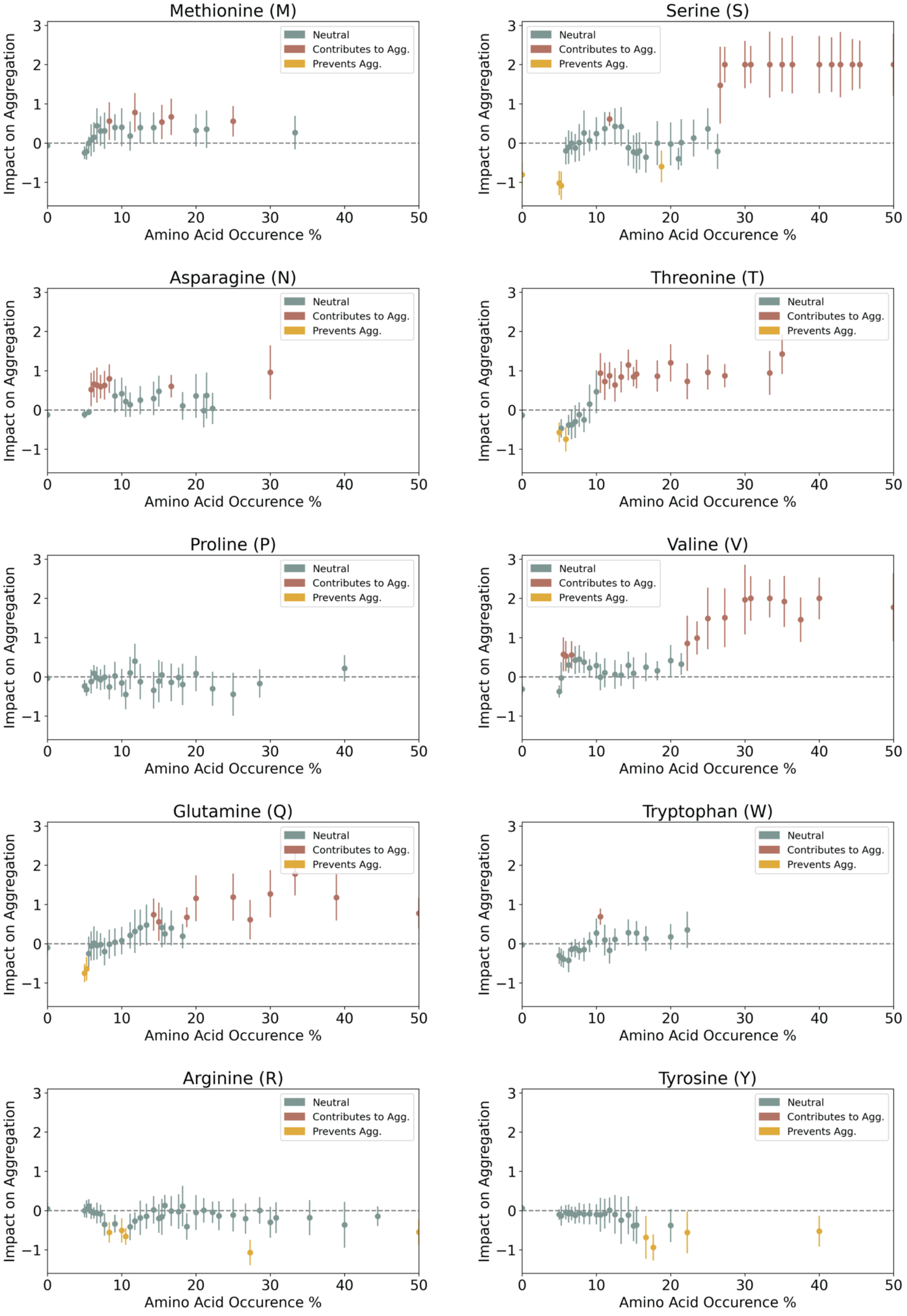

SI Figure 7. *Amino acid occurrence vs impact on the model's prediction for methionine, asparagine, proline, glutamine, arginine, serine, threonine, valine, tryptophan, and tyrosine.*

4 Dipeptide Sequences (Motifs)

Using the same techniques that were used to explain the impact of different amino acids, we can go beyond probing the importance of single amino acids and investigate amino acid patterns. The same composition vector from section 5 was used again, but the occurrence of dipeptide motifs to the vector was appended. A dipeptide motif is defined as two amino acids next to each other in a peptide sequence. For example, in the peptide sequence ASTR, ST would be a motif just as much as AS would be one. As the model's interpretability highly corresponds with the amount of data used to train the model, only motifs occurring more than 20 times in the dataset were considered, which led to a total of 83 unique motifs. The impact of the motifs is shown in Figure 5 with GS and LL being most strongly correlated with aggregation.

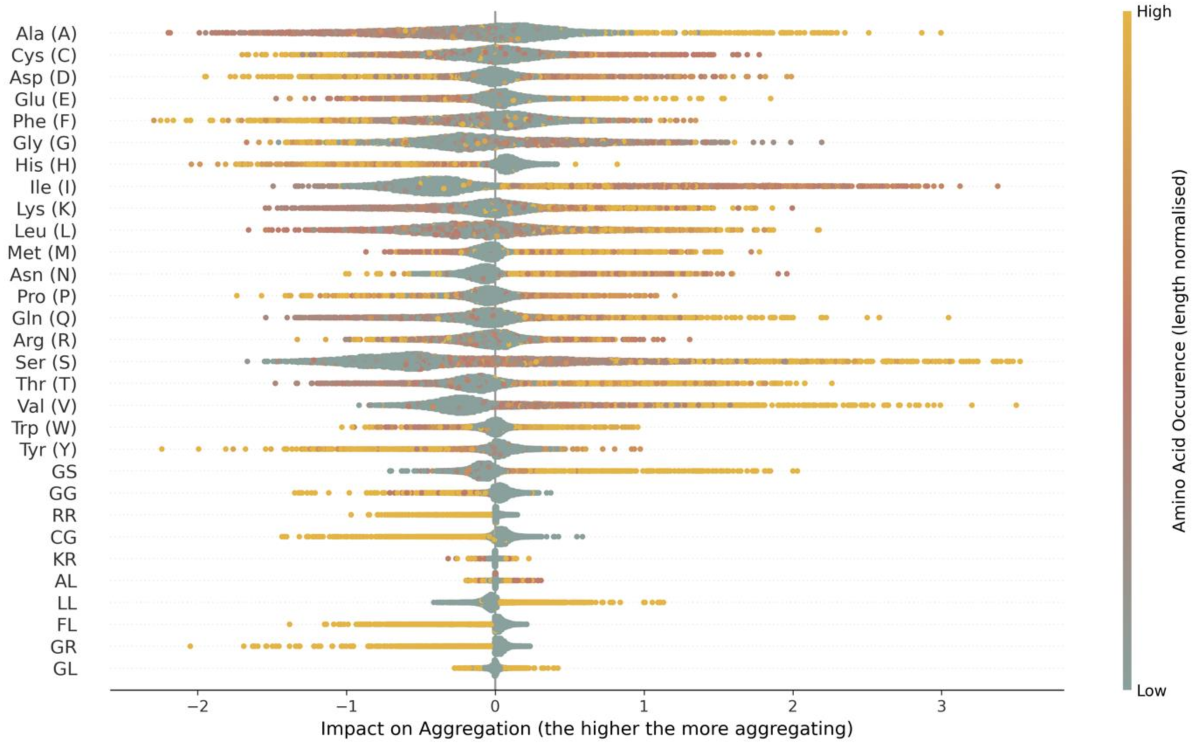

SI Figure 8. *The impact on the model prediction of the individual amino acids as well as dipeptide motifs.*

5 Analytical methods

5.1 Liquid Chromatography with High-Resolution Electrospray Ionization Mass Spectrometry (LC–MS)

For determination of peptide masses and purity by LC–MS, the filtered peptide solution was diluted in 10–50% acetonitrile (MeCN) in water with 0.1% TFA (60–500 μL) to a final concentration of approximately 0.1 mg/mL. The samples were analyzed on an Acquity UPLC (Waters, Milford, USA) connected to an Acquity el diode array detector and a Synapt G2HR-ESI-QTOF-MS (Waters, Milford, USA).

For standard analysis of all peptide samples, LC–MS spectra were recorded on an Acquity BEH C8 HPLC column (2.1 × 100 mm, 1.7 μm particle size, Waters) kept at 30 °C at a flow rate of 0.4 mL/min with UV detection at 190–300 nm. A binary solvent system was used, wherein Solvent A was water containing 0.02% formic acid and 0.04% TFA, and Solvent B was MeCN containing 0.04% formic acid and 0.02% TFA. The LC–MS gradient used was as follows: isocratic at 3% Solvent B for 3 min, then linear gradient of 3–95% Solvent B over 9 min, followed by isocratic at 95% Solvent B for 1 min.

UV spectra recorded at 1.2 nm resolution and 20 points s^{-1}; ESI: positive ionization mode, capillary voltage 3.0 kV, sampling cone 40V, extraction cone 4V, N_2 cone gas 4 L h^{-1}, N_2 desolvation gas 800 L min^{-1}, source temperature 120 °C; mass analyzer in resolution mode: mass range 150–3000 *m/z* with a scan rate of 1 Hz; mass calibration to <2 ppm within 50–2500 *m/z* with a 5.0 mM aq. soln. of HCO_2Na, lock masses: *m/z* 195.0882 (caffein, 0.7 ng mL^{-1}) and 556.2771 (Leucine-enkephalin, 2 ng mL^{-1}).

All mass spectra show deconvoluted masses from the raw *m/z* values, calculated using Mestrelab Research S.L.© MestReNova v. 14.1 Mnova MS Suite. Purity based on LC–MS was calculated by calculating the Area Under the Curve (AUC) of desired product peak as a percentage of the AUC of all peaks (within 2–8 min) of the absorbance chromatogram (λ = 214 nm).

5.2 Analytical Ultra-High Performance Liquid Chromatography (UHPLC)

For determination of peptide masses by UHPLC, the filtered peptide solution was diluted in 10–50% acetonitrile (MeCN) in water with 0.1% TFA (500 μL) to a final concentration of approximately 0.1 mM. The samples were analyzed on an Agilent 1290 Infinity II Series UHPLC, which is connected to an Agilent 1260 Infinity II Series VWD, and an Agilent 6546 LC/Q-TOF. Separation was carried out on an Agilent Poroshell 300SB-C8 HPLC column (5 μm particle size, 2.1 × 75 mm) which was at 50 °C, with a sample injection volume of 5 μL. The elution was performed at a flow rate of 0.8 mL/min with solvent A: H2O + 0.1% formic acid and solvent B: MeCN + 0.1 formic acid with the following UHPLC gradient: gradient: isocratic at 5% Solvent B for 1.5 min, followed by a linear gradient of 5–95% Solvent B over 5 min, followed by isocratic at 95% Solvent B for 1 min.

6 Screening of the effect of sequence randomization on aggregation property of peptides

6.1 Peptide randomization method

To have reproducible and unbiased randomization across all sequences, the built-in random function of python was applied with reproducible random seeds using the following code:

```
import random

sequence = 'YLRIVQCRSVEGSCGF'

for i in range(4):
    random.seed(i)
    sequence_shuffled = list(sequence)
    random.shuffle(sequence_shuffled)
    print(f"{i+1}: {''.join(sequence_shuffled)}\n")
```

6.2 Barstar[75–90] shuffling

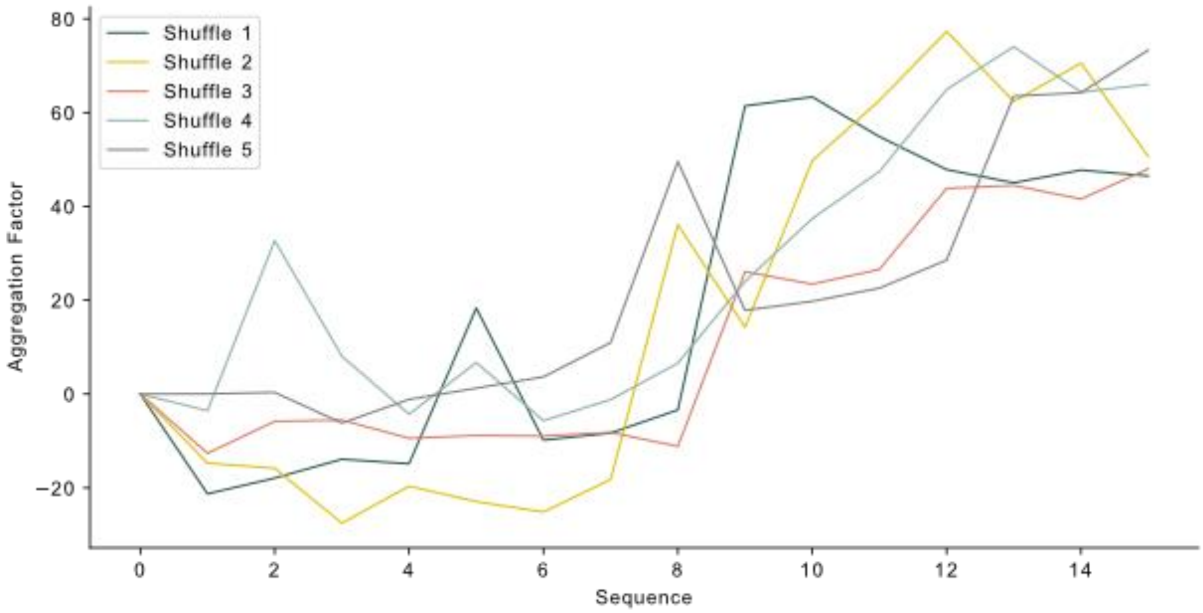

SI Figure 9. Plot of aggregation factor values for the different shuffled barstar[75–90] fragments.

6.2.1 Shuffling 1

H_2N-ILARDEATIG CKFESI-$CONH_2$

The peptide shuffling 1 of barstar[75–90] was synthesized on commercially available Novabiochem® NovaPEG Rink Amide resin (0.41 mmol/g, 101.9 mg, 42 µmol) using the standard AFPS protocol. (**SI Figure 10**) Total synthesis time to afford resin-bound peptide was approximately 0.8 h. Cleavage of the peptidyl-resin (16.8 mg, approx. 6.9 µmol) afforded the crude peptide as a colorless solid (4.2 mg, 42% purity by LCMS [**SI Figure 11**], 40% purity by UHPLC [**SI Figure 12**]).

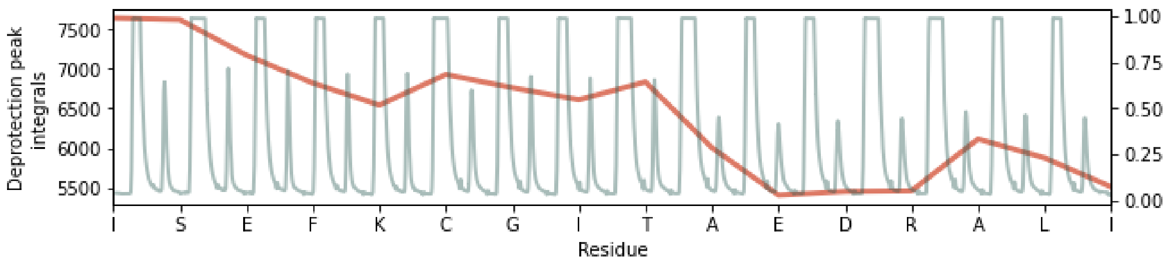

SI Figure 10. UV trace (λ = 310 nm) from AFPS of shuffling 1 of barstar [75–90] (green) and deprotection peak integrals (red).

LC-MS of crude

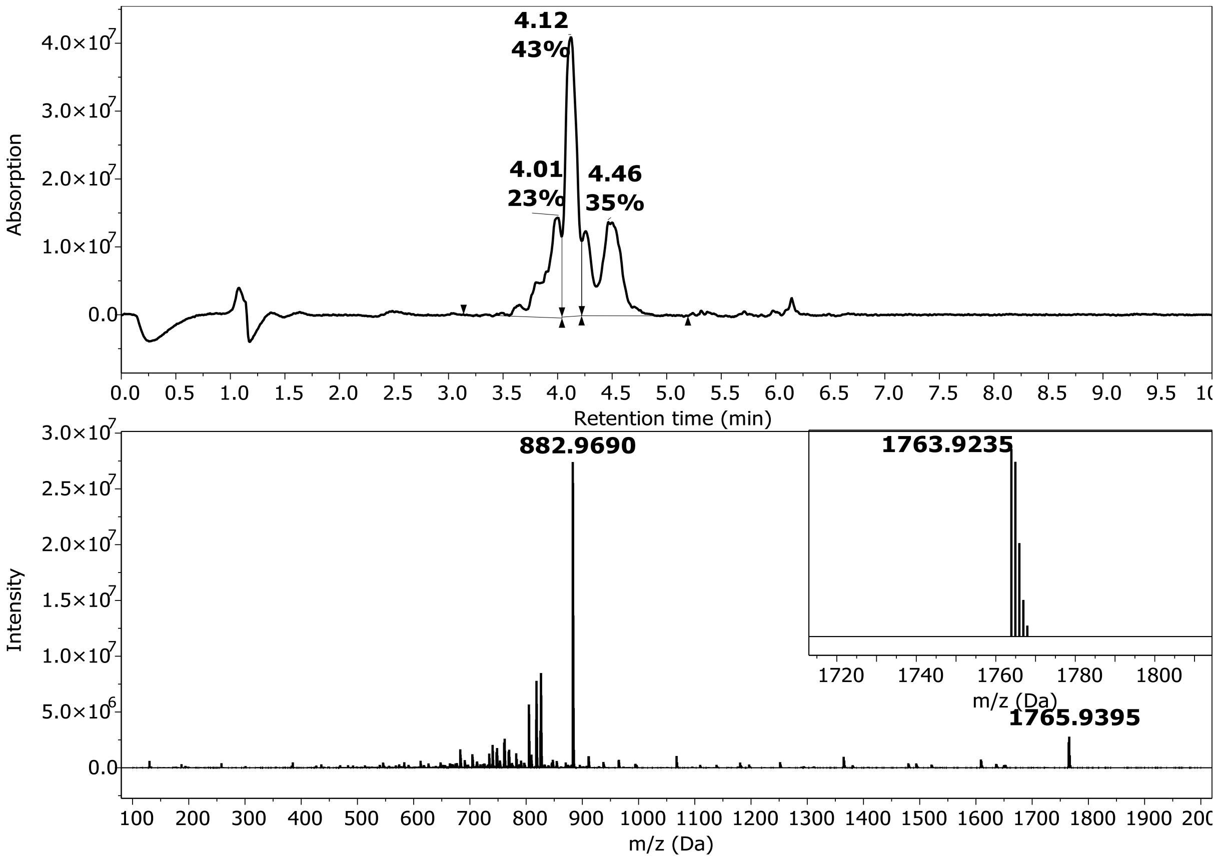

SI Figure 11. LCMS Profile of crude Top: Absorbance chromatogram of shuffling 1 of barstar[75–90] Rt 4.12 min, 43% purity Bottom: ESI-TOF spectrum found within Rt 2–8 min (insert: deconvoluted masses). Monoisotopic mass (ESI+) calcd. for $C_{77}H_{129}N_{21}O_{24}S$ 1763.9240, found 1763.9235.

UHPLC

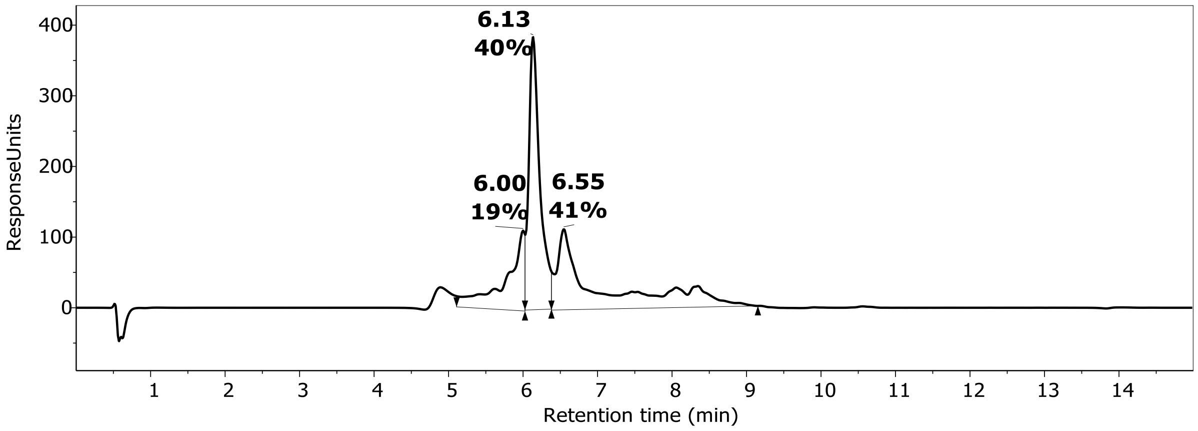

SI Figure 12. UHPLC profile of crude Rt 6.13 min, 40% purity based on Area Under Curve (AUC) at λ = 214 nm.

6.2.2 Shuffling 2

H_2N- EIFLEAACGT SRIIDK-$CONH_2$

The peptide shuffling 2 of barstar[75–90] was synthesized on commercially available Novabiochem® NovaPEG Rink Amide resin (0.41 mmol/g, 100.5 mg, 41 µmol) using the standard AFPS protocol. (**SI Figure 13**) Total synthesis time to afford resin-bound peptide was approximately 0.8 h. Cleavage of the peptidyl-resin (17.0 mg, approx. 7.0 µmol) afforded the crude peptide as a colorless solid (3.0 mg, 21% purity by LCMS [**SI Figure 14**], 18% purity by UHPLC [**SI Figure 15**]).

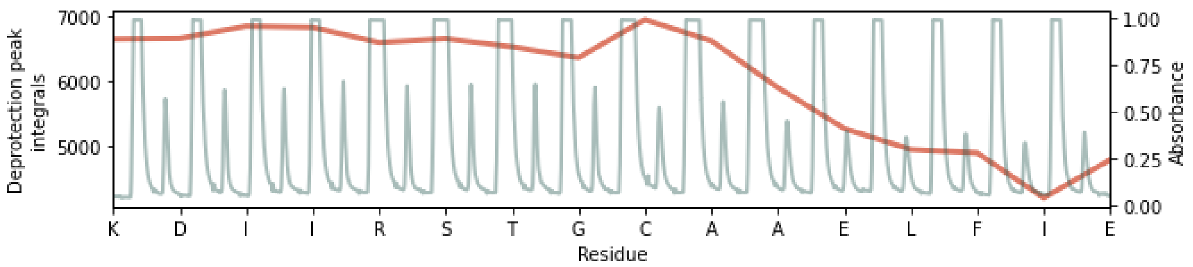

***SI Figure 13**. UV trace (λ = 310 nm) from AFPS of shuffling 2 of barstar [75–90] (green) and deprotection peak integrals (red).*

LC-MS of crude

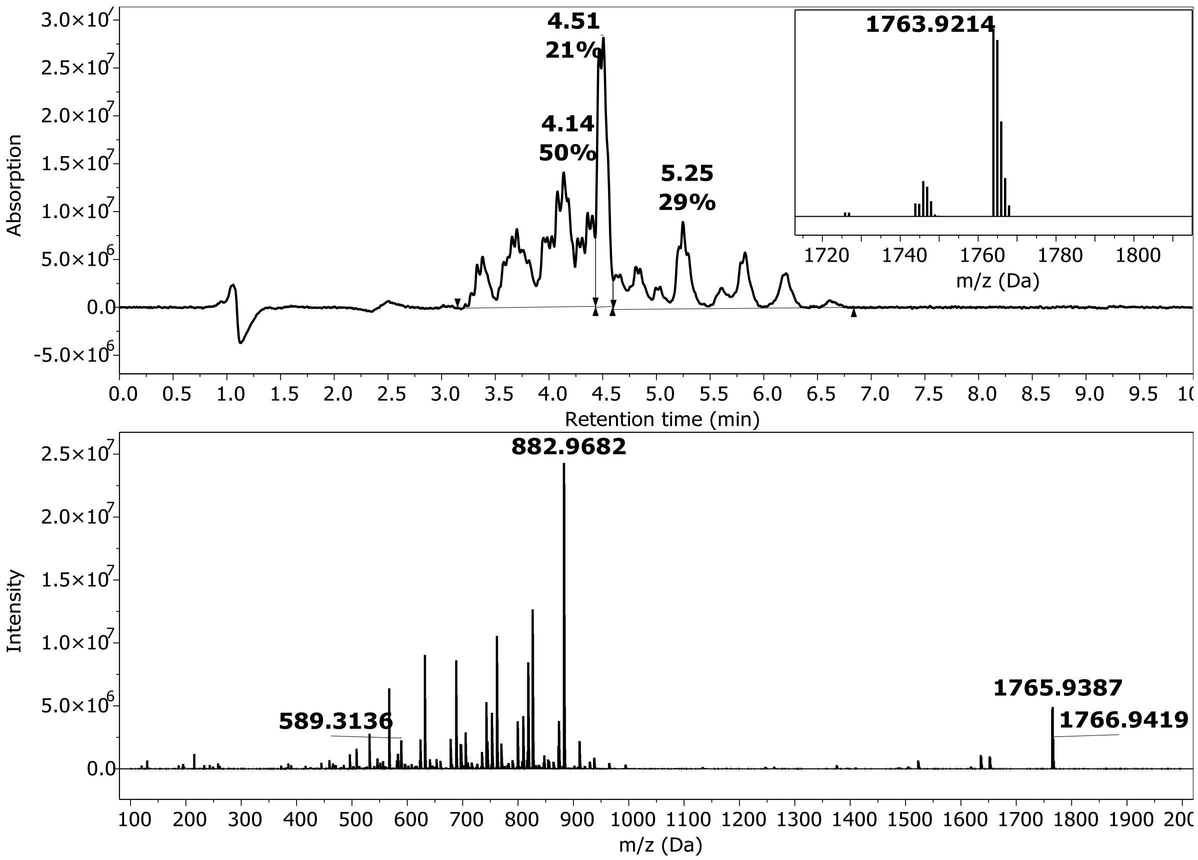

SI Figure 14. *LCMS Profile of crude Top: Absorbance chromatogram of shuffling 2 of barstar[75–90] Rt 4.51 min, 21% purity Bottom: ESI-TOF spectrum found within Rt 2–8 min (insert: deconvoluted masses). Monoisotopic mass (ESI+) calcd. for* $C_{77}H_{129}N_{21}O_{24}S$ *1763.9240, found 1763.9214.*

UHPLC

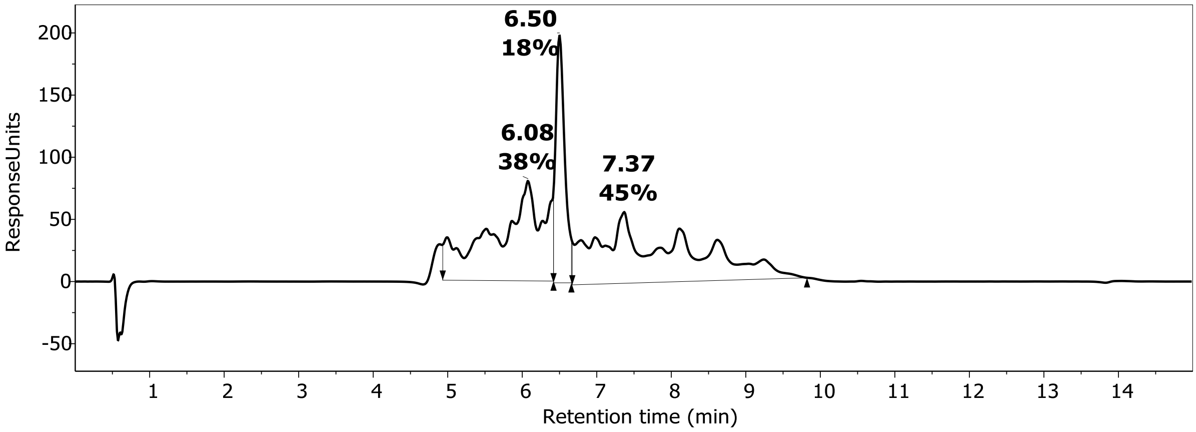

***SI Figure 15**. UHPLC profile of crude Rt 6.50 min, 18% purity based on Area Under Curve (AUC) at λ = 214 nm.*

6.2.3 Shuffling 3

H_2N- ITGIEFCADK IEALSR-$CONH_2$

The peptide shuffling 3 of barstar[75–90] was synthesized on commercially available Novabiochem® NovaPEG Rink Amide resin (0.41 mmol/g, 100.0 mg, 41 µmol) using the standard AFPS protocol. (**SI Figure 16**) Total synthesis time to afford resin-bound peptide was approximately 0.8 h. Cleavage of the peptidyl-resin (16.7 mg, approx. 6.9 µmol) afforded the crude peptide as a colorless solid (2.4 mg, 24% purity by LCMS [**SI Figure 17**], 18% purity by UHPLC [**SI Figure 18**])

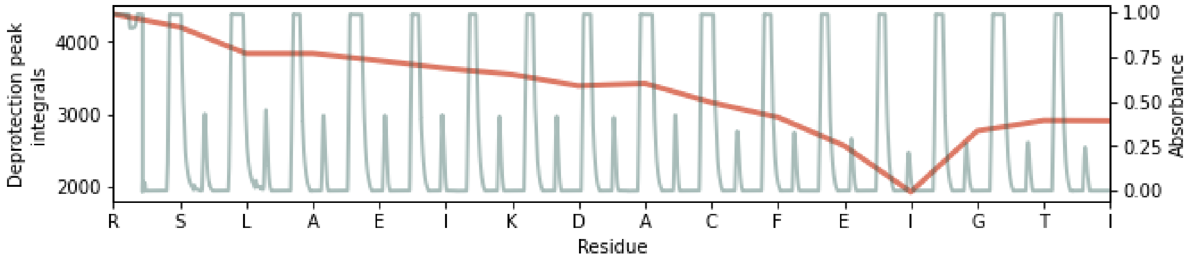

***SI Figure 16**. UV trace (λ = 310 nm) from AFPS of shuffling 3 of barstar [75–90] (green) and deprotection peak integrals (red).*

LC-MS of crude

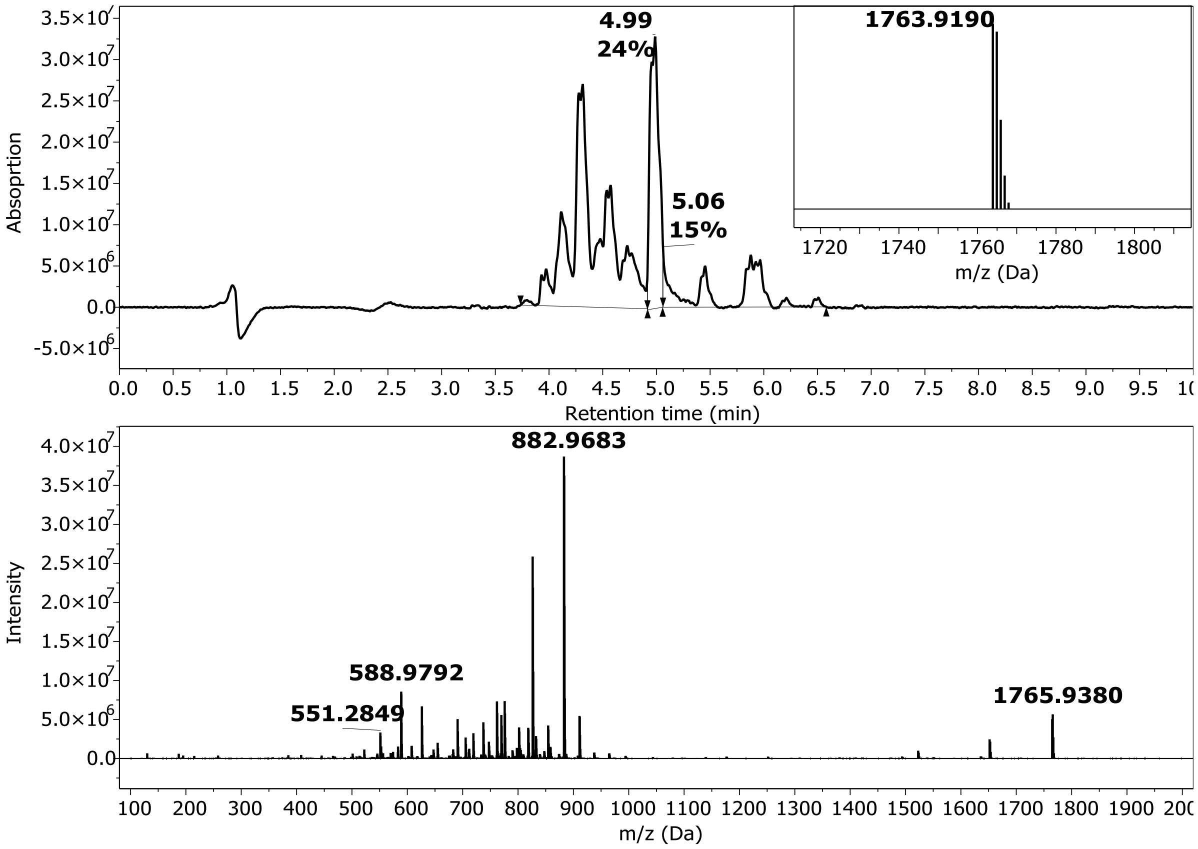

***SI Figure 17**. LCMS Profile of crude Top: Absorbance chromatogram of shuffling 3 of barstar[75–90] Rt 4.99 min, 24% purity Bottom: ESI-TOF spectrum found within Rt 2–8 min (insert: deconvoluted masses). Monoisotopic mass (ESI+) calcd. for $C_{77}H_{129}N_{21}O_{24}S$ 1763.9240, found 1763.9190.*

UHPLC

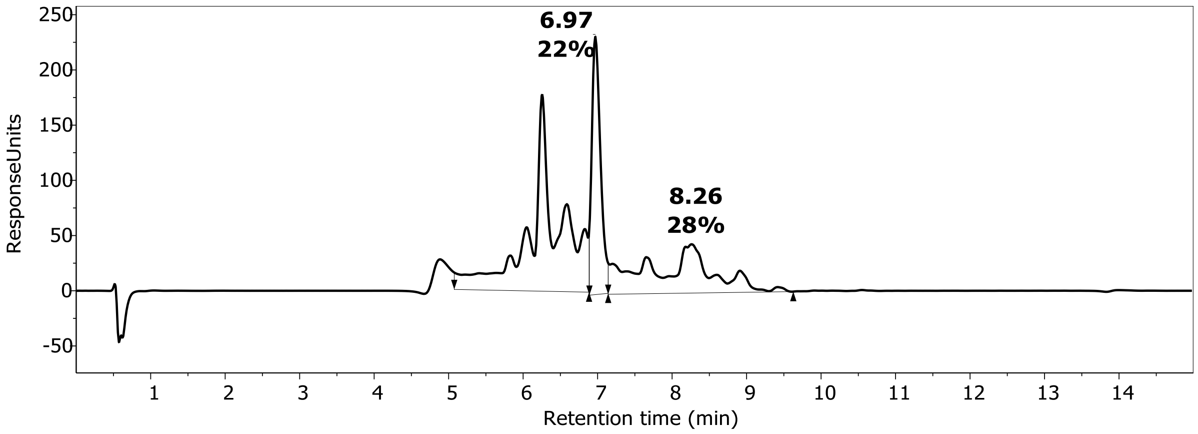

***SI Figure 18**. UHPLC profile of crude Rt 6.97 min, 22% purity based on Area Under Curve (AUC) at λ = 214 nm.*

6.2.4 Shuffling 4

H_2N- KTIIIAEFRS LAECDG-$CONH_2$

The peptide shuffling 4 of barstar[75–90] was synthesized on commercially available Novabiochem® NovaPEG Rink Amide resin (0.41 mmol/g, 101.4 mg, 42 μmol) using the standard AFPS protocol. (**SI Figure 19**) Total synthesis time to afford resin-bound peptide was approximately 0.8 h. Cleavage of the peptidyl-resin (16.8 mg, approx. 6.9 μmol) afforded the crude peptide as a colorless solid (4.6 mg, 21% purity by LCMS [**SI Figure 20**], 23% purity by UHPLC [**SI Figure 21**])

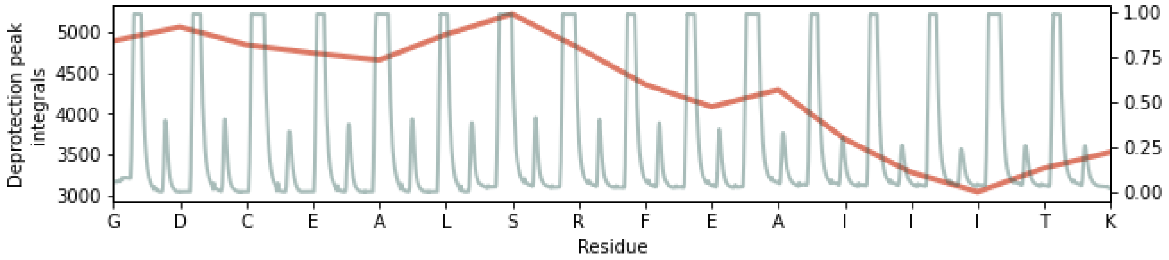

SI Figure 19. *UV trace (λ = 310 nm) from AFPS of shuffling 4 of barstar [75–90] (green) and deprotection peak integrals (red).*

LC-MS of crude

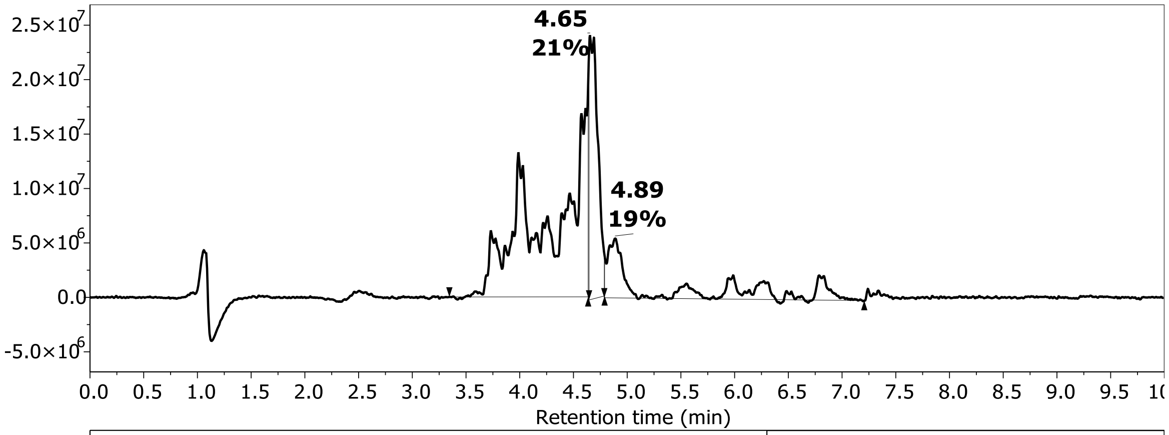

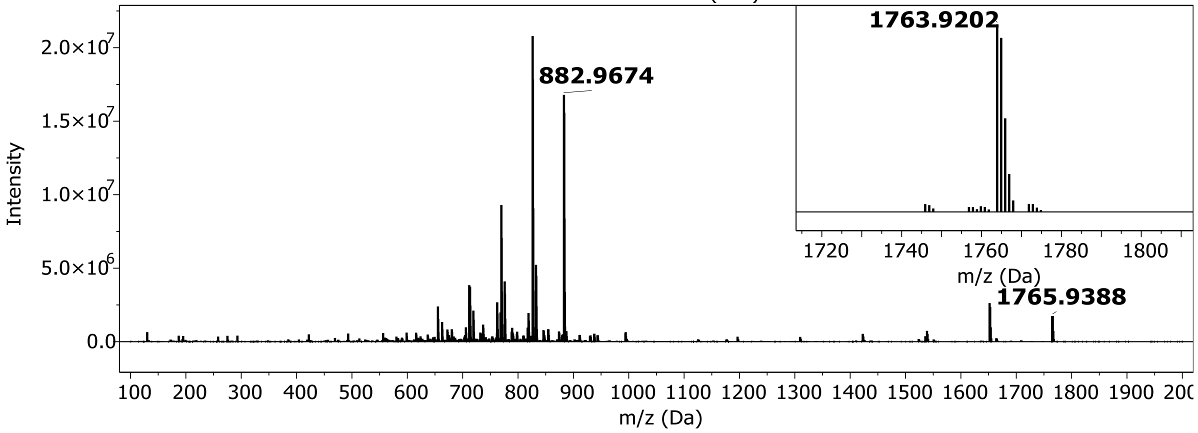

SI Figure 20. *LCMS Profile of crude Top: Absorbance chromatogram of shuffling 4 of barstar[75–90] Rt 4.65 min, 21% purity Bottom: ESI-TOF spectrum found within Rt 2–8 min (insert: deconvoluted masses). Monoisotopic mass (ESI+) calcd. for* $C_{77}H_{129}N_{21}O_{24}S$ *1763.9240, found 1763.9202.*

UHPLC

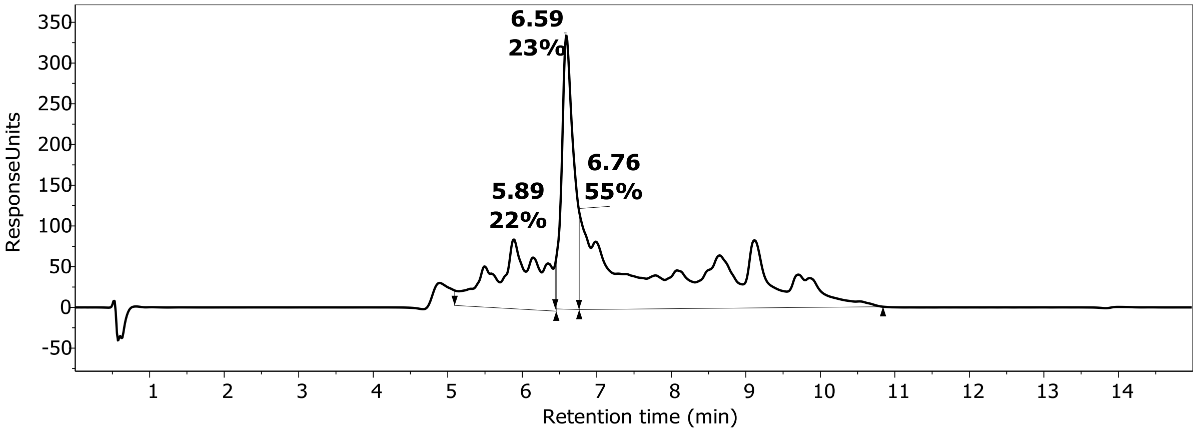

SI Figure 21. *UHPLC profile of crude Rt 6.59 min, 23% purity based on Area Under Curve (AUC) at λ = 214 nm.*

6.2.5 Shuffling 5

H_2N- IAIDLAFCIE SETRKG-$CONH_2$

The peptide shuffling 5 of barstar[75–90] was synthesized on commercially available Novabiochem® NovaPEG Rink Amide resin (0.41 mmol/g, 101.7 mg, 42 μmol) using the standard AFPS protocol. (**SI Figure 22**) Total synthesis time to afford resin-bound peptide was approximately 0.8 h. Cleavage of the peptidyl-resin (16.8 mg, approx. 6.9 μmol) afforded the crude peptide as a colorless solid (4.6 mg, 37% purity by LCMS [**SI Figure 23**], 31% purity by UHPLC [**SI Figure 24**]).

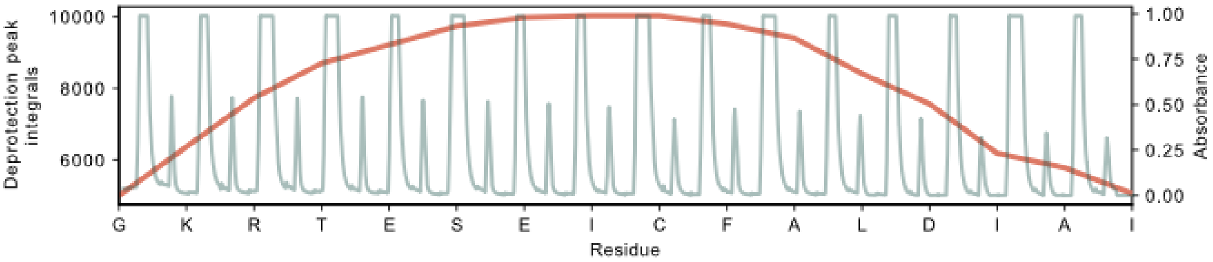

SI Figure 22*. UV trace (λ = 310 nm) from AFPS of shuffling 5 of barstar [75–90] (green) and deprotection peak integrals (red).*

LC-MS of crude

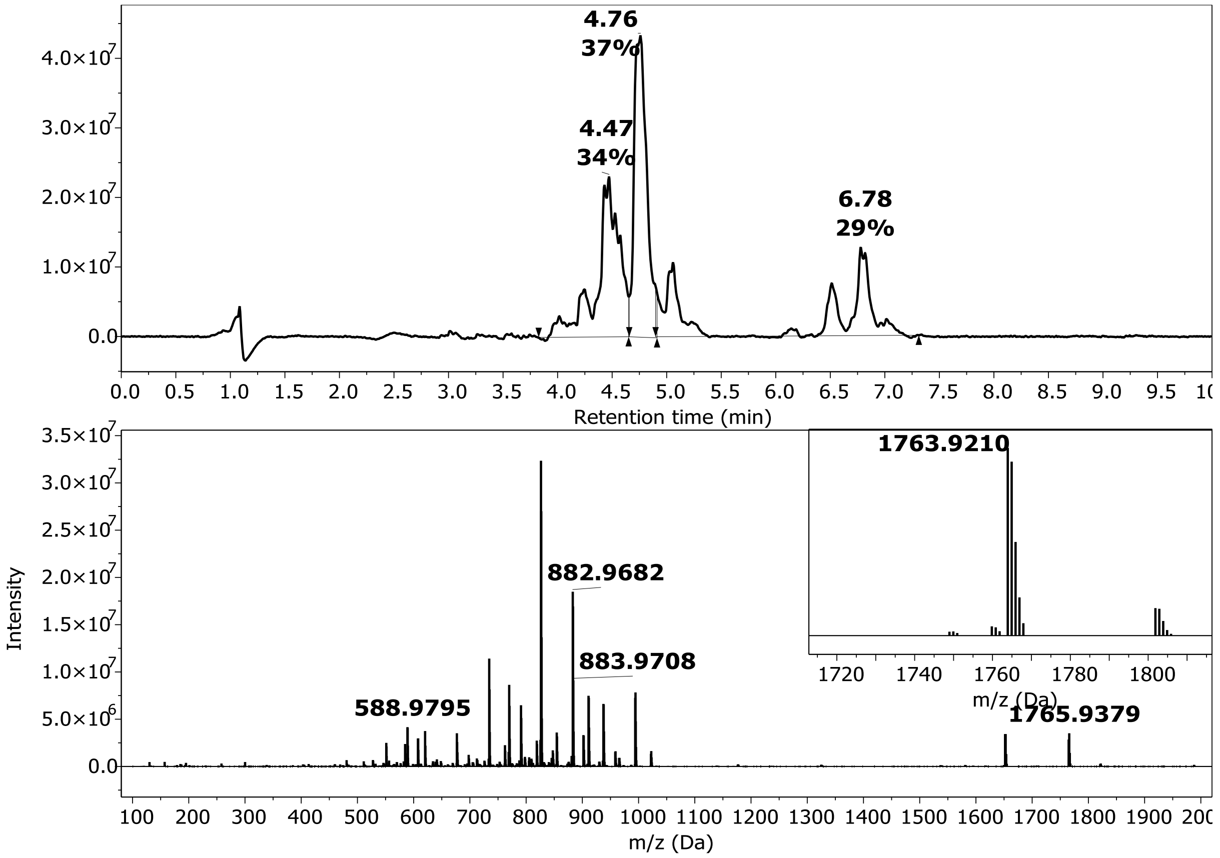

SI Figure 23. *LCMS Profile of crude Top: Absorbance chromatogram of shuffling 5 of barstar[75–90] Rt 4.76 min, 37% purity Bottom: ESI-TOF spectrum found within Rt 2–8 min (insert: deconvoluted masses). Monoisotopic mass (ESI+) calcd. for $C_{77}H_{129}N_{21}O_{24}S$ 1763.9240, found 1763.9210.*

UHPLC

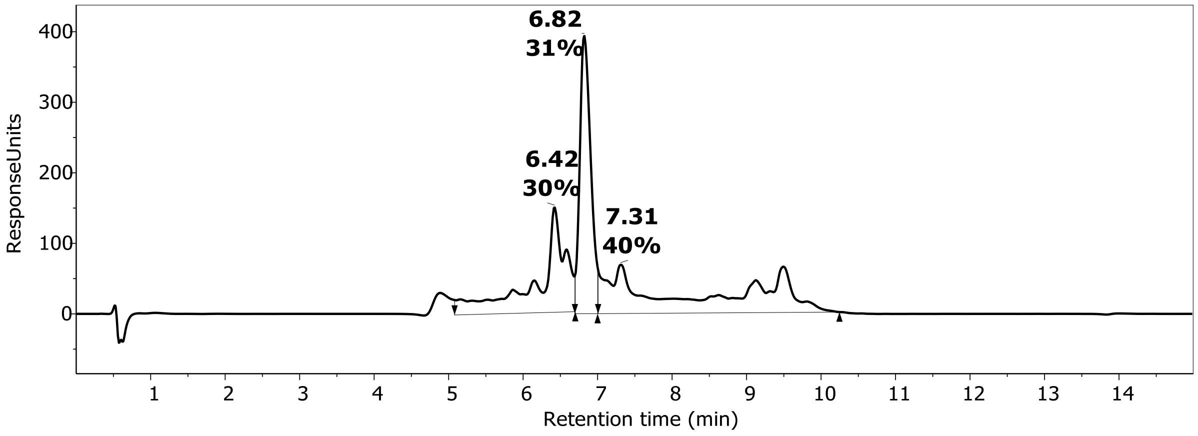

SI Figure 24. *UHPLC profile of crude Rt 6.82 min, 31% purity based on Area Under Curve (AUC) at λ = 214 nm.*

6.3 hGH[176–191]F176Y shuffling

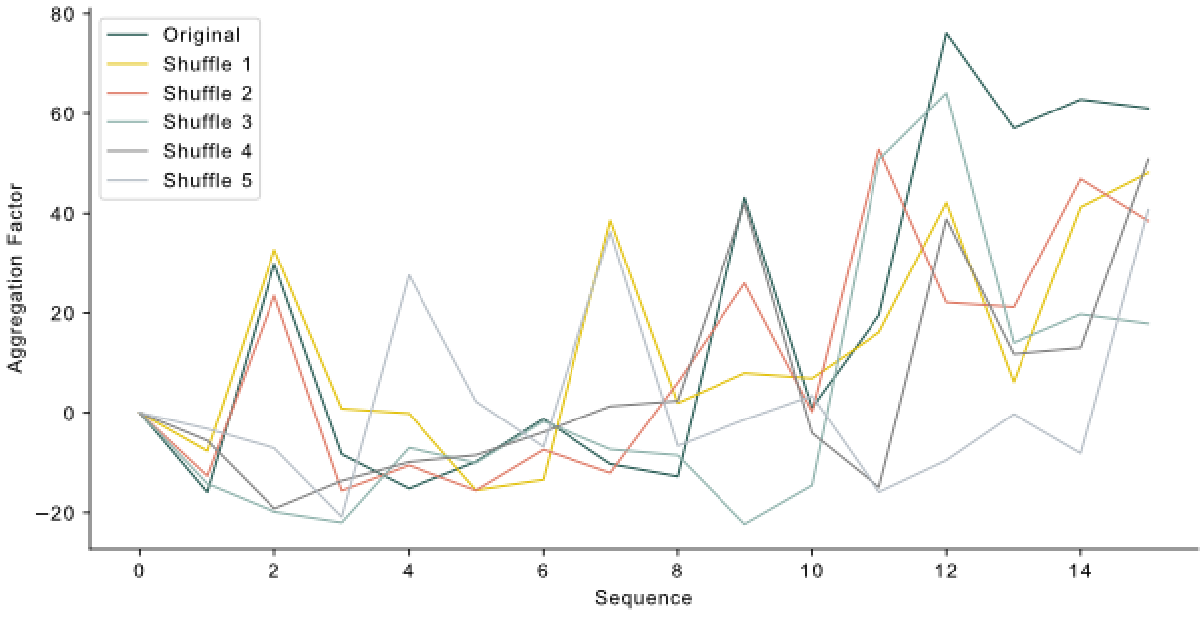

SI Figure 25*. Plot of aggregation factor values for the different shuffled hGH[176–191]F176Y fragments.*

6.3.1 Original sequence

H_2N- YLRIVQCRSV EGSCGF-$CONH_2$

The peptide hGH[176–191]F176Y was synthesized on commercially available Novabiochem® NovaPEG Rink Amide resin (0.41 mmol/g, 101.0 mg, 41 µmol) using the standard AFPS protocol. (**SI Figure 26**) Total synthesis time to afford resin-bound peptide was approximately 0.8 h. Cleavage of the peptidyl-resin (16.1 mg, approx. 6.6 µmol) afforded the crude peptide as a colorless solid (2.4 mg, 26% purity by LCMS [**SI Figure 27**], 23% purity by UHPLC [**SI Figure 28**])

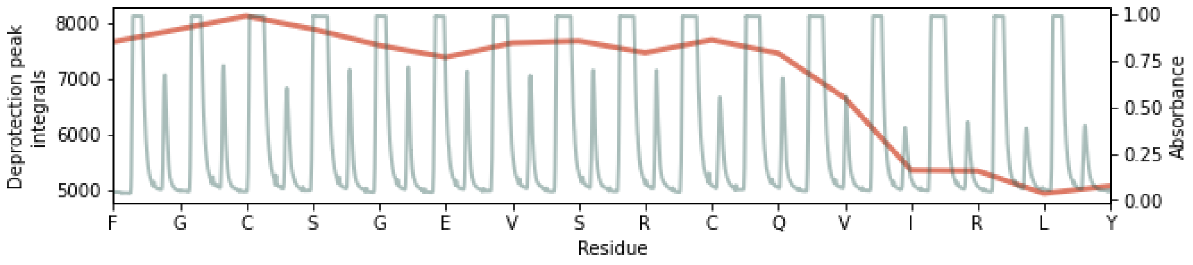

SI Figure 26*. UV trace (λ = 310 nm) from AFPS of hGH[176–191]F176Y (green) and deprotection peak integrals (red).*

LC-MS of crude

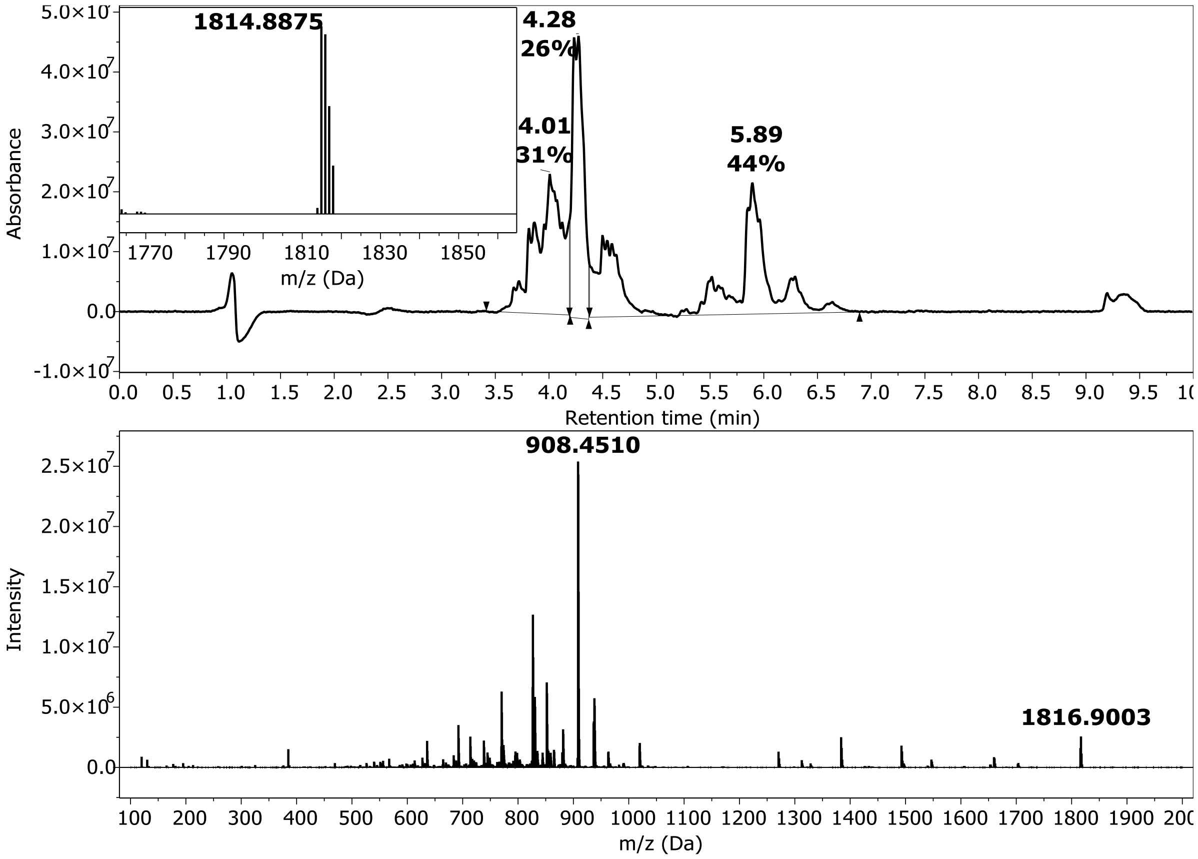

***SI Figure 27**. LCMS Profile of crude Top: Absorbance chromatogram of hGH[176–191]F176YRt 4.28 min, 26% purity Bottom: ESI-TOF spectrum found within Rt 2–8 min (insert: deconvoluted masses). Monoisotopic mass (ESI+) calcd. for $C_{78}H_{126}N_{24}O_{22}S_2$ 1814.8920, found 1814.8875.*

UHPLC

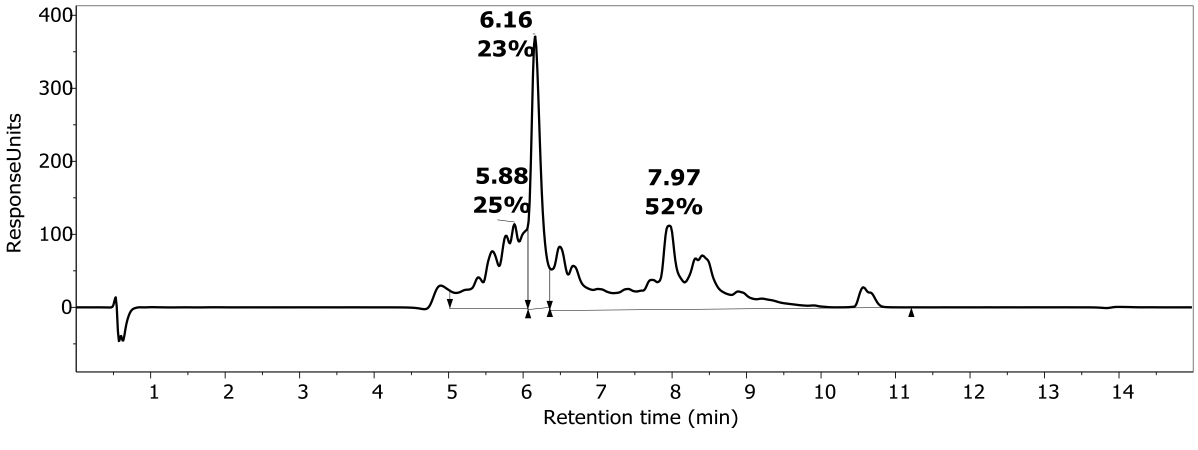

***SI Figure 28**. UHPLC profile of crude Rt 6.16 min, 23% purity based on Area Under Curve (AUC) at λ = 214 nm.*

6.3.2 Shuffling 1

H$_2$N- REYGCQISRG FLSCVV-CONH$_2$

The peptide shuffling 1 of hGH[176–191]F176Y was synthesized on commercially available Novabiochem® NovaPEG Rink Amide resin (0.41 mmol/g, 100.9 mg, 41 µmol) using the standard AFPS protocol. (**SI Figure 29**) Total synthesis time to afford resin-bound peptide was approximately 0.8

h. Cleavage of the peptidyl-resin (16.1 mg, approx. 6.6 µmol) afforded the crude peptide as a colorless solid (4.2 mg, 42% purity by LCMS [**SI Figure 30**], 38% purity by UHPLC [**SI Figure 31**]).

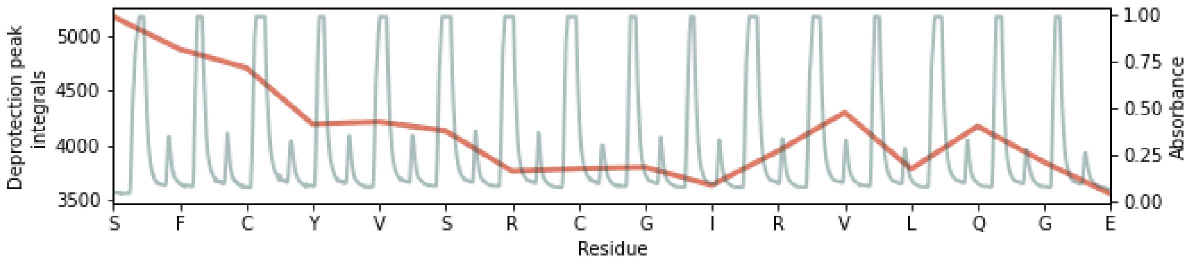

***SI Figure 29**. UV trace (λ = 310 nm) from AFPS of shuffling 1 of hGH[176–191]F176Y (green) and deprotection peak integrals (red).*

LC-MS of crude

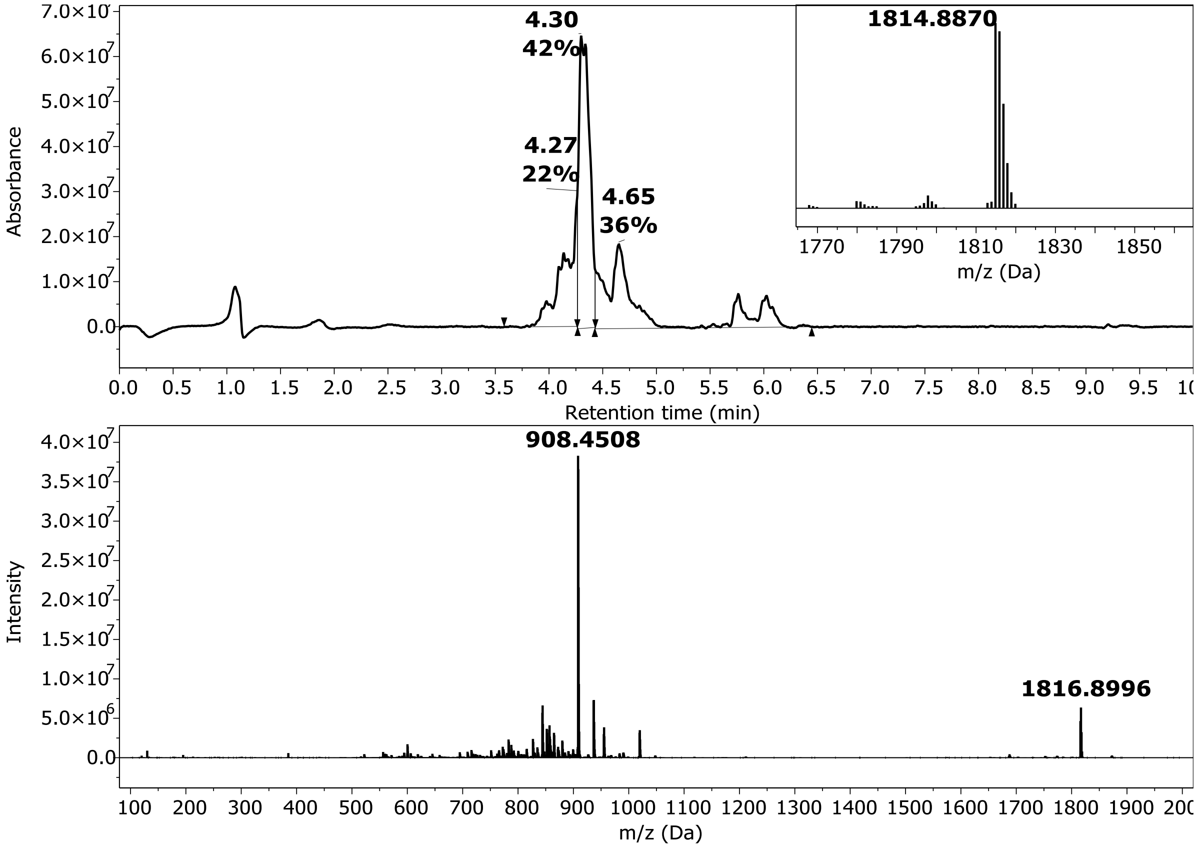

***SI Figure 30**. LCMS Profile of crude Top: Absorbance chromatogram of shuffling 1 of hGH[176–191]F176Y Rt 4.30 min, 42% purity Bottom: ESI-TOF spectrum found within Rt 2–8 min (insert: deconvoluted masses). Monoisotopic mass (ESI+) calcd. for $C_{78}H_{126}N_{24}O_{22}S_2$ 1814.8920, found 1814.8870.*

UHPLC

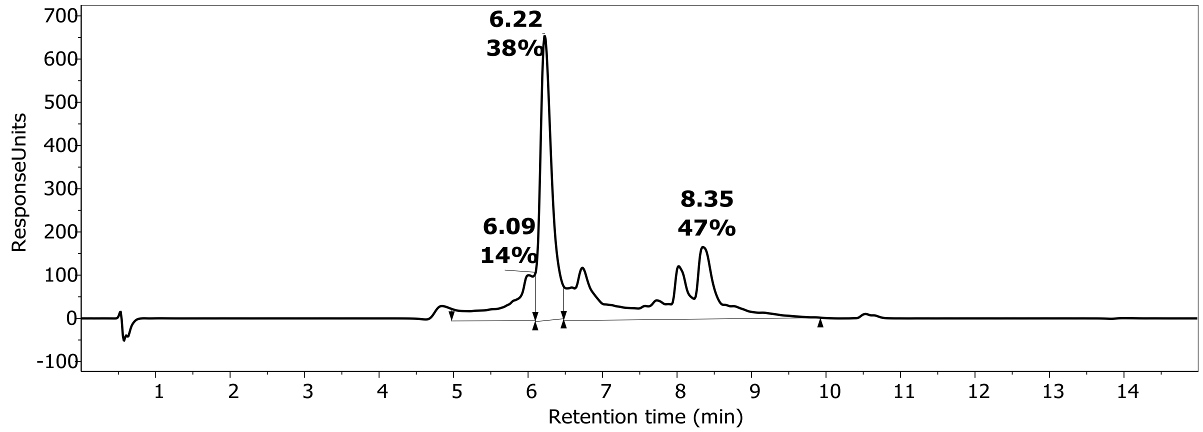

SI Figure 31. UHPLC profile of crude Rt 6.22 min, 38% purity based on Area Under Curve (AUC) at λ = 214 nm.

6.3.3 Shuffling 2

H_2N- REYGCQISRG FLSCVV-$CONH_2$

The peptide shuffling 2 of hGH[176–191]F176Y was synthesized on commercially available Novabiochem® NovaPEG Rink Amide resin (0.41 mmol/g, 101.7 mg, 42 µmol) using the standard AFPS protocol. (**SI Figure 32**) Total synthesis time to afford resin-bound peptide was approximately 0.8 h. Cleavage of the peptidyl-resin (16.9 mg, approx. 6.9 µmol) afforded the crude peptide as a colorless solid (3.2 mg, 26% purity by LCMS [**SI Figure 33**], 41% purity by UHPLC [**SI Figure 34**]).

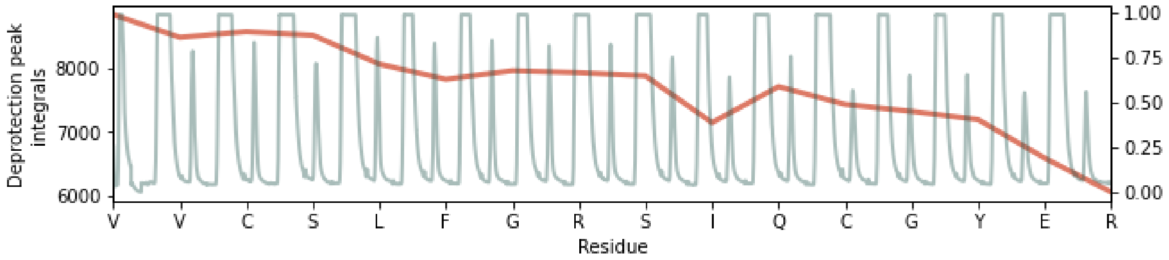

SI Figure 32. UV trace (λ = 310 nm) from AFPS of shuffling 2 of hGH[176–191]F176Y (green) and deprotection peak integrals (red).

LC-MS of crude

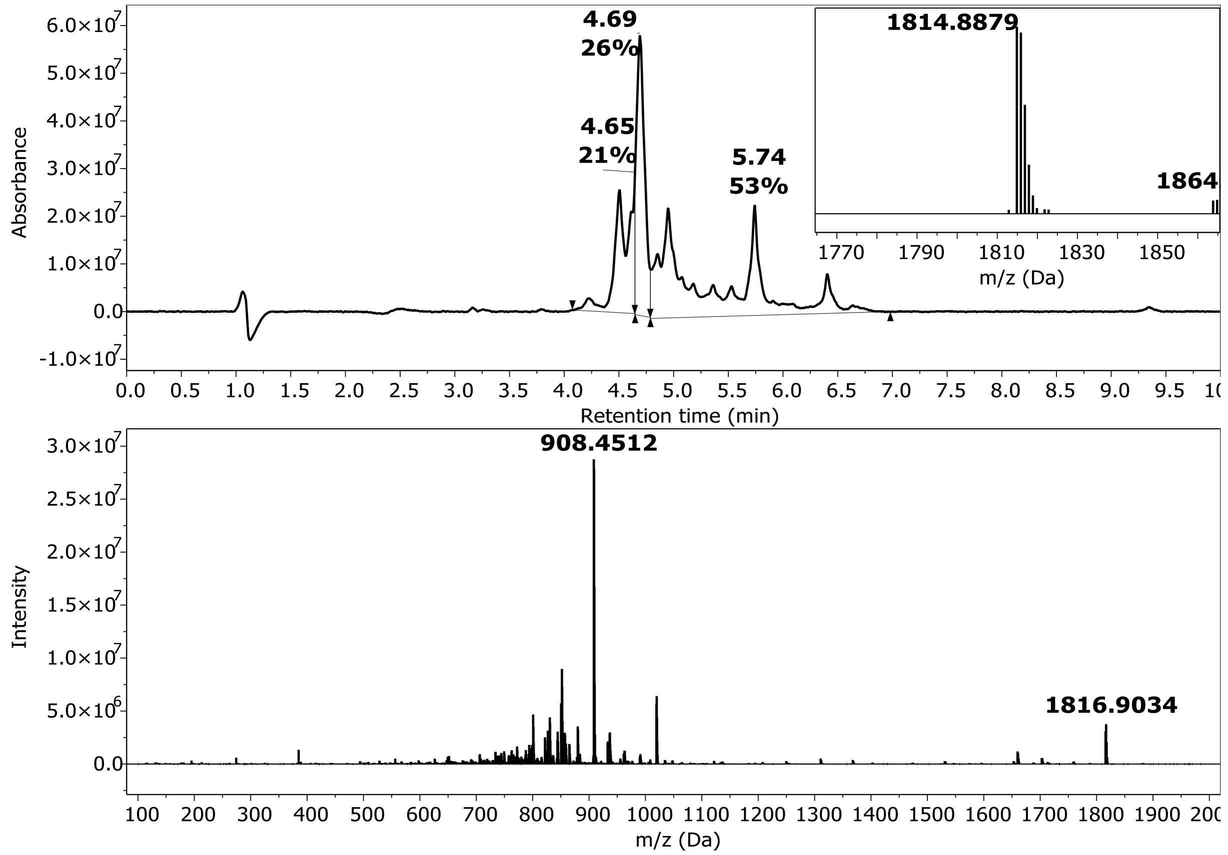

***SI Figure 33**. LCMS Profile of crude Top: Absorbance chromatogram of shuffling 2 of hGH[176–191]F176Y Rt 4.69 min, 26% purity Bottom: ESI-TOF spectrum found within Rt 2–8 min (insert: deconvoluted masses). Monoisotopic mass (ESI+) calcd. for $C_{78}H_{126}N_{24}O_{22}S_2$ 1814.8920, found 1814.8879.*

UHPLC

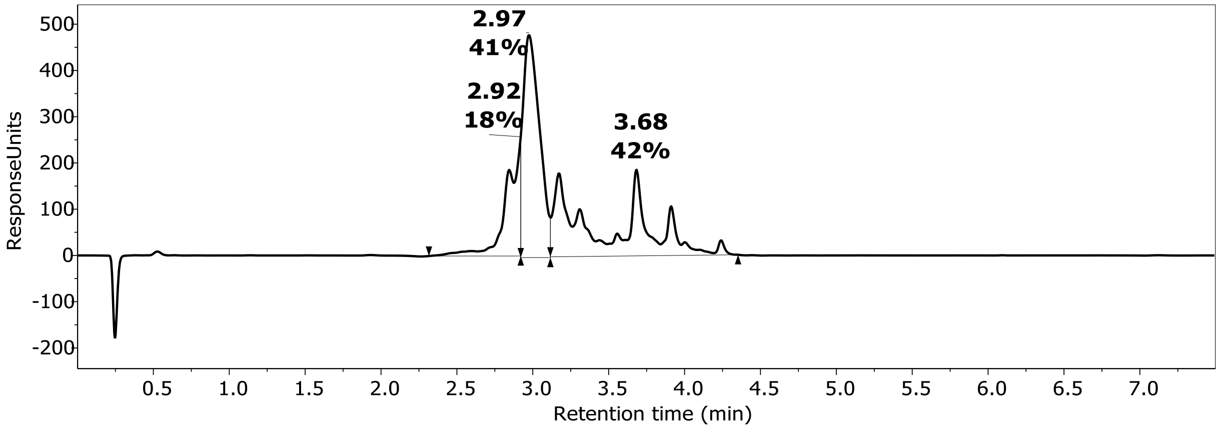

***SI Figure 34**. UHPLC profile of crude Rt 2.97 min, 41% purity based on Area Under Curve (AUC) at λ = 214 nm.*

6.3.4 Shuffling 3

H_2N- SGRCCYSIVV ERQGFL-$CONH_2$

The peptide shuffling 3 of hGH[176–191]F176Y was synthesized on commercially available Novabiochem® NovaPEG Rink Amide resin (0.41 mmol/g, 101.7 mg, 42 μmol) using the standard AFPS protocol. (**SI Figure 35**) Total synthesis time to afford resin-bound peptide was approximately 0.8

h. Cleavage of the peptidyl-resin (16.7 mg, approx. 6.9 µmol) afforded the crude peptide as a colorless solid (2.9 mg, 38% purity by LCMS [**SI Figure 36**], 44% purity by UHPLC [**SI Figure 37**])

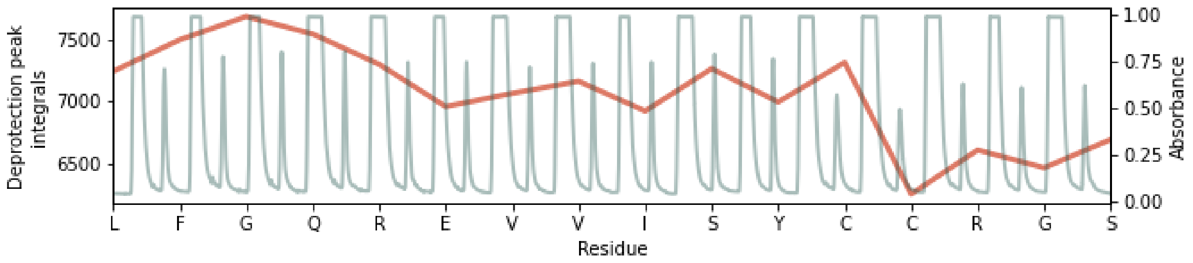

***SI Figure 35**. UV trace (λ = 310 nm) from AFPS of shuffling 3 of hGH[176–191]F176Y (green) and deprotection peak integrals (red).*

LC-MS of crude

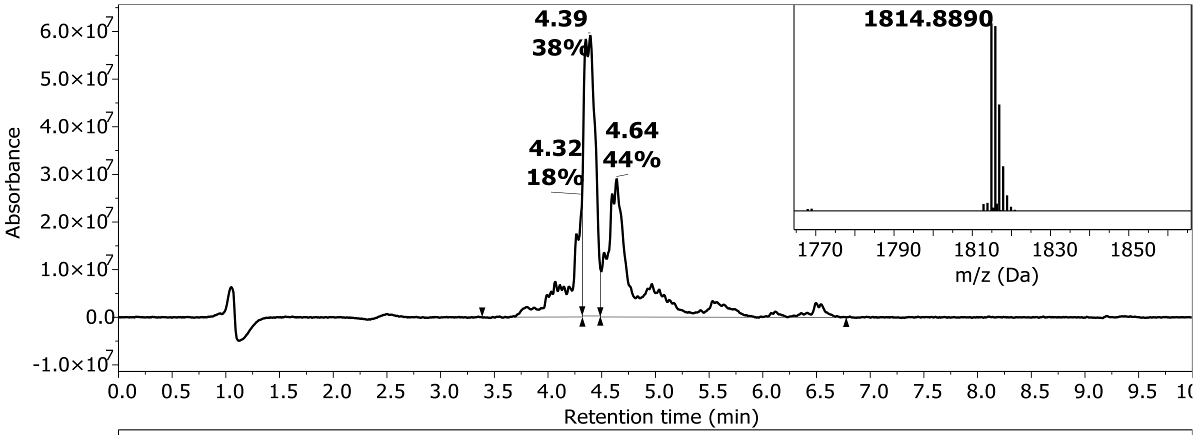

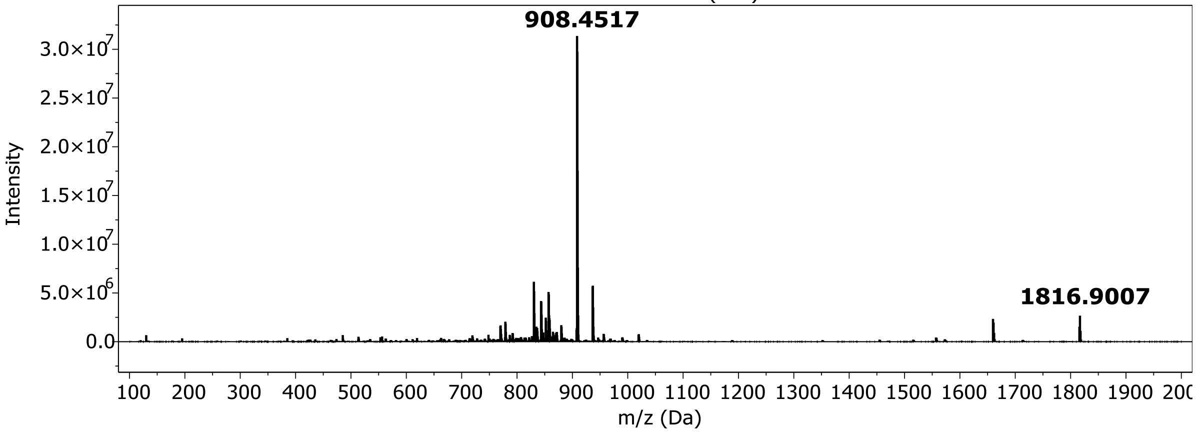

***SI Figure 36**. LCMS Profile of crude Top: Absorbance chromatogram of shuffling 3 of hGH[176–191]F176Y Rt 4.39 min, 38% purity Bottom: ESI-TOF spectrum found within Rt 2–8 min (insert: deconvoluted masses). Monoisotopic mass (ESI+) calcd. for $C_{78}H_{126}N_{24}O_{22}S_2$ 1814.8920, found 1814.8890.*

UHPLC

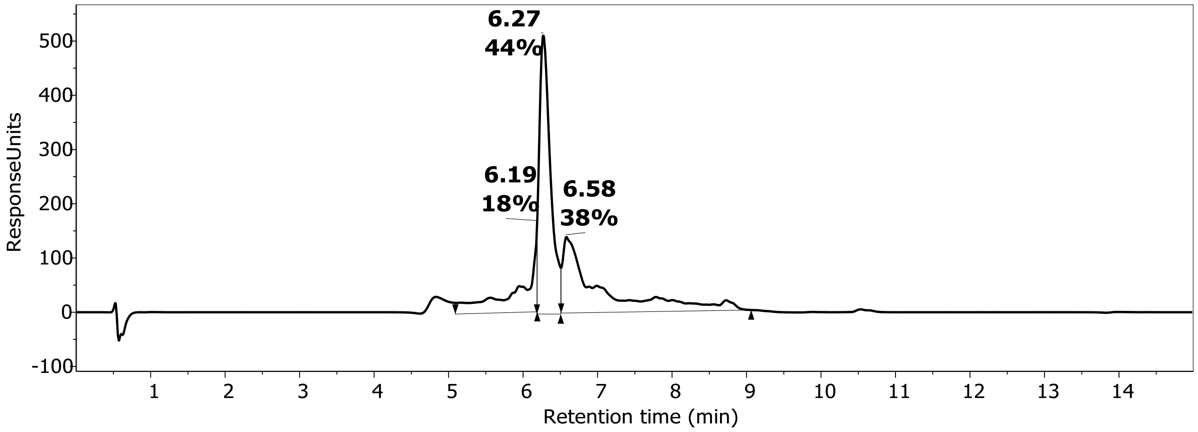

SI Figure 37. UHPLC profile of crude Rt 6.27 min, 44% purity based on Area Under Curve (AUC) at λ = 214 nm.

6.3.5 Shuffling 4

H_2N- VGECSICYLF GQRSVR-$CONH_2$

The peptide shuffling 4 of hGH[176–191]F176Y was synthesized on commercially available Novabiochem® NovaPEG Rink Amide resin (0.41 mmol/g, 101.7 mg, 42 µmol) using the standard AFPS protocol. (**SI Figure 38**) Total synthesis time to afford resin-bound peptide was approximately 0.8 h. Cleavage of the peptidyl-resin (16.6 mg, approx. 6.9 µmol) afforded the crude peptide as a colorless solid (0.8 mg, 38% purity by LCMS [**SI Figure 39**], 38% purity by UHPLC [**SI Figure 40**])

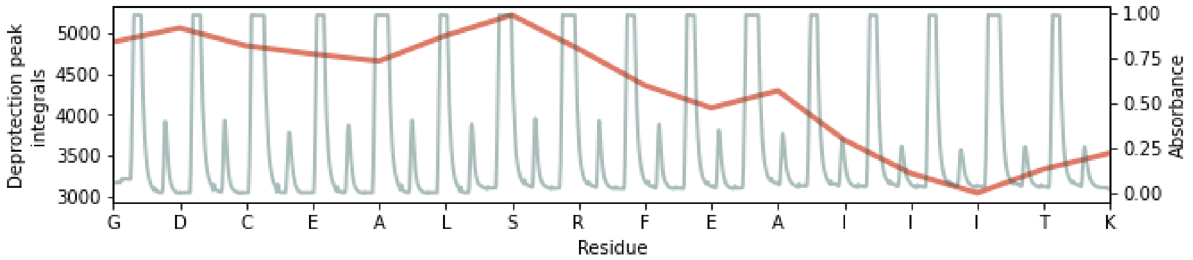

SI Figure 38. UV trace (λ = 310 nm) from AFPS of shuffling 4 of hGH[176–191]F176Y (green) and deprotection peak integrals (red).

LC-MS of crude

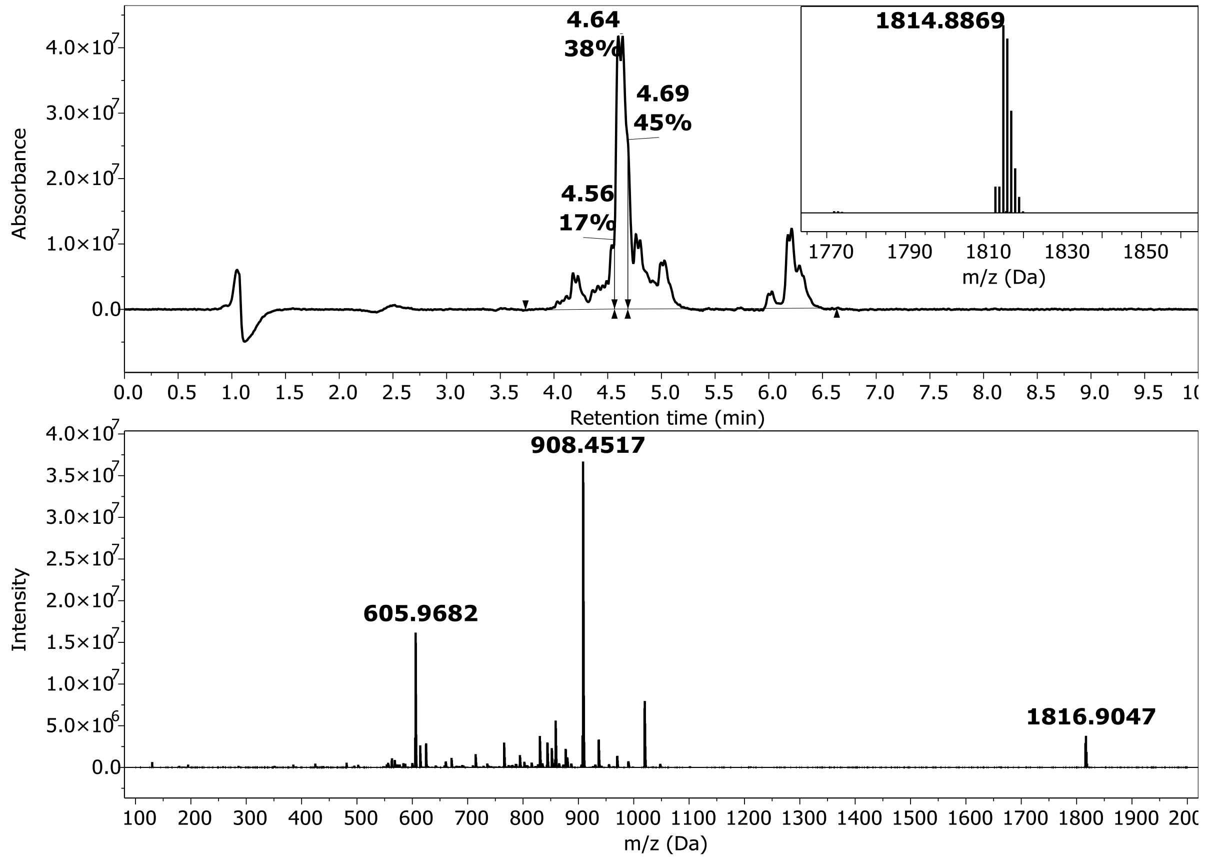

SI Figure 39. LCMS Profile of crude Top: Absorbance chromatogram of shuffling 4 of hGH[176–191]F176Y Rt 4.64 min, 38% purity Bottom: ESI-TOF spectrum found within Rt 2–8 min (insert: deconvoluted masses). Monoisotopic mass (ESI+) calcd. for $C_{78}H_{126}N_{24}O_{22}S_{2}$ 1814.8920, found 1814.8869.

UHPLC

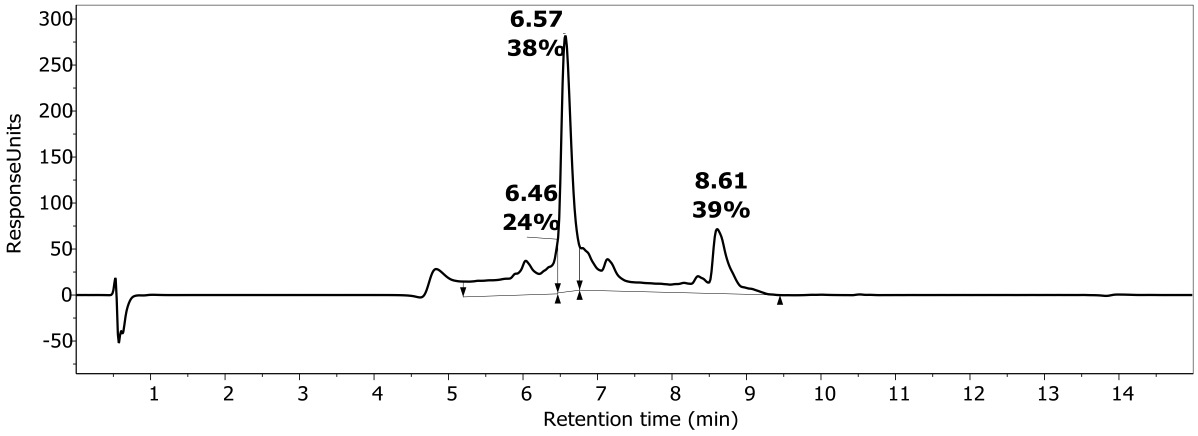

SI Figure 40. UHPLC profile of crude Rt 6.57 min, 38% purity based on Area Under Curve (AUC) at λ = 214 nm.

6.3.6 Shuffling 5

H_2N- EQSVGIYSCR FCGLVR-$CONH_2$

The peptide shuffling 5 of hGH[176–191]F176Y was synthesized on commercially available Novabiochem® NovaPEG Rink Amide resin (0.41 mmol/g, 101.0 mg, 41 µmol) using the standard AFPS protocol. (**SI Figure 41**) Total synthesis time to afford resin-bound peptide was approximately 0.8

h. Cleavage of the peptidyl-resin (16.4 mg, approx. 6.7 µmol) afforded the crude peptide as a colorless solid (1.4 mg, 51% purity by LCMS [**SI Figure 42**], 64% purity by UHPLC [**SI Figure 43**]).

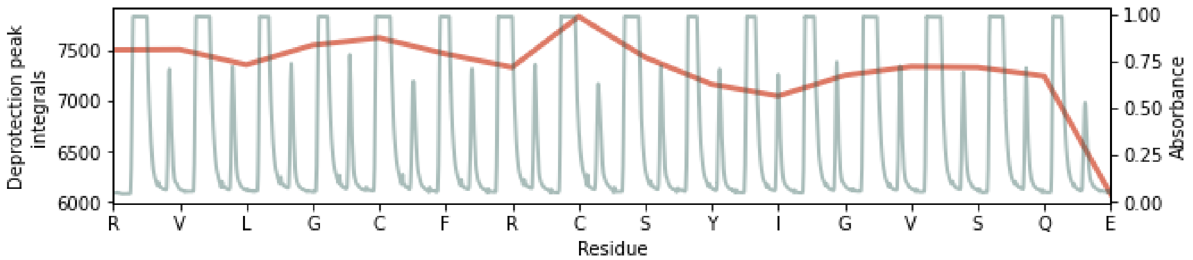

***SI Figure 41**. UV trace (λ = 310 nm) from AFPS of shuffling 5 of hGH[176–191]F176Y (green) and deprotection peak integrals (red).*

LC-MS of crude

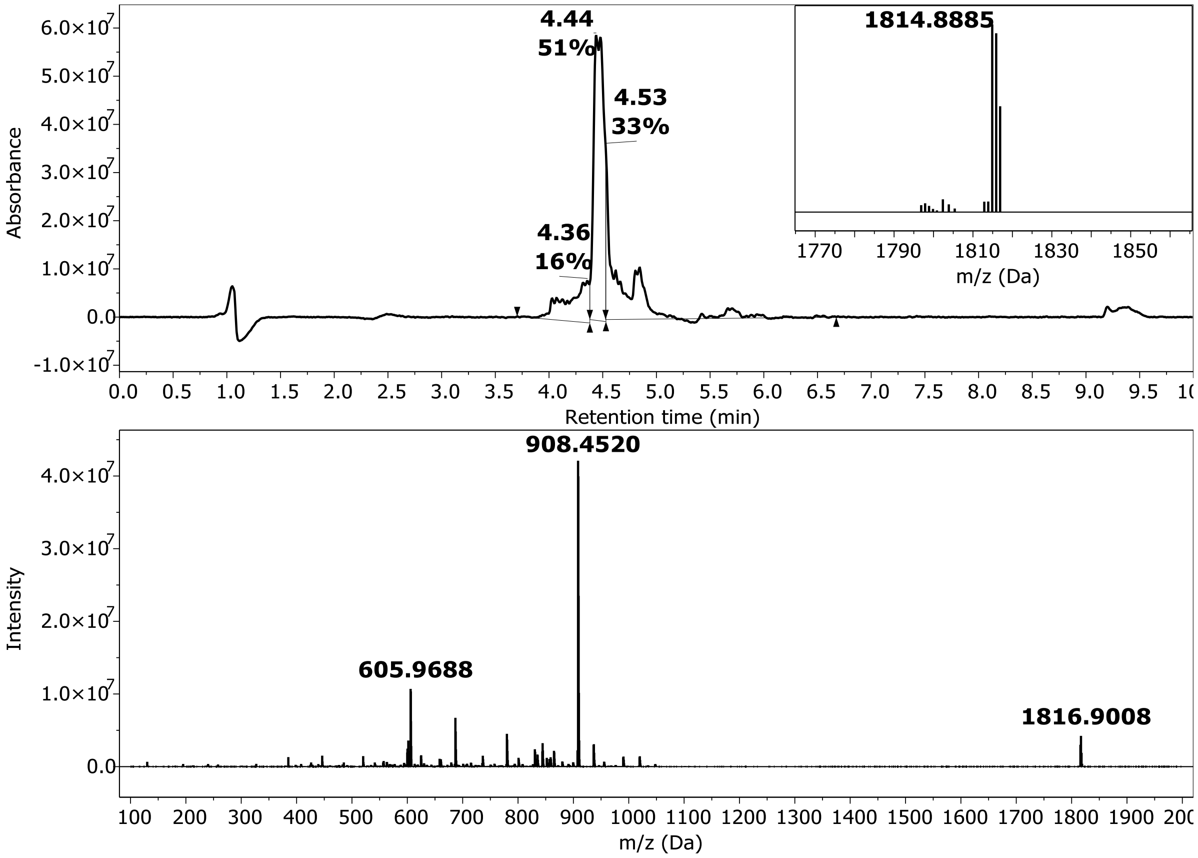

***SI Figure 42**. LCMS Profile of crude Top: Absorbance chromatogram of shuffling 5 of hGH[176–191]F176Y Rt 4.44 min, 51% purity Bottom: ESI-TOF spectrum found within Rt 2–8 min (insert: deconvoluted masses). Monoisotopic mass (ESI+) calcd. for $C_{78}H_{126}N_{24}O_{22}S_2$ 1814.8920, found 1814.8885.*

UHPLC

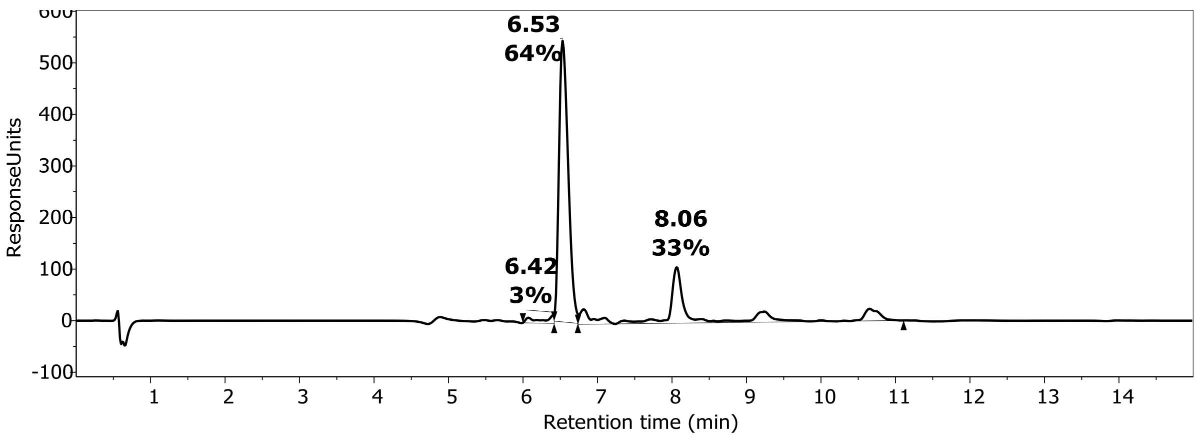

SI Figure 43. UHPLC profile of crude Rt 6.53 min, 64% purity based on Area Under Curve (AUC) at λ = 214 nm.

6.4 GLP-1 shuffling

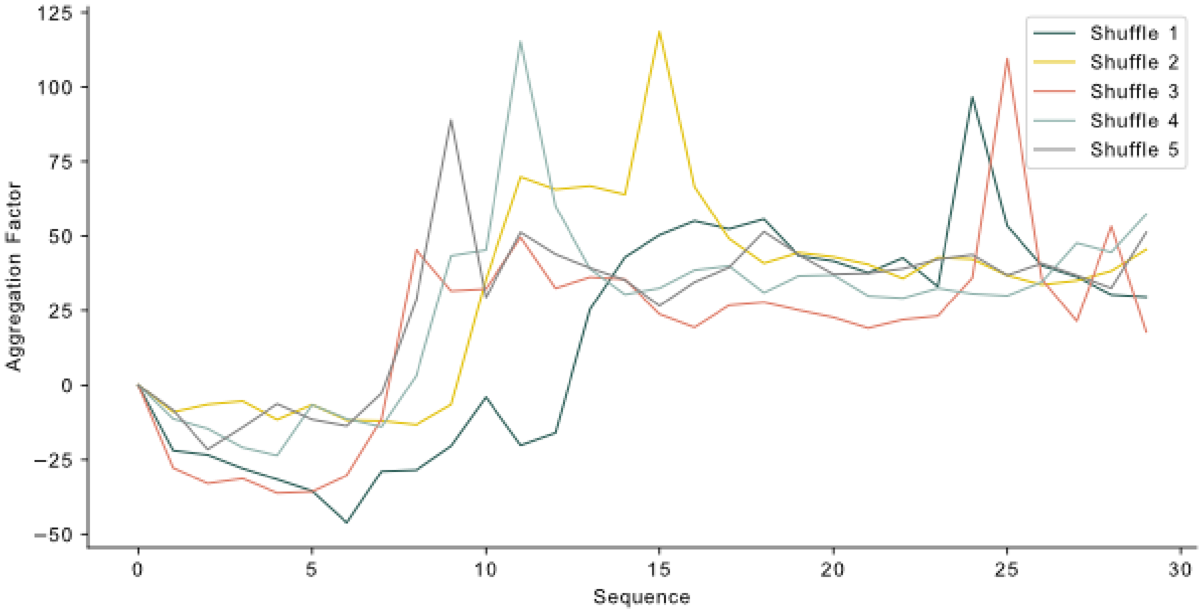

SI Figure 44. Plot of aggregation factor values for the different shuffled GLP-1 fragments.

6.4.1 Shuffling 1

H_2N- GSAAFHSVRE ELFEKTATSI VGGQDALWYK-$CONH_2$

The peptide shuffling 1 of GLP-1 was synthesized on commercially available Novabiochem® NovaPEG Rink Amide resin (0.41 mmol/g, 100.1 mg, 41 µmol) using the standard AFPS protocol(**SI Figure 45**) Total synthesis time to afford resin-bound peptide was approximately 1.5 h. Cleavage of the peptidyl-resin (16.9 mg, approx. 6.9 µmol) afforded the crude peptide as a colorless solid (5.5 mg, 21% purity by LCMS [**SI Figure 46**], 28% purity by UHPLC [**SI Figure 47**]).

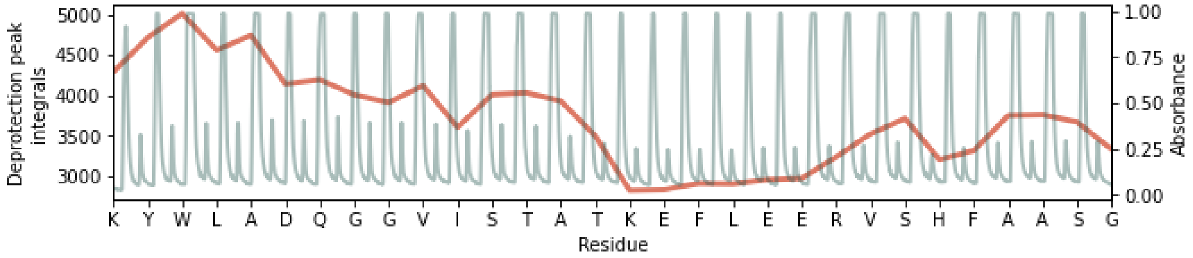

SI Figure 45. *UV trace (λ = 310 nm) from AFPS of shuffling 1 of GLP-1 (green) and deprotection peak integrals (red).*

LC-MS of crude

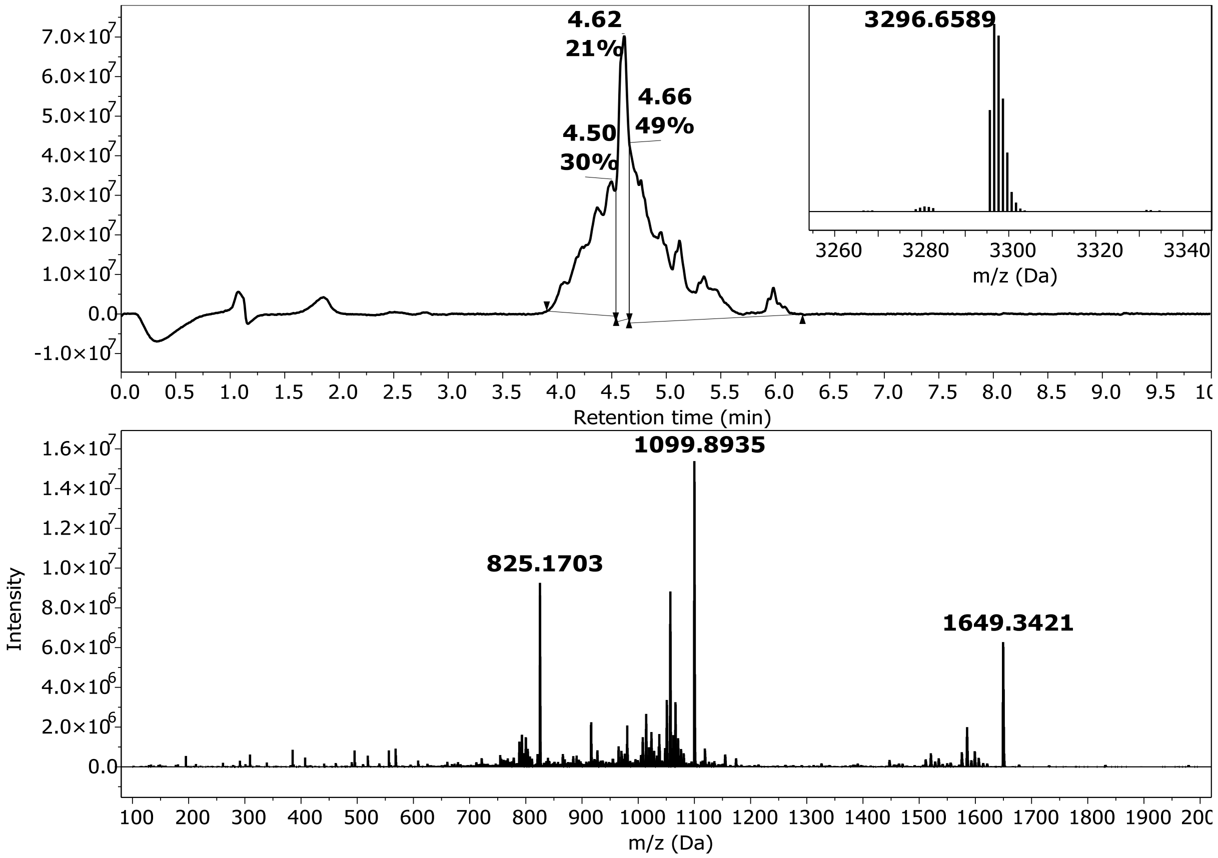

SI Figure 46. *LCMS Profile of crude Top: Absorbance chromatogram of shuffling 1 of GLP-1 Rt 4.62 min, 21% purity Bottom: ESI-TOF spectrum found within Rt 2–8 min (insert: deconvoluted masses). Monoisotopic mass (ESI+) calcd. for* $C_{149}H_{226}N_{40}O_{45}$ *3295.6626, found 3295.6551.*

UHPLC

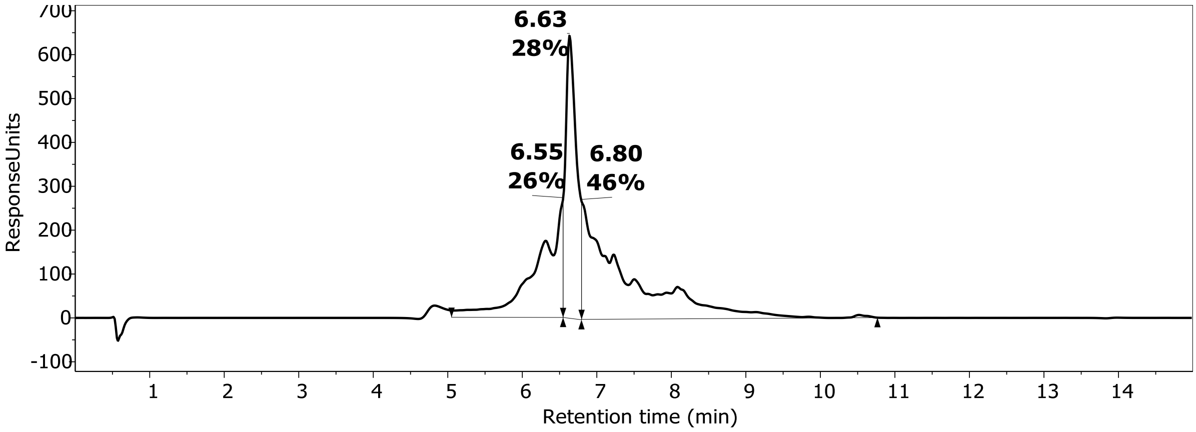

SI Figure 47. UHPLC profile of crude Rt 6.63 min, 28% purity based on Area Under Curve (AUC) at λ = 214 nm.

6.4.2 Shuffling 2

H_2N- VQSSAAFRSE VGALHKITYF EGGDEWLKAT-$CONH_2$

The peptide shuffling 2 of GLP-1 was synthesized on commercially available Novabiochem® NovaPEG Rink Amide resin (0.41 mmol/g, 100.8 mg, 41 µmol) using the standard AFPS protocol. (**SI Figure 48**) Total synthesis time to afford resin-bound peptide was approximately 1.5 h. Cleavage of the peptidyl-resin (16.8 mg, approx. 7.0 µmol) afforded the crude peptide as a colorless solid (6.9 mg, 20% purity by LCMS [**SI Figure 49**], 30% purity by UHPLC [**SI Figure 50**]).

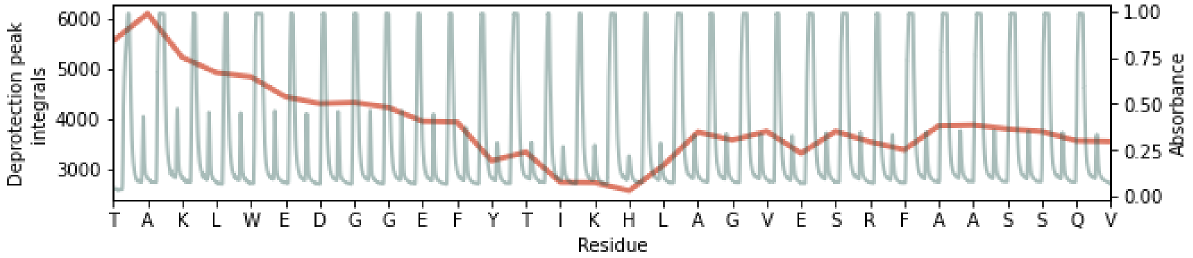

SI Figure 48. UV trace (λ = 310 nm) from AFPS of shuffling 2 of GLP-1 (green) and deprotection peak integrals (red).

LC-MS of crude

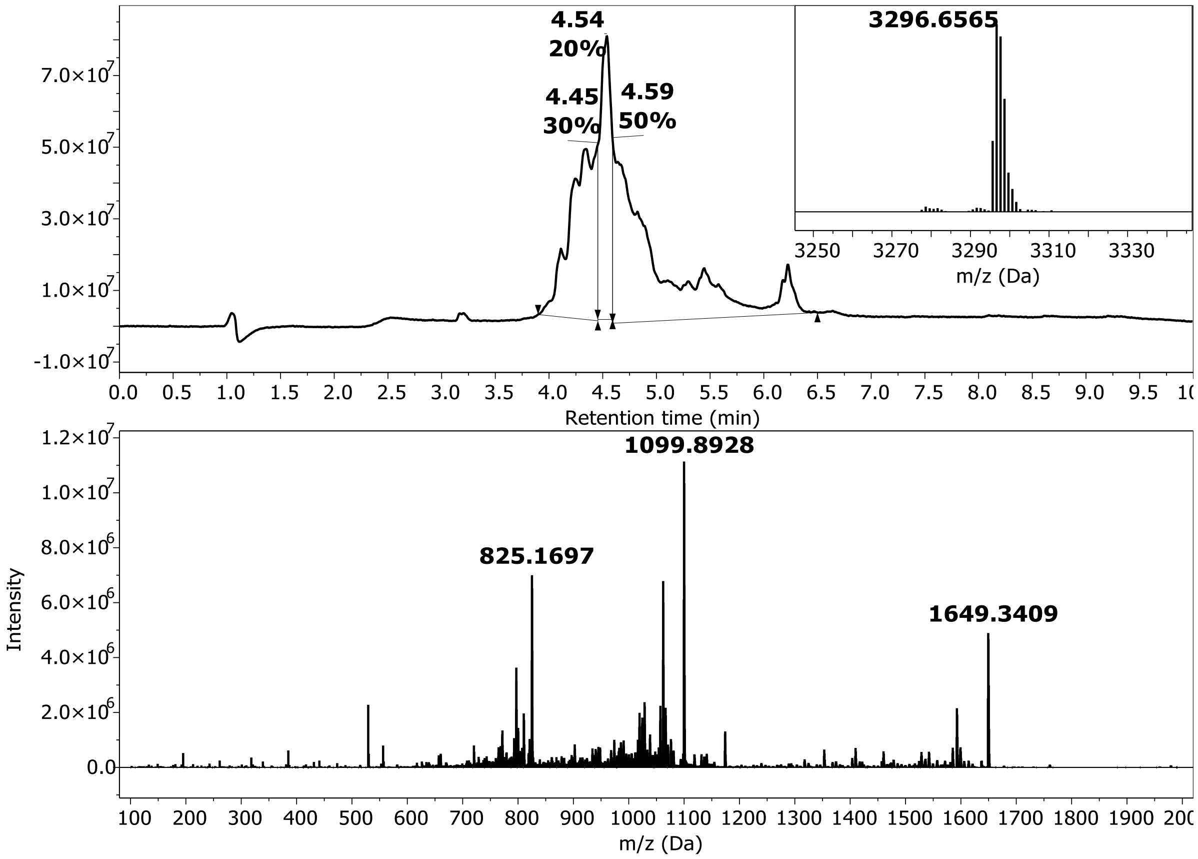

***SI Figure 49**. LCMS Profile of crude Top: Absorbance chromatogram of shuffling 2 of GLP-1 Rt 4.54 min, 20% purity Bottom: ESI-TOF spectrum found within Rt 2–8 min (insert: deconvoluted masses). Monoisotopic mass (ESI+) calcd. for for $C_{149}H_{226}N_{40}O_{45}$ 3295.6626, found 3295.6554.*

UHPLC

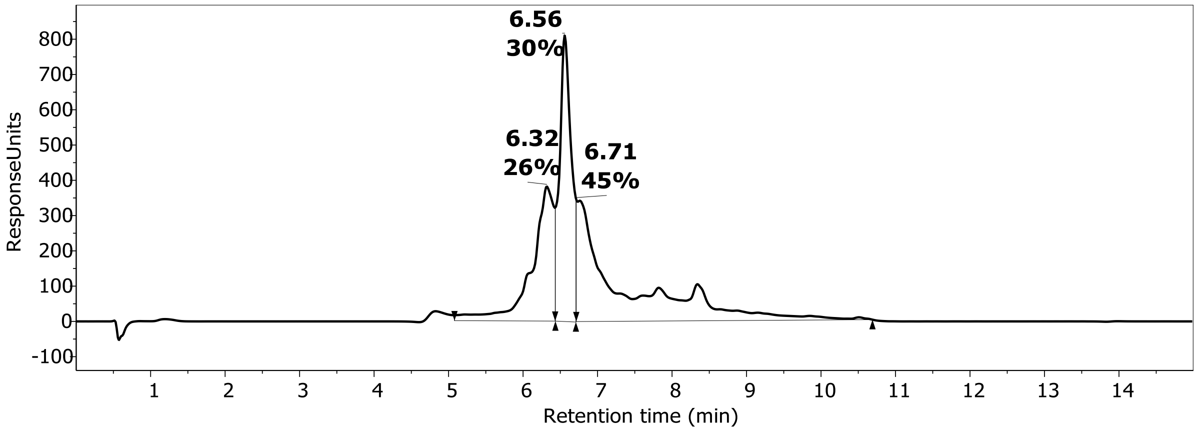

***SI Figure 50.** UHPLC profile of crude Rt 6.56 min, 30% purity based on Area Under Curve (AUC) at λ = 214 nm.*

6.4.3 Shuffling 3

H_2N- GIAGHLTSEQ EWYASLAGTK DVFFSVEARK-$CONH_2$

The peptide shuffling 3 of GLP-1 was synthesized on commercially available Novabiochem® NovaPEG Rink Amide resin (0.41 mmol/g, 101.9 mg, 42 µmol) using the standard AFPS protocol. (**SI Figure 51**) Total synthesis time to afford resin-bound peptide was approximately 1.5 h. Cleavage of the

peptidyl-resin (16.2 mg, approx. 6.6 µmol) afforded the crude peptide as a colorless solid (6.6 mg, 20% purity by LCMS [**SI Figure 52**], 36% purity by UHPLC [**SI Figure 53**]).

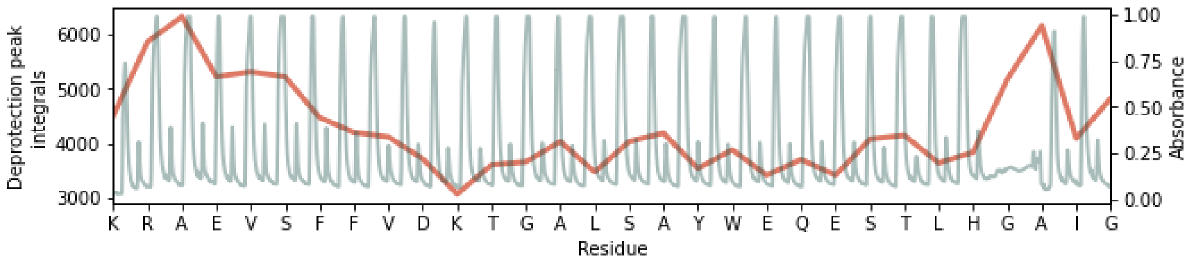

SI Figure 51. *UV trace (λ = 310 nm) from AFPS of shuffling 3 of GLP-1 (green) and deprotection peak integrals (red).*

LC-MS of crude

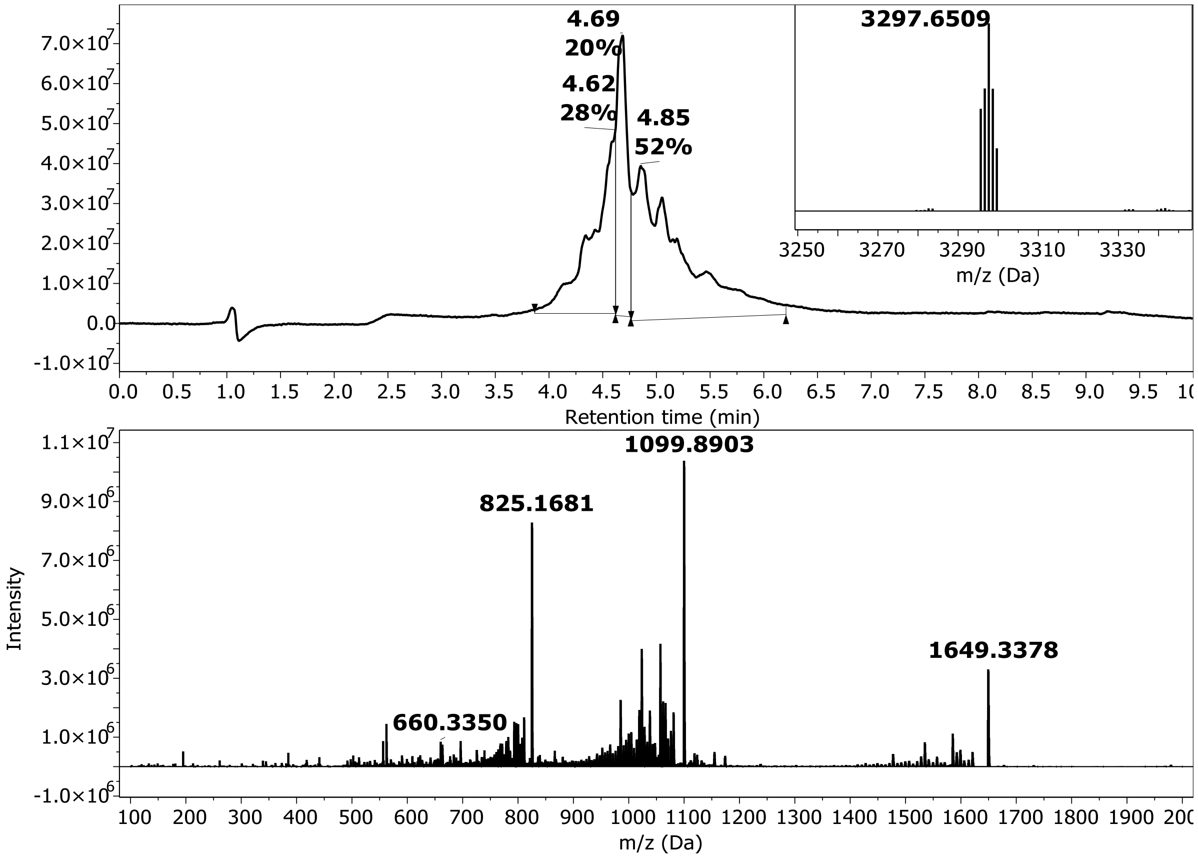

SI Figure 52. *LCMS Profile of crude Top: Absorbance chromatogram of shuffling 3 of GLP-1 Rt 4.69 min, 20% purity Bottom: ESI-TOF spectrum found within Rt 2–8 min (insert: deconvoluted masses). Monoisotopic mass (ESI+) calcd. for for $C_{149}H_{226}N_{40}O_{45}$ 3295.6626, found 3295.6471.*

UHPLC

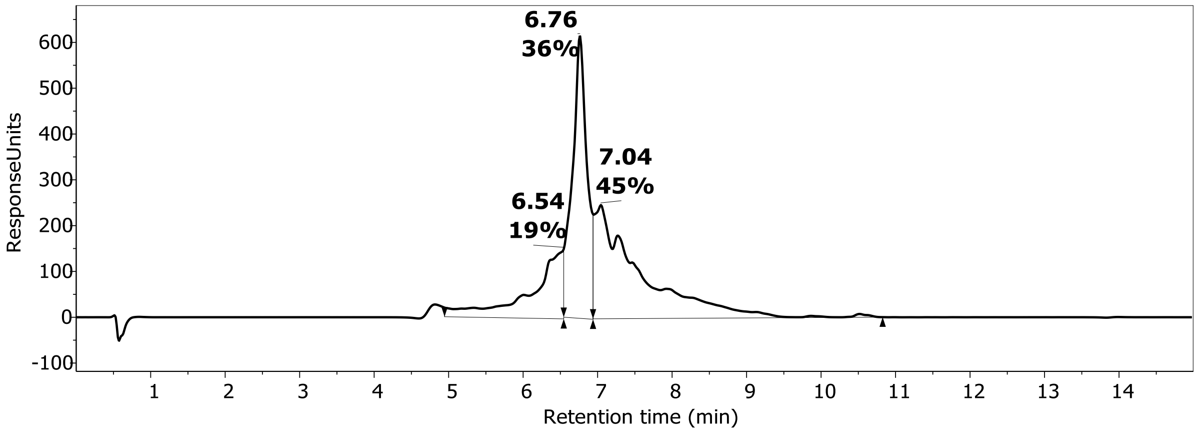

SI Figure 53. UHPLC profile of crude Rt 6.76 min, 36% purity based on Area Under Curve (AUC) at λ = 214 nm.

6.4.4 Shuffling 4

H_2N- VFVESFAITY LQKLGRDAHW EGEGKSTAAS-$CONH_2$

The peptide shuffling 4 of GLP-1 was synthesized on commercially available Novabiochem® NovaPEG Rink Amide resin (0.41 mmol/g, 101.0 mg, 42 µmol) using the standard AFPS protocol. (**SI Figure 54**) Total synthesis time to afford resin-bound peptide was approximately 1.5 h. Cleavage of the peptidyl-resin (16.8 mg, approx. 6.9 µmol) afforded the crude peptide as a colorless solid (7.1 mg, 19% purity by LCMS [**SI Figure 55**], 29% purity by UHPLC [**SI Figure 56**]).

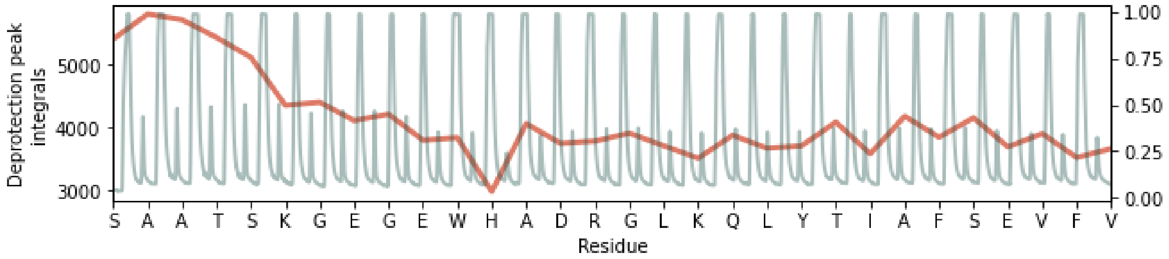

SI Figure 54. UV trace (λ = 310 nm) from AFPS of shuffling 4 of GLP-1 (green) and deprotection peak integrals (red).

LC-MS of crude

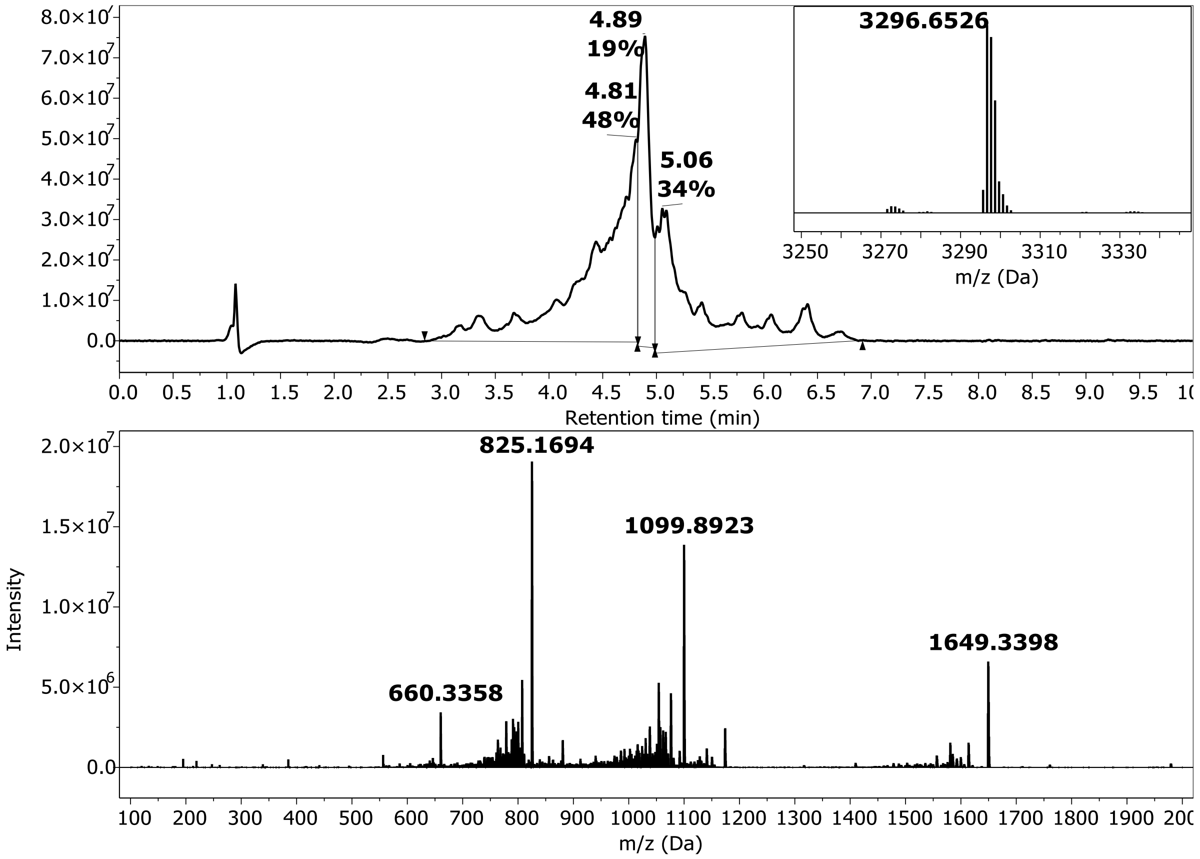

***SI Figure 55**. LCMS Profile of crude Top: Absorbance chromatogram of shuffling 4 of GLP-1 Rt 4.89 min, 19% purity Bottom: ESI-TOF spectrum found within Rt 2–8 min (insert: deconvoluted masses). Monoisotopic mass (ESI+) calcd. for for $C_{149}H_{226}N_{40}O_{45}$ 3295.6626, found 3295.6539.*

UHPLC

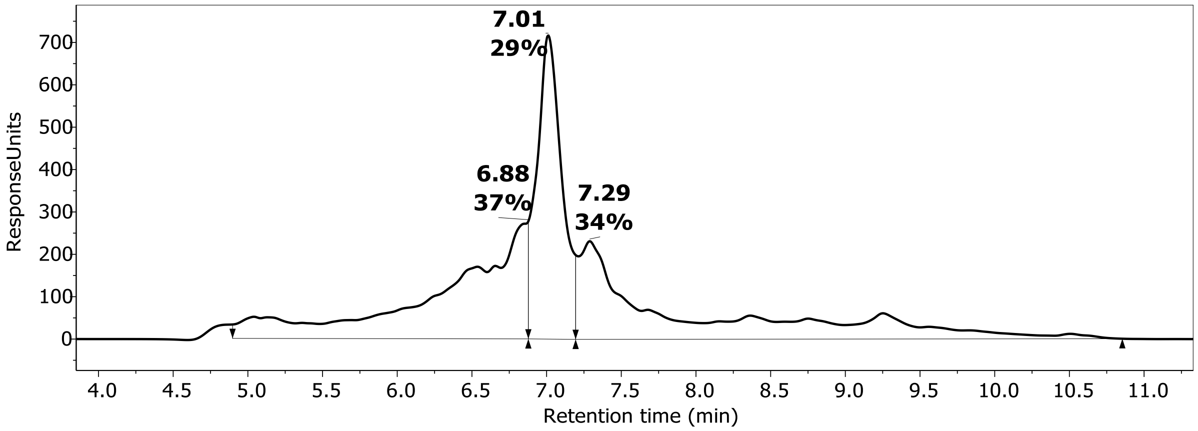

***SI Figure 56**. UHPLC profile of crude Rt 7.01 min, 29% purity based on Area Under Curve (AUC) at λ = 214 nm.*

6.4.5 Shuffling 5

H_2N- AWTLSKEKSQ FVFEDRAGAL HIETGYAGVS-$CONH_2$

The peptide shuffling 5 of GLP-1 was synthesized on commercially available Novabiochem® NovaPEG Rink Amide resin (0.41 mmol/g, 101.1 mg, 41 µmol) using the standard AFPS protocol. (**SI Figure 57**) Total synthesis time to afford resin-bound peptide was approximately 1.5 h. Cleavage of the

peptidyl-resin (16.4 mg, approx. 6.7 µmol) afforded the crude peptide as a colorless solid (6.3 mg, 35% purity by LCMS [**SI Figure 58**], 41% purity by UHPLC [**SI Figure 59**]).

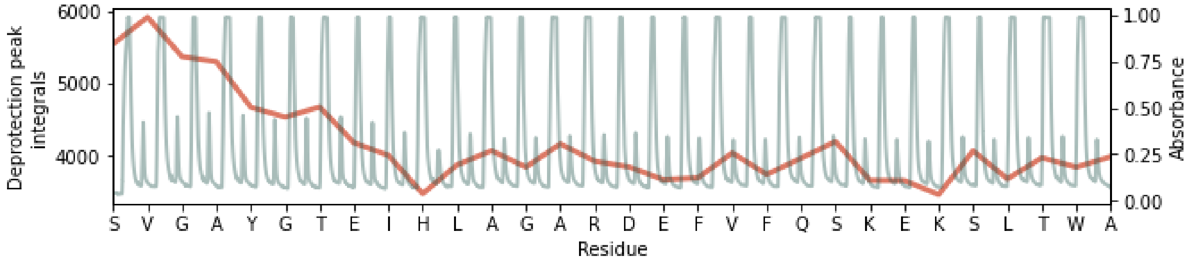

***SI Figure 57**. UV trace (λ = 310 nm) from AFPS of shuffling 5 of GLP-1 (green) and deprotection peak integrals (red).*

LC-MS of crude

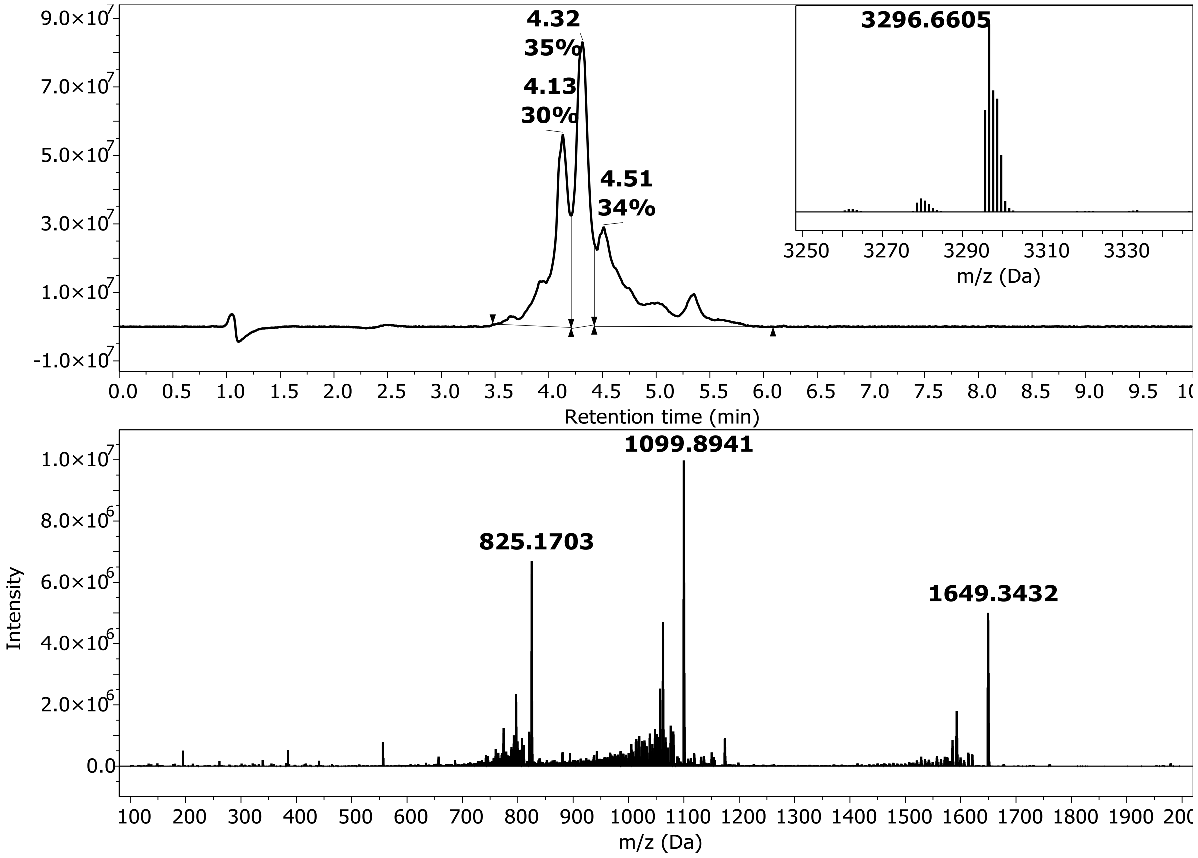

***SI Figure 58**. LCMS Profile of crude Top: Absorbance chromatogram of shuffling 5 of GLP-1 Rt 4.32 min, 35% purity Bottom: ESI-TOF spectrum found within Rt 2–8 min (insert: deconvoluted masses). Monoisotopic mass (ESI+) calcd. for for $C_{149}H_{226}N_{40}O_{45}$ 3295.6626, found 3295.6572.*

UHPLC

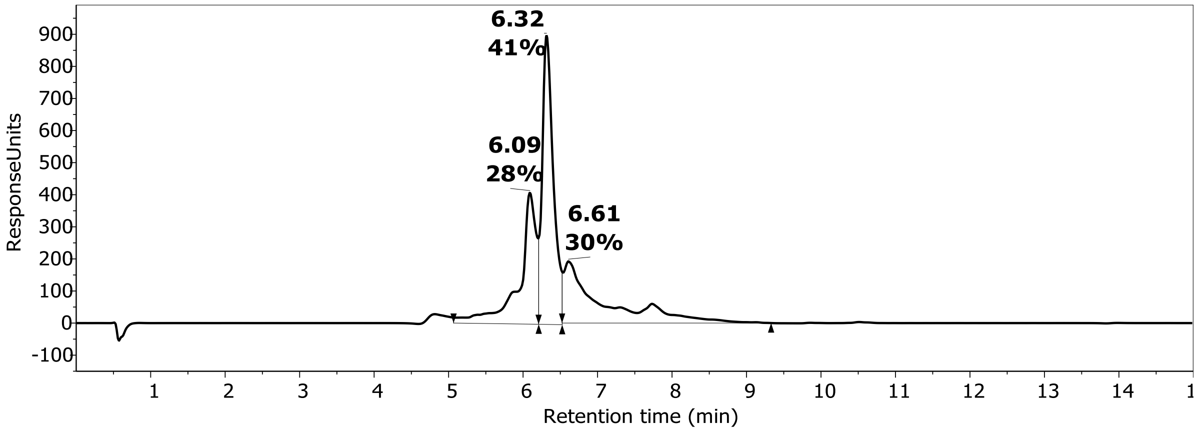

***SI Figure 59**. UHPLC profile of crude Rt 6.32 min, 41% purity based on Area Under Curve (AUC) at λ = 214 nm.*

6.5 MYC[123–143] shuffling

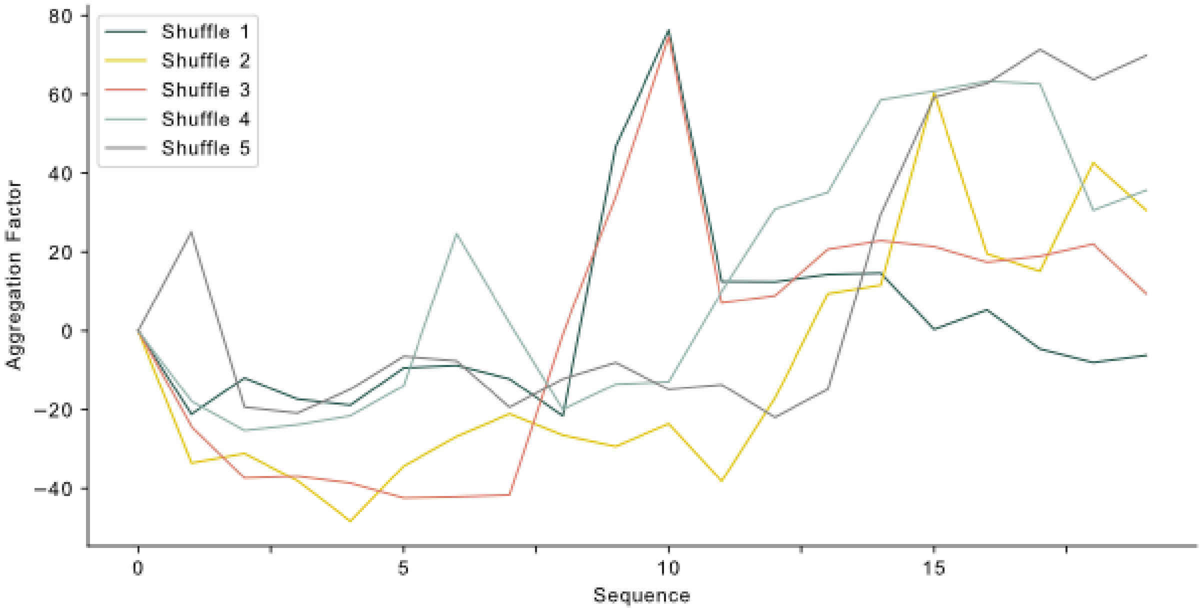

***SI Figure 60**. Plot of aggregation factor values for the different shuffled MYC[123–143] fragments.*

6.5.1 Shuffling 1

H_2N- MAAFFAWKNC IQIKISDIGS-CONH_2

The peptide shuffling 1 of MYC[123–143] was synthesized on commercially available Novabiochem® NovaPEG Rink Amide resin (0.41 mmol/g, 100.8 mg, 41 µmol) using the standard AFPS protocol(**SI Figure 61**) Total synthesis time to afford resin-bound peptide was approximately 1.0 h. Cleavage of the peptidyl-resin (16.8 mg, approx. 6.9 µmol) afforded the crude peptide as a colorless solid (4.4 mg, 28% purity by LCMS [**SI Figure 62**], 41% purity by UHPLC [**SI Figure 63**]).

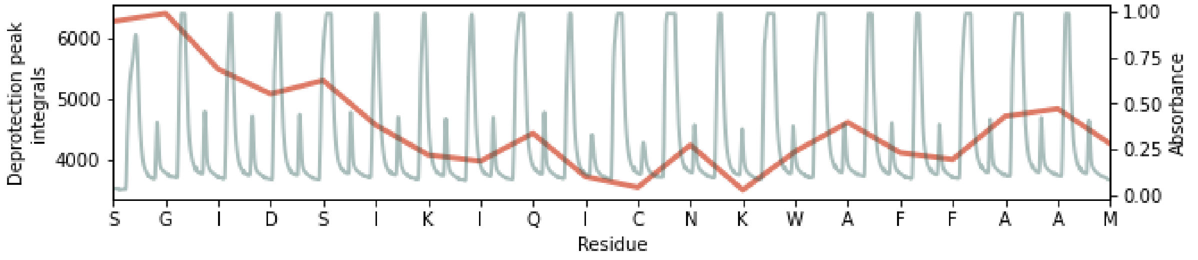

SI Figure 61. UV trace (λ = 310 nm) from AFPS of shuffling 1 of MYC[123–143] (green) and deprotection peak integrals (red).

LC-MS of crude

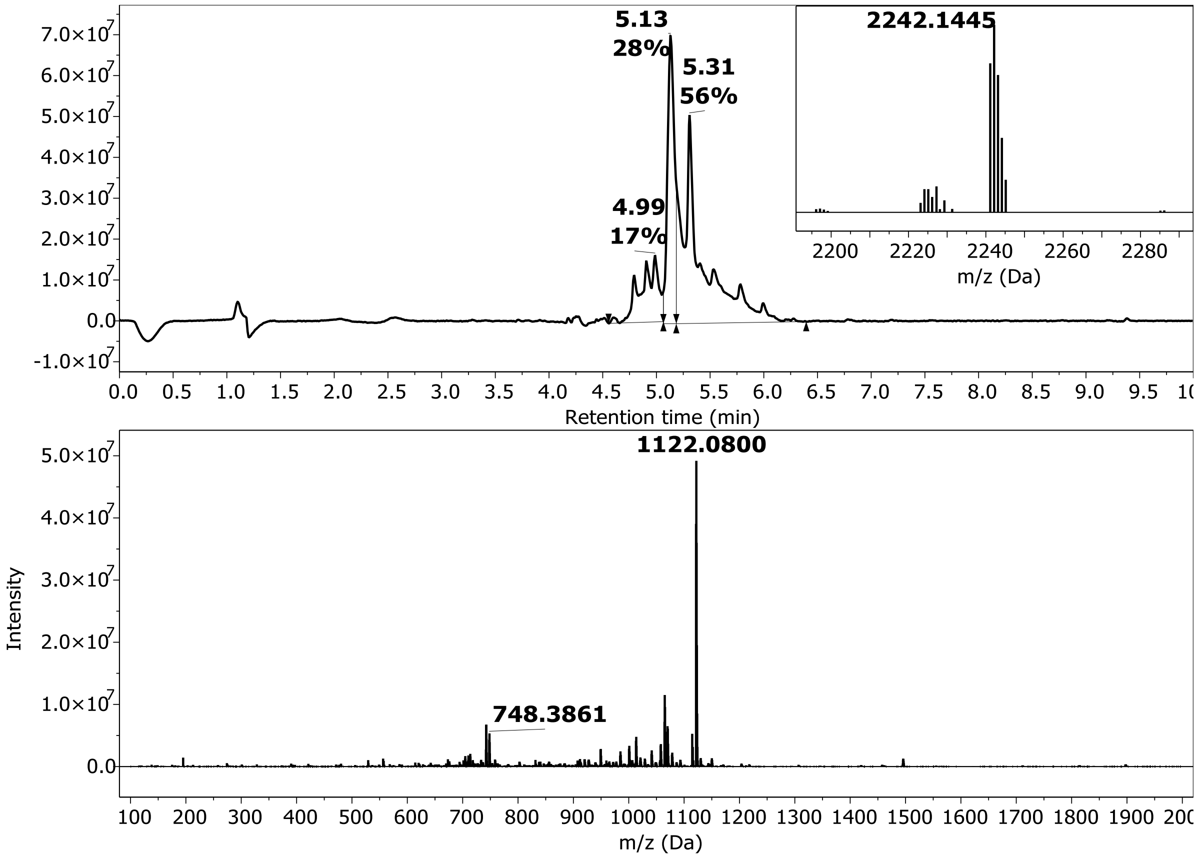

SI Figure 62*. LCMS Profile of crude Top: Absorbance chromatogram of shuffling 1 of MYC[123–143] Rt 5.13 min, 28% purity Bottom: ESI-TOF spectrum found within Rt 2–8 min (insert: deconvoluted masses). Monoisotopic mass (ESI+) calcd. for $C_{103}H_{159}N_{25}O_{27}S_2$ 2241.1439, found 2241.1423.*

UHPLC

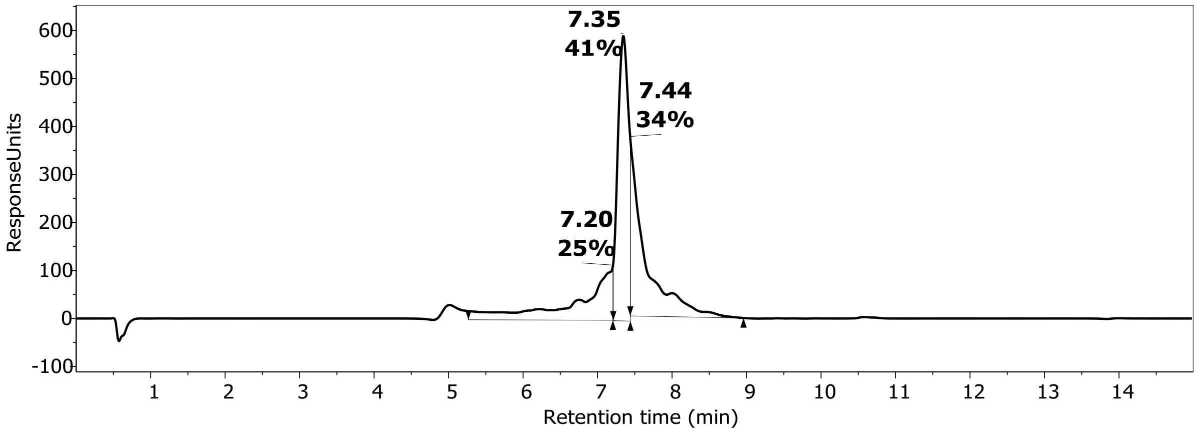

SI Figure 63, UHPLC profile of crude Rt 7.35 min, 41% purity based on Area Under Curve (AUC) at λ = 214 nm.

6.5.2 Shuffling 2

H_2N- WIAKCFAISI MGFSQNDKAI-$CONH_2$

The peptide shuffling 2 of MYC[123–143] was synthesized on commercially available Novabiochem® NovaPEG Rink Amide resin (0.41 mmol/g, 102.1 mg, 42 µmol) using the standard AFPS protocol. (**SI Figure 64**) Total synthesis time to afford resin-bound peptide was approximately 1.0 h. Cleavage of the peptidyl-resin (16.3 mg, approx. 6.7 µmol) afforded the crude peptide as a colorless solid (4.6 mg, 37% purity by LCMS [**SI Figure 65**], 40% purity by UHPLC [**SI Figure 66**]).

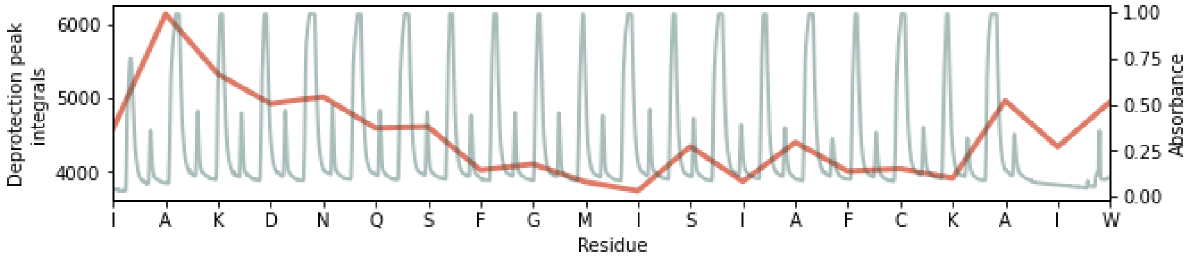

***SI Figure 64**. UV trace (λ = 310 nm) from AFPS of shuffling 2 of MYC[123–143] (green) and deprotection peak integrals (red).*

LC-MS of crude

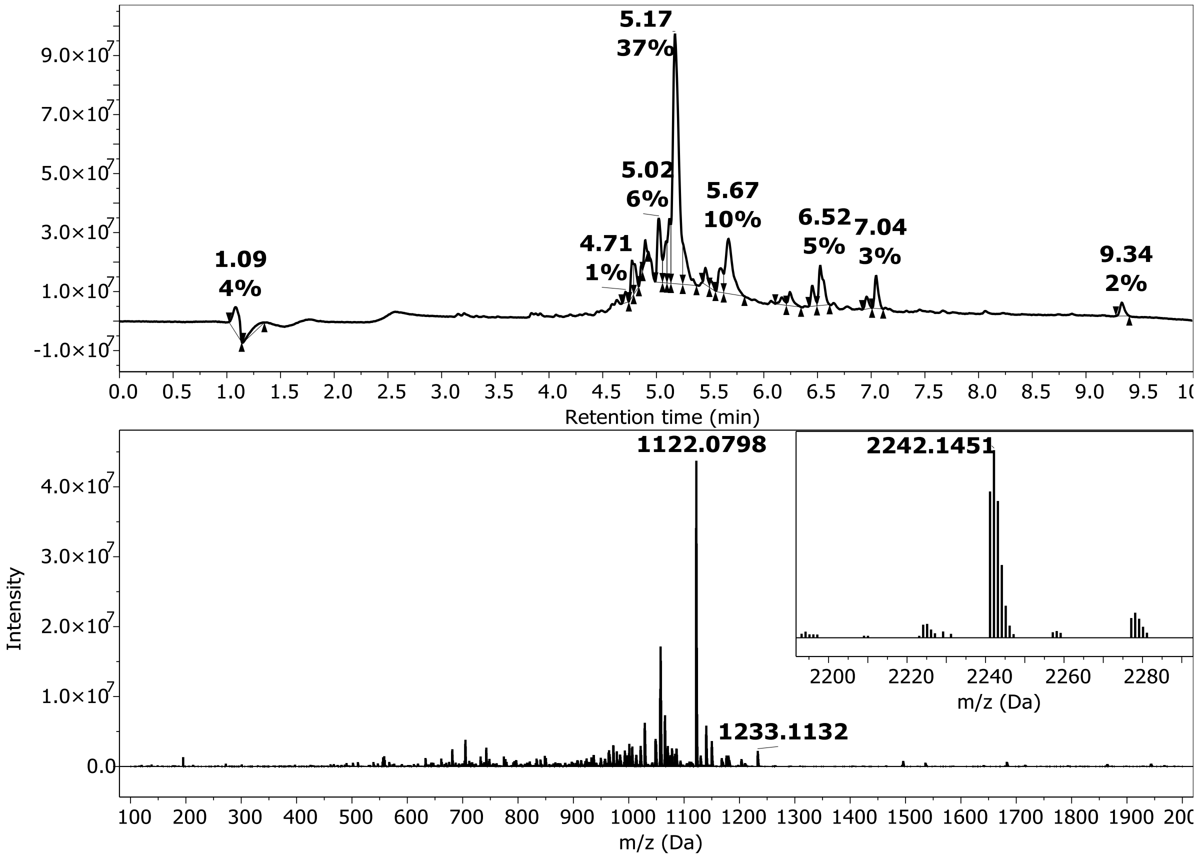

SI Figure 65. LCMS Profile of crude Top: Absorbance chromatogram of shuffling 2 of MYC[123–143] Rt 5.17 min, 37% purity Bottom: ESI-TOF spectrum found within Rt 2–8 min (insert: deconvoluted masses). Monoisotopic mass (ESI+) calcd. for for $C_{103}H_{159}N_{25}O_{27}S_2$ 2241.1439, found 2241.1427.

UHPLC

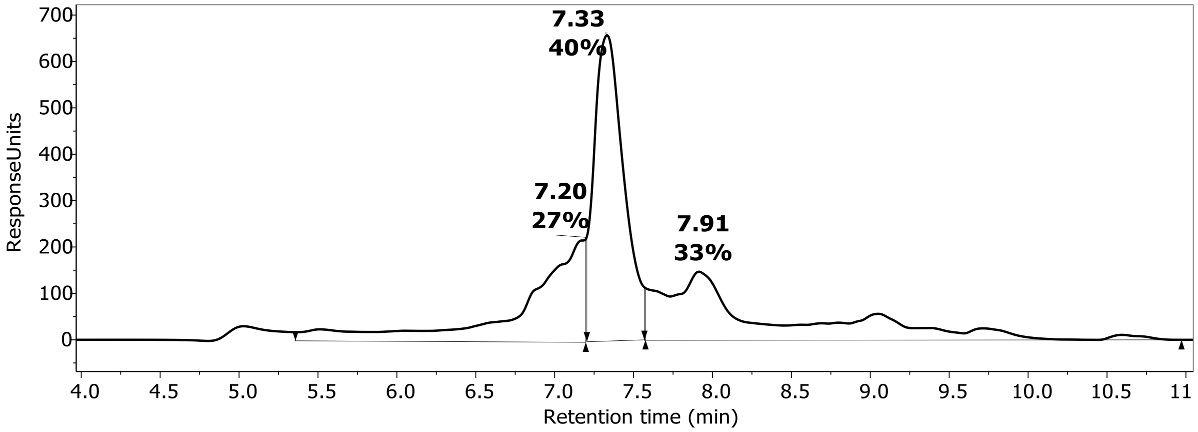

SI Figure 66. UHPLC profile of crude Rt 7.33 min, 40% purity based on Area Under Curve (AUC) at λ = 214 nm.

6.5.3 Shuffling 3

H_2N- QIADKSGFNC FIMSAIWAKI-$CONH_2$

The peptide shuffling 3 of MYC[123–143] was synthesized on commercially available Novabiochem® NovaPEG Rink Amide resin (0.41 mmol/g, 101.5 mg, 42 μmol) using the standard AFPS protocol. (**SI Figure 67**) Total synthesis time to afford resin-bound peptide was approximately 1.0 h. Cleavage of the

peptidyl-resin (16.0 mg, approx. 6.6 µmol) afforded the crude peptide as a colorless solid (3.2 mg, 24% purity by LCMS [**SI Figure 68**], 37% purity by UHPLC [**SI Figure 69**]).

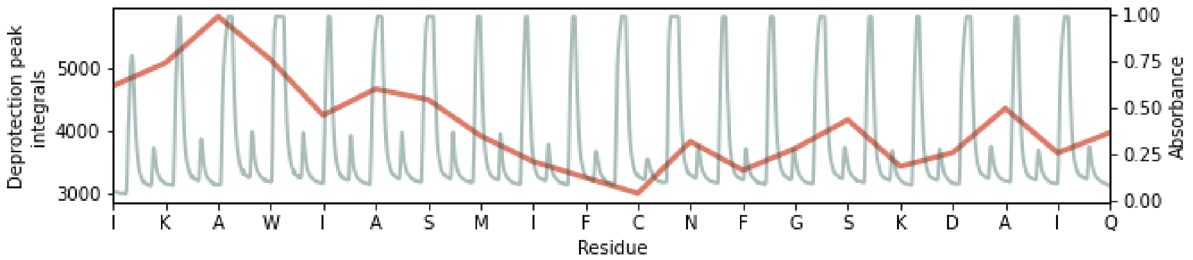

SI Figure 67. *UV trace (λ = 310 nm) from AFPS of shuffling 3 of MYC[123–143] (green) and deprotection peak integrals (red).*

LC-MS of crude

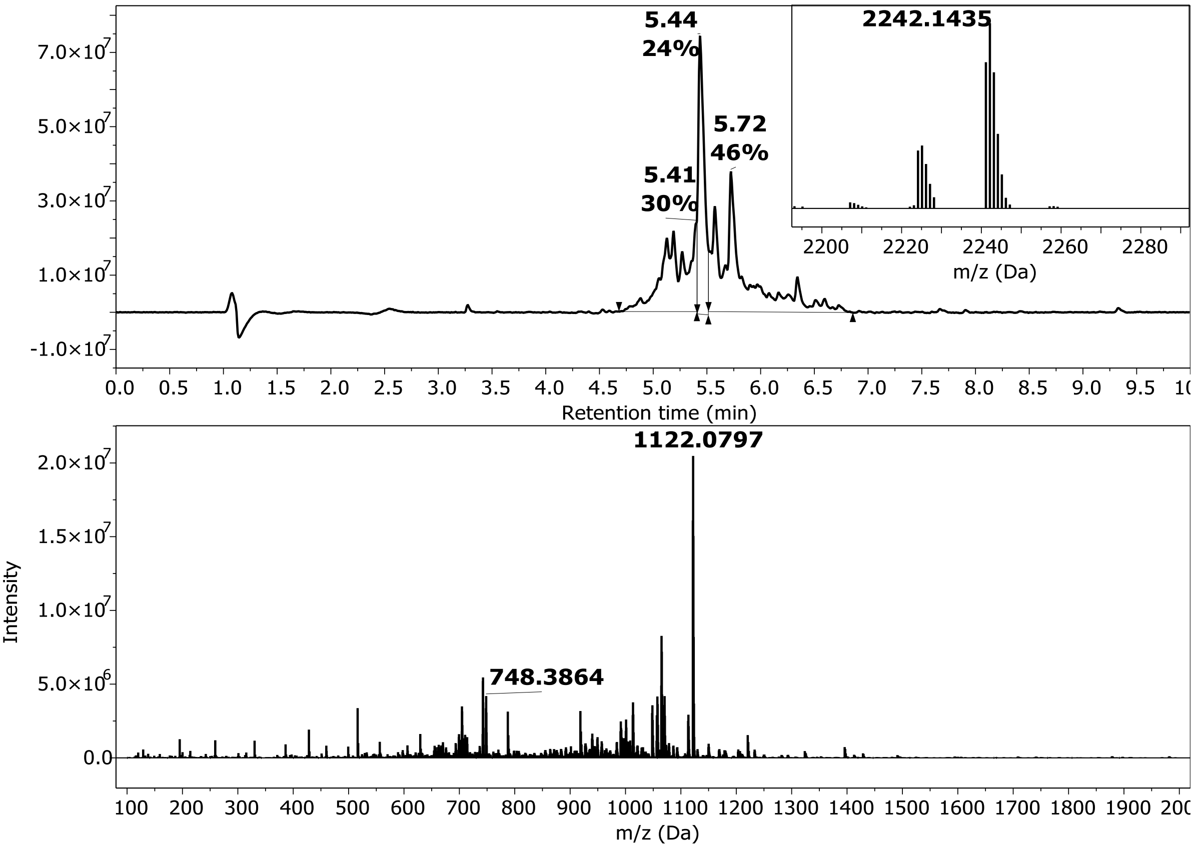

SI Figure 68. *LCMS Profile of crude Top: Absorbance chromatogram of shuffling 3 of MYC[123–143] Rt 5.44 min, 24% purity Bottom: ESI-TOF spectrum found within Rt 2–8 min (insert: deconvoluted masses). Monoisotopic mass (ESI+) calcd. for for $C_{103}H_{159}N_{25}O_{27}S_2$ 2241.1439, found 2241.1414.*

UHPLC

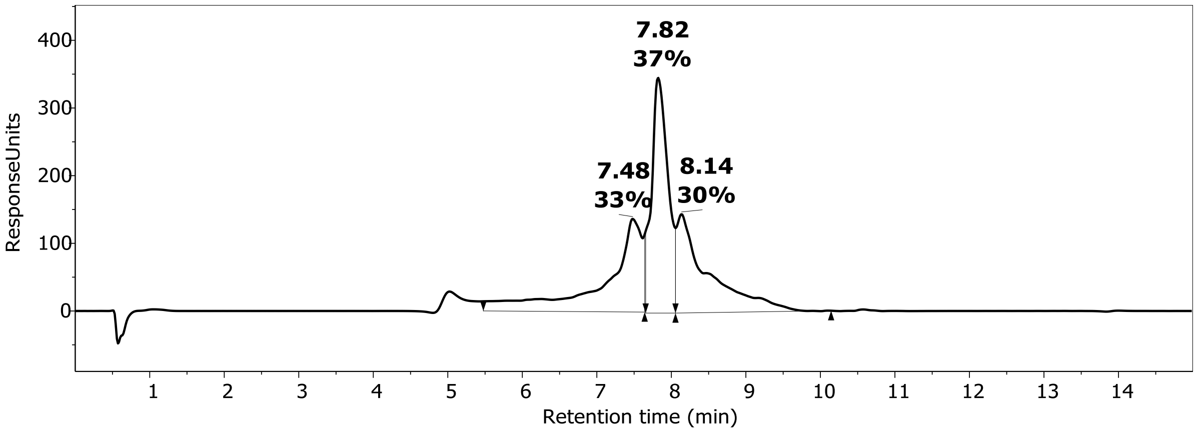

***SI Figure 69**. UHPLC profile of crude Rt 7.82 min, 37% purity based on Area Under Curve (AUC) at λ = 214 nm.*

6.5.4 Shuffling 4

H_2N- DNIISAKSFI GMKCFWIAAQ-$CONH_2$

The peptide shuffling 4 of MYC[123–143] was synthesized on commercially available Novabiochem® NovaPEG Rink Amide resin (0.41 mmol/g, 101.4 mg, 42 µmol) using the standard AFPS protocol. (**SI Figure 70**) Total synthesis time to afford resin-bound peptide was approximately 1.0 h. Cleavage of the peptidyl-resin (17.1 mg, approx. 7.0 µmol) afforded the crude peptide as a colorless solid (5.2 mg, 17% purity by LCMS [**SI Figure 71**], 37% purity by UHPLC [**SI Figure 72**]).

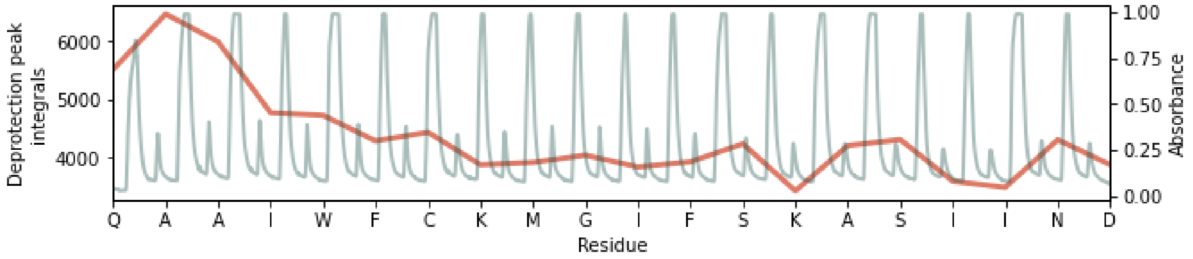

***SI Figure 70**. UV trace (λ = 310 nm) from AFPS of shuffling 4 of MYC[123–143] (green) and deprotection peak integrals (red).*

LC-MS of crude

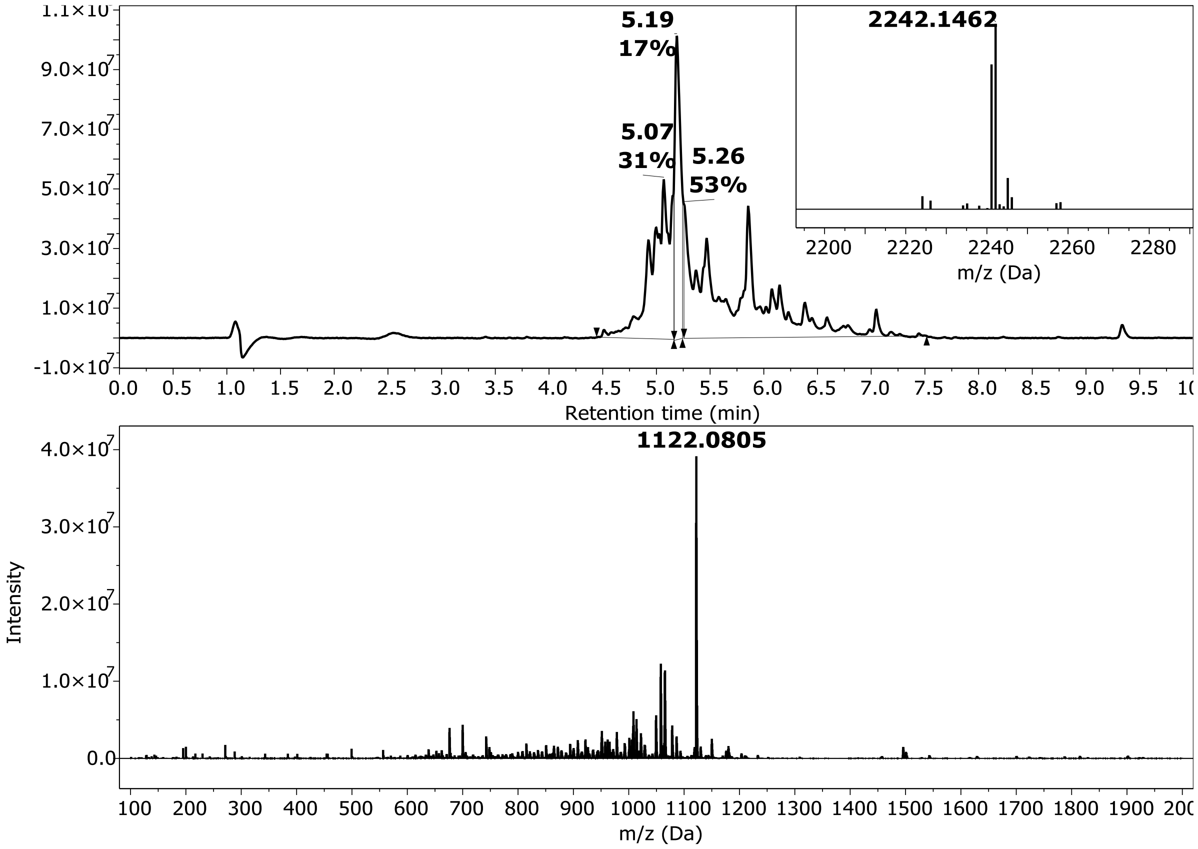

***SI Figure 71**. LCMS Profile of crude Top: Absorbance chromatogram of shuffling 4 of MYC[123–143] Rt 5.19 min, 17% purity Bottom: ESI-TOF spectrum found within Rt 2–8 min (insert: deconvoluted masses). Monoisotopic mass (ESI+) calcd. for for $C_{103}H_{159}N_{25}O_{27}S_2$ 2241.1439, found 2241.1434.*

UHPLC

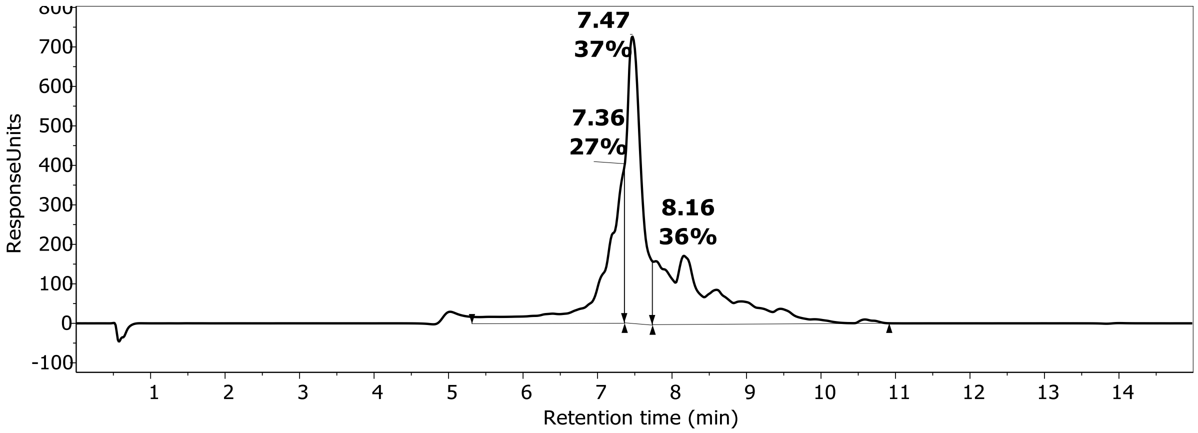

***SI Figure 72**: UHPLC profile of crude Rt 7.47 min, 37% purity based on Area Under Curve (AUC) at λ = 214 nm.*

6.5.5 Shuffling 5

H₂N- AKMFIAAWID IFGIKSSNCQ-CONH₂

The peptide shuffling 5 of MYC[123–143] was synthesized on commercially available Novabiochem® NovaPEG Rink Amide resin (0.41 mmol/g, 101.6 mg, 42 μmol) using the standard AFPS protocol. (**SI Figure 73**) Total synthesis time to afford resin-bound peptide was approximately 1.0 h. Cleavage of the

peptidyl-resin (17.3 mg, approx. 7.1 µmol) afforded the crude peptide as a colorless solid (4.5 mg, 21% purity by LCMS [**SI Figure 74**], 33% purity by UHPLC [**SI Figure 75**]).

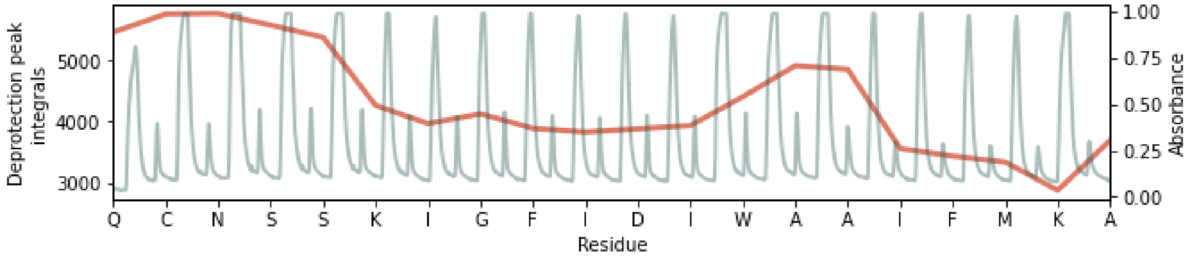

SI Figure 73. *UV trace (λ = 310 nm) from AFPS of shuffling 5 of MYC[123–143] (green) and deprotection peak integrals (red).*

LC-MS of crude

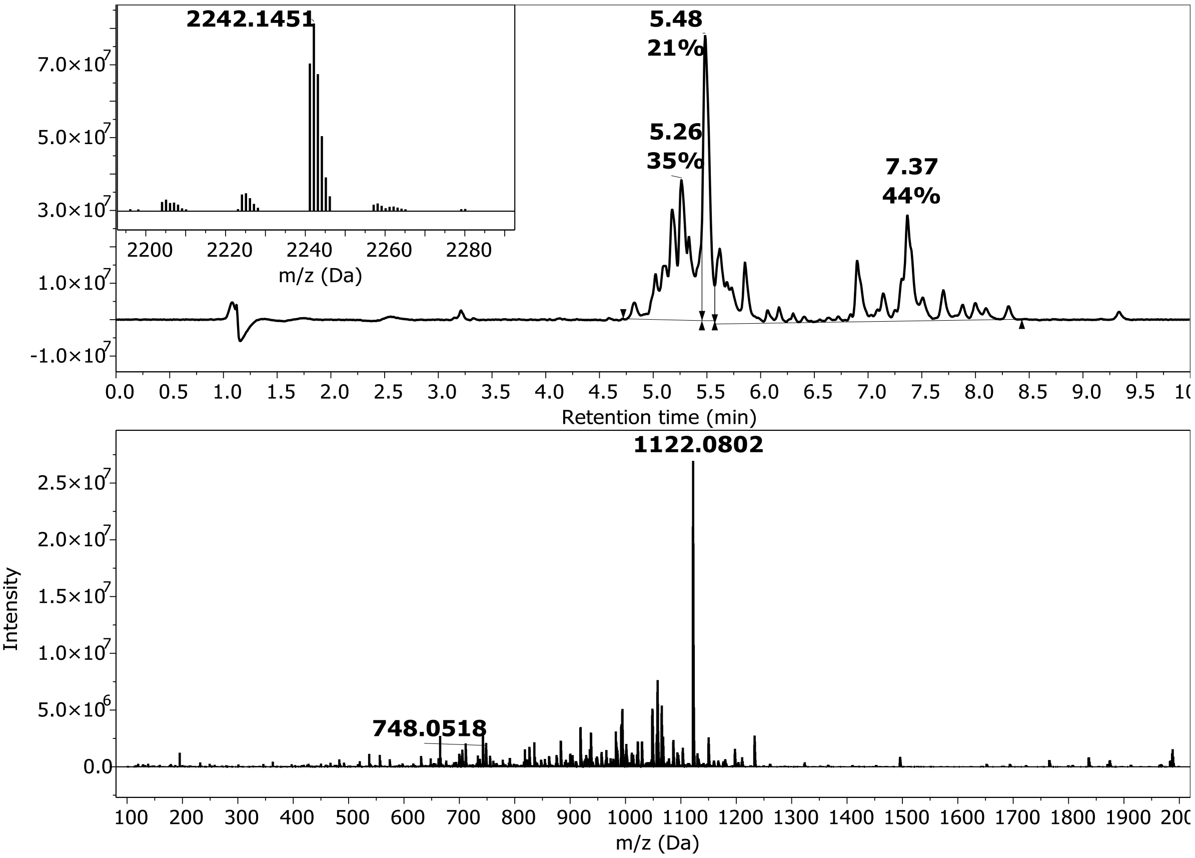

SI Figure 74*. LCMS Profile of crude Top: Absorbance chromatogram of shuffling 5 of MYC[123–143] Rt 5.48 min, 21% purity Bottom: ESI-TOF spectrum found within Rt 2–8 min (insert: deconvoluted masses). Monoisotopic mass (ESI+) calcd. for for $C_{103}H_{159}N_{25}O_{27}S_2$ 2241.1439, found 2241.1434.*

UHPLC

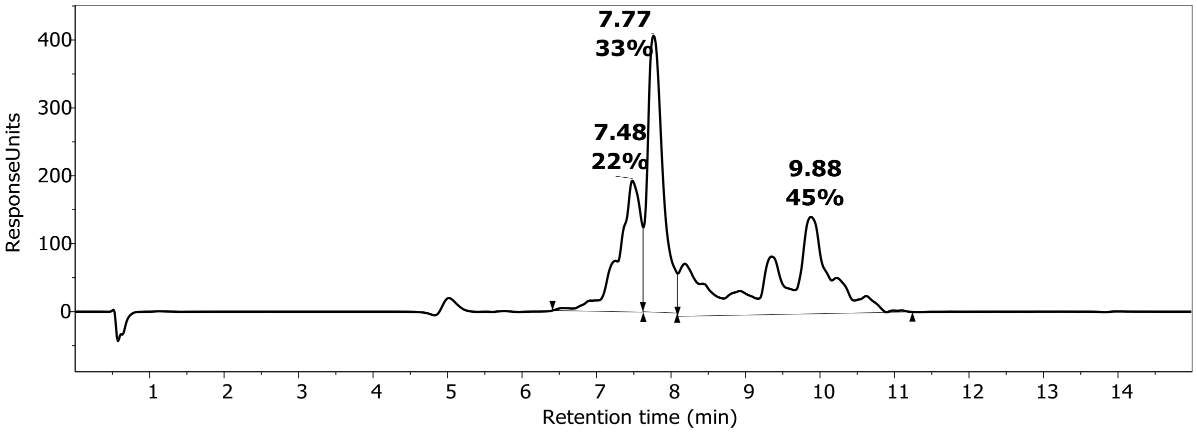

***SI Figure 75**. UHPLC profile of crude Rt 7.77 min, 33% purity based on Area Under Curve (AUC) at λ = 214 nm.*

6.6 NBDY [41–68] shuffling

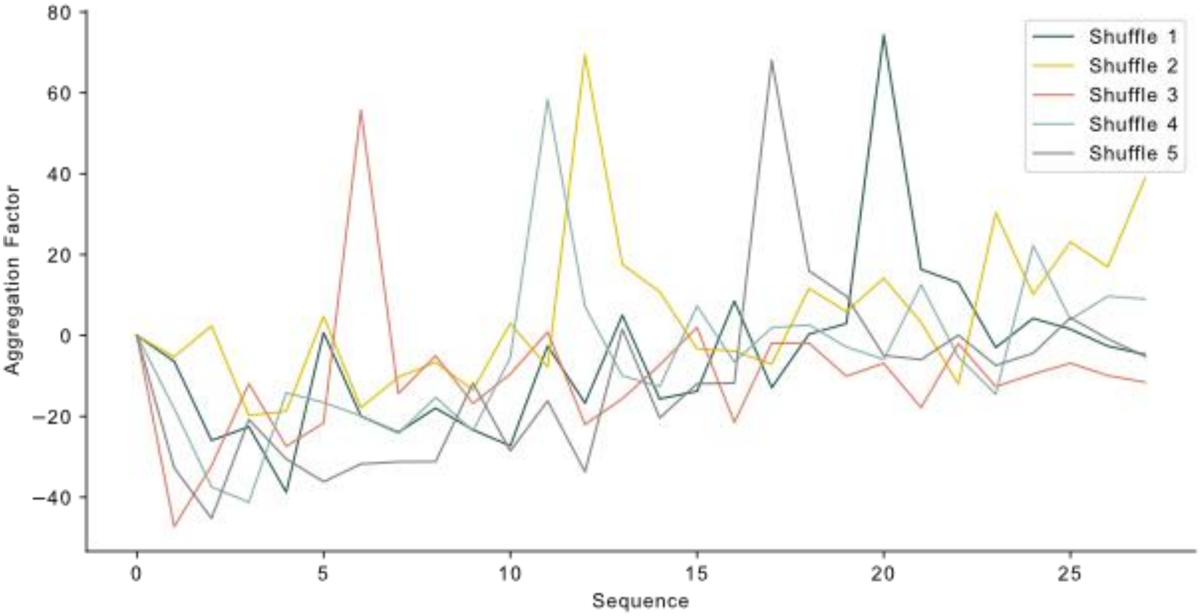

***SI Figure 76**. Plot of aggregation factor values for the different shuffled NBDY[41–68] fragments.*

6.6.1 Shuffling 1

H_2N- GAAGTPLHKL PPSGPTPSSE SAPNPPPK-$CONH_2$

The peptide shuffling 1 of NBDY[41–68] was synthesized on commercially available Novabiochem® NovaPEG Rink Amide resin (0.41 mmol/g, 100.9 mg, 41 µmol) using the standard AFPS protocol(**SI Figure 77**) Total synthesis time to afford resin-bound peptide was approximately 1.4 h. Cleavage of the peptidyl-resin (16.4 mg, approx. 6.7 µmol) afforded the crude peptide as a colorless solid (4.7 mg, 52% purity by LCMS [**SI Figure 78**], 66% purity by UHPLC [**SI Figure 79**]).

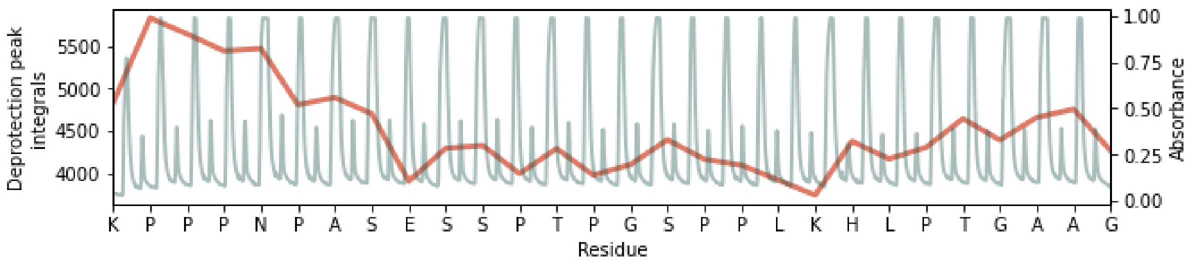

SI Figure 77. *UV trace (λ = 310 nm) from AFPS of shuffling 1 of NBDY[41–68] (green) and deprotection peak integrals (red).*

LC-MS of crude

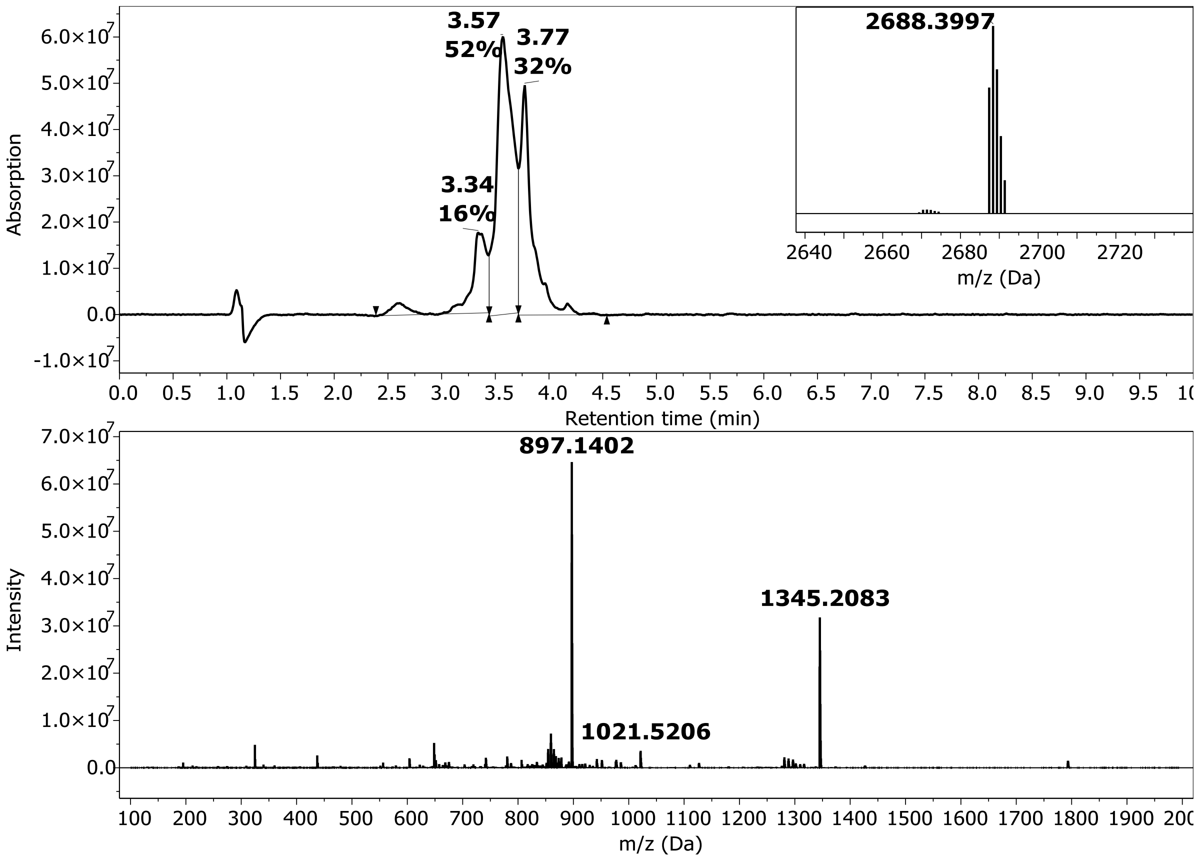

SI Figure 78. *LCMS Profile of crude Top: Absorbance chromatogram of shuffling 1 of NBDY[41–68] Rt 3.57 min, 52% purity Bottom: ESI-TOF spectrum found within Rt 2–8 min (insert: deconvoluted masses). Monoisotopic mass (ESI+) calcd. for* $C_{119}H_{190}N_{34}O_{37}$ *2687.4031, found 2687.3946.*

UHPLC

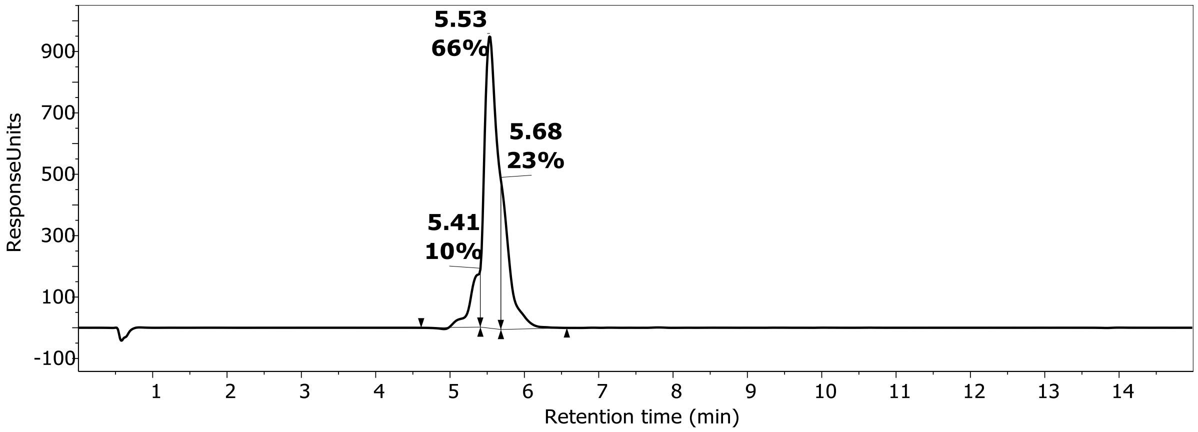

SI Figure 79. UHPLC profile of crude Rt 5.53 min, 66% purity based on Area Under Curve (AUC) at λ = 214 nm.

6.6.2 Shuffling 2

H_2N- PEPAPNTKKP SGAPLHTPSA SGPGPPLS-$CONH_2$

The peptide shuffling 2 of NBDY[41–68] was synthesized on commercially available Novabiochem® NovaPEG Rink Amide resin (0.41 mmol/g, 101.3 mg, 41 µmol) using the standard AFPS protocol. (**SI Figure 80**) Total synthesis time to afford resin-bound peptide was approximately 1.4 h. Cleavage of the peptidyl-resin (15.8 mg, approx. 6.5 µmol) afforded the crude peptide as a colorless solid (5.3 mg, 42% purity by LCMS [**SI Figure 81**], 74% purity by UHPLC [**SI Figure 82**]).

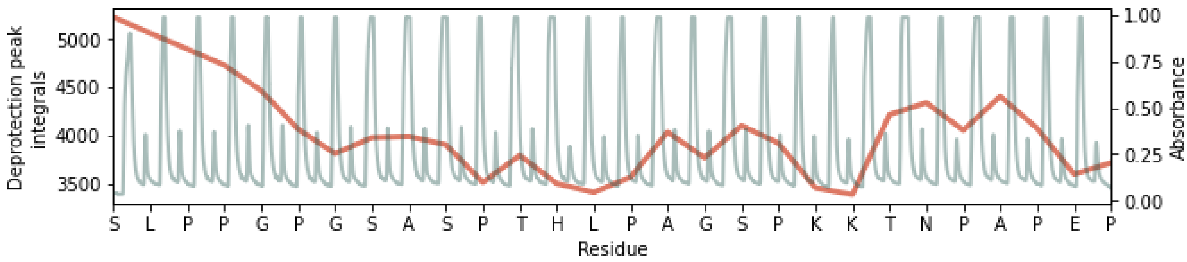

SI Figure 80. UV trace (λ = 310 nm) from AFPS of shuffling 2 of NBDY[41–68] (green) and deprotection peak integrals (red).

LC-MS of crude

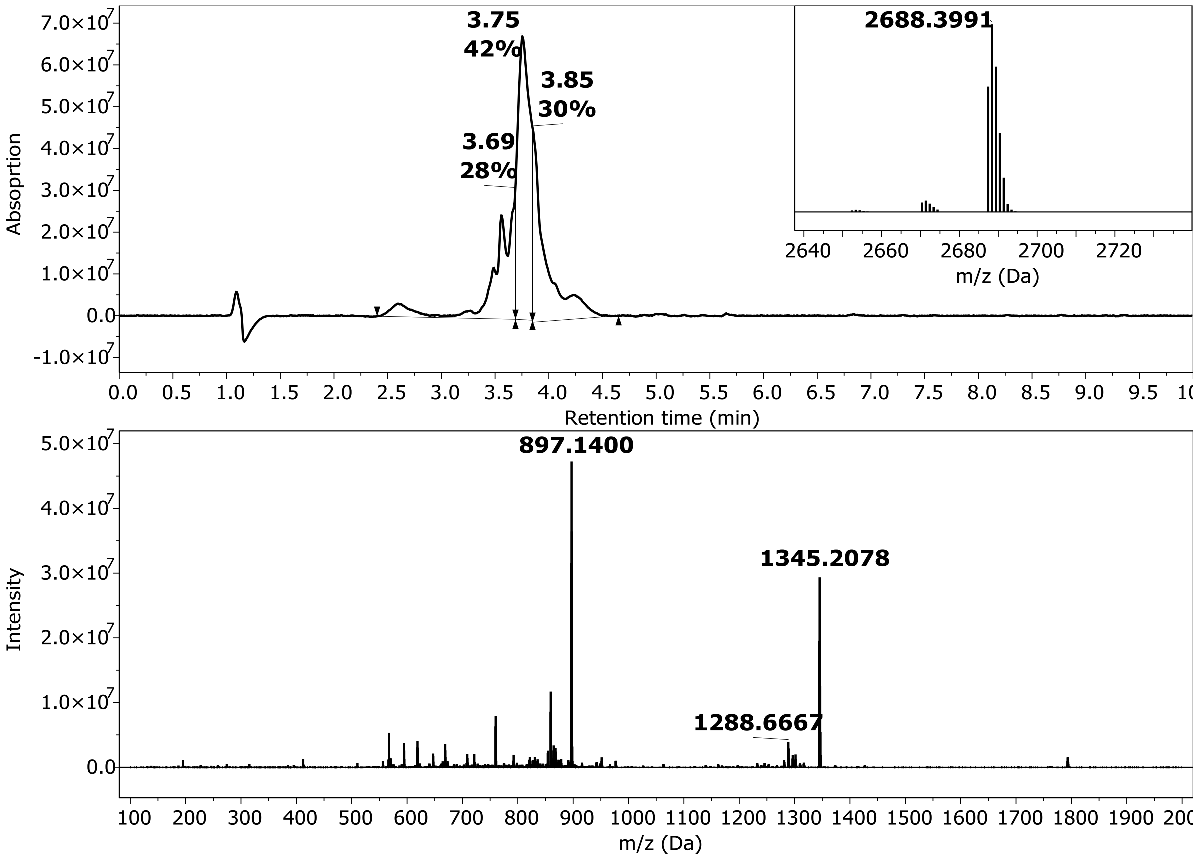

***SI Figure 81**. LCMS Profile of crude Top: Absorbance chromatogram of shuffling 2 of NBDY[41–68] Rt 3.75 min, 42% purity Bottom: ESI-TOF spectrum found within Rt 2–8 min (insert: deconvoluted masses). Monoisotopic mass (ESI+) calcd. for for $C_{119}H_{190}N_{34}O_{37}$ 2687.4031, found 2687.3942.*

UHPLC

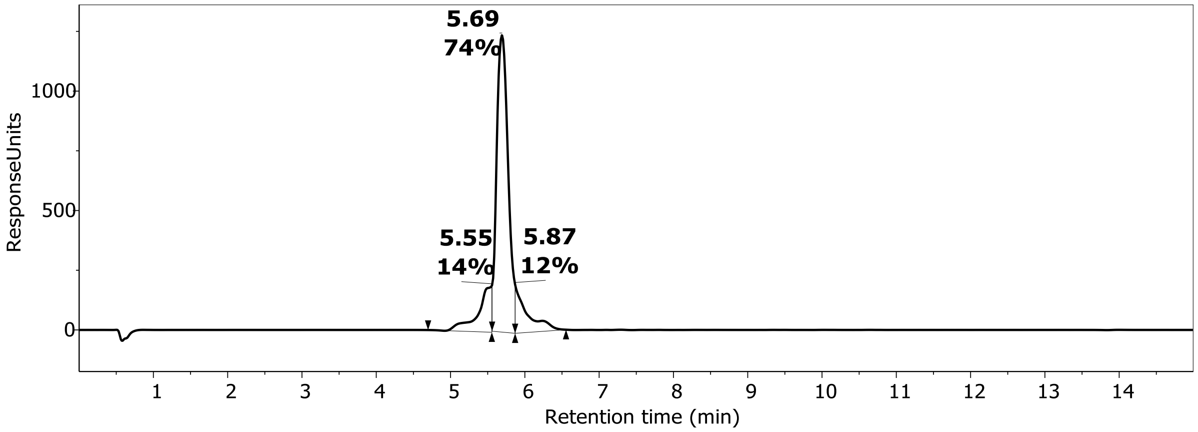

***SI Figure 82**: UHPLC profile of crude Rt 5.69 min, 74% purity based on Area Under Curve (AUC) at λ = 214 nm.*

6.6.3 Shuffling 3

H_2N- GSGSPPSLAA KPPLAPPETP SHTPPGNK-$CONH_2$

The peptide shuffling 3 of NBDY[41–68] was synthesized on commercially available Novabiochem® NovaPEG Rink Amide resin (0.41 mmol/g, 101.3 mg, 41 µmol) using the standard AFPS protocol. (**SI Figure 83**) Total synthesis time to afford resin-bound peptide was approximately 1.4 h. Cleavage of the

peptidyl-resin (17.5 mg, approx. 7.2 µmol) afforded the crude peptide as a colorless solid (5.4 mg, 39% purity by LCMS [**SI Figure 84**], 78% purity by UHPLC [**SI Figure 85**]).

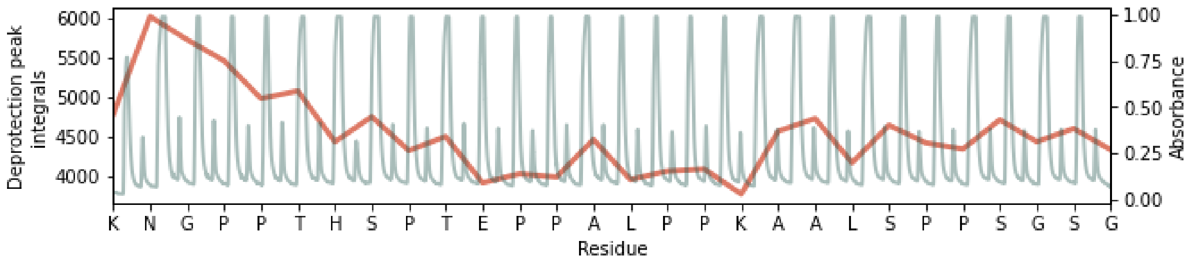

SI Figure 83. *UV trace (λ = 310 nm) from AFPS of shuffling 3 of NBDY[41–68] (green) and deprotection peak integrals (red).*

LC-MS of crude

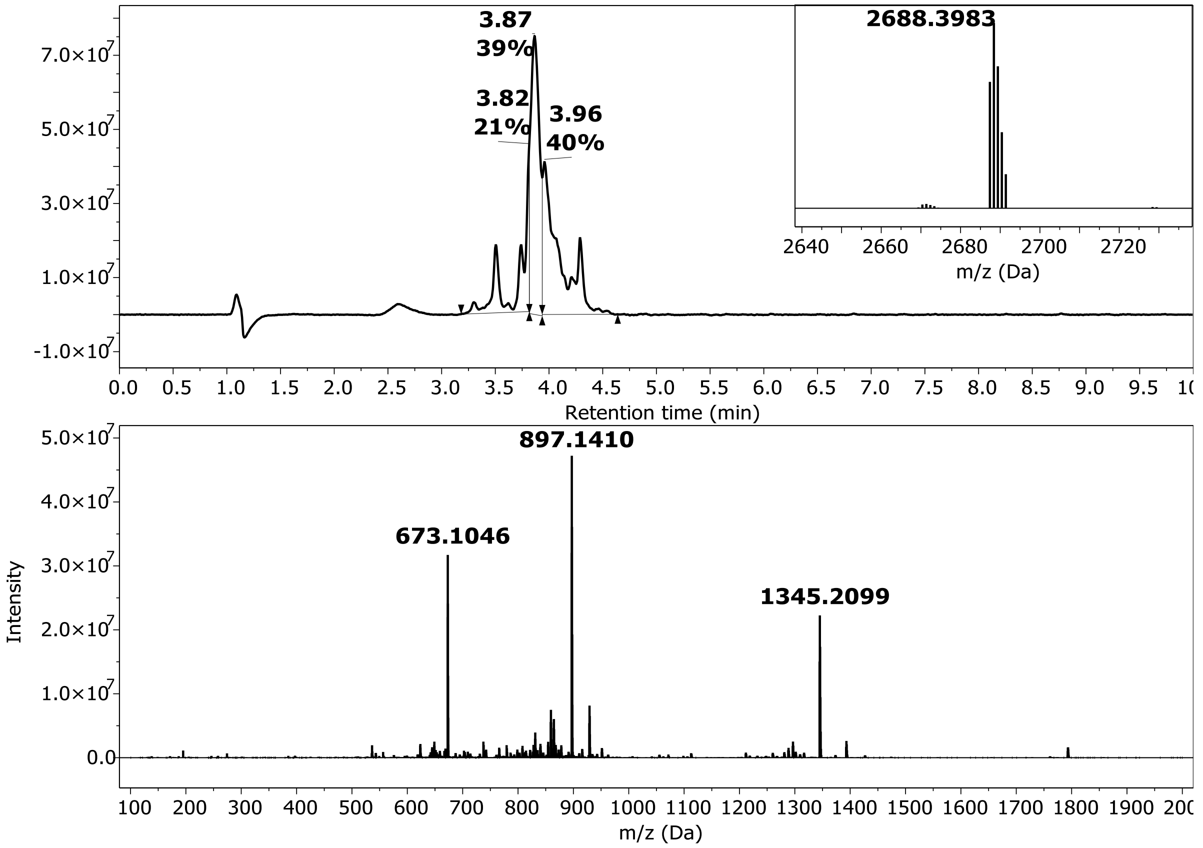

SI Figure 84. *LCMS Profile of crude Top: Absorbance chromatogram of shuffling 3 of NBDY[41–68] Rt 3.87 min, 39% purity Bottom: ESI-TOF spectrum found within Rt 2–8 min (insert: deconvoluted masses). Monoisotopic mass (ESI+) calcd. for for $C_{119}H_{190}N_{34}O_{37}$ 2687.4031, found 2687.3938.*

UHPLC

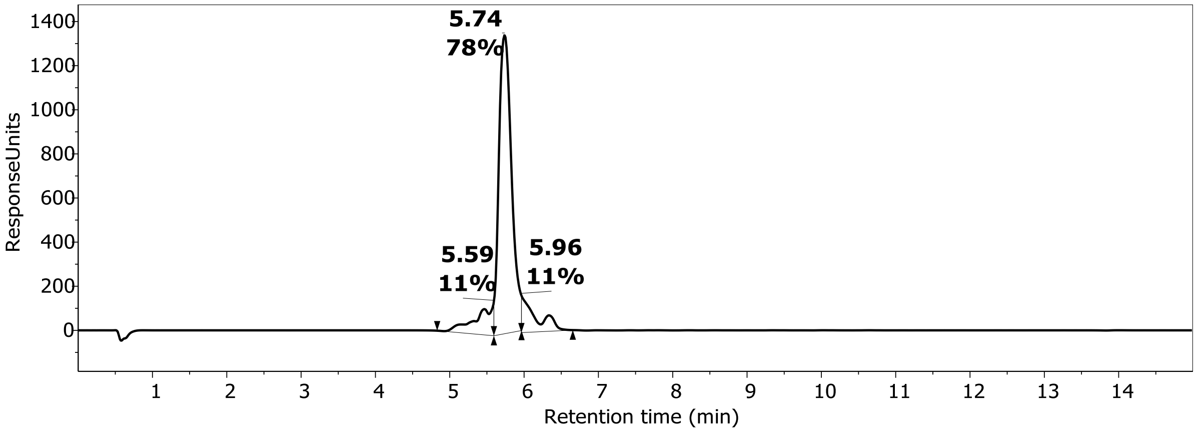

***SI Figure 85**: UHPLC profile of crude Rt 5.74 min, 78% purity based on Area Under Curve (AUC) at λ = 214 nm.*

6.6.4 Shuffling 4

H_2N- TPPPNTPASA KPPGAPHPGE SSKPSGLL-$CONH_2$

The peptide shuffling 4 of NBDY [41–68] was synthesized on commercially available Novabiochem® NovaPEG Rink Amide resin (0.41 mmol/g, 99.1 mg, 42 µmol) using the standard AFPS protocol. (**SI Figure 86**) Total synthesis time to afford resin-bound peptide was approximately 1.4 h. Cleavage of the peptidyl-resin (17.6 mg, approx. 7.2 µmol) afforded the crude peptide as a colorless solid (5.5 mg, 54% purity by LCMS [**SI Figure 87**], 77% purity by UHPLC [**SI Figure 88**]).

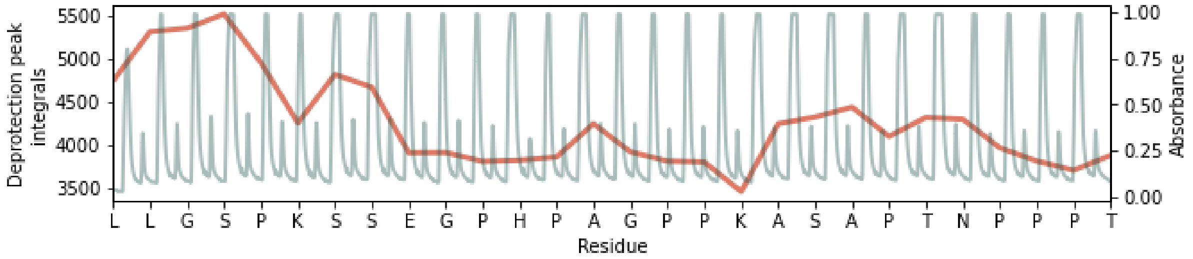

***SI Figure 86**: UV trace (λ = 310 nm) from AFPS of shuffling 4 of NBDY [41–68] (green) and deprotection peak integrals (red).*

LC-MS of crude

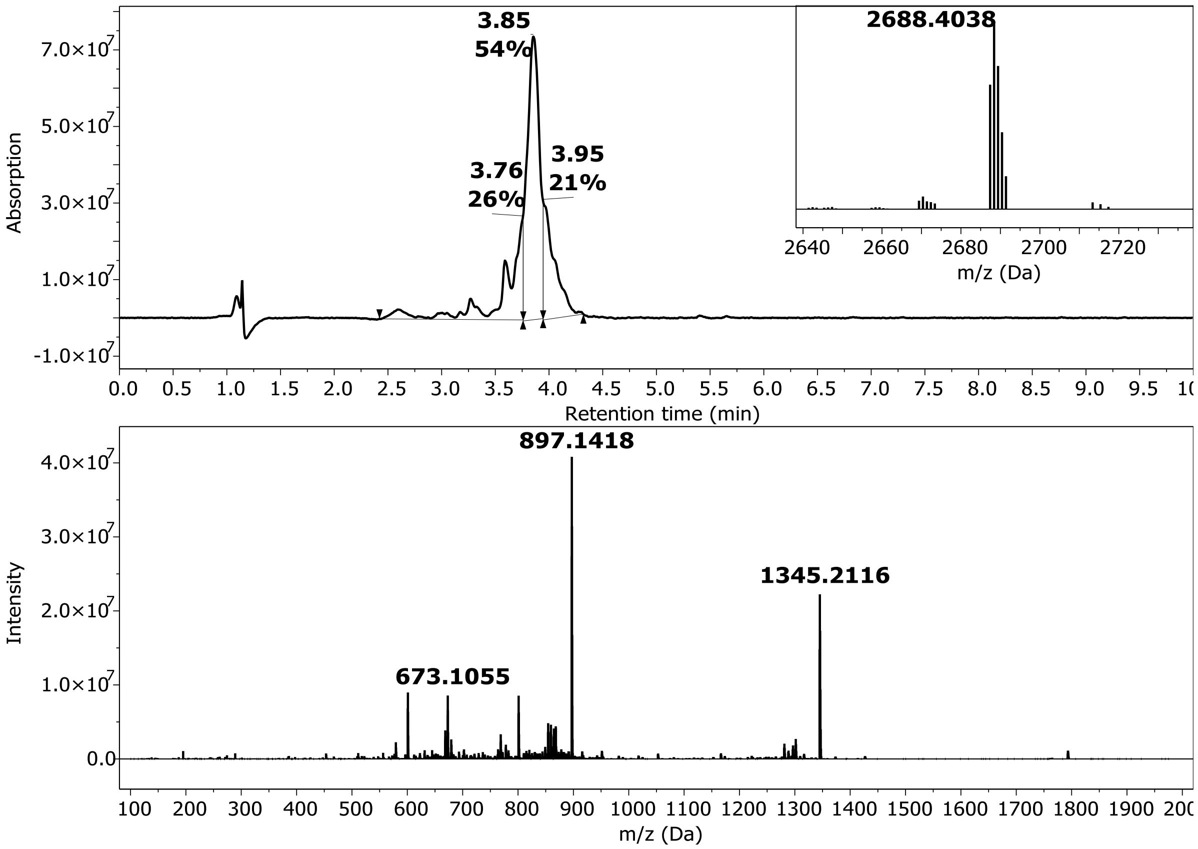

***SI Figure 87**. LCMS Profile of crude Top: Absorbance chromatogram of shuffling 4 of NBDY[41–68] Rt 3.85 min, 54% purity Bottom: ESI-TOF spectrum found within Rt 2–8 min (insert: deconvoluted masses). Monoisotopic mass (ESI+) calcd. for for $C_{119}H_{190}N_{34}O_{37}$ 2687.4031, found 2687.3995.*

UHPLC

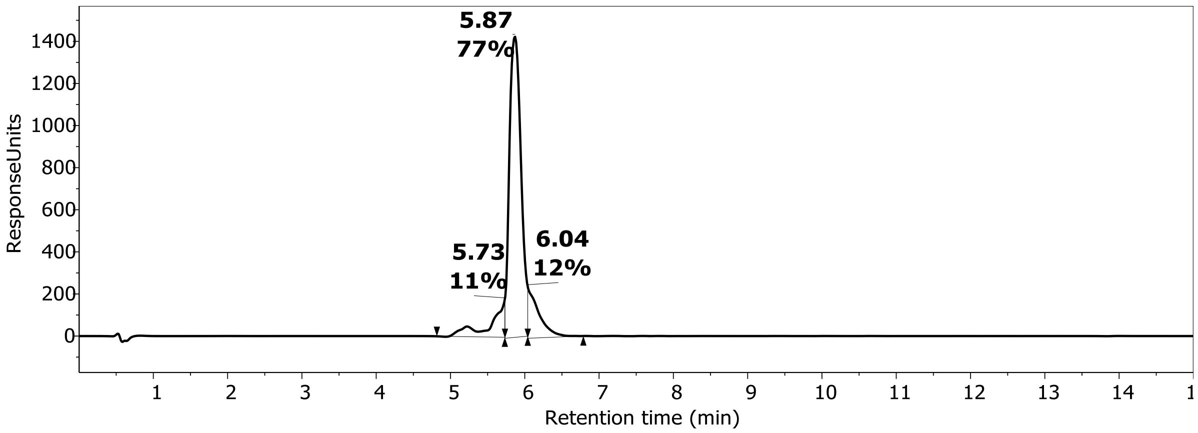

***SI Figure 88**: UHPLC profile of crude Rt 5.87 min, 77% purity based on Area Under Curve (AUC) at λ = 214 nm.*

6.6.5 Shuffling 5

H_2N- TAPKALGAPK HTPPPNEPPS GSSPPGSL-$CONH_2$

The peptide shuffling 5 of NBDY[41–68] was synthesized on commercially available Novabiochem® NovaPEG Rink Amide resin (0.41 mmol/g, 101.9 mg, 42 µmol) using the standard AFPS protocol. (**SI Figure 89**) Total synthesis time to afford resin-bound peptide was approximately 1.4 h. Cleavage of the

peptidyl-resin (17.4 mg, approx. 7.1 μmol) afforded the crude peptide as a colorless solid (5.6 mg, 49% purity by LCMS [**SI Figure 90**], 75% purity by UHPLC [**SI Figure 91**]).

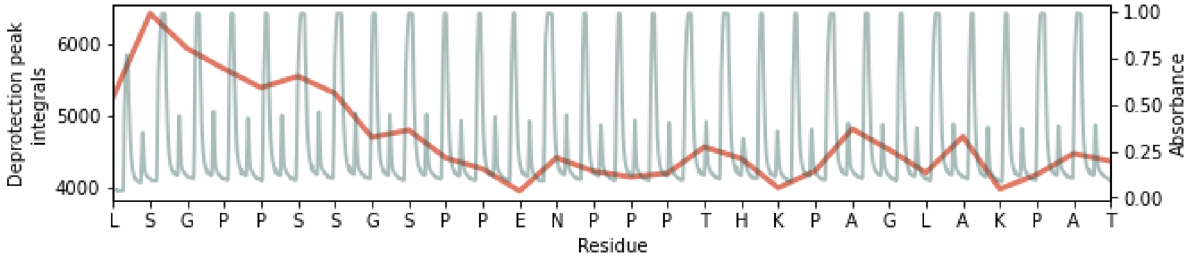

SI Figure 89. *UV trace (λ = 310 nm) from AFPS of shuffling 5 of NBDY[41–68] (green) and deprotection peak integrals (red).*

LC-MS of crude

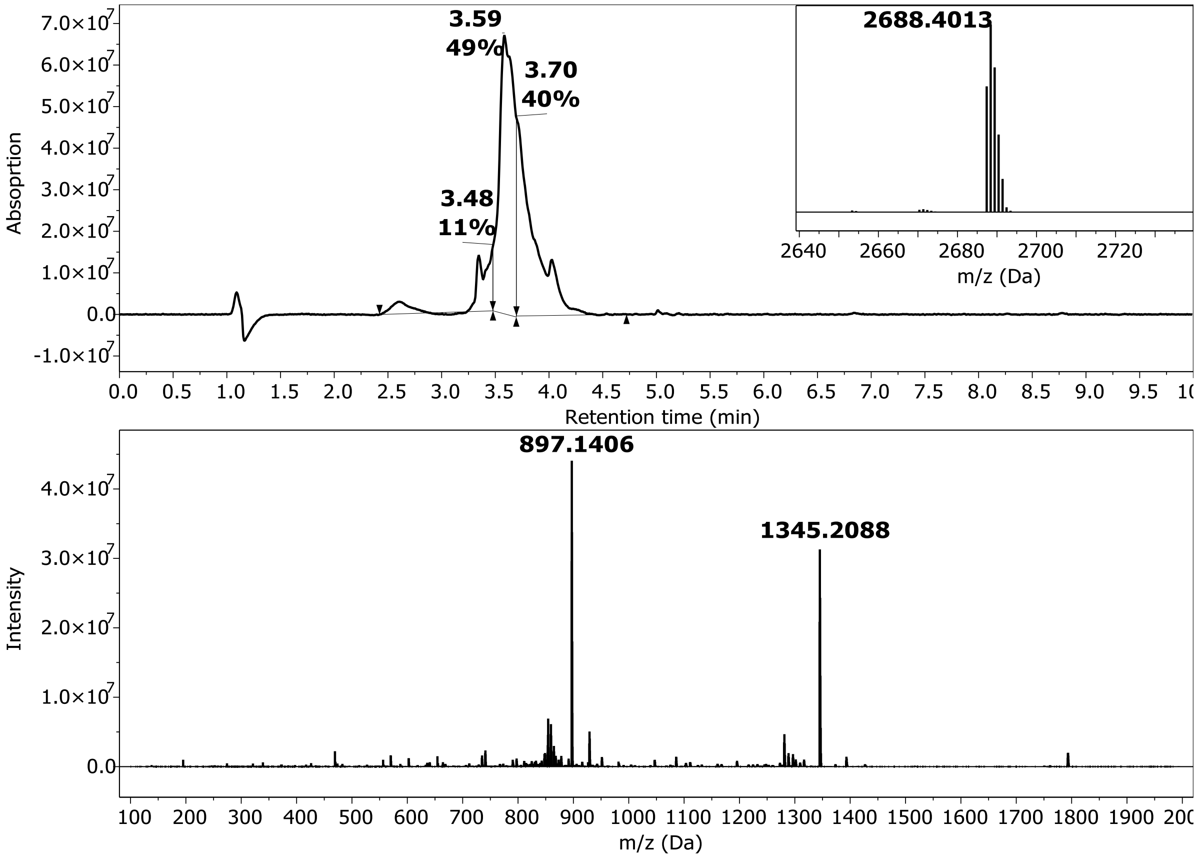

SI Figure 90. *LCMS Profile of crude Top: Absorbance chromatogram of shuffling 5 of NBDY[41–68] Rt 3.59 min, 49% purity Bottom: ESI-TOF spectrum found within Rt 2–8 min (insert: deconvoluted masses). Monoisotopic mass (ESI+) calcd. for for $C_{119}H_{190}N_{34}O_{37}$ 2687.4031, found 2687.3970.*

UHPLC

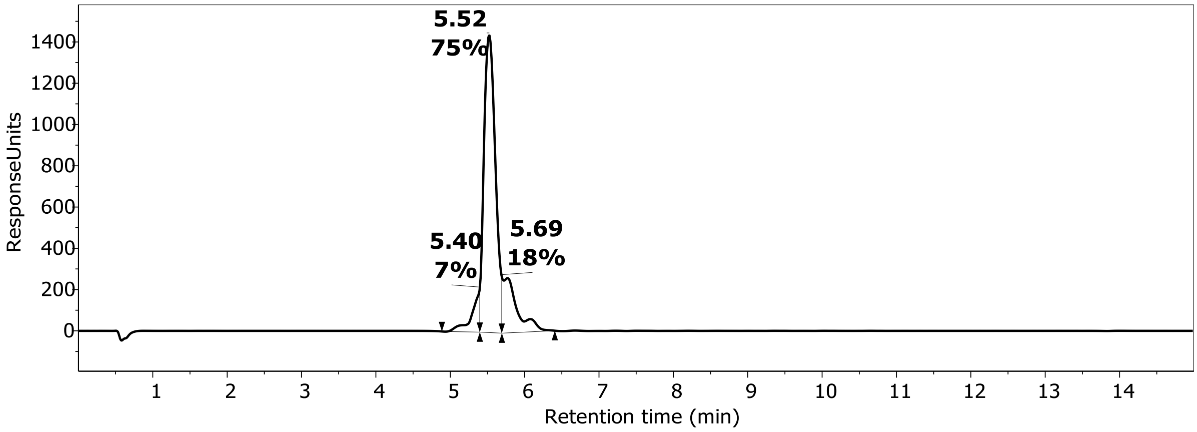

SI Figure 91. *UHPLC profile of crude Rt 5.52 min, 75% purity based on Area Under Curve (AUC) at λ = 214 nm.*

6.7 GHRH shuffling

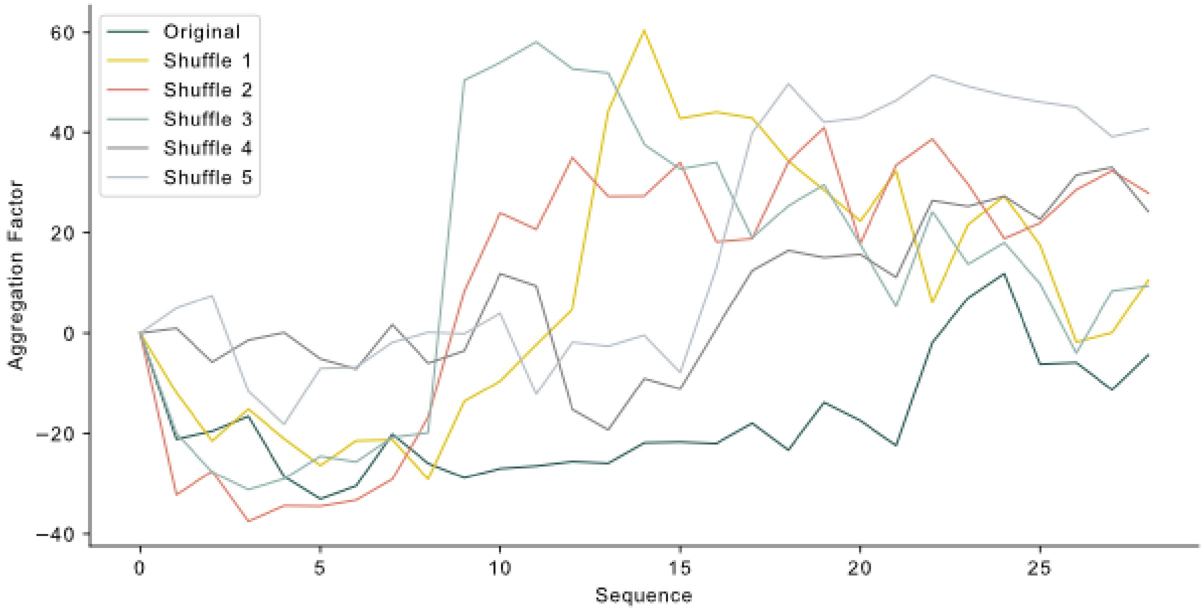

SI Figure 92*: Plot of aggregation factor values for the different shuffled GHRH fragments*

6.7.1 Original sequence

H_2N- YADAIFTNSY RKVLGQLSAR KLLQDILSA-$CONH_2$

The peptide GHRH was synthesized on commercially available Novabiochem® NovaPEG Rink Amide resin (0.41 mmol/g, 100.9 mg, 41 µmol) using the standard AFPS protocol. (**SI Figure 93**) Total synthesis time to afford resin-bound peptide was approximately 1.5 h. Cleavage of the peptidyl-resin (16.4 mg, approx. 6.7 µmol) afforded the crude peptide as a colorless solid (6.0 mg, 45% purity by LCMS [**SI Figure 94**], 73% purity by UHPLC [**SI Figure 95**]).

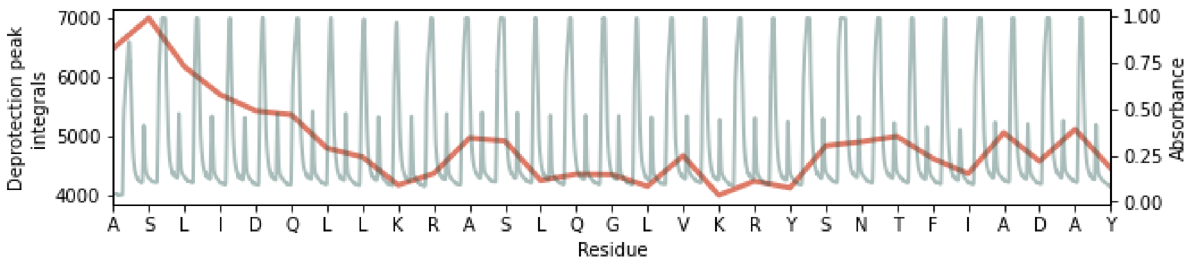

SI Figure 93: *UV trace (λ = 310 nm) from AFPS of GHRH (green) and deprotection peak integrals (red).*

LC-MS of crude

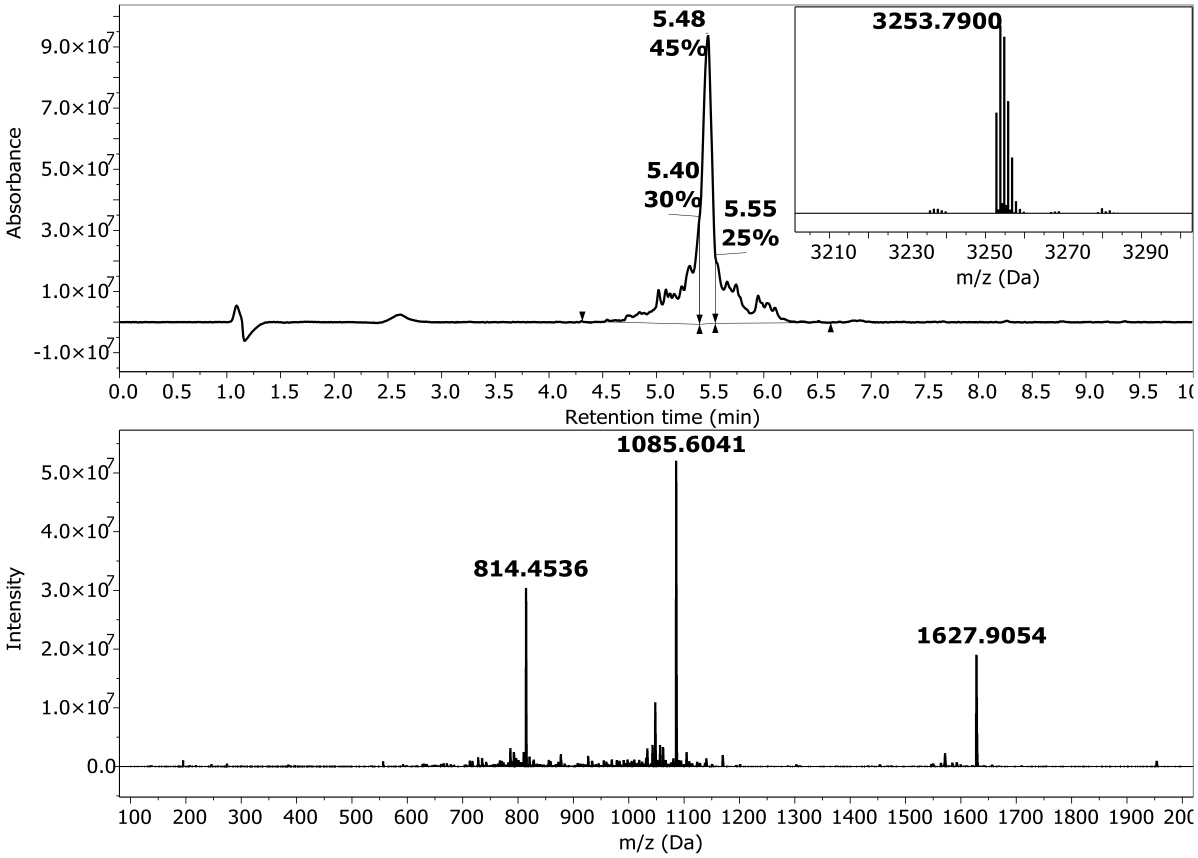

SI Figure 94*: LCMS Profile of crude Top: Absorbance chromatogram of GHRH Rt 5.48 min, 26% purity Bottom: ESI-TOF spectrum found within Rt 2–8 min (insert: deconvoluted masses). Monoisotopic mass (ESI+) calcd. for* $C_{147}H_{240}N_{40}O_{43}$ *3253.7823, found 3253.7900.*

UHPLC

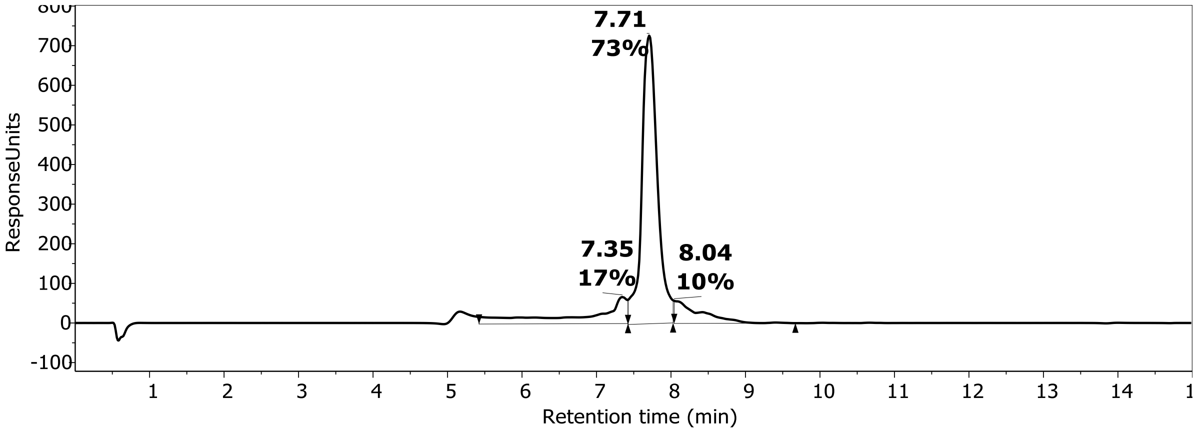

***SI Figure 95**. UHPLC profile of crude Rt 7.71 min, 73% purity based on Area Under Curve (AUC) at λ = 214 nm.*

6.7.2 Shuffling 1

H_2N- ARSLFYNIQG RLKDAITKLY AQLSALDVS-$CONH_2$

The peptide shuffling 1 of GHRH was synthesized on commercially available Novabiochem® NovaPEG Rink Amide resin (0.41 mmol/g, 101.7 mg, 42 μmol) using the standard AFPS protocol. (**SI Figure 96**) Total synthesis time to afford resin-bound peptide was approximately 1.5 h. Cleavage of the peptidyl-resin (18.3 mg, approx. 7.5 μmol) afforded the crude peptide as a colorless solid (5.5 mg, 22% purity by LCMS [**SI Figure 97**], 35% purity by UHPLC [**SI Figure 98**]).

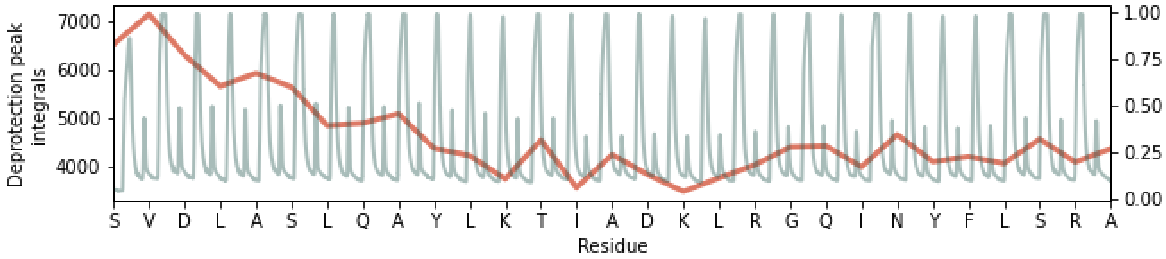

***SI Figure 96**. UV trace (λ = 310 nm) from AFPS of shuffling 1 of GHRH (green) and deprotection peak integrals (red).*

LC-MS of crude

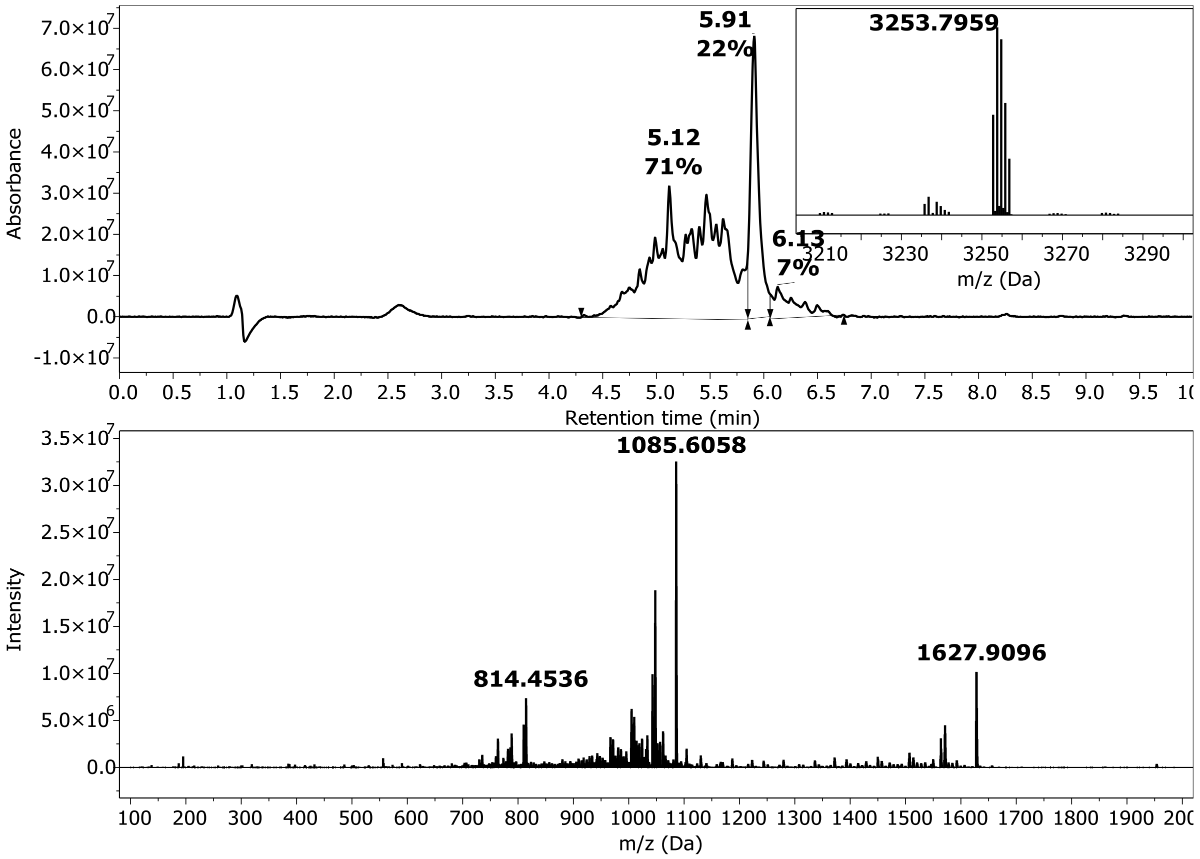

SI Figure 97*. LCMS Profile of crude Top: Absorbance chromatogram of shuffling 1 of GHRH Rt 5.91 min, 22% purity Bottom: ESI-TOF spectrum found within Rt 2–8 min (insert: deconvoluted masses). Monoisotopic mass (ESI+) calcd. for* $C_{147}H_{240}N_{40}O_{43}$ *3253.7823, found 3253.7959.*

UHPLC

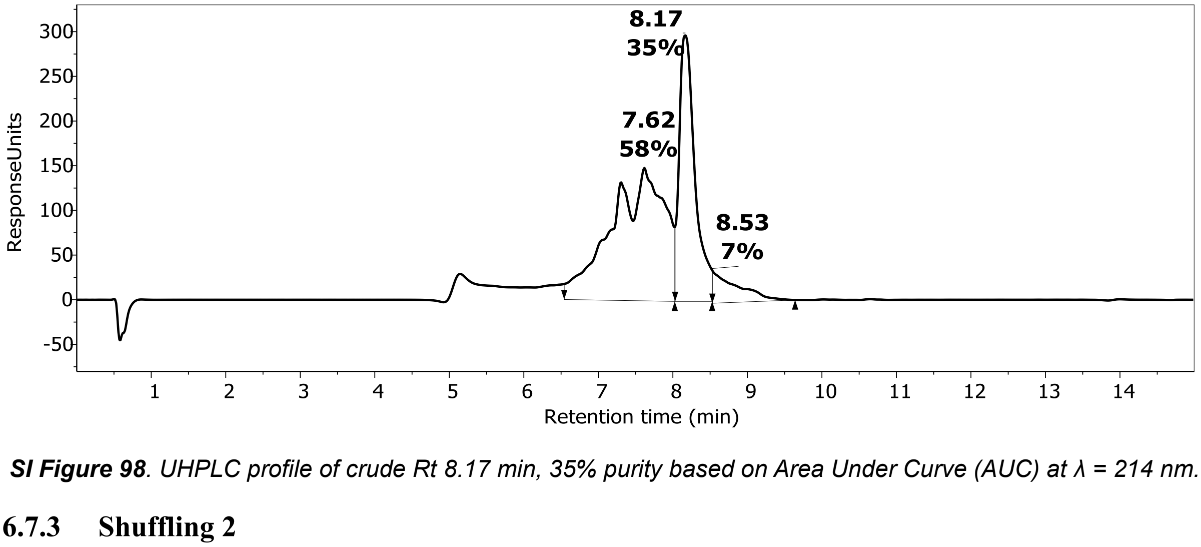

SI Figure 98*. UHPLC profile of crude Rt 8.17 min, 35% purity based on Area Under Curve (AUC) at λ = 214 nm.*

6.7.3 Shuffling 2

H_2N- LLKRQAFANK YSSLYRLTVL GQASDDIAI-$CONH_2$

The peptide shuffling 2 of GHRH was synthesized on commercially available Novabiochem® NovaPEG Rink Amide resin (0.41 mmol/g, 100.8 mg, 41 µmol) using the standard AFPS protocol. (**SI Figure 99**) Total synthesis time to afford resin-bound peptide was approximately 1.5 h. Cleavage of the

peptidyl-resin (16.8 mg, approx. 6.9 µmol) afforded the crude peptide as a colorless solid (5.9 mg, 23% purity by LCMS [**SI Figure 100**], 52% purity by UHPLC [**SI Figure 101**]).

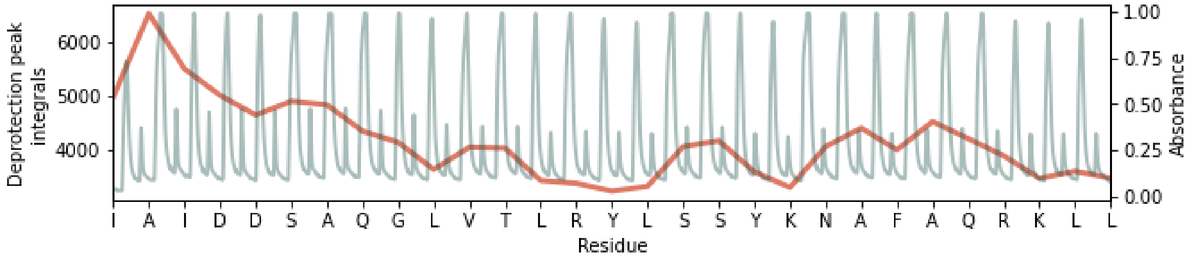

SI Figure 99. *UV trace (λ = 310 nm) from AFPS of shuffling 2 of GHRH (green) and deprotection peak integrals (red).*

LC-MS of crude

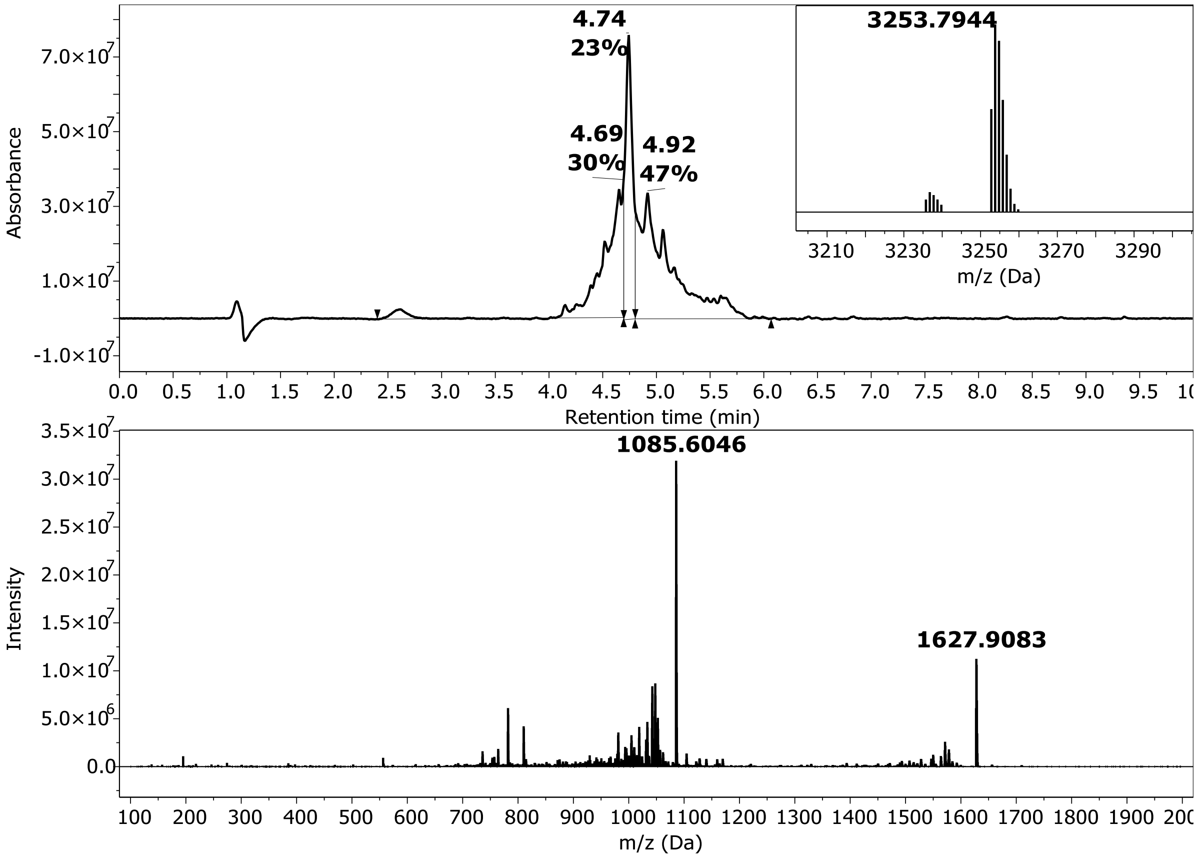

SI Figure 100. *LCMS Profile of crude Top: Absorbance chromatogram of shuffling 2 of GHRH Rt 4.74 min, 23% purity Bottom: ESI-TOF spectrum found within Rt 2–8 min (insert: deconvoluted masses). Monoisotopic mass (ESI+) calcd. for $C_{147}H_{240}N_{40}O_{43}$ 3253.7823, found 3253.7944.*

UHPLC

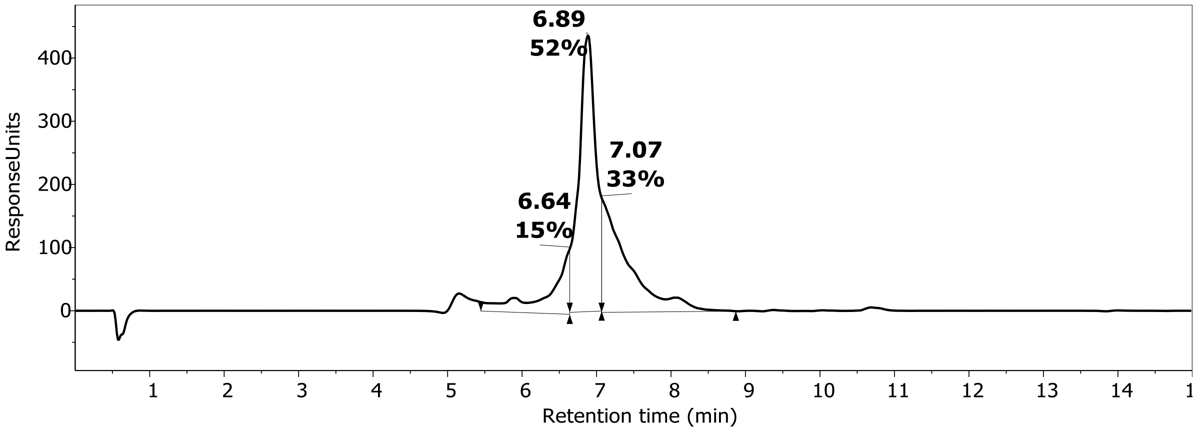

***SI Figure 101**. UHPLC profile of crude Rt 6.89 min, 52% purity based on Area Under Curve (AUC) at λ = 214 nm.*

6.7.4 Shuffling 3

H_2N- AKSQYDINGL RQVARLLLTS YLFKIDAAS-$CONH_2$

The peptide shuffling 3 of GHRH was synthesized on commercially available Novabiochem® NovaPEG Rink Amide resin (0.41 mmol/g, 100.6 mg, 41 µmol) using the standard AFPS protocol. (**SI Figure 102**) Total synthesis time to afford resin-bound peptide was approximately 1.5 h. Cleavage of the peptidyl-resin (17.7 mg, approx. 7.3 µmol) afforded the crude peptide as a colorless solid (5.5 mg, 18% purity by LCMS [**SI Figure 103**], 28% purity by UHPLC [**SI Figure 104**]).

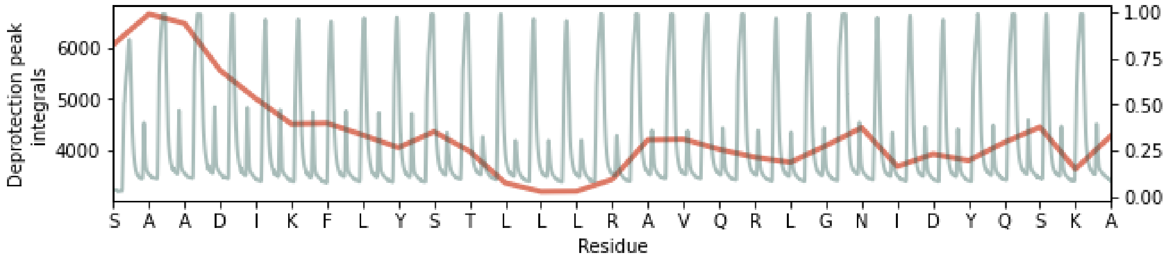

***SI Figure 102**. UV trace (λ = 310 nm) from AFPS of shuffling 3 of GHRH (green) and deprotection peak integrals (red).*

LC-MS of crude

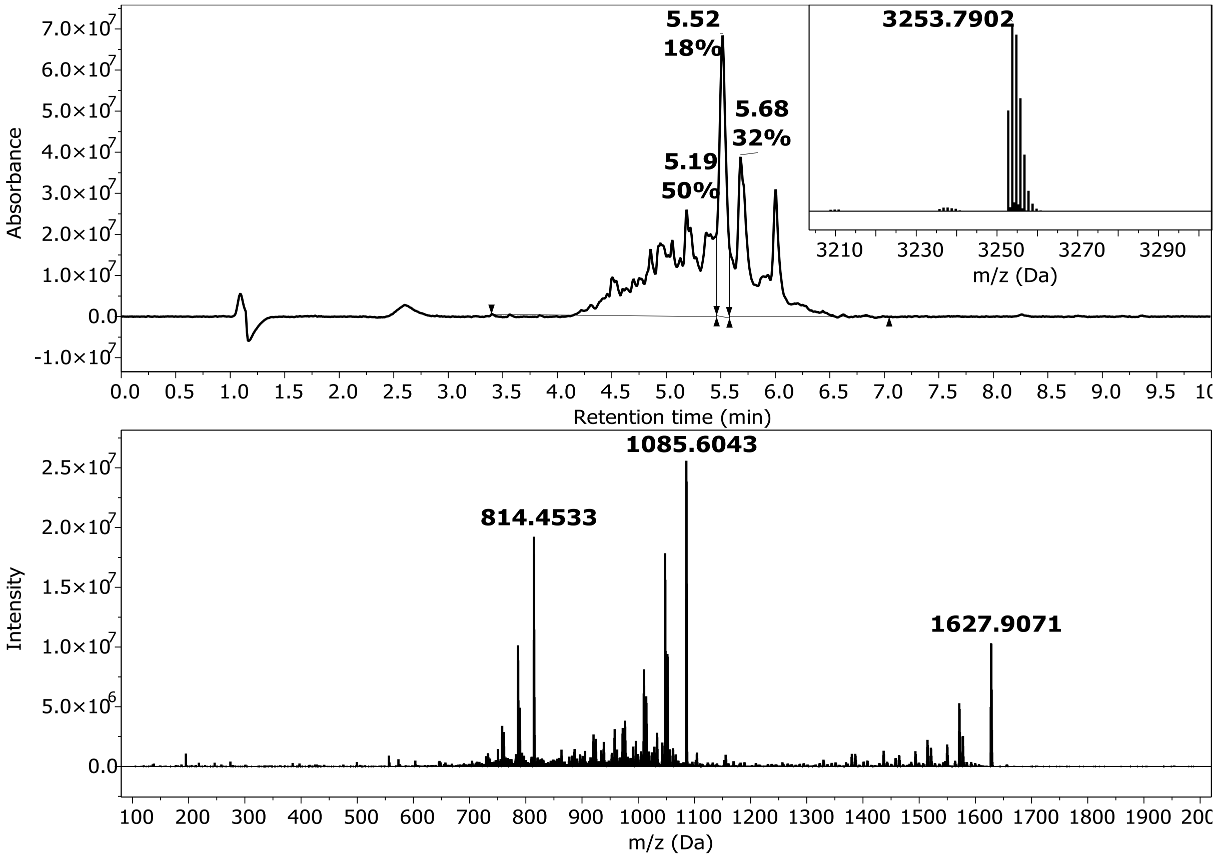

***SI Figure 103**. LCMS Profile of crude Top: Absorbance chromatogram of shuffling 3 of GHRH Rt 5.52 min, 18% purity Bottom: ESI-TOF spectrum found within Rt 2–8 min (insert: deconvoluted masses). Monoisotopic mass (ESI+) calcd. for $C_{147}H_{240}N_{40}O_{43}$ 3253.7823, found 3253.7902.*

UHPLC

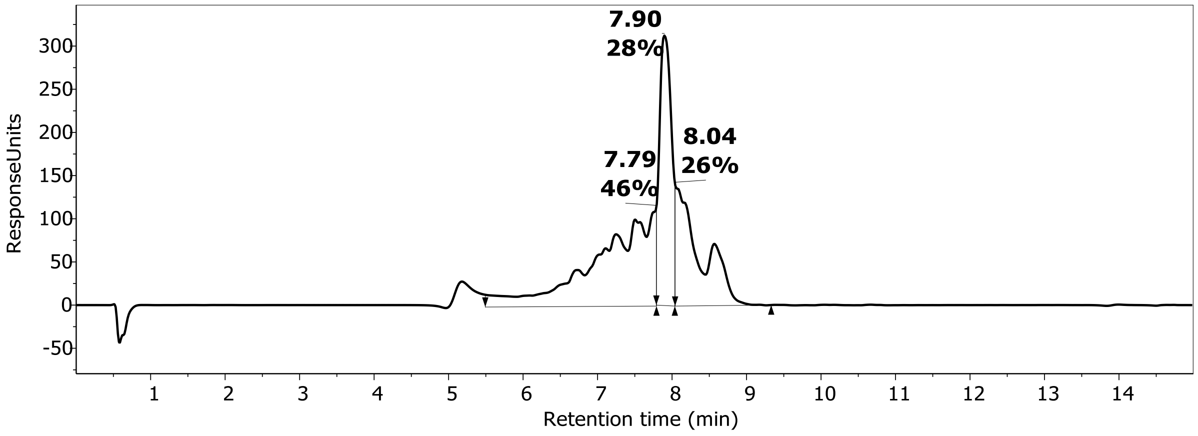

***SI Figure 104**. UHPLC profile of crude Rt 7.90 min, 28% purity based on Area Under Curve (AUC) at λ = 214 nm.*

6.7.5 Shuffling 4

H_2N- ILYGRFAQTV LLLDAASLYD SKQRKISAN-$CONH_2$

The peptide shuffling 4 of GHRH was synthesized on commercially available Novabiochem® NovaPEG Rink Amide resin (0.41 mmol/g, 100.1 mg, 41 μmol) using the standard AFPS protocol. (**SI Figure 105**) Total synthesis time to afford resin-bound peptide was approximately 1.5 h. Cleavage of

the peptidyl-resin (16.1 mg, approx. 6.6 µmol) afforded the crude peptide as a colorless solid (5.2 mg, 29% purity by LCMS [**SI Figure 106**], 49% purity by UHPLC [**SI Figure 107**]).

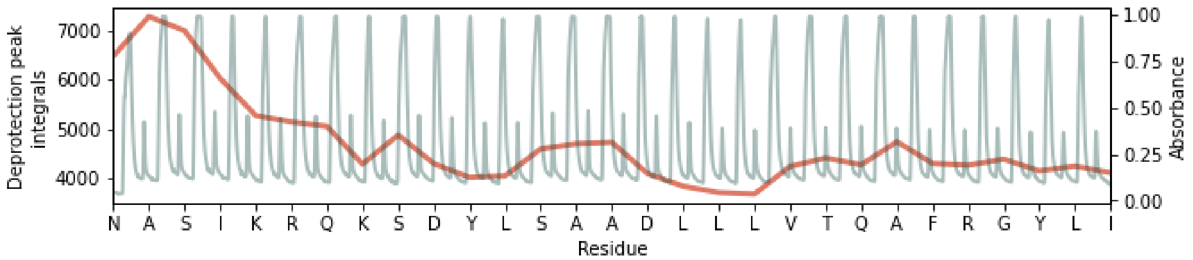

***SI Figure 105**. UV trace (λ = 310 nm) from AFPS of shuffling 4 of GHRH (green) and deprotection peak integrals (red).*

LC-MS of crude

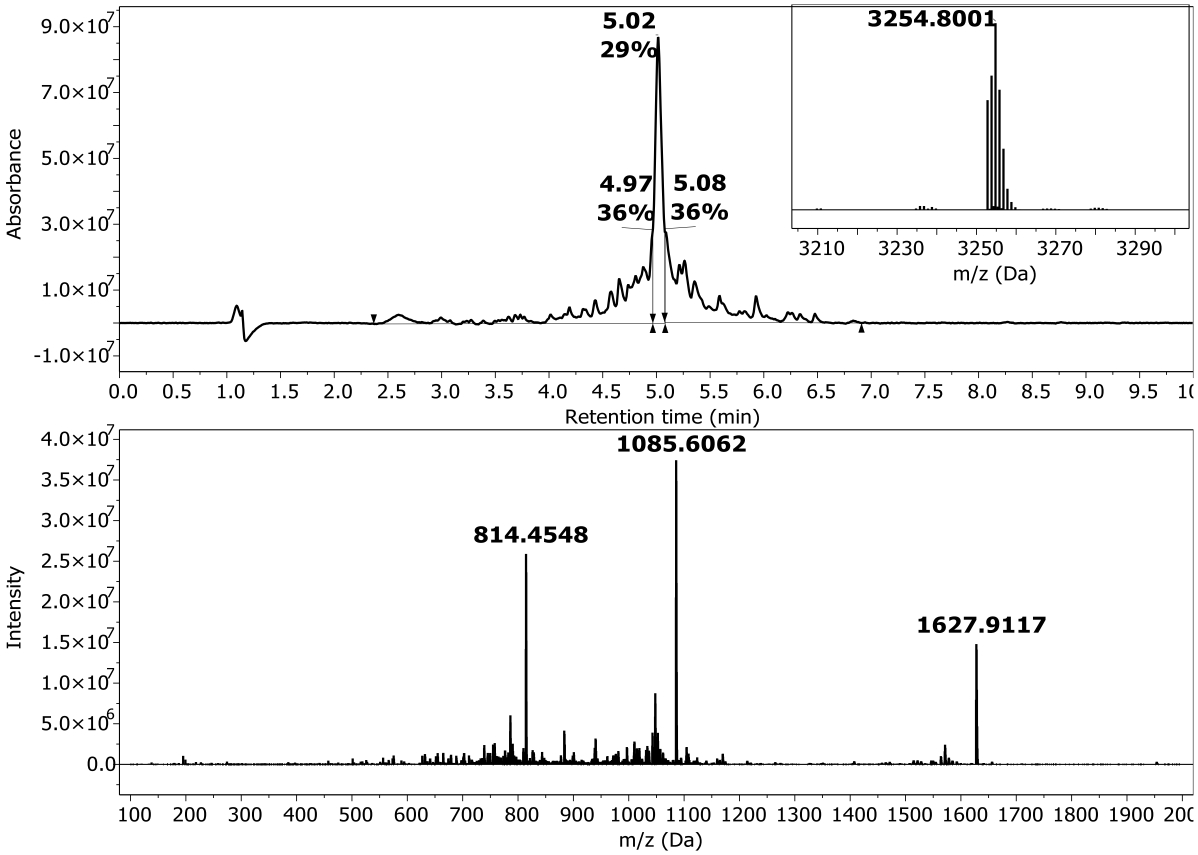

***SI Figure 106**. LCMS Profile of crude Top: Absorbance chromatogram of shuffling 4 of GHRH Rt 5.02 min, 29% purity Bottom: ESI-TOF spectrum found within Rt 2–8 min (insert: deconvoluted masses). Monoisotopic mass (ESI+) calcd. for $C_{147}H_{240}N_{40}O_{43}$ 3253.7823, found 3253.8002.*

UHPLC

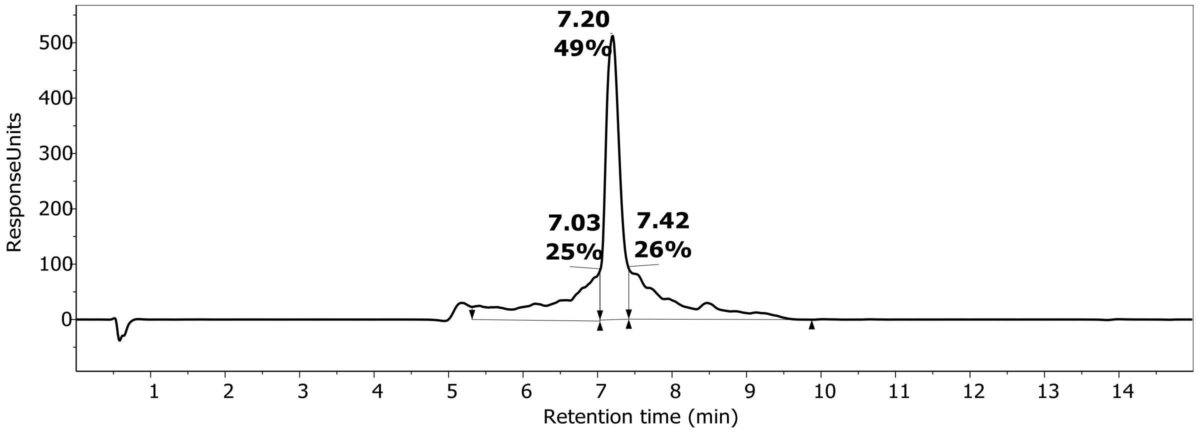

SI Figure 107*. UHPLC profile of crude Rt 7.20 min, 49% purity based on Area Under Curve (AUC) at λ = 214 nm.*

6.7.6 Shuffling 5

H_2N- TGKALRARIK LFLSLASSDY LDIQVQAYN-$CONH_2$

The peptide shuffling 5 of GHRH was synthesized on commercially available Novabiochem® NovaPEG Rink Amide resin (0.41 mmol/g, 100.8 mg, 41 µmol) using the standard AFPS protocol. (**SI Figure 108**) Total synthesis time to afford resin-bound peptide was approximately 1.5 h. Cleavage of the peptidyl-resin (17.7 mg, approx. 7.3 µmol) afforded the crude peptide as a colorless solid (5.9 mg, 32% purity by LCMS [**SI Figure 109**], 46% purity by UHPLC [**SI Figure 110**]).

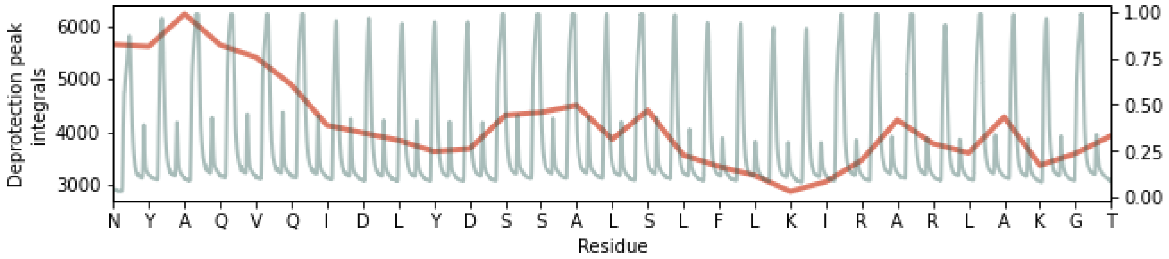

SI Figure 108*. UV trace (λ = 310 nm) from AFPS of shuffling 5 of GHRH (green) and deprotection peak integrals (red).*

LC-MS of crude

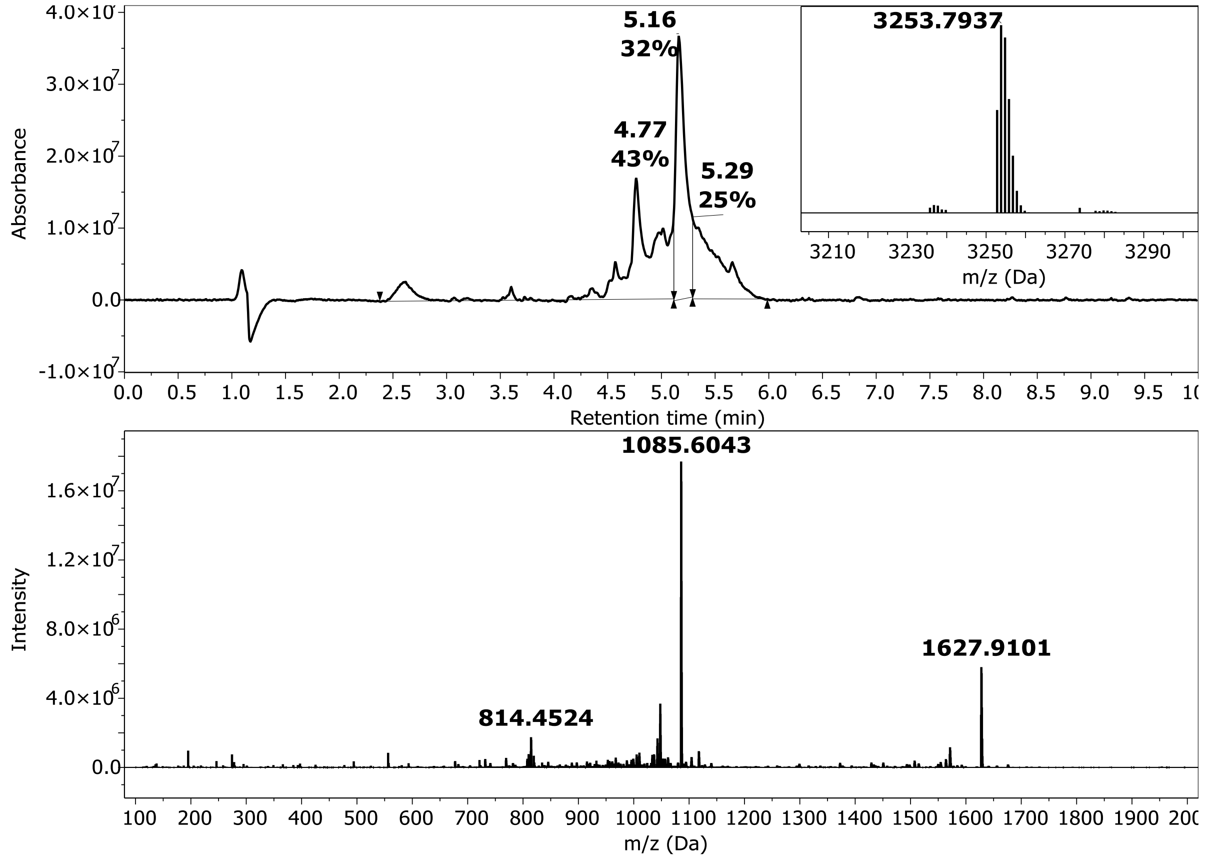

SI Figure 109. *LCMS Profile of crude Top: Absorbance chromatogram of shuffling 5 of GHRH Rt 5.16 min, 32% purity Bottom: ESI-TOF spectrum found within Rt 2–8 min (insert: deconvoluted masses). Monoisotopic mass (ESI+) calcd. for* $C_{147}H_{240}N_{40}O_{43}$ *3253.7823, found 3253.7937.*

UHPLC

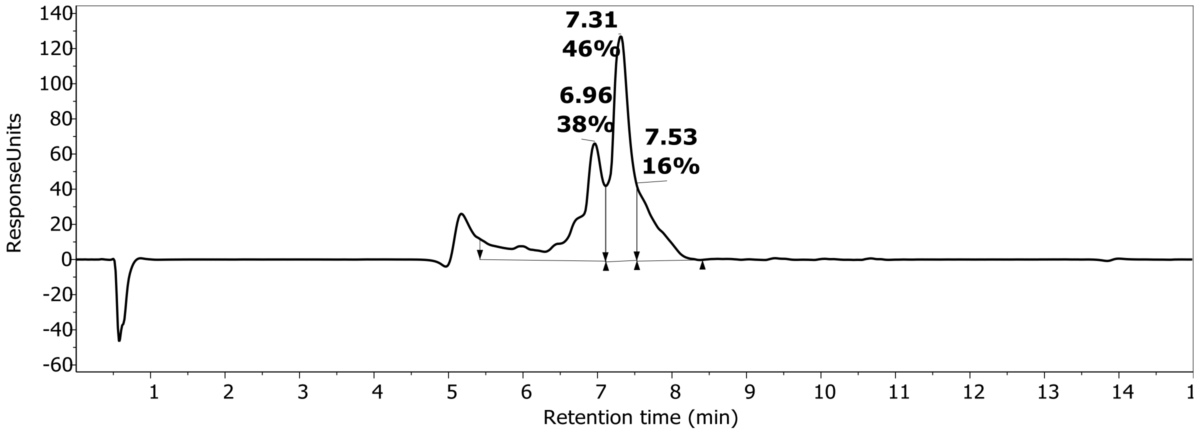

SI Figure 110. *UHPLC profile of crude Rt 7.31 min, 46% purity based on Area Under Curve (AUC) at λ = 214 nm.*

6.8 MYC[421–439] shuffling

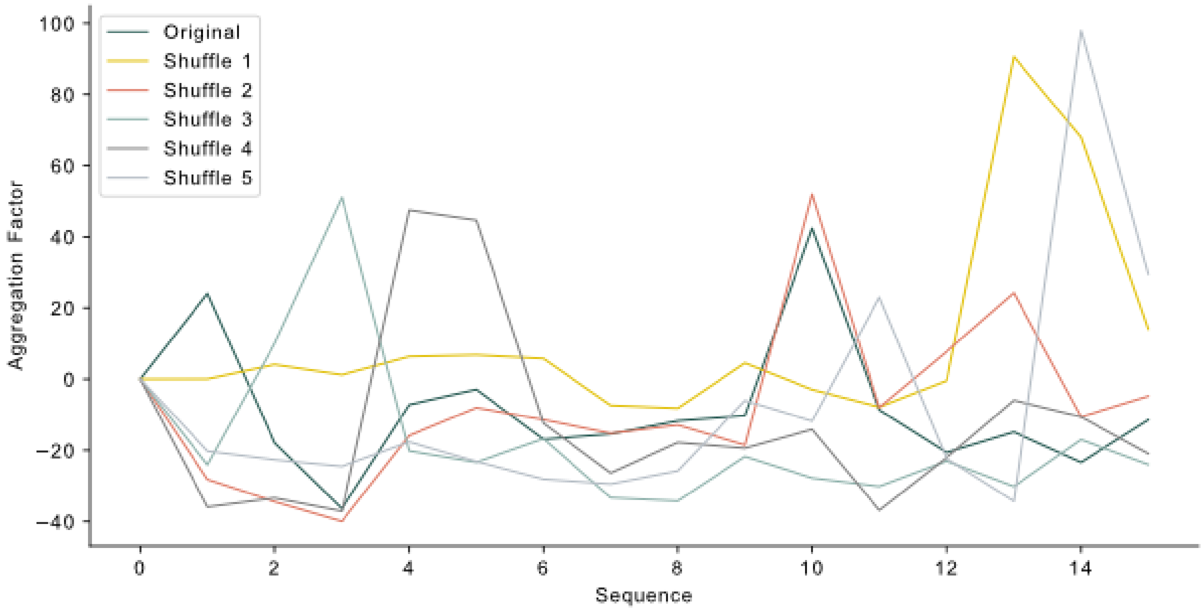

SI Figure 111: *Plot of aggregation factor values for the different shuffled MYC [421–439] fragments*

6.8.1 Original sequence

H_2N- REQLKHKLEQ LRNSCA-$CONH_2$

The peptide MYC[421–439] was synthesized on commercially available Novabiochem® NovaPEG Rink Amide resin (0.41 mmol/g, 100.3 mg, 41 µmol) using the standard AFPS protocol. (**SI Figure 112**) Total synthesis time to afford resin-bound peptide was approximately 0.8 h. Cleavage of the peptidyl-resin (19.1 mg, approx. 7.8 µmol) afforded the crude peptide as a colorless solid (3.4 mg, 47% purity by LCMS [**SI Figure 113**], 83% purity by UHPLC [**SI Figure 114**]).

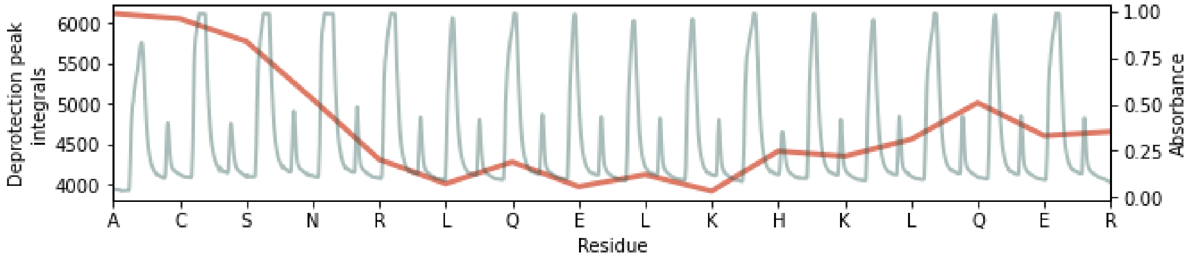

SI Figure 112*. UV trace (λ = 310 nm) from AFPS of MYC[421–439] (green) and deprotection peak integrals (red).*

LC-MS of crude

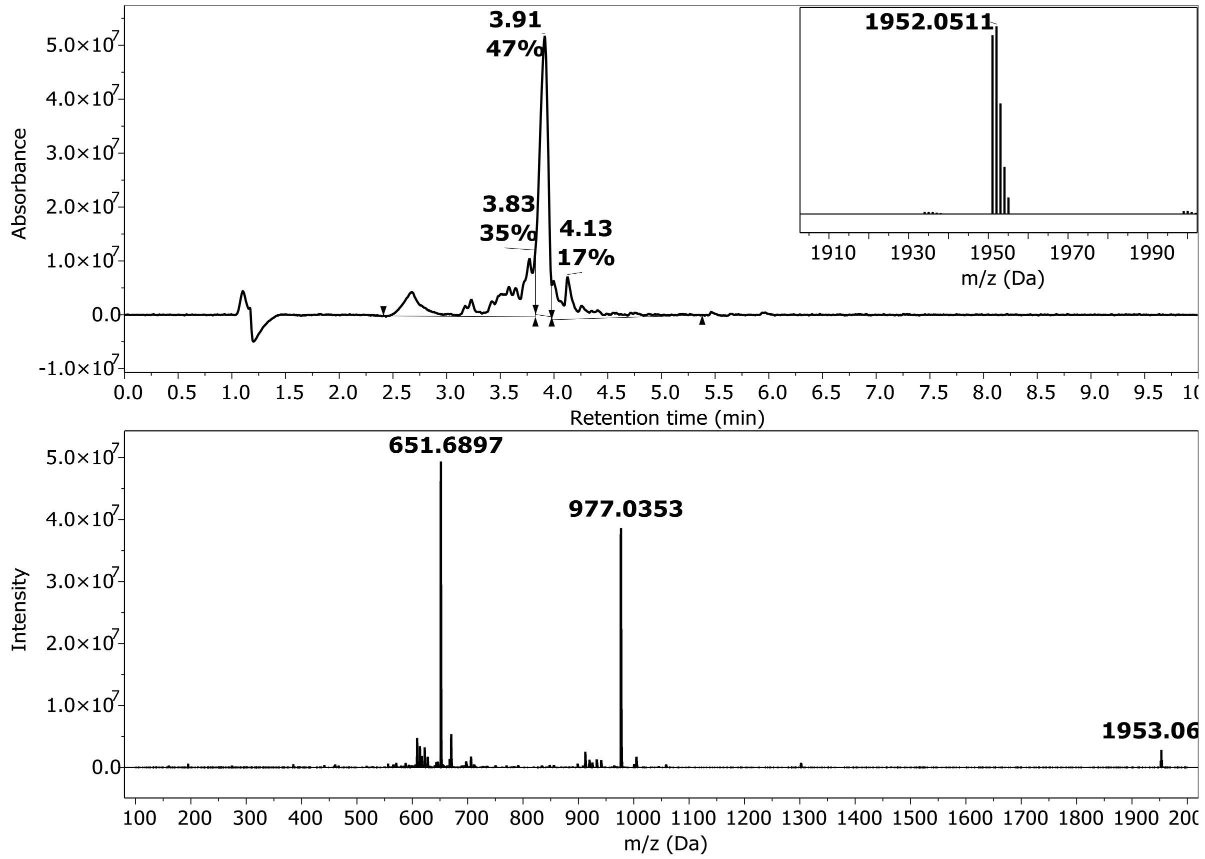

***SI Figure 113**. LCMS Profile of crude Top: Absorbance chromatogram of MYC[421–439] Rt 3.91 min, 47% purity Bottom: ESI-TOF spectrum found within Rt 2–8 min (insert: deconvoluted masses). Monoisotopic mass (ESI+) calcd. for $C_{81}H_{142}N_{30}O_{24}S$ 1951.0534, found 1951.0495.*

UHPLC

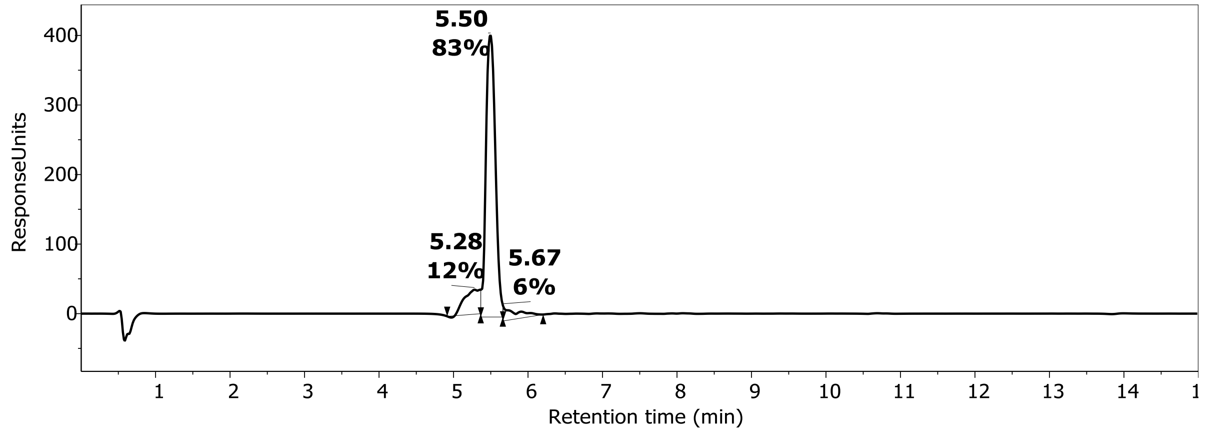

***SI Figure 114**. UHPLC profile of crude Rt 5.50 min, 83% purity based on Area Under Curve (AUC) at λ = 214 nm.*

6.8.2 Shuffling 1

H_2N- LCHEQQLRSL EKRKAN-$CONH_2$

The peptide shuffling 1 of MYC[421–439] was synthesized on commercially available Novabiochem® NovaPEG Rink Amide resin (0.41 mmol/g, 100.9 mg, 41 µmol) using the standard AFPS protocol(**SI Figure 115**) Total synthesis time to afford resin-bound peptide was approximately 0.8 h. Cleavage of

the peptidyl-resin (20.1 mg, approx. 8.2 μmol) afforded the crude peptide as a colorless solid (4.1 mg, 48% purity by LCMS [**SI Figure 116**], 86% purity by UHPLC [**SI Figure 117**]).

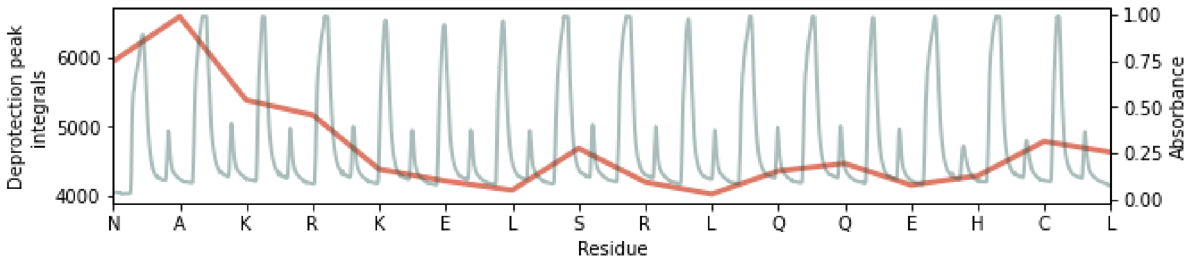

SI Figure 115*. UV trace (λ = 310 nm) from AFPS of shuffling 1 of MYC[421–439] (green) and deprotection peak integrals (red).*

LC-MS of crude

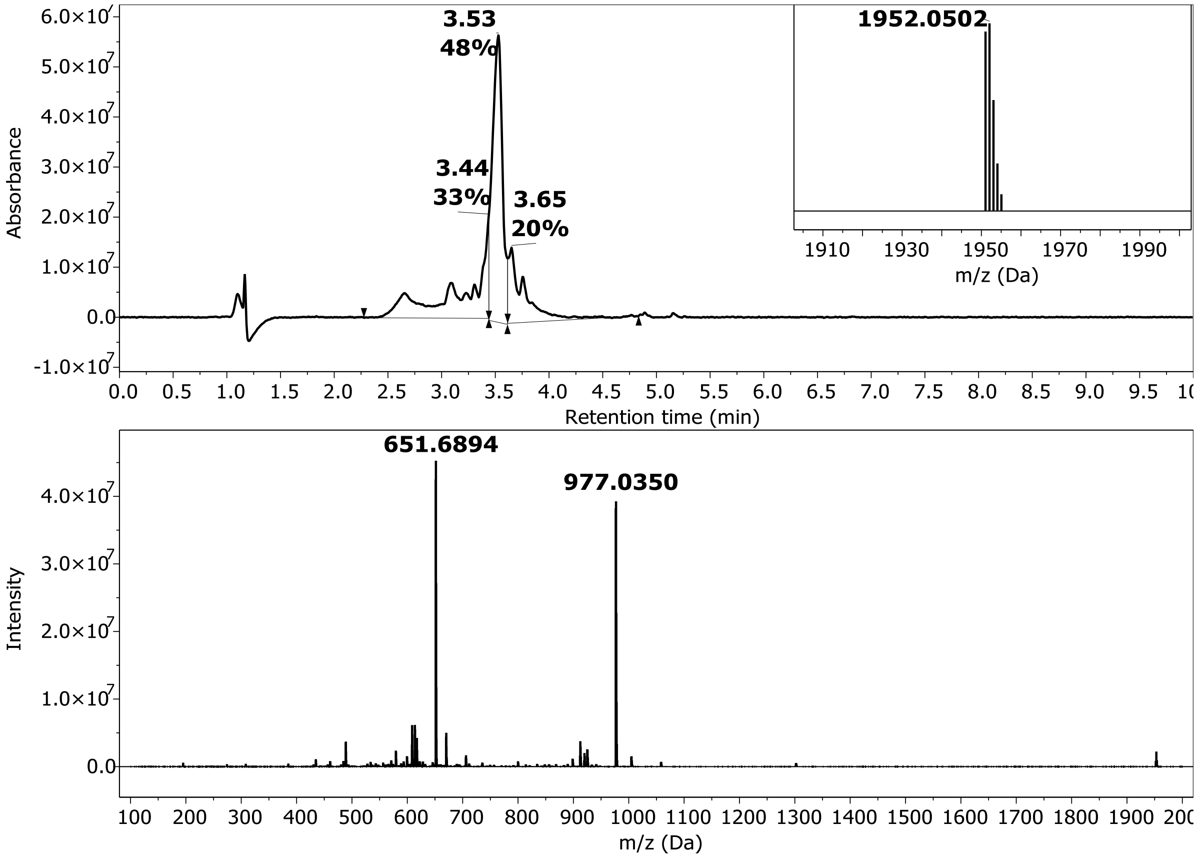

SI Figure 116*. LCMS Profile of crude Top: Absorbance chromatogram of shuffling 1 of MYC[421–439] Rt 3.53 min, 48% purity Bottom: ESI-TOF spectrum found within Rt 2–8 min (insert: deconvoluted masses). Monoisotopic mass (ESI+) calcd. for $C_{81}H_{142}N_{30}O_{24}S$ 1951.0534, found 1951.0485.*

UHPLC

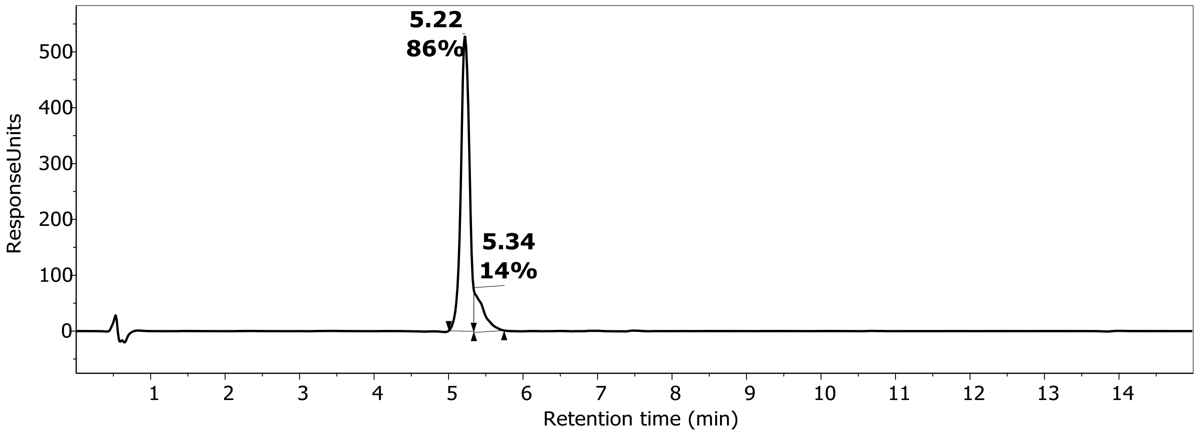

***SI Figure 117**. UHPLC profile of crude Rt 5.22 min, 86% purity based on Area Under Curve (AUC) at λ = 214 nm.*

6.8.3 Shuffling 2

H_2N- QLRCKHLELR AENSQK-$CONH_2$

The peptide shuffling 2 of MYC[421–439] was synthesized on commercially available Novabiochem® NovaPEG Rink Amide resin (0.41 mmol/g, 101.5 mg, 42 µmol) using the standard AFPS protocol. (**SI Figure 118**) Total synthesis time to afford resin-bound peptide was approximately 0.8 h. Cleavage of the peptidyl-resin (19.7 mg, approx. 8.1 µmol) afforded the crude peptide as a colorless solid (3.9 mg, 57% purity by LCMS [**SI Figure 119**], 84% purity by UHPLC [**SI Figure 120**]).

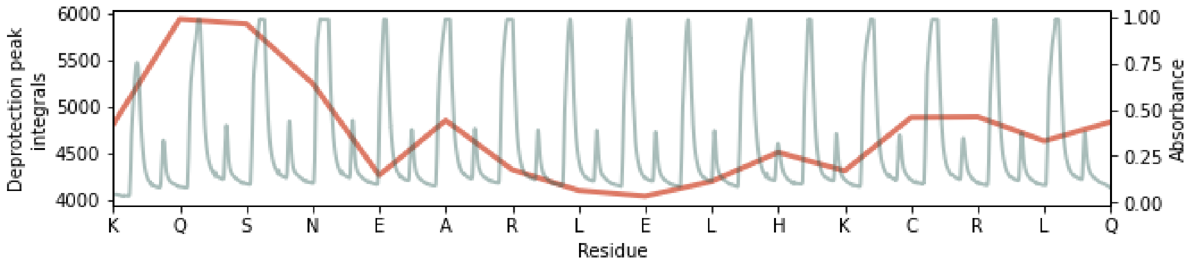

***SI Figure 118**. UV trace (λ = 310 nm) from AFPS of shuffling 2 of MYC[421–439] (green) and deprotection peak integrals (red).*

LC-MS of crude

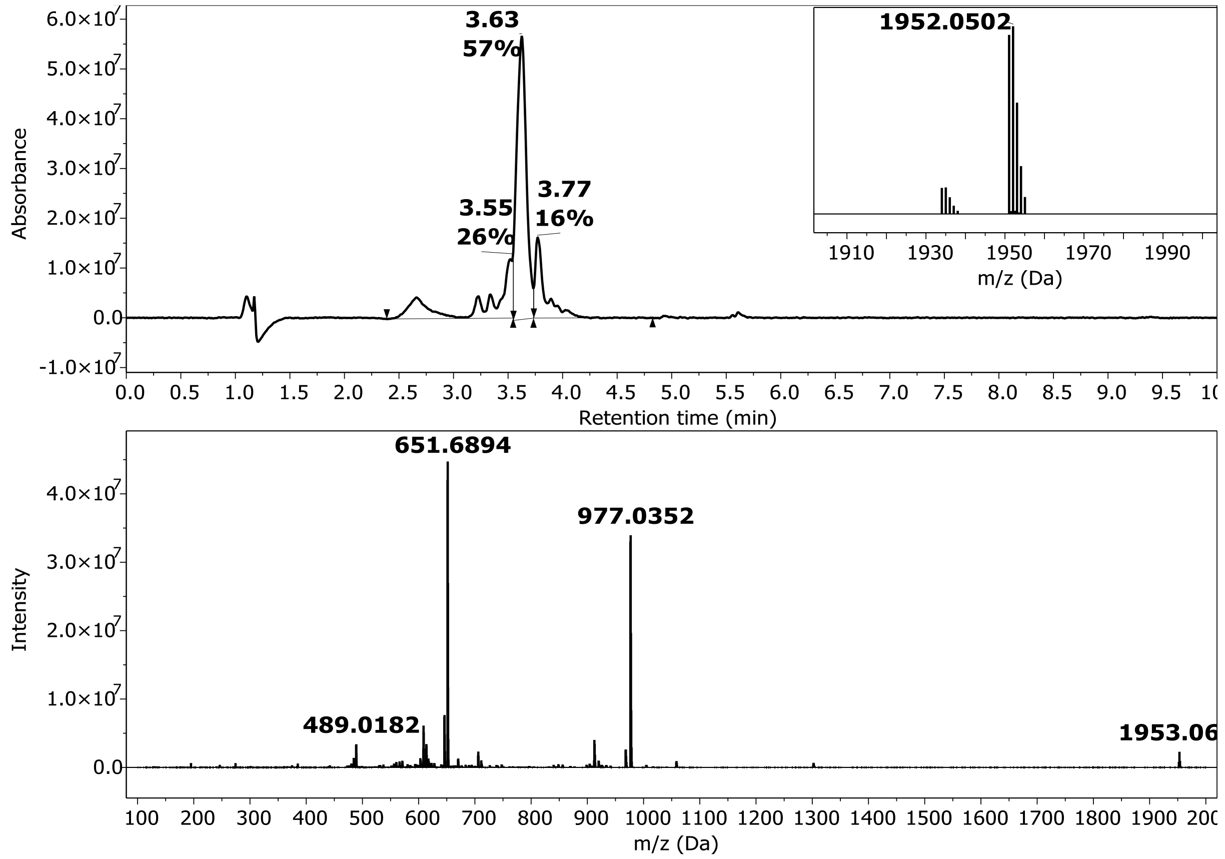

***SI Figure 119**. LCMS Profile of crude Top: Absorbance chromatogram of shuffling 2 of MYC[421–439] Rt 3.63 min, 57% purity Bottom: ESI-TOF spectrum found within Rt 2–8 min (insert: deconvoluted masses). Monoisotopic mass (ESI+) calcd. for for $C_{81}H_{142}N_{30}O_{24}S$ 1951.0534, found 1951.0485.*

UHPLC

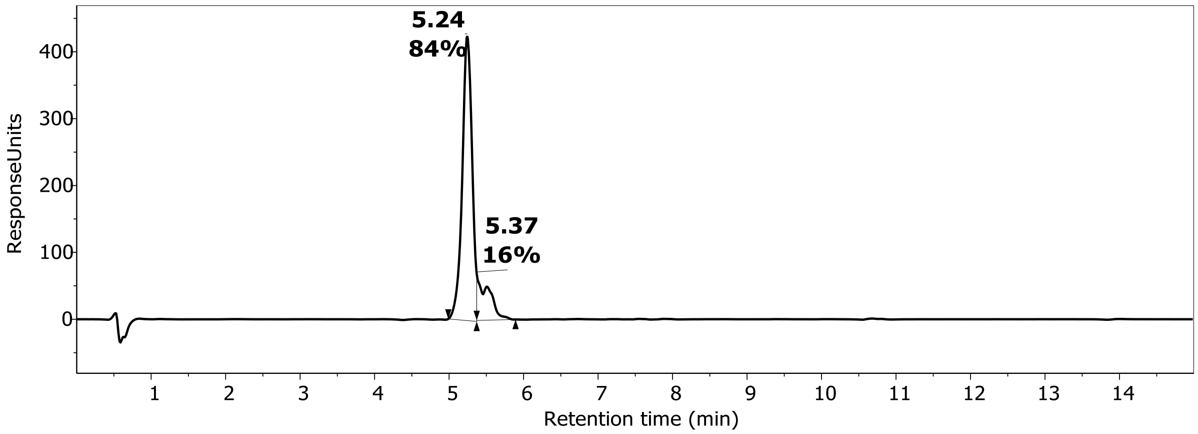

***SI Figure 120**. UHPLC profile of crude Rt 5.24 min, 84% purity based on Area Under Curve (AUC) at λ = 214 nm.*

6.8.4 Shuffling 3

H_2N- NRLSKRELQK LQHCAE-$CONH_2$

The peptide shuffling 3 of MYC [421–439] was synthesized on commercially available Novabiochem® NovaPEG Rink Amide resin (0.41 mmol/g, 100.6 mg, 41 µmol) using the standard AFPS protocol. (**SI Figure 121**) Total synthesis time to afford resin-bound peptide was approximately 0.8 h. Cleavage of

the peptidyl-resin (20.4 mg, approx. 8.4 µmol) afforded the crude peptide as a colorless solid (3.8 mg, 47% purity by LCMS [**SI Figure 122**], 75% purity by UHPLC [**SI Figure 123**]).

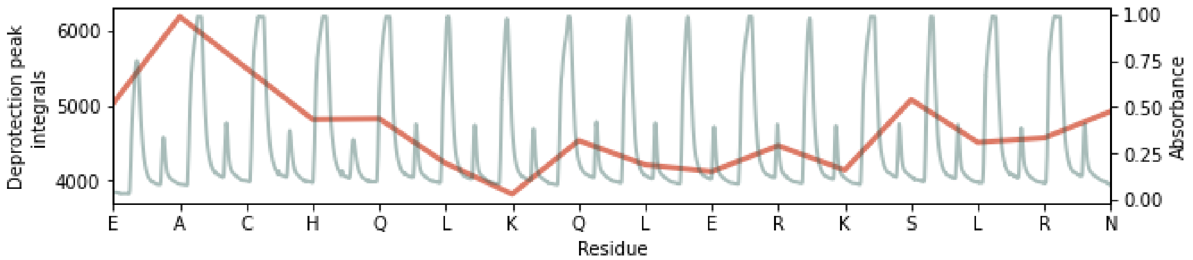

SI Figure 121. *UV trace (λ = 310 nm) from AFPS of shuffling 3 of MYC[421–439] (green) and deprotection peak integrals (red).*

LC-MS of crude

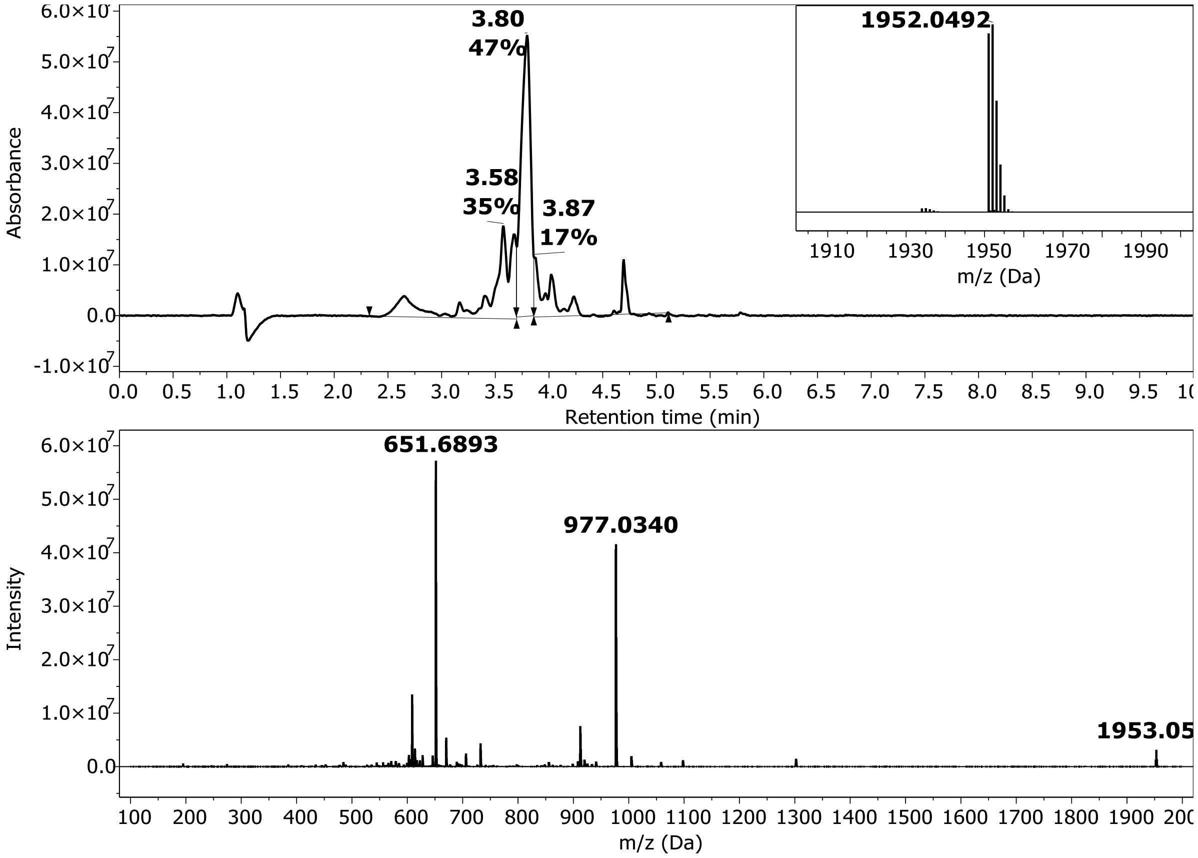

SI Figure 122. *LCMS Profile of crude Top: Absorbance chromatogram of shuffling 3 of MYC[421–439] Rt 3.80 min, 47% purity Bottom: ESI-TOF spectrum found within Rt 2–8 min (insert: deconvoluted masses). Monoisotopic mass (ESI+) calcd. for for* $C_{81}H_{142}N_{30}O_{24}S$ *1951.0534, found 1951.0481.*

UHPLC

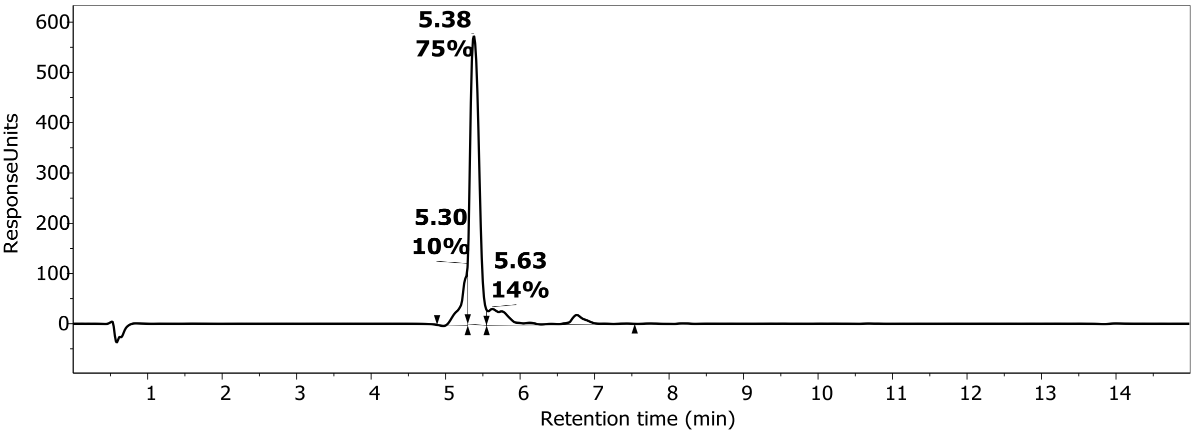

***SI Figure 123**. UHPLC profile of crude Rt 5.38 min, 75% purity based on Area Under Curve (AUC) at λ = 214 nm.*

6.8.5 Shuffling 4

H_2N- KRLSNLKREA CHQEQL-$CONH_2$

The peptide shuffling 4 of MYC[421–439] was synthesized on commercially available Novabiochem® NovaPEG Rink Amide resin (0.41 mmol/g, 101.1 mg, 41 µmol) using the standard AFPS protocol. (**SI Figure 124**) Total synthesis time to afford resin-bound peptide was approximately 0.8 h. Cleavage of the peptidyl-resin (20.6 mg, approx. 8.5 µmol) afforded the crude peptide as a colorless solid (3.5 mg, 53% purity by LCMS [**SI Figure 125**], 64% purity by UHPLC [**SI Figure 126**]).

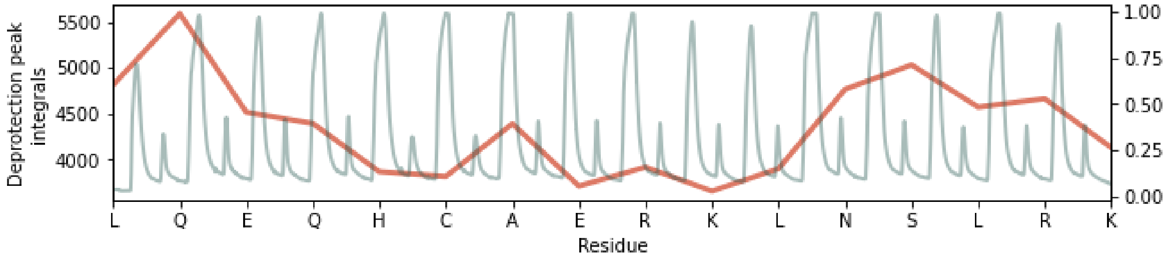

***SI Figure 124**. UV trace (λ = 310 nm) from AFPS of shuffling 4 of MYC[421–439] (green) and deprotection peak integrals (red).*

LC-MS of crude

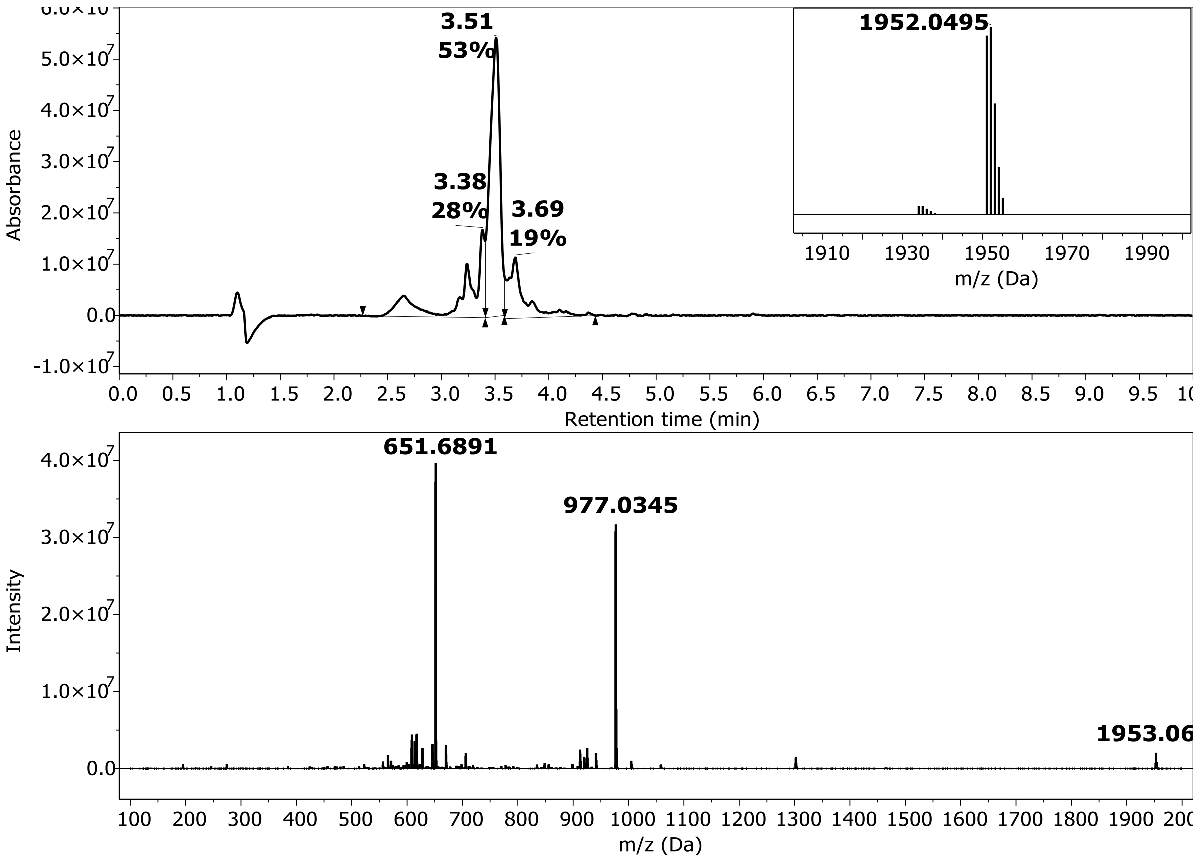

***SI Figure 125**. LCMS Profile of crude Top: Absorbance chromatogram of shuffling 4 of MYC[421–439] Rt 3.51 min, 53% purity Bottom: ESI-TOF spectrum found within Rt 2–8 min (insert: deconvoluted masses). Monoisotopic mass (ESI+) calcd. for for $C_{81}H_{142}N_{30}O_{24}S$ 1951.0534, found 1951.0480.*

UHPLC

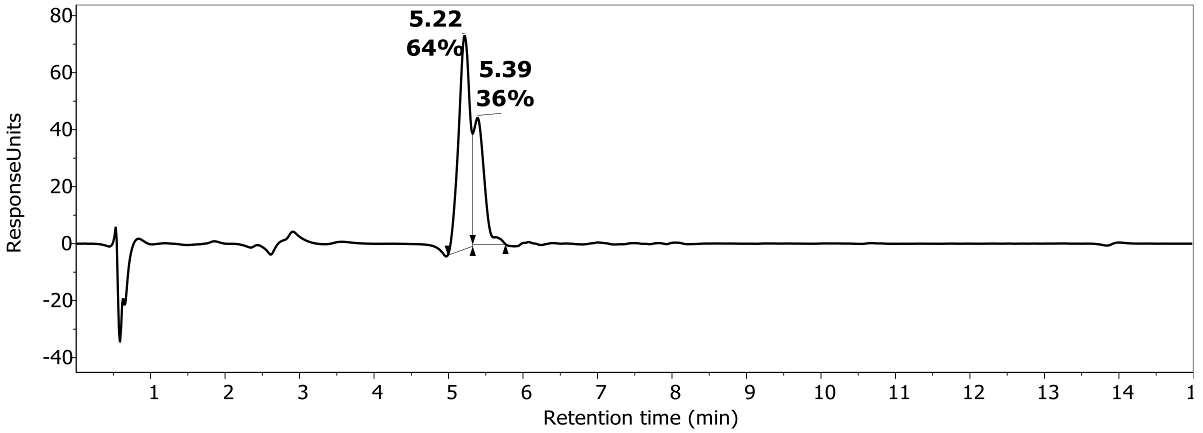

***SI Figure 126**. UHPLC profile of crude Rt 5.22 min, 64% purity based on Area Under Curve (AUC) at λ = 214 nm.*

6.8.6 Shuffling 5

H_2N- LHNQCLRESQ AKREKL-$CONH_2$

The peptide shuffling 5 of MYC[421–439] was synthesized on commercially available Novabiochem® NovaPEG Rink Amide resin (0.41 mmol/g, 100.0 mg, 41 μmol) using the standard AFPS protocol. (**SI Figure 127**) Total synthesis time to afford resin-bound peptide was approximately 0.8 h. Cleavage of the peptidyl-resin (20.4 mg, approx. 8.4 μmol) afforded the crude peptide as a colorless solid (4.2 mg, 51% purity by LCMS [**SI Figure 128**], 41% purity by UHPLC [**SI Figure 129**]).

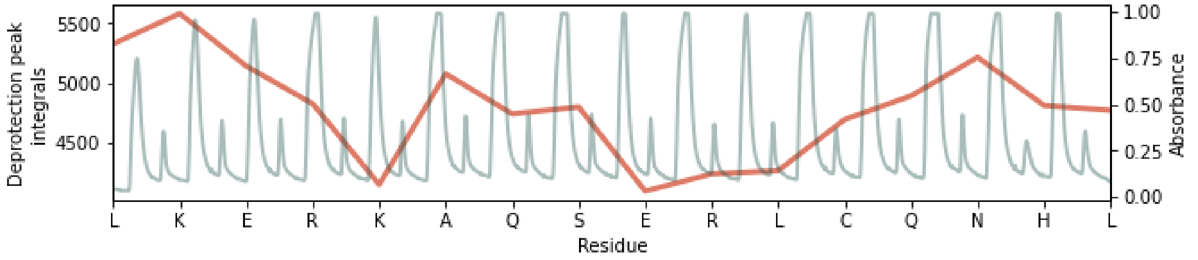

SI Figure 127. *UV trace (λ = 310 nm) from AFPS of shuffling 5 of MYC[421–439] (green) and deprotection peak integrals (red).*

LC-MS of crude

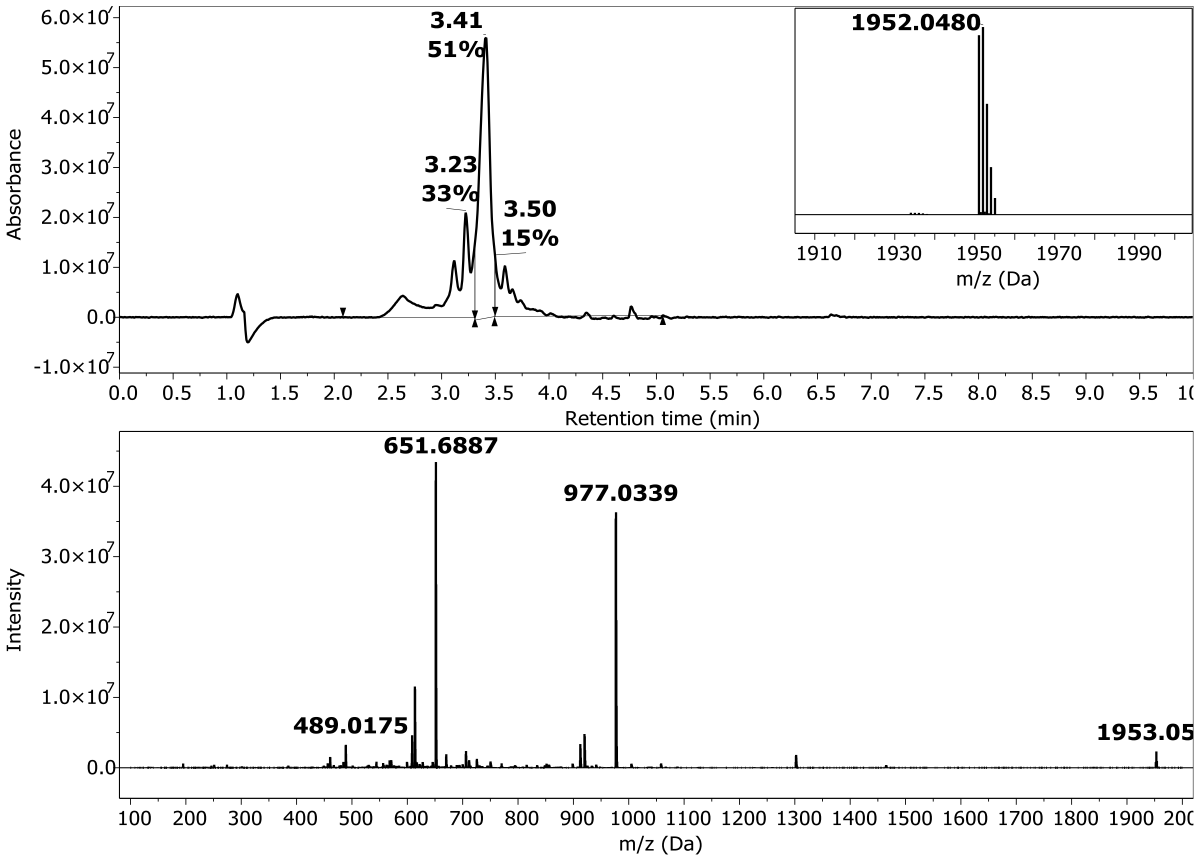

SI Figure 128. *LCMS Profile of crude Top: Absorbance chromatogram of shuffling 5 of MYC [421–439] Rt 3.41 min, 51% purity Bottom: ESI-TOF spectrum found within Rt 2–8 min (insert: deconvoluted masses). Monoisotopic mass (ESI+) calcd. for for* $C_{81}H_{142}N_{30}O_{24}S$ *1951.0534, found 1951.0464.*

UHPLC

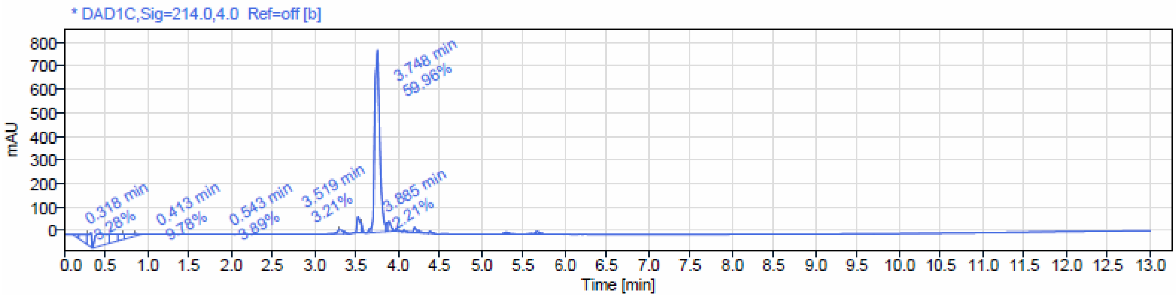

***SI Figure 129**. UHPLC profile of crude Rt 3.748 min, 60% purity based on Area Under Curve (AUC) at λ = 214 nm.*

This measurement was done the following way:

For determination of peptide masses by UHPLC the filtered peptide solution was diluted in 10–50% acetonitrile (MeCN) in water with 0.1% TFA (500 µL) to a final concentration of approximately 0.1 mM. The samples were analyzed on an Agilent 1290 Infinity II Series UHPLC, which is connected to an Agilent 1290 Infinity II Series DAD. Separation was carried out on an Agilent Zorbax 300 SB-C8 HPLC column (1.8 µm particle size, 2.1 × 100 mm) which was at 40 °C, with a sample injection volume of 5 µL. The elution was performed at a flow rate of 0.8 mL/min with solvent A: 95% H_2O + 0.1% trifluoroacetic acid with 5% MeCN and solvent B: 95% MeCN + 0.1% trifluoroacetic with 5% H_2O acid with the following UHPLC gradient: gradient: isocratic at 0% Solvent B for 1.5 min, followed by a linear gradient of 0–100% Solvent B over 10 min, followed by isocratic at 100% Solvent B for 1.5 min.

6.9 PCP-4[43–62] shuffling

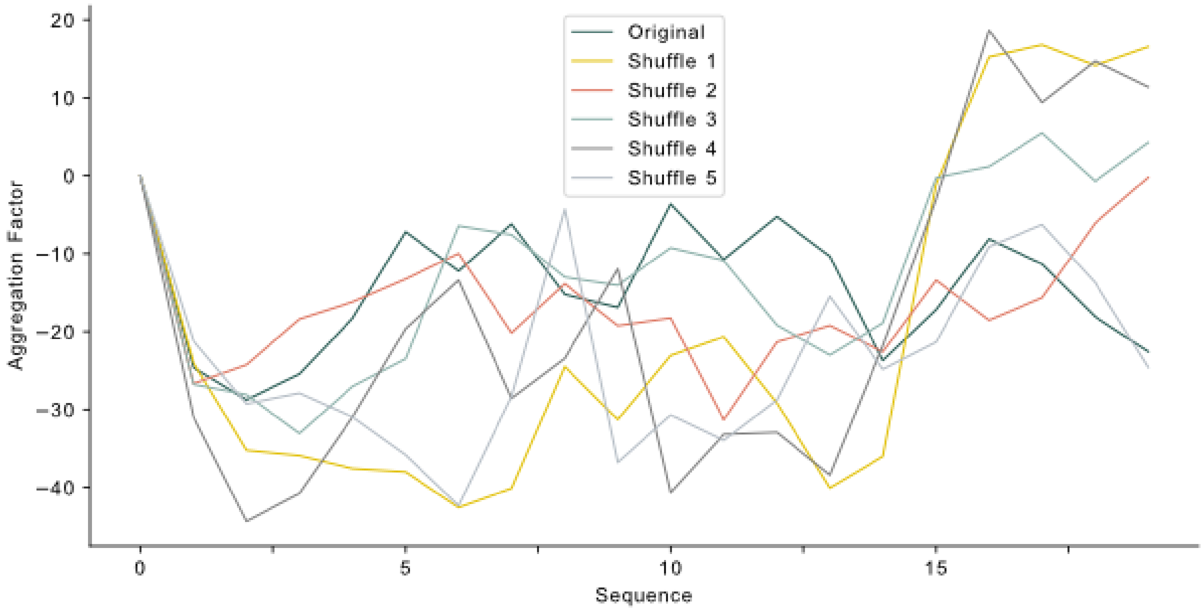

SI Figure 130. *Plot of aggregation factor values for the different shuffled PCP-4[43–62] fragments*

6.9.1 Original sequence

H_2N- AVAIQSQFRK FQKKKAGSQS-$CONH_2$

The peptide PCP-4[43–62] was synthesized on commercially available Novabiochem® NovaPEG Rink Amide resin (0.41 mmol/g, 101.1 mg, 41 μmol) using the standard AFPS protocol. (**SI Figure 131**) Total synthesis time to afford resin-bound peptide was approximately 1.0 h. Cleavage of the peptidyl-resin (17.2 mg, approx. 7.1 μmol) afforded the crude peptide as a colorless solid (7.2 mg, 47% purity by LCMS [**SI Figure 132**], 86% purity by UHPLC [**SI Figure 133**]).

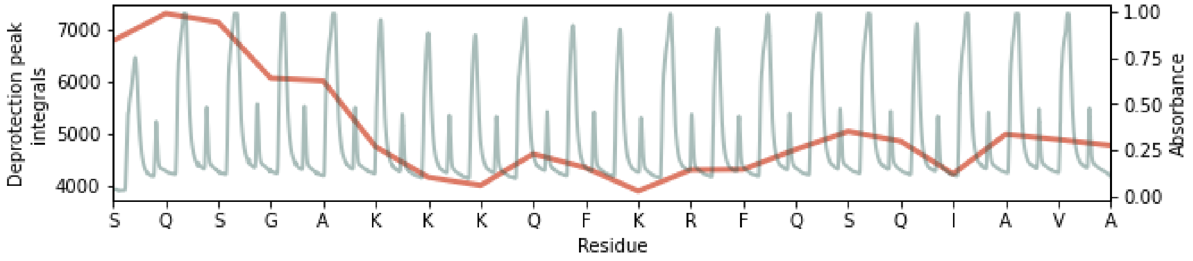

SI Figure 131. *UV trace (λ = 310 nm) from AFPS of PCP-4[43–62] (green) and deprotection peak integrals (red).*

LC-MS of crude

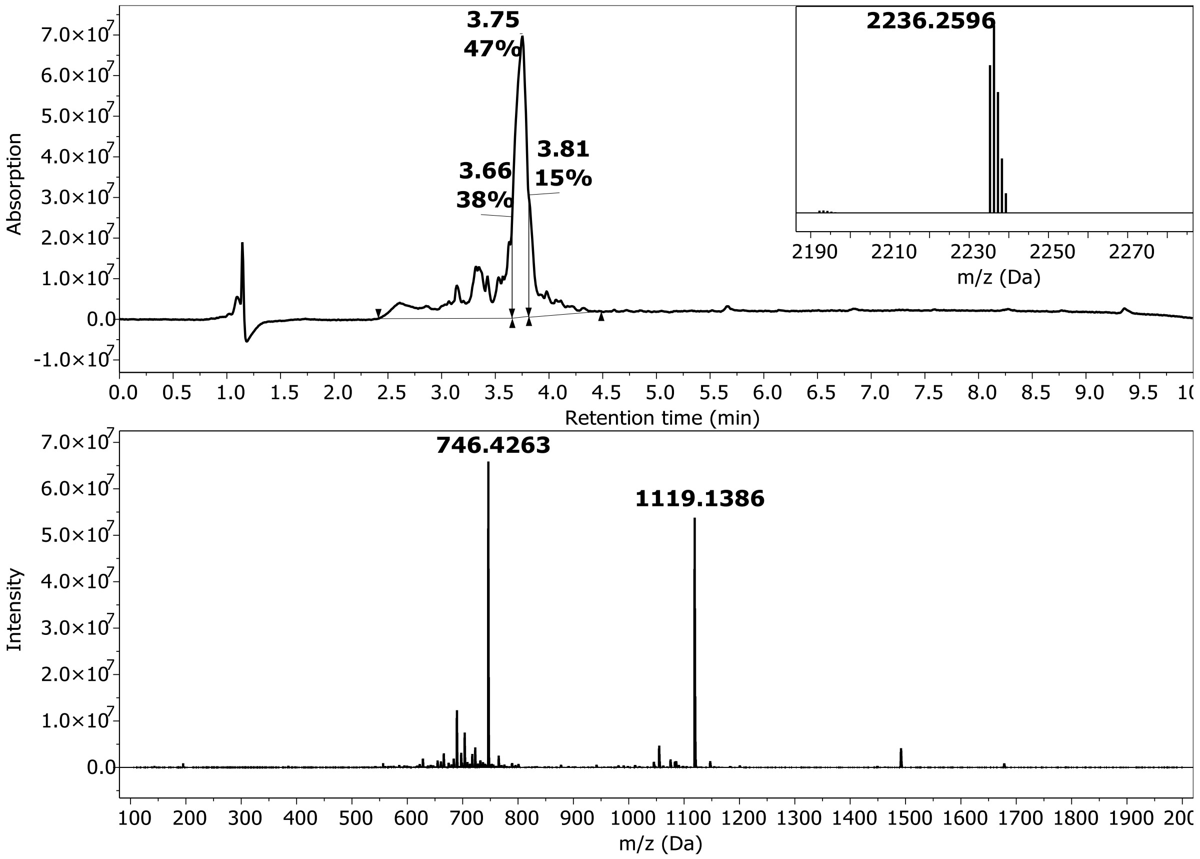

***SI Figure 132**. LCMS Profile of crude Top: Absorbance chromatogram of PCP-4[43–62] Rt 3.75 min, 47% purity Bottom: ESI-TOF spectrum found within Rt 2–8 min (insert: deconvoluted masses). Monoisotopic mass (ESI+) calcd. for $C_{99}H_{166}N_{32}O_{27}$ 2235.2600, found 2235.2573.*

UHPLC

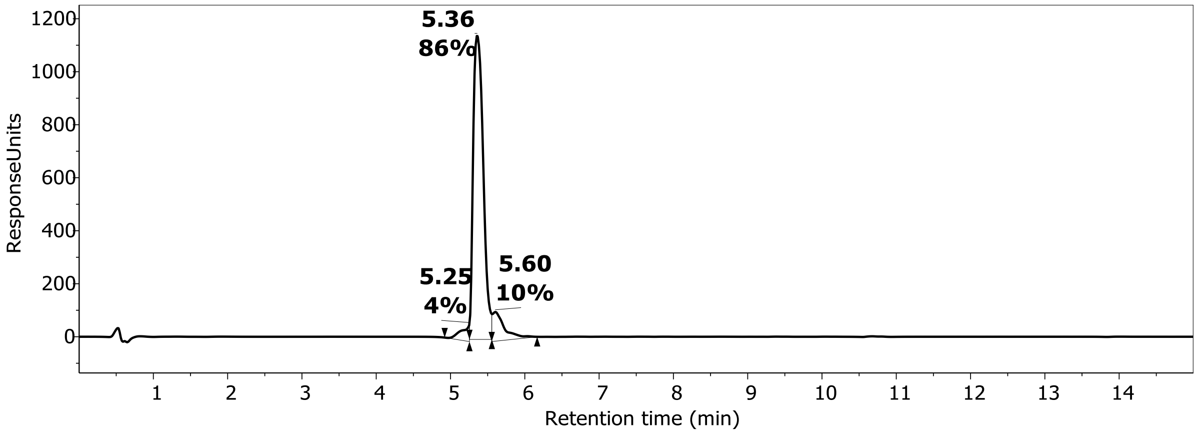

***SI Figure 133**: UHPLC profile of crude Rt 5.36 min, 86% purity based on Area Under Curve (AUC) at λ = 214 nm.*

6.9.2 Shuffling 1

H_2N- FQGKASQAIK SFQSQARVKK-$CONH_2$

The peptide shuffling 1 of PCP-4[43–62] was synthesized on commercially available Novabiochem® NovaPEG Rink Amide resin (0.41 mmol/g, 100.1 mg, 41 µmol) using the standard AFPS protocol(**SI Figure 134**) Total synthesis time to afford resin-bound peptide was approximately 1.0 h. Cleavage of

the peptidyl-resin (17.1 mg, approx. 7.0 µmol) afforded the crude peptide as a colorless solid (4.3 mg, 26% purity by LCMS [**SI Figure 135**], 55% purity by UHPLC [**SI Figure 136**]).

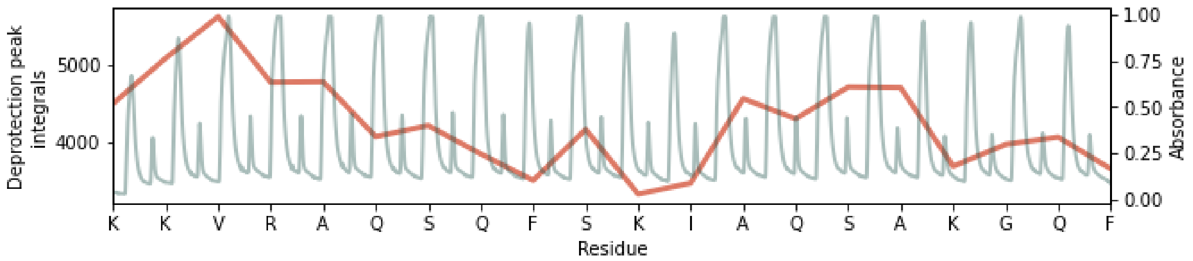

SI Figure 134. *UV trace (λ = 310 nm) from AFPS of shuffling 1 of PCP-4[43–62] (green) and deprotection peak integrals (red).*

LC-MS of crude

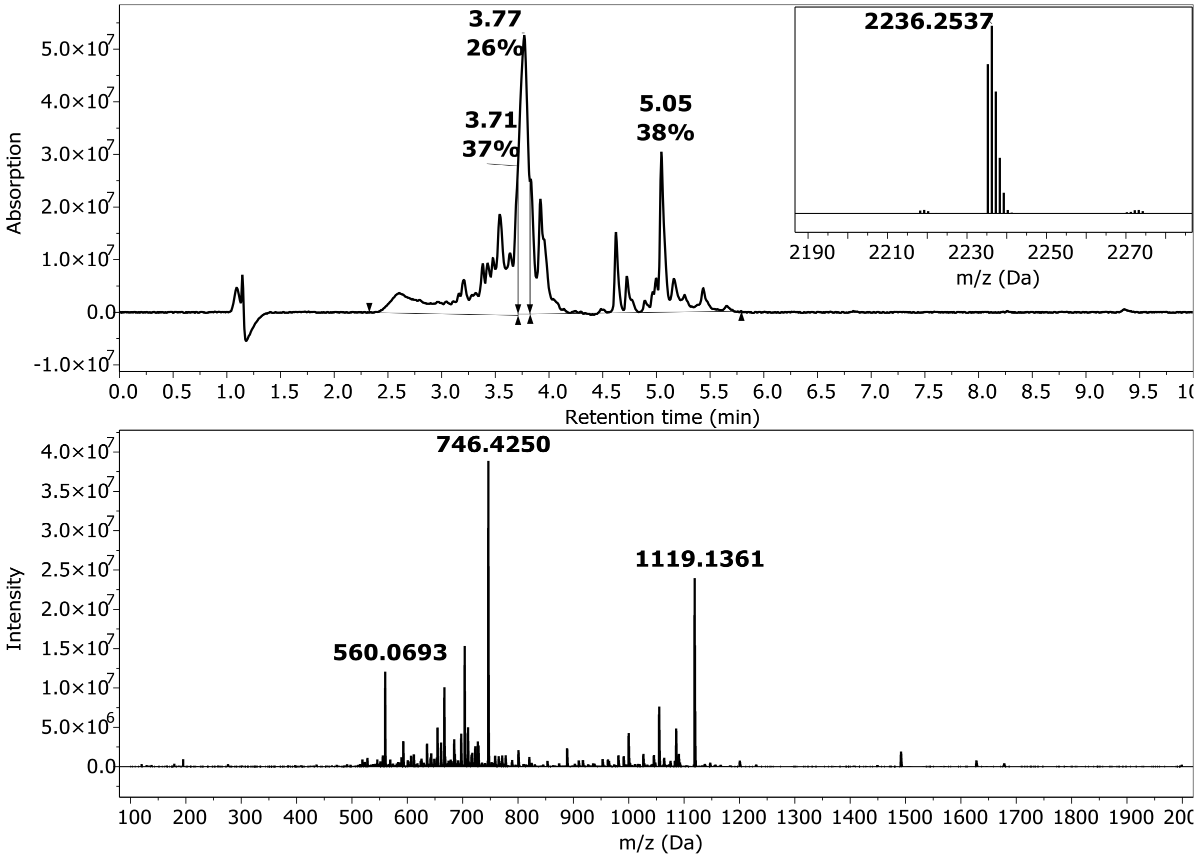

SI Figure 135. *LCMS Profile of crude Top: Absorbance chromatogram of shuffling 1 of PCP-4[43–62] Rt 3.77 min, 26% purity Bottom: ESI-TOF spectrum found within Rt 2–8 min (insert: deconvoluted masses). Monoisotopic mass (ESI+) calcd. for $C_{99}H_{166}N_{32}O_{27}$ 2235.2600, found 2235.2506.*

UHPLC

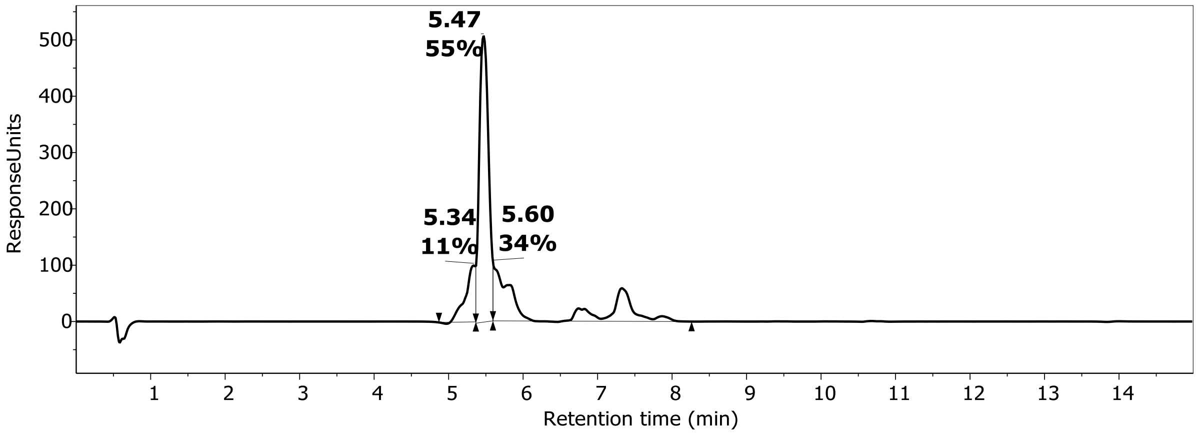

***SI Figure 136**. UHPLC profile of crude Rt 5.47 min, 55% purity based on Area Under Curve (AUC) at λ = 214 nm.*

6.9.3 Shuffling 2

H_2N- QSSSKAGVAQ FKKKFIRAQQ-$CONH_2$

The peptide shuffling 2 of PCP-4 [43–62] was synthesized on commercially available Novabiochem® NovaPEG Rink Amide resin (0.41 mmol/g, 99.6 mg, 42 µmol) using the standard AFPS protocol. (**SI Figure 137**) Total synthesis time to afford resin-bound peptide was approximately 1.0 h. Cleavage of the peptidyl-resin (15.1 mg, approx. 6.2 µmol) afforded the crude peptide as a colorless solid (3.9 mg, 45% purity by LCMS [**SI Figure 138**], 66% purity by UHPLC [**SI Figure 139**]).

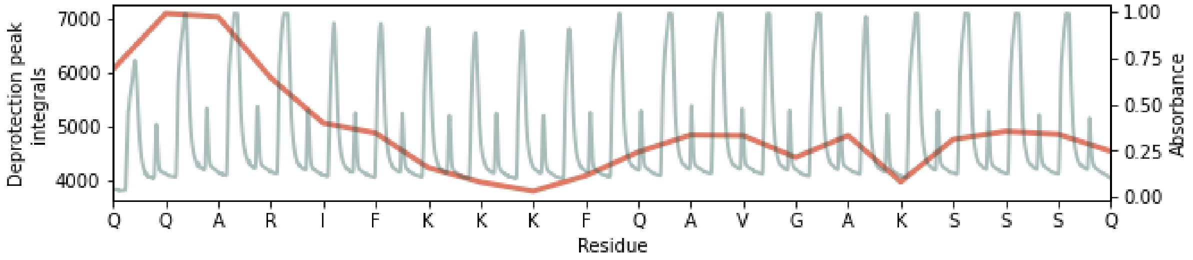

***SI Figure 137**. UV trace (λ = 310 nm) from AFPS of shuffling 2 of PCP-4[43–62] (green) and deprotection peak integrals (red).*

LC-MS of crude

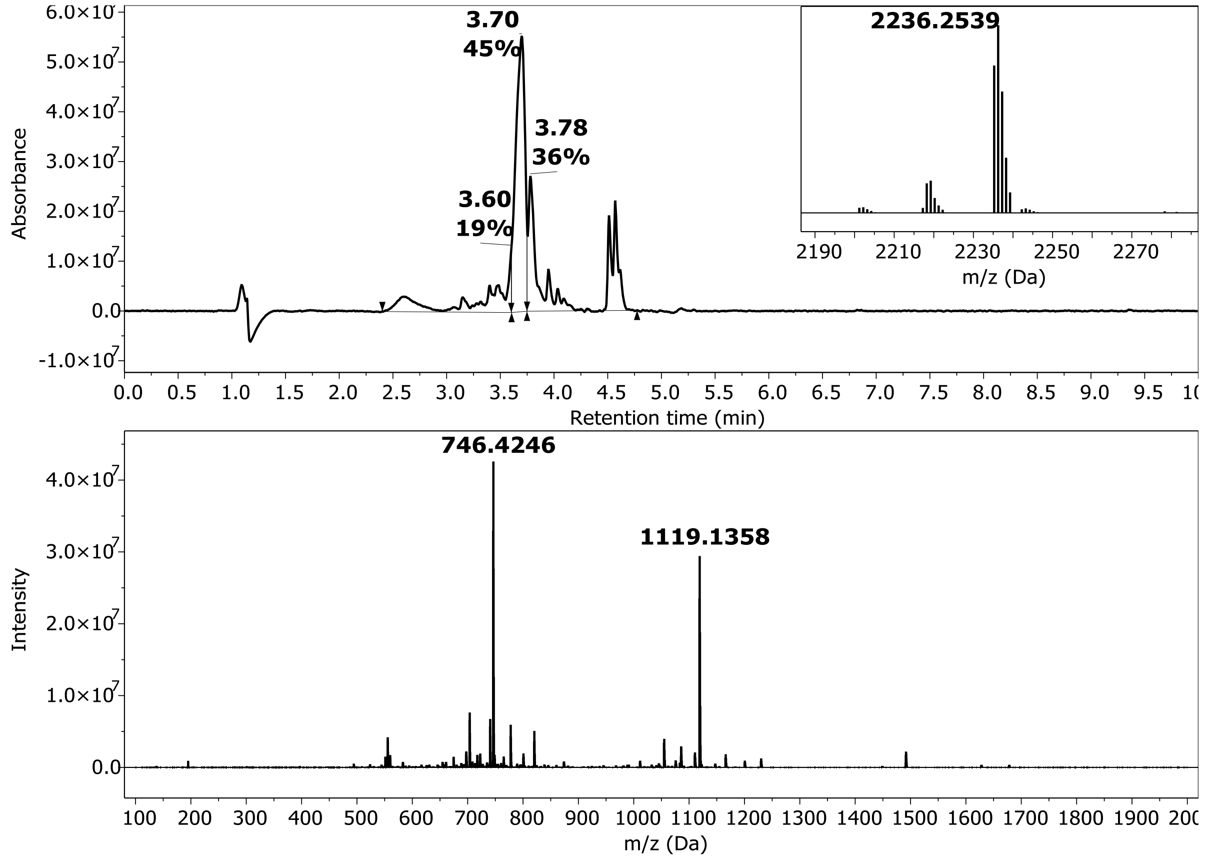

SI Figure 138. LCMS Profile of crude Top: Absorbance chromatogram of shuffling 2 of PCP-4[43–62] Rt 3.70 min, 45% purity Bottom: ESI-TOF spectrum found within Rt 2–8 min (insert: deconvoluted masses). Monoisotopic mass (ESI+) calcd. for for $C_{99}H_{166}N_{32}O_{27}$ 2235.2600, found 2235.2510.

UHPLC

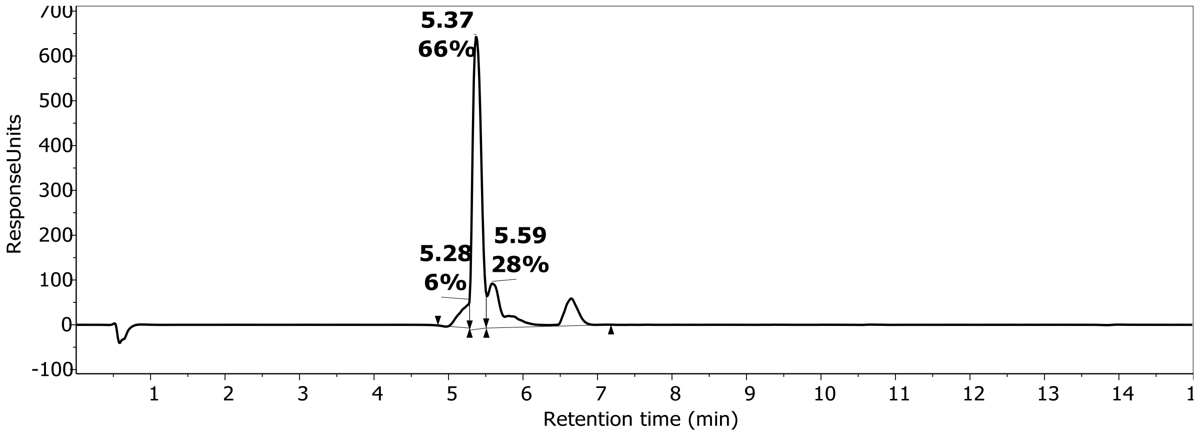

SI Figure 139: UHPLC profile of crude Rt 5.37 min, 66% purity based on Area Under Curve (AUC) at λ = 214 nm.

6.9.4 Shuffling 3

H_2N- FQSRSAKAIK KQFKGSQQAV-$CONH_2$

The peptide shuffling 3 of PCP-4[43–62] was synthesized on commercially available Novabiochem® NovaPEG Rink Amide resin (0.41 mmol/g, 99.8 mg, 41 µmol) using the standard AFPS protocol. (**SI Figure 140**) Total synthesis time to afford resin-bound peptide was approximately 1.0 h. Cleavage of

the peptidyl-resin (17.4 mg, approx. 7.1 µmol) afforded the crude peptide as a colorless solid (4.9 mg, 48% purity by LCMS [**SI Figure 141**], 78% purity by UHPLC [**SI Figure 142**]).

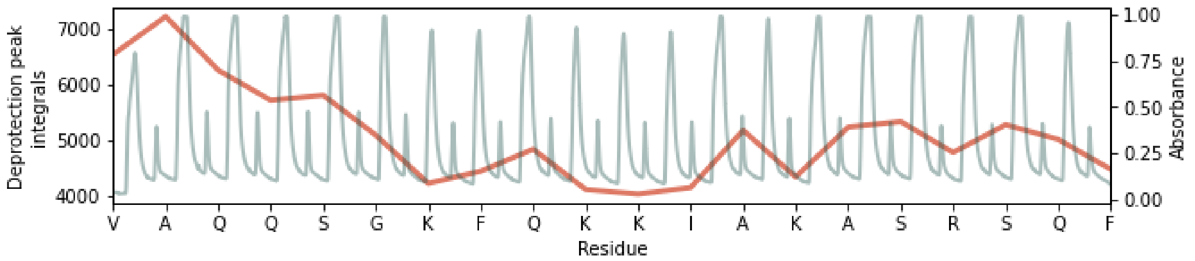

SI Figure 140. *UV trace (λ = 310 nm) from AFPS of shuffling 3 of PCP-4[43–62] (green) and deprotection peak integrals (red).*

LC-MS of crude

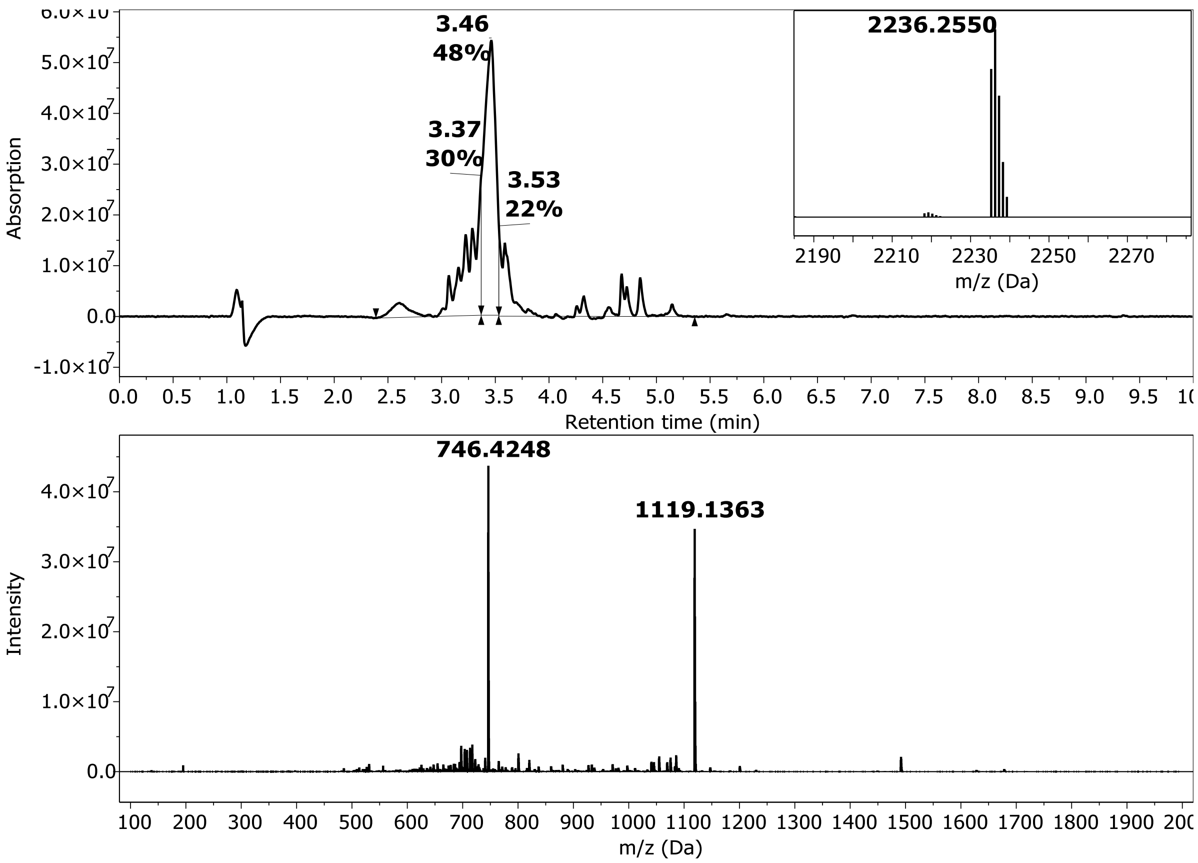

SI Figure 141. *LCMS Profile of crude Top: Absorbance chromatogram of shuffling 3 of PCP-4 [43–62] Rt 3.46 min, 48% purity Bottom: ESI-TOF spectrum found within Rt 2–8 min (insert: deconvoluted masses). Monoisotopic mass (ESI+) calcd. for for $C_{99}H_{166}N_{32}O_{27}$ 2235.2600, found 2235.2524.*

UHPLC

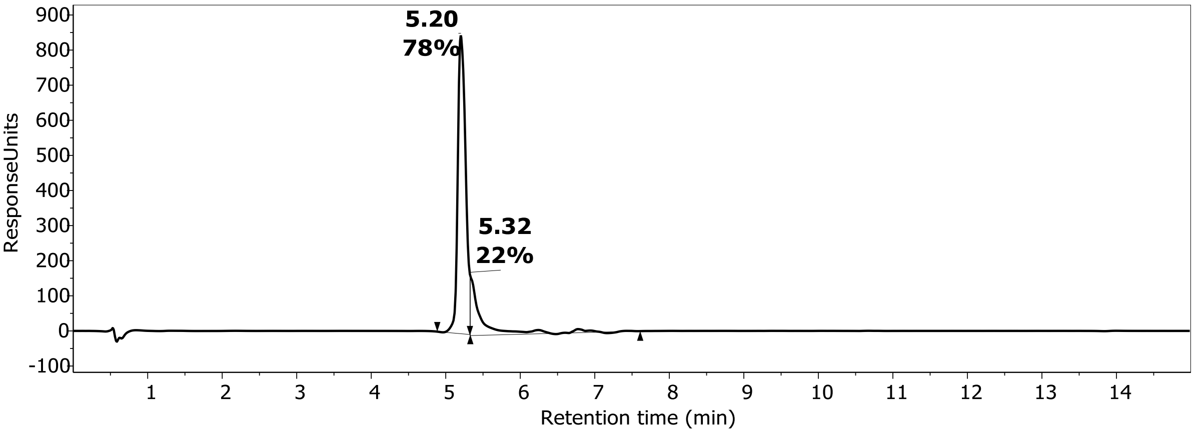

***SI Figure 142**. UHPLC profile of crude Rt 5.20 min, 78% purity based on Area Under Curve (AUC) at λ = 214 nm.*

6.9.5 Shuffling 4

H_2N- RIQSAGAKAV KFSKKQQSQF-$CONH_2$

The peptide shuffling 4 of PCP-4 [43–62] was synthesized on commercially available Novabiochem® NovaPEG Rink Amide resin (0.41 mmol/g, 100.6 mg, 41 µmol) using the standard AFPS protocol. (**SI Figure 143**). Total synthesis time to afford resin-bound peptide was approximately 1.0 h. Cleavage of the peptidyl-resin (16.5 mg, approx. 6.8 µmol) afforded the crude peptide as a colorless solid (3.5 mg, 28% purity by LCMS [**SI Figure 144**], 71% purity by UHPLC [**SI Figure 145**]).

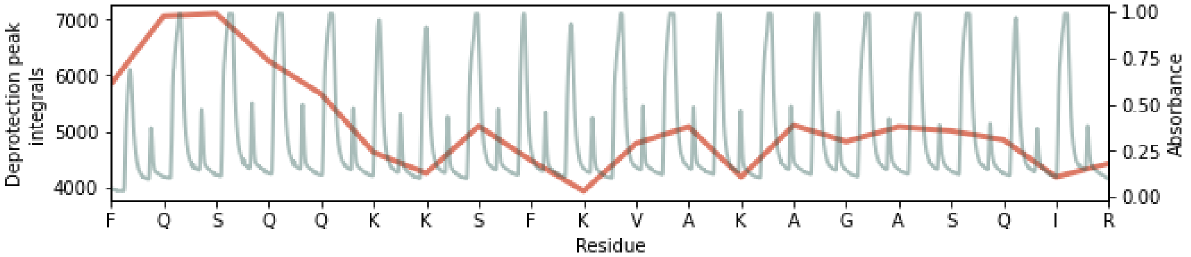

***SI Figure 143**. UV trace (λ = 310 nm) from AFPS of shuffling 4 of PCP-4[43–62] (green) and deprotection peak integrals (red).*

LC-MS of crude

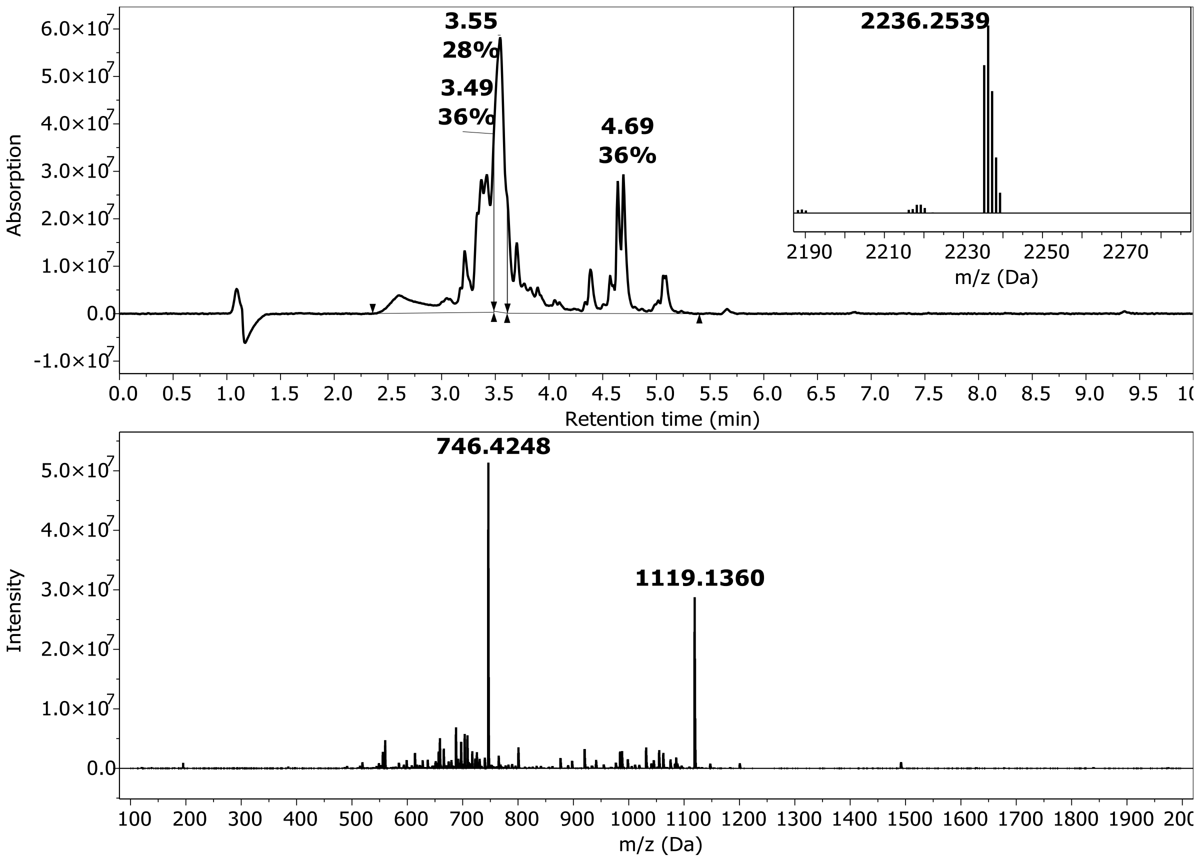

SI Figure 144. LCMS Profile of crude Top: Absorbance chromatogram of shuffling 4 of PCP-4[43–62] Rt 3.55 min, 28% purity Bottom: ESI-TOF spectrum found within Rt 2–8 min (insert: deconvoluted masses). Monoisotopic mass (ESI+) calcd. for for $C_{99}H_{166}N_{32}O_{27}$ 2235.2600, found 2235.2512.

UHPLC

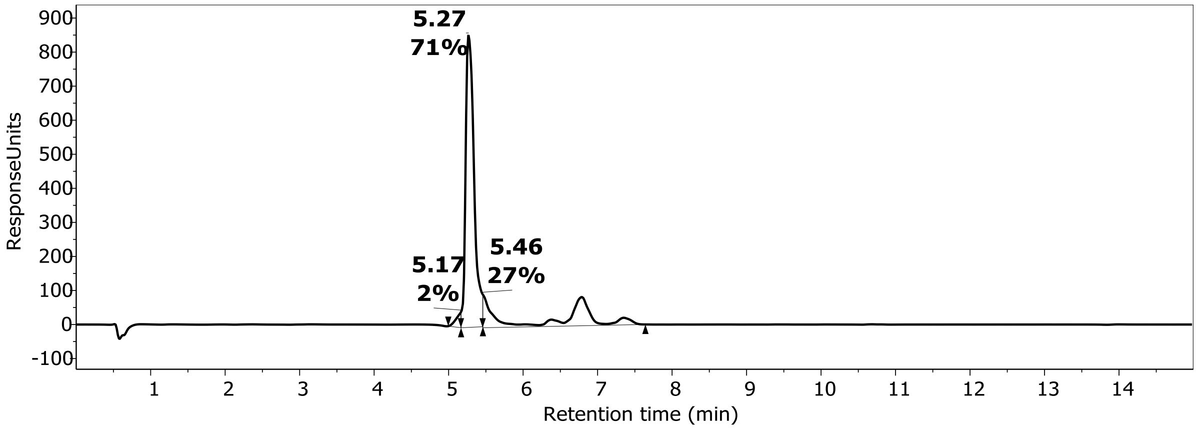

SI Figure 145. UHPLC profile of crude Rt 5.27 min, 71% purity based on Area Under Curve (AUC) at λ = 214 nm.

6.9.6 Shuffling 5

H_2N- SSFKSQGQQR QAKVAAKIKF-$CONH_2$

The peptide shuffling 5 of PCP-4[43–62] was synthesized on commercially available Novabiochem® NovaPEG Rink Amide resin (0.41 mmol/g, 100.3 mg, 41 µmol) using the standard AFPS protocol. (**SI Figure 146**) Total synthesis time to afford resin-bound peptide was approximately 1.0 h. Cleavage of the peptidyl-resin (17.5 mg, approx. 7.2 µmol) afforded the crude peptide as a colorless solid (4.1 mg, 45% purity by LCMS [**SI Figure 147**], 78% purity by UHPLC [**SI Figure 148**]).

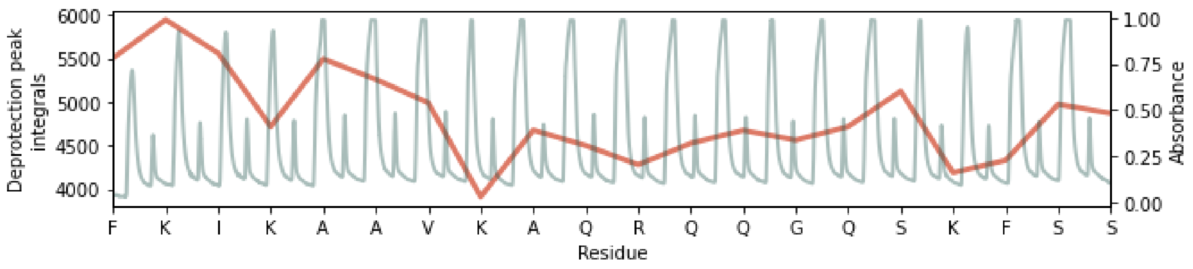

***SI Figure 146**. UV trace (λ = 310 nm) from AFPS of shuffling 5 of PCP-4[43–62] (green) and deprotection peak integrals (red).*

LC-MS of crude

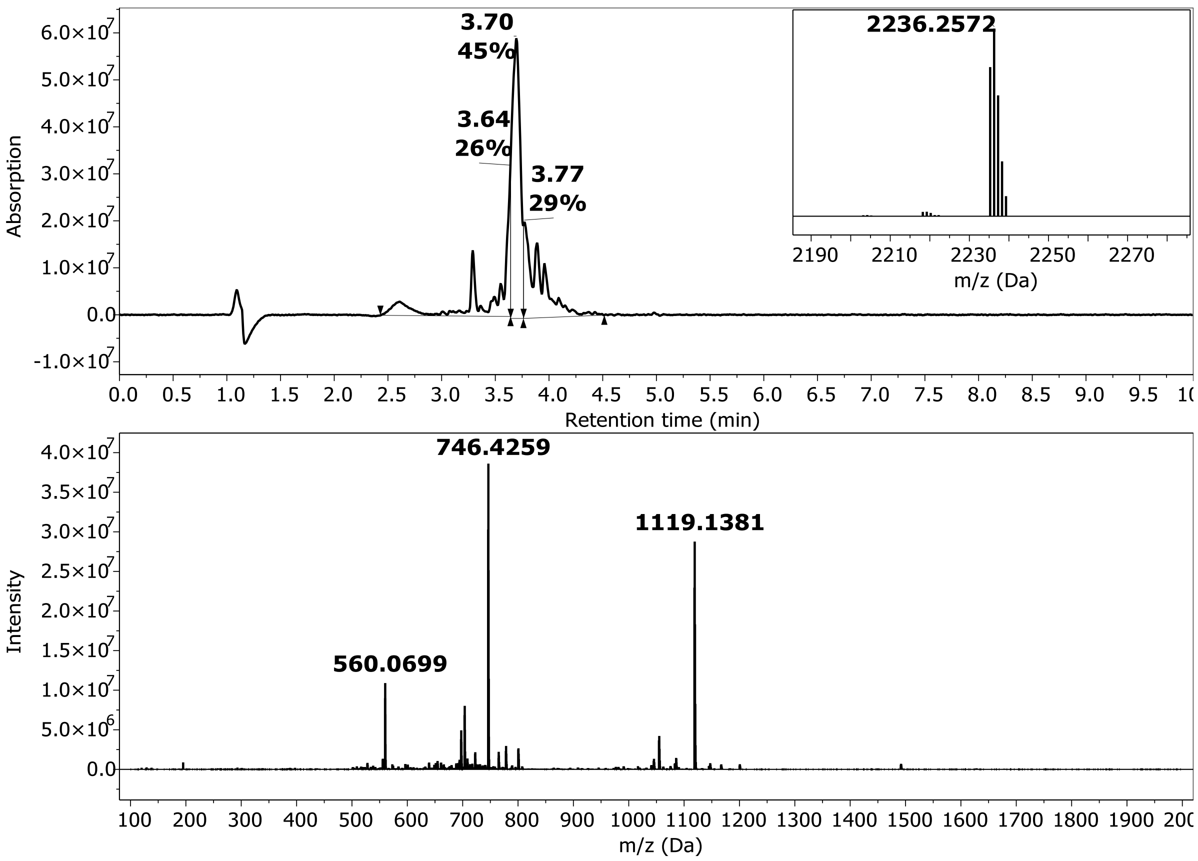

***SI Figure 147**: LCMS Profile of crude Top: Absorbance chromatogram of shuffling 5 of PCP-4 [43–62] Rt 3.70 min, 45% purity Bottom: ESI-TOF spectrum found within Rt 2–8 min (insert: deconvoluted masses). Monoisotopic mass (ESI+) calcd. for for $C_{99}H_{166}N_{32}O_{27}$ 2235.2600, found 2235.2544.*

UHPLC

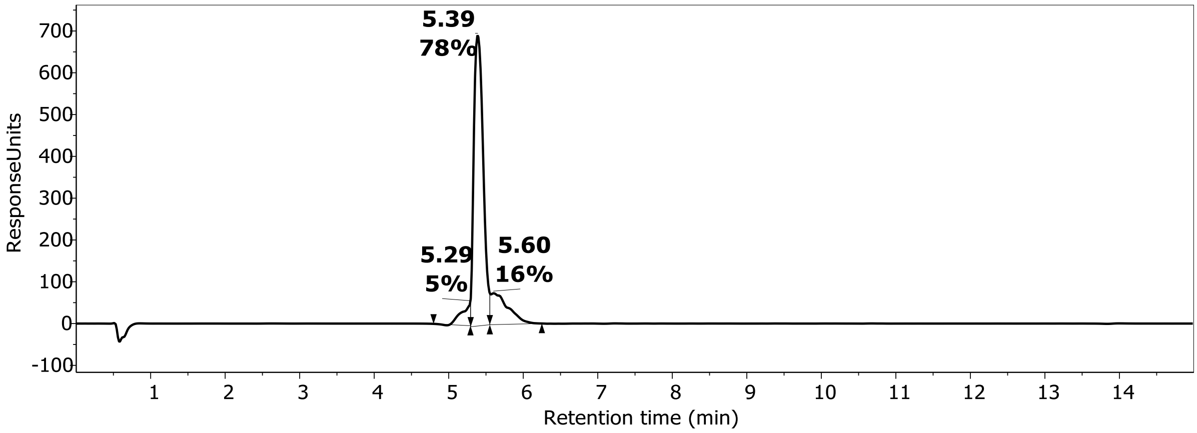

***SI Figure 148**. UHPLC profile of crude Rt 5.39 min, 78% purity based on Area Under Curve (AUC) at λ = 214 nm.*

7 Rational incorporation of pseudoproline to reduce aggregation

7.1 Example 1: hGH[176–191]

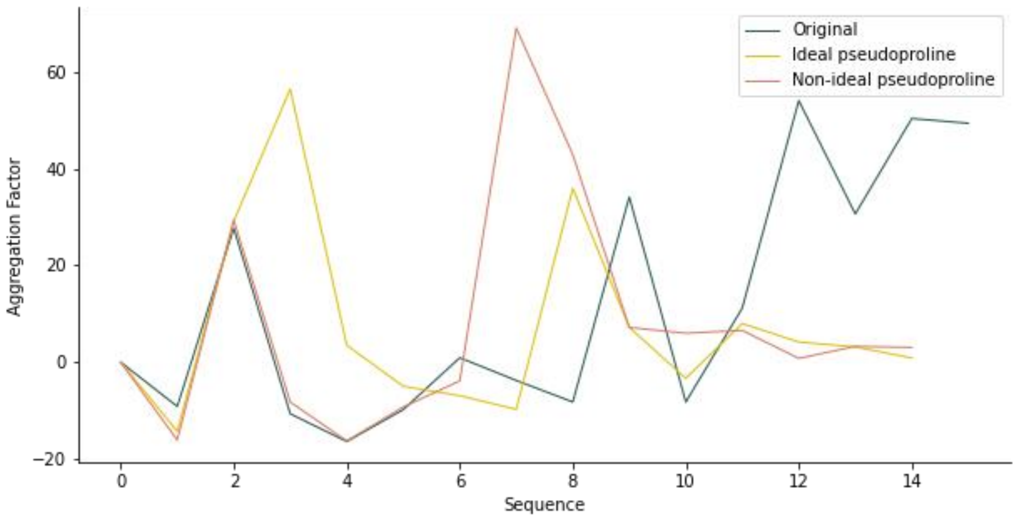

SI Figure 149*. Plot of aggregation factor values for the original and pseudoproline containing hGH[176–191] fragments*

7.1.1 Original sequence

H$_2$N- YLRIVQCRSV EGSCGF-CONH$_2$

The peptide hGH[176–191] was synthesized on commercially available Novabiochem® NovaPEG Rink Amide resin (0.41 mmol/g, 99.9 mg, 41 µmol) using the standard AFPS protocol (**SI Figure 150**). Total synthesis time to afford resin-bound peptide was approximately 0.8 h. Cleavage of the peptidyl-resin (17.3 mg, approx. 7.1 µmol) afforded the crude peptide as a colorless solid (2.4 mg, 28% purity by LCMS [**SI Figure 151**], 23% purity by UHPLC [**SI Figure 152**]).

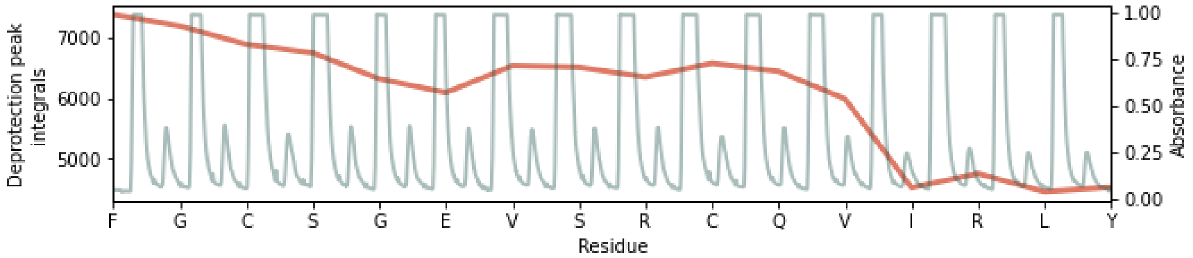

SI Figure 150*. UV trace (λ = 310 nm) from AFPS of hGH[176–191] (green) and deprotection peak integrals (red).*

LC-MS of crude

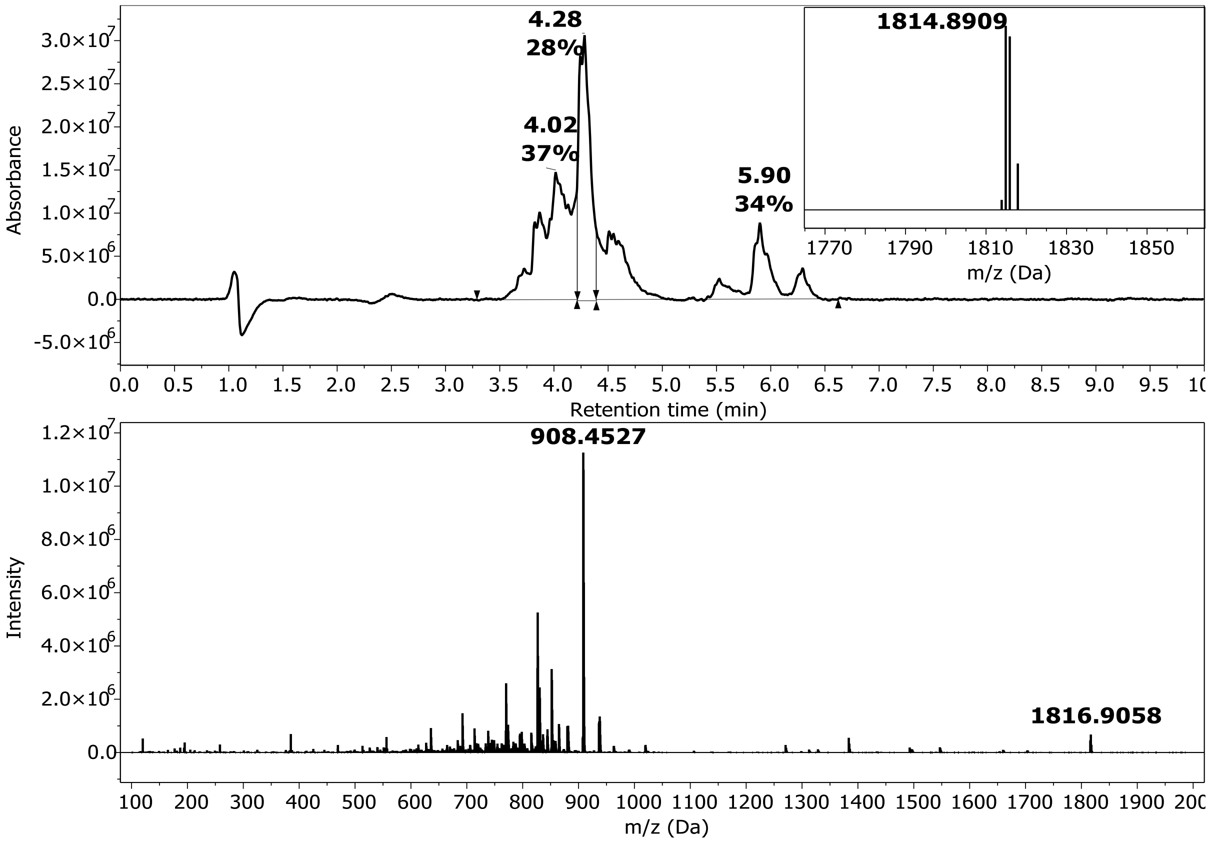

***SI Figure 151**. LCMS Profile of crude Top: Absorbance chromatogram of hGH[176–191] Rt 4.28 min, 28% purity Bottom: ESI-TOF spectrum found within Rt 2–8 min (insert: deconvoluted masses). Monoisotopic mass (ESI+) calcd. for $C_{78}H_{126}N_{24}O_{22}S_2$ 1814.8920, found 1814.8909.*

UHPLC

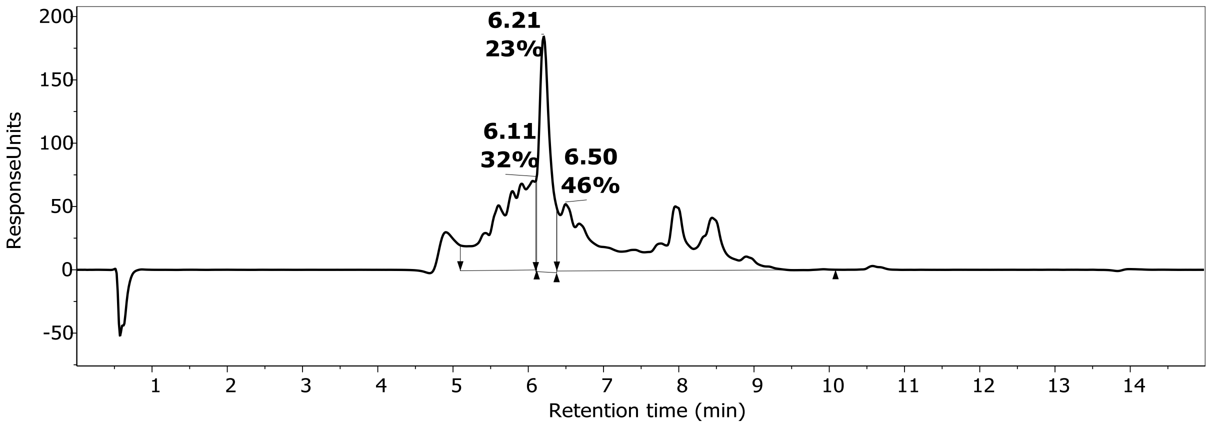

***SI Figure 152**. UHPLC profile of crude Rt 6.21 min, 23% purity based on Area Under Curve (AUC) at λ = 214 nm.*

7.1.2 Use of suggested pseudoproline

H_2N- YLRIVQCRSV E**GS**CGF-$CONH_2$

The peptide pseudoproline dimer (underlined part of the sequence) containing hGH[176–191] was synthesized on commercially available Novabiochem® NovaPEG Rink Amide resin (0.41 mmol/g, 100.7 mg, 41 μmol) using the standard AFPS protocol(**SI Figure 153**) Total synthesis time to afford resin-bound peptide was approximately 0.8 h. Cleavage of the peptidyl-resin (16.0 mg, approx. 6.6

μmol) afforded the crude peptide as a colorless solid (1.7 mg, 62% purity by LCMS [**SI Figure 154**], 69% purity by UHPLC [**SI Figure 155**]).

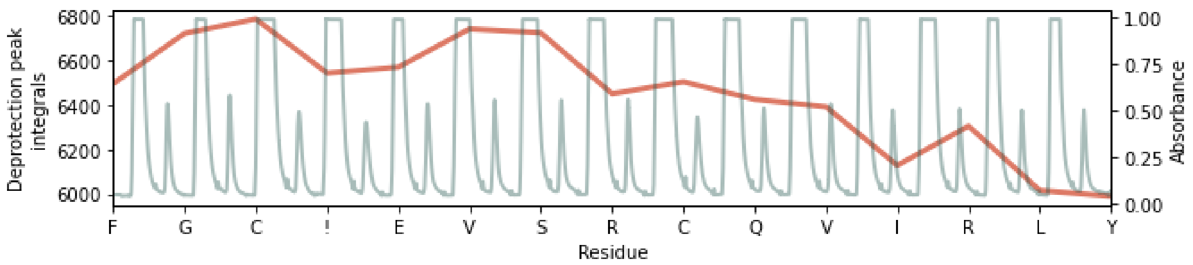

***SI Figure 153**. UV trace (λ = 310 nm) from AFPS of pseudoproline dimer containing of hGH[176–191] (green) and deprotection peak integrals (red).*

LC-MS of crude

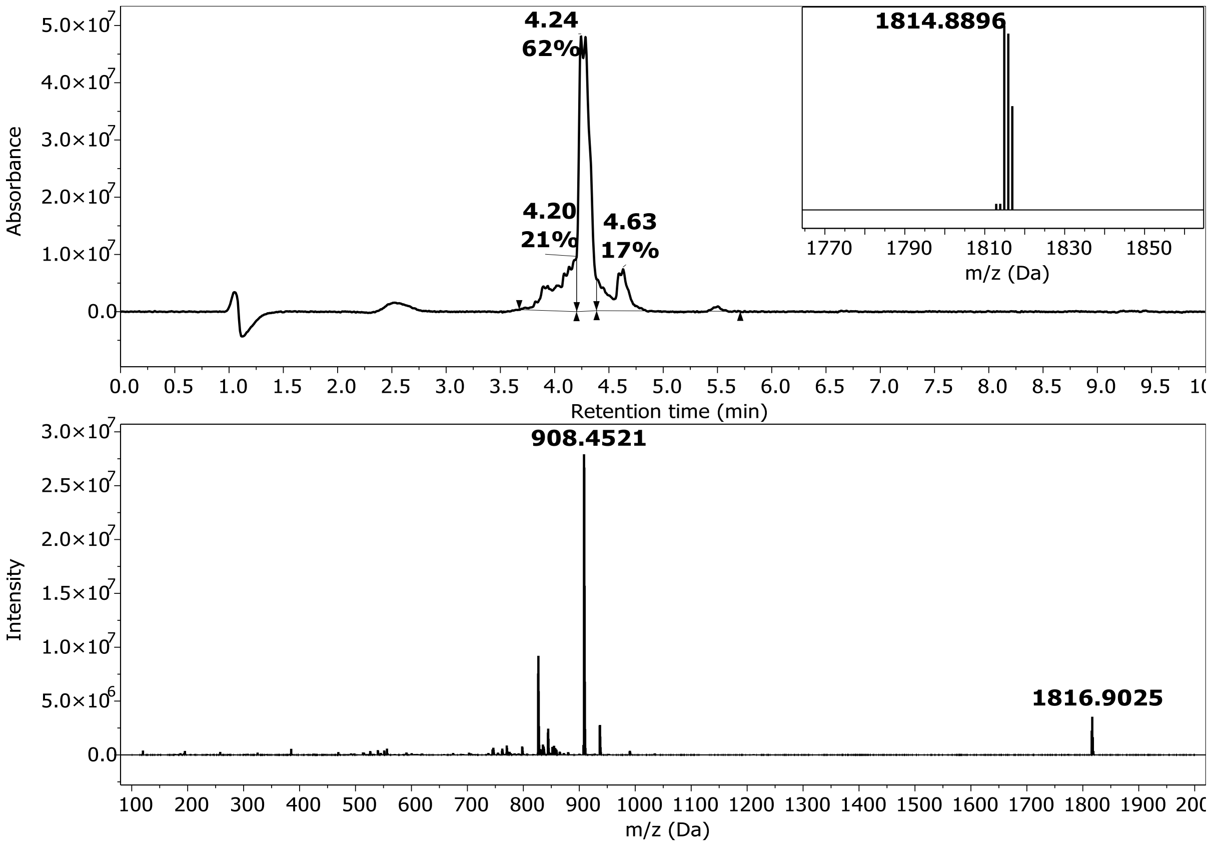

***SI Figure 154**. LCMS Profile of crude Top: Absorbance chromatogram of pseudoproline dimer containing hGH[176–191] Rt 4.24 min, 62% purity Bottom: ESI-TOF spectrum found within Rt 2–8 min (insert: deconvoluted masses). Monoisotopic mass (ESI+) calcd. for $C_{78}H_{126}N_{24}O_{22}S_2$ 1814.8920, found 1814.8896.*

UHPLC

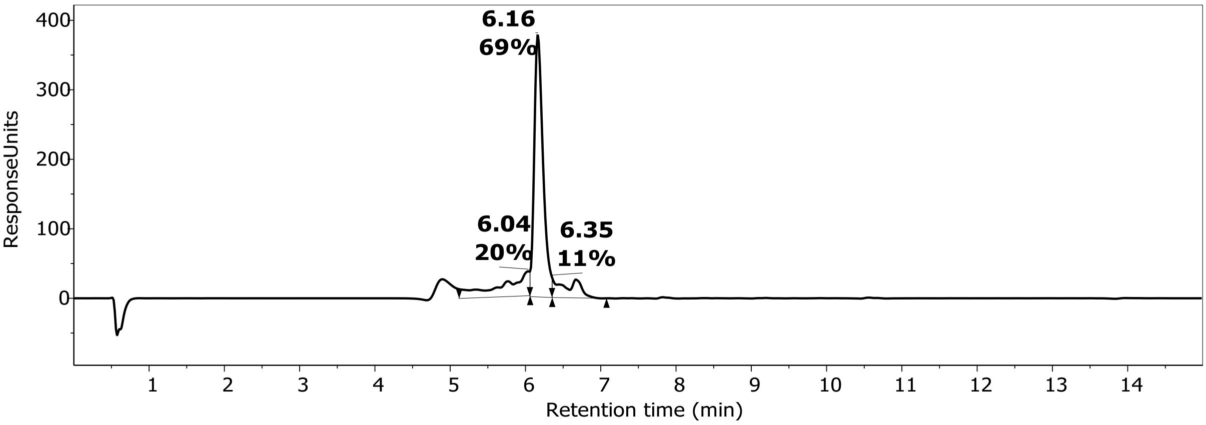

***SI Figure 155**. UHPLC profile of crude Rt 6.16 min, 69% purity based on Area Under Curve (AUC) at λ = 214 nm.*

7.1.3 Use of the non-optimal pseudoproline

H_2N- YLRIVQC**RS**V EGSCGF-CONH_2

The peptide pseudoproline dimer (underlined part of the sequence) containing hGH[176–191] was synthesized on commercially available Novabiochem® NovaPEG Rink Amide resin (0.41 mmol/g, 72.4 mg, 30 µmol) using the standard AFPS protocol (**SI Figure 156**) Total synthesis time to afford resin-bound peptide was approximately 0.8 h. Cleavage of the peptidyl-resin (17.0 mg, approx. 7.0 µmol) afforded the crude peptide as a colorless solid (5.9 mg, 40% purity by LCMS [**SI Figure 157**], 60% purity by UHPLC [**SI Figure 158**]).

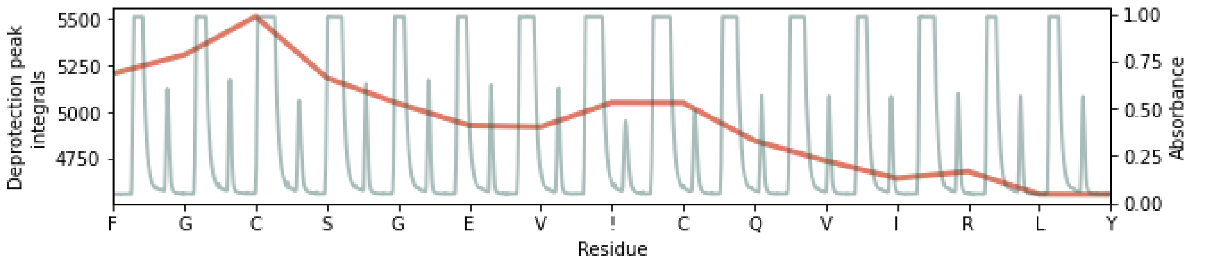

SI Figure 156. *UV trace (λ = 310 nm) from AFPS of pseudoproline dimer containing of hGH[176–191] (green) and deprotection peak integrals (red).*

LC-MS of crude

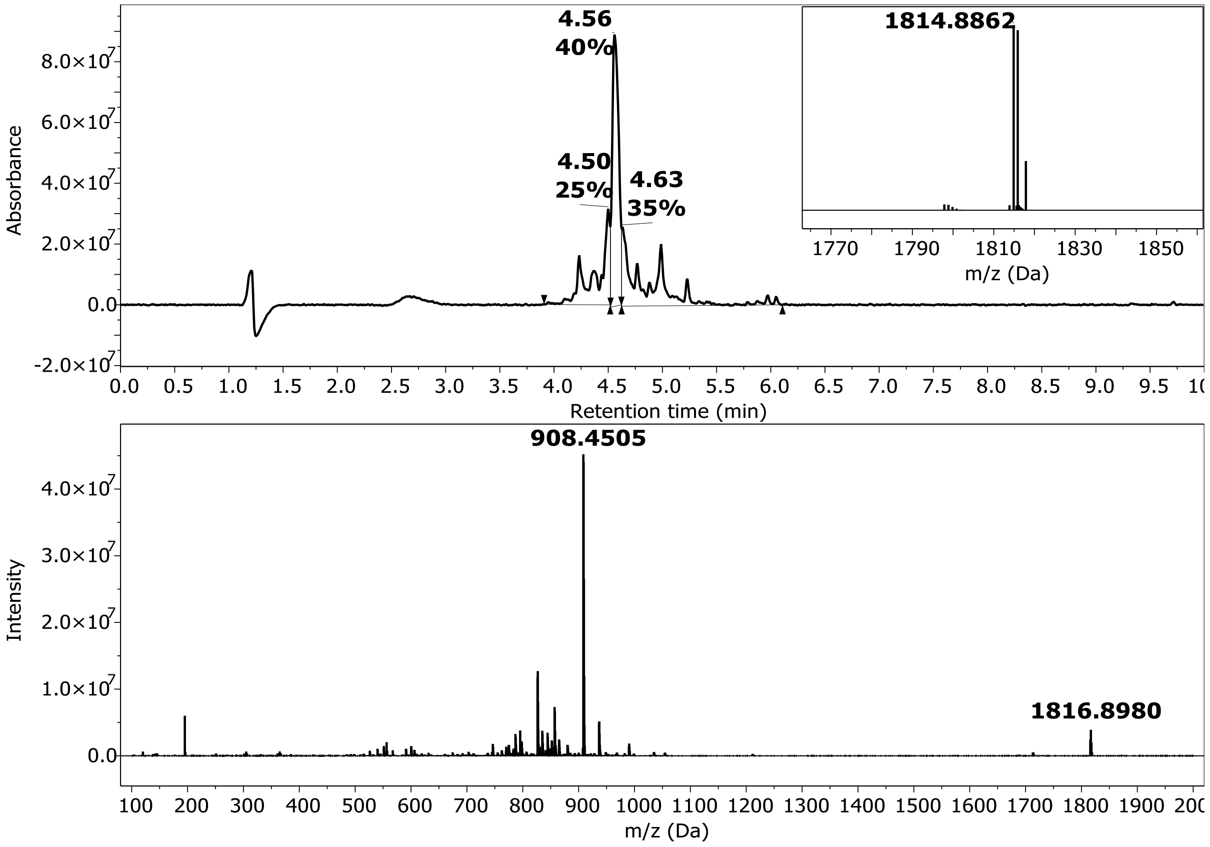

SI Figure 157. *LCMS Profile of crude Top: Absorbance chromatogram of pseudoproline dimer containing hGH[176–191] Rt 4.56 min, 40% purity Bottom: ESI-TOF spectrum found within Rt 2–8 min (insert: deconvoluted masses). Monoisotopic mass (ESI+) calcd. for $C_{78}H_{126}N_{24}O_{22}S_2$ 1814.8920, found 1814.8862.*

UHPLC

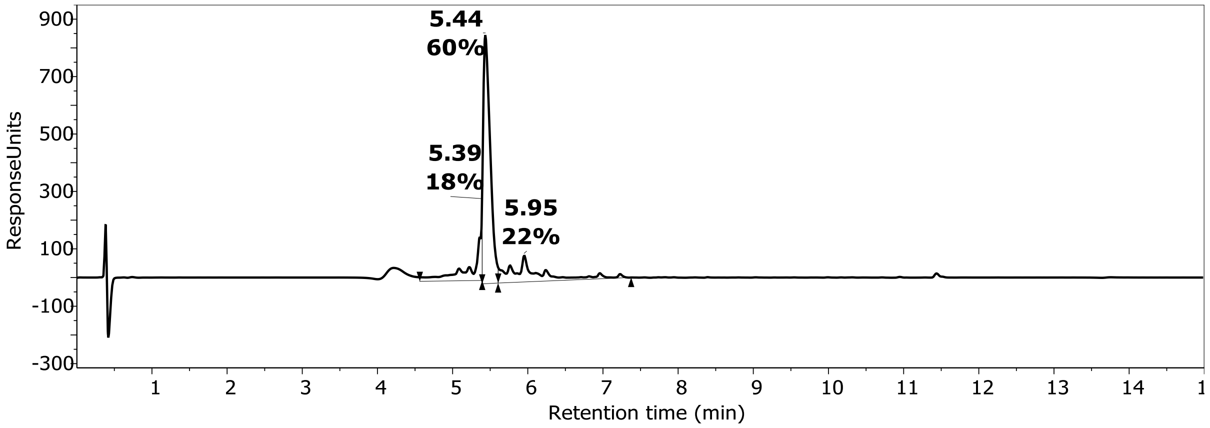

SI Figure 158. *UHPLC profile of crude Rt 5.44 min, 60% purity based on Area Under Curve (AUC) at λ = 214 nm.*

7.2 Example 2: GB1

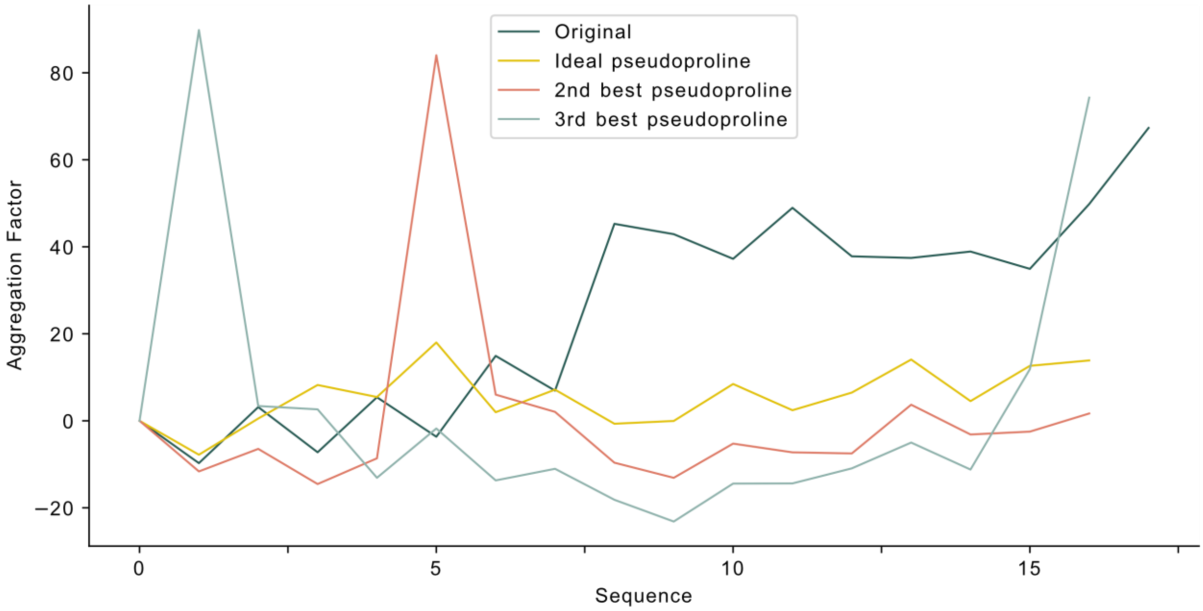

SI Figure 159*. Plot of aggregation factor values for the original and pseudoproline containing GB1 fragments.*

7.2.1 Original sequence

H_2N- VAGEWTYD DATKTFTVTE-$CONH_2$

The peptide GB1 was synthesized on commercially available Novabiochem® NovaPEG Rink Amide resin (0.41 mmol/g, 101.3 mg, 41 µmol) using the standard AFPS protocol. (**SI Figure 160**) Total synthesis time to afford resin-bound peptide was approximately 1.0 h. Cleavage of the peptidyl-resin (18.2 mg, approx. 7.5 µmol) afforded the crude peptide as a colorless solid (4.2 mg, 25% purity by LCMS [**SI Figure 161**], 17% purity by UHPLC [**SI Figure 162**]).

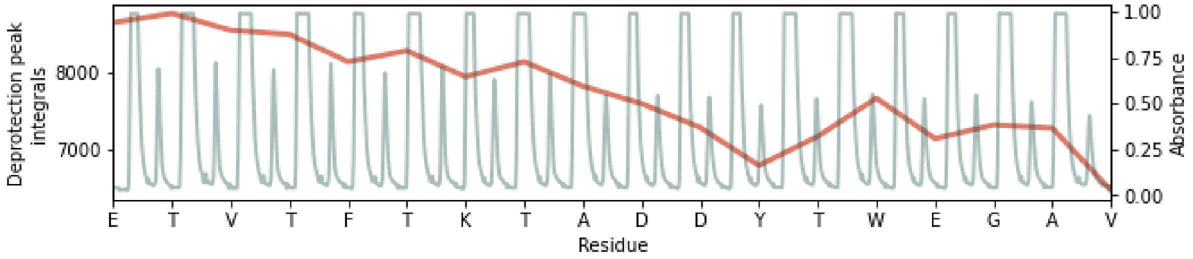

SI Figure 160*. UV trace (λ = 310 nm) from AFPS of GB1 (green) and deprotection peak integrals (red).*

LC-MS of crude

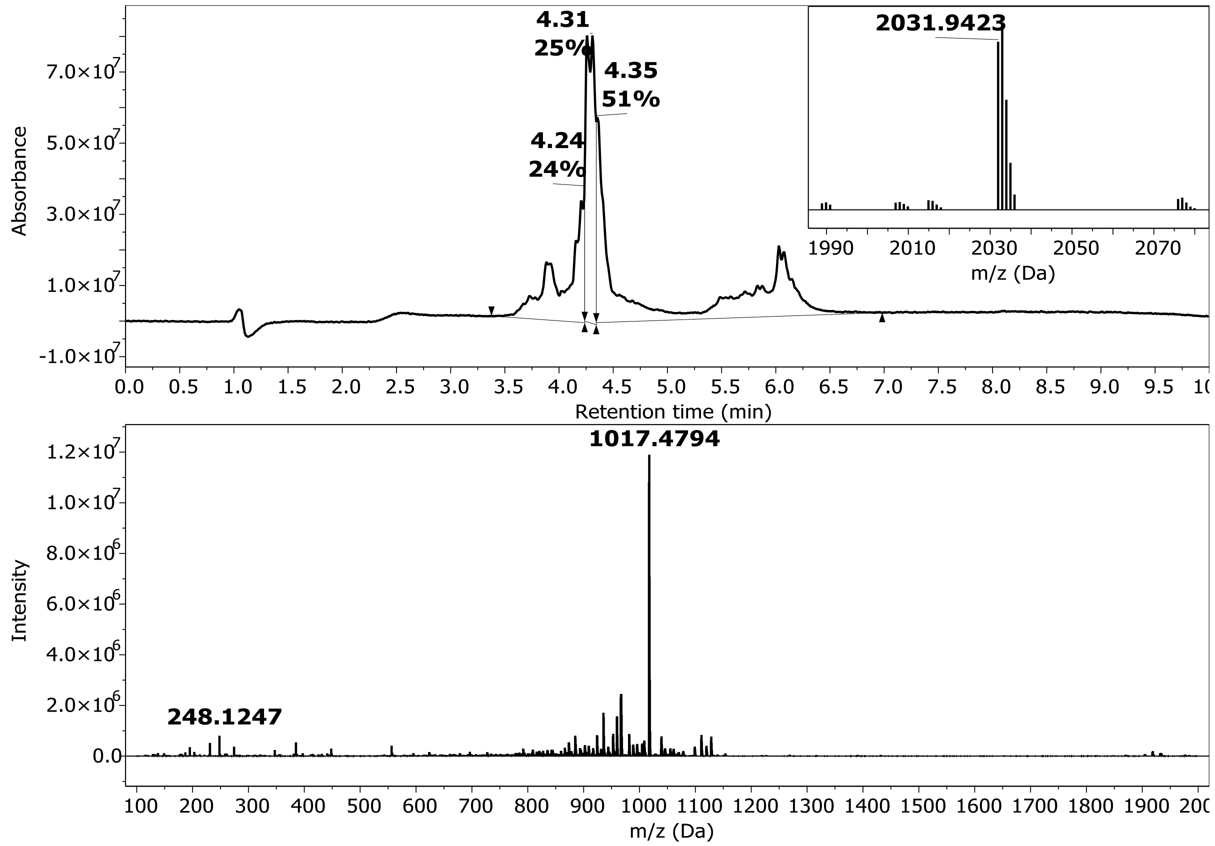

***SI Figure 161**. LCMS Profile of crude Top: Absorbance chromatogram of pseudoproline dimer-containing GB1. Rt 4.32 min, 25% purity. Bottom: ESI-TOF spectrum found within Rt 2–8 min (insert: deconvoluted masses). Monoisotopic mass (ESI+) calcd. for $C_{91}H_{133}N_{21}O_{32}$ 2031.9426, found 2031.9423.*

UHPLC

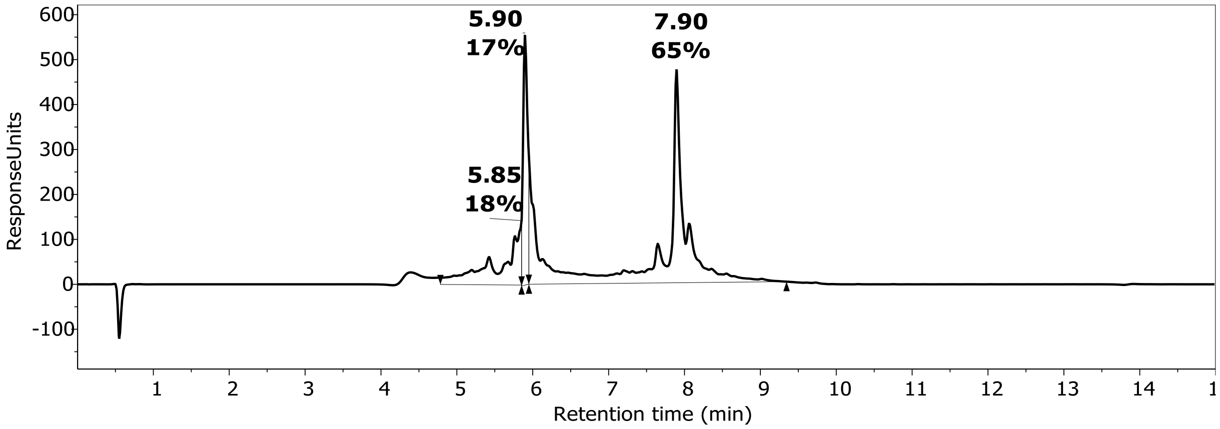

***SI Figure 162**. UHPLC profile of crude Rt 5.90 min, 17% purity based on Area Under Curve (AUC) at λ = 214 nm.*

7.2.2 Use of suggested pseudoproline

H_2N- VAGEWTYD DATKT**FT**VTE-$CONH_2$

The peptide pseudoproline dimer (underlined part of the sequence) containing GB1 was synthesized on commercially available Novabiochem® NovaPEG Rink Amide resin (0.41 mmol/g, 99.6 mg, 41 µmol) using the standard AFPS protocol(**SI Figure 163**) Total synthesis time to afford resin-bound peptide was approximately 1.0 h. Cleavage of the peptidyl-resin (17.1 mg, approx. 7.0 µmol) afforded the crude

peptide as a colorless solid (4.6 mg, 53% purity by LCMS [**SI Figure 164**], 75% purity by UHPLC [**SI Figure 165**]).

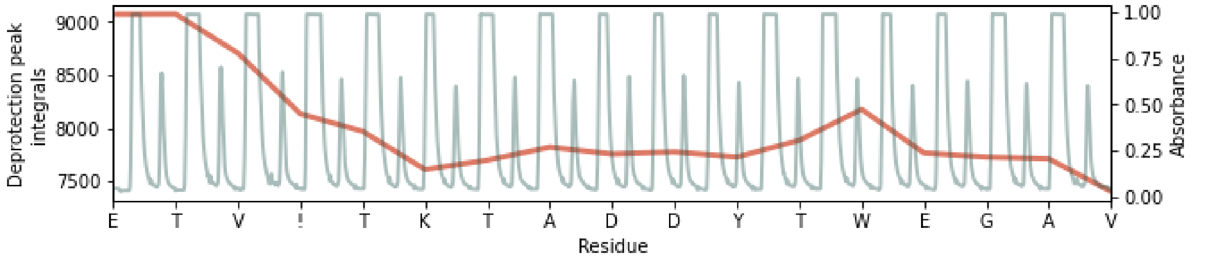

***SI Figure 163**. UV trace (λ = 310 nm) from AFPS of pseudoproline dimer containing GB1 (green) and deprotection peak integrals (red).*

LC-MS of crude

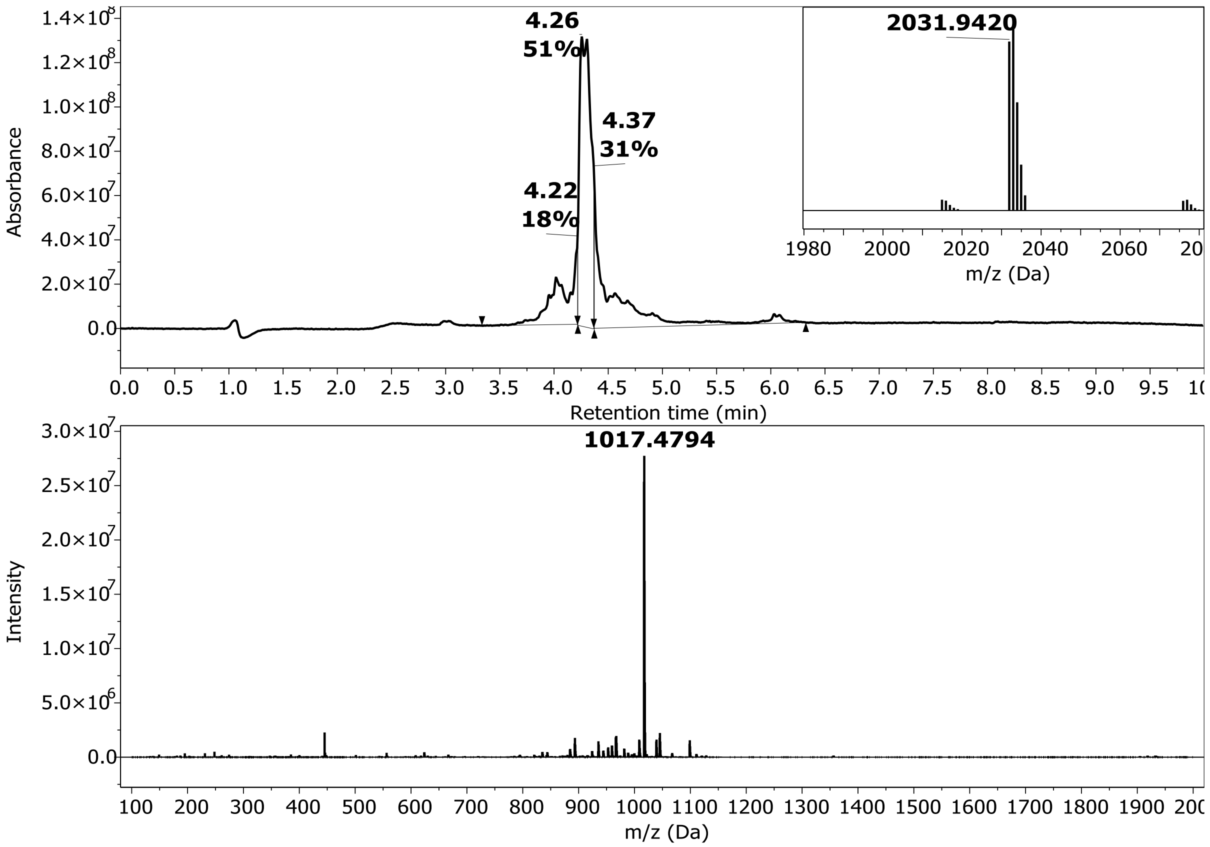

***SI Figure 164**. LCMS Profile of crude Top: Absorbance chromatogram of pseudoproline dimer containing GB1 Rt 4.31 min, 53% purity. Bottom: ESI-TOF spectrum found within Rt 2–8 min (insert: deconvoluted masses). Monoisotopic mass (ESI+) calcd. for $C_{91}H_{133}N_{21}O_{32}$ 2031.9426, found 2031.9420.*

UHPLC

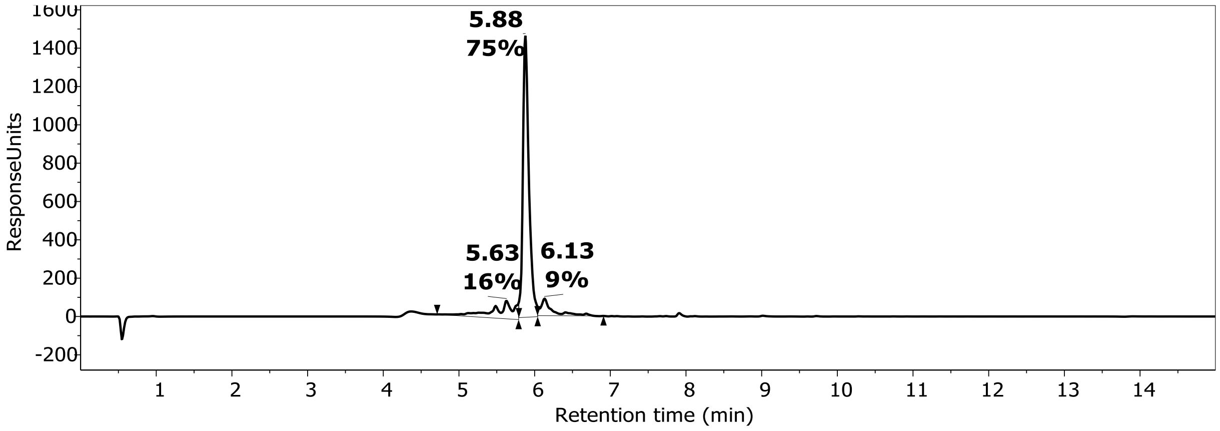

SI Figure 165. *UHPLC profile of crude Rt 5.88 min, 75% purity based on Area Under Curve (AUC) at λ = 214 nm.*

7.2.3 Use of 2nd suggested pseudoproline

H$_2$N- VAGEWTYD DAT**KT**FTVTE-CONH$_2$

The peptide pseudoproline dimer (underlined part of the sequence) containing GB1 was synthesized on commercially available Novabiochem® NovaPEG Rink Amide resin (0.41 mmol/g, 68.9 mg, 28 µmol) using the standard AFPS protocol(**SI Figure 166**) Total synthesis time to afford resin-bound peptide was approximately 1.0 h. Cleavage of the peptidyl-resin (17.4 mg, approx. 7.0 µmol) afforded the crude peptide as a colorless solid (4.6 mg, 48% purity by LCMS [**SI Figure 167**], 71% purity by UHPLC [**SI Figure 168**]).

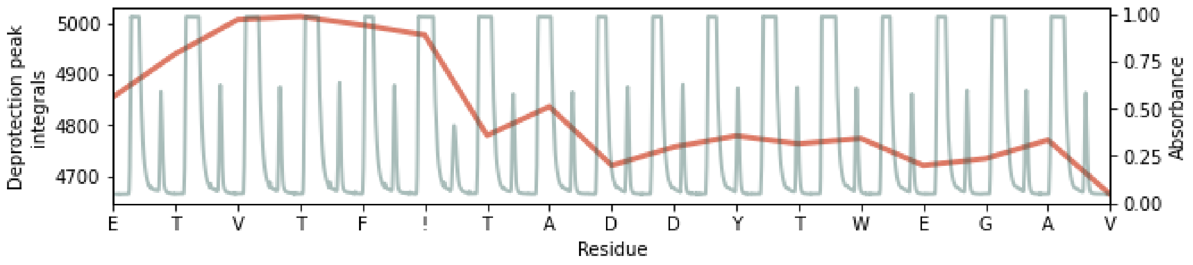

SI Figure 166. *UV trace (λ = 310 nm) from AFPS of pseudoproline dimer containing GB1 (green) and deprotection peak integrals (red).*

LC-MS of crude

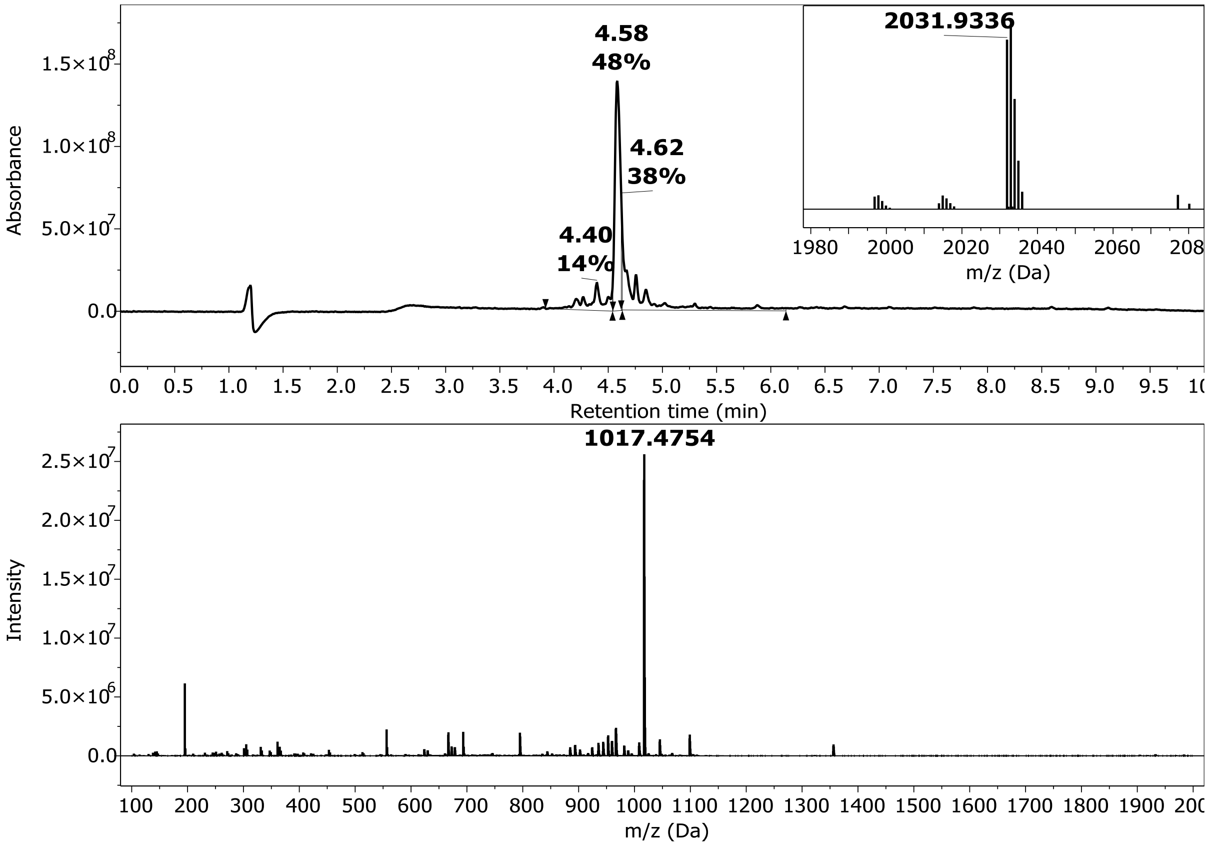

SI Figure 167. *LCMS Profile of crude Top: Absorbance chromatogram of pseudoproline dimer containing GB1 Rt 4.58 min, 48% purity. Bottom: ESI-TOF spectrum found within Rt 2–8 min (insert: deconvoluted masses). Monoisotopic mass (ESI+) calcd. for* $C_{91}H_{133}N_{21}O_{32}$ *2031.9426, found 2031.9336.*

UHPLC

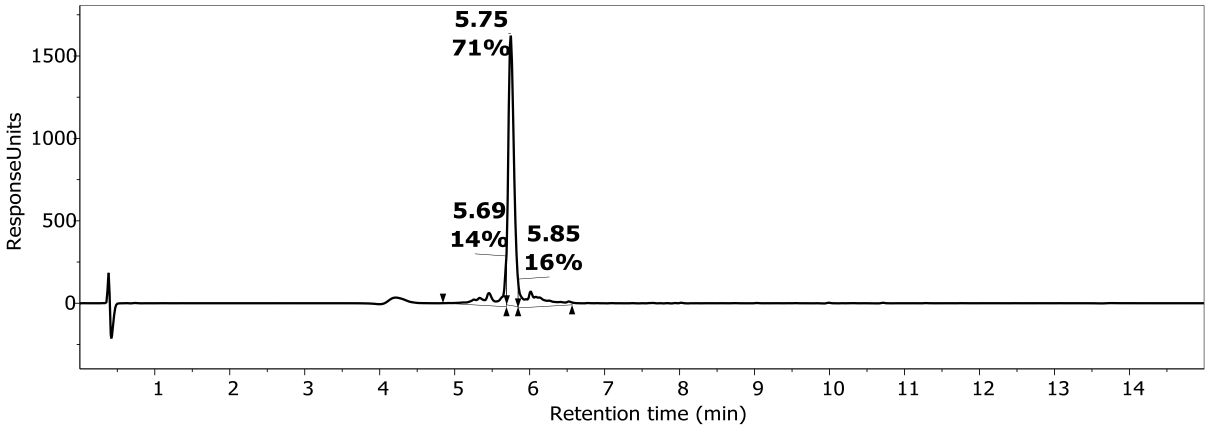

***SI Figure 168**. UHPLC profile of crude Rt 5.75 min, 71% purity based on Area Under Curve (AUC) at λ = 214 nm.*

7.2.4 Use of 3rd suggested pseudoproline

H2N- VAGEWTYD DATKTFT**VT**E-CONH2

The peptide pseudoproline dimer (underlined part of the sequence) containing GB1 was synthesized on commercially available Novabiochem® NovaPEG Rink Amide resin (0.41 mmol/g, 99.6 mg, 41 µmol) using the standard AFPS protocol(**SI Figure 169**) Total synthesis time to afford resin-bound peptide was approximately 1.0 h. Cleavage of the peptidyl-resin (17.1 mg, approx. 7.0 µmol) afforded the crude peptide as a colorless solid (4.6 mg, 31% purity by LCMS [**SI Figure 170**], 51% purity by UHPLC [**SI Figure 171**]).

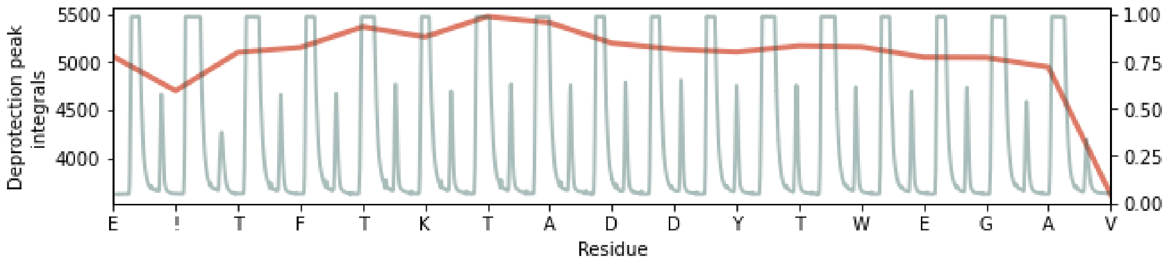

***SI Figure 169**. UV trace (λ = 310 nm) from AFPS of pseudoproline dimer containing GB1 (green) and deprotection peak integrals (red).*

LC-MS of crude

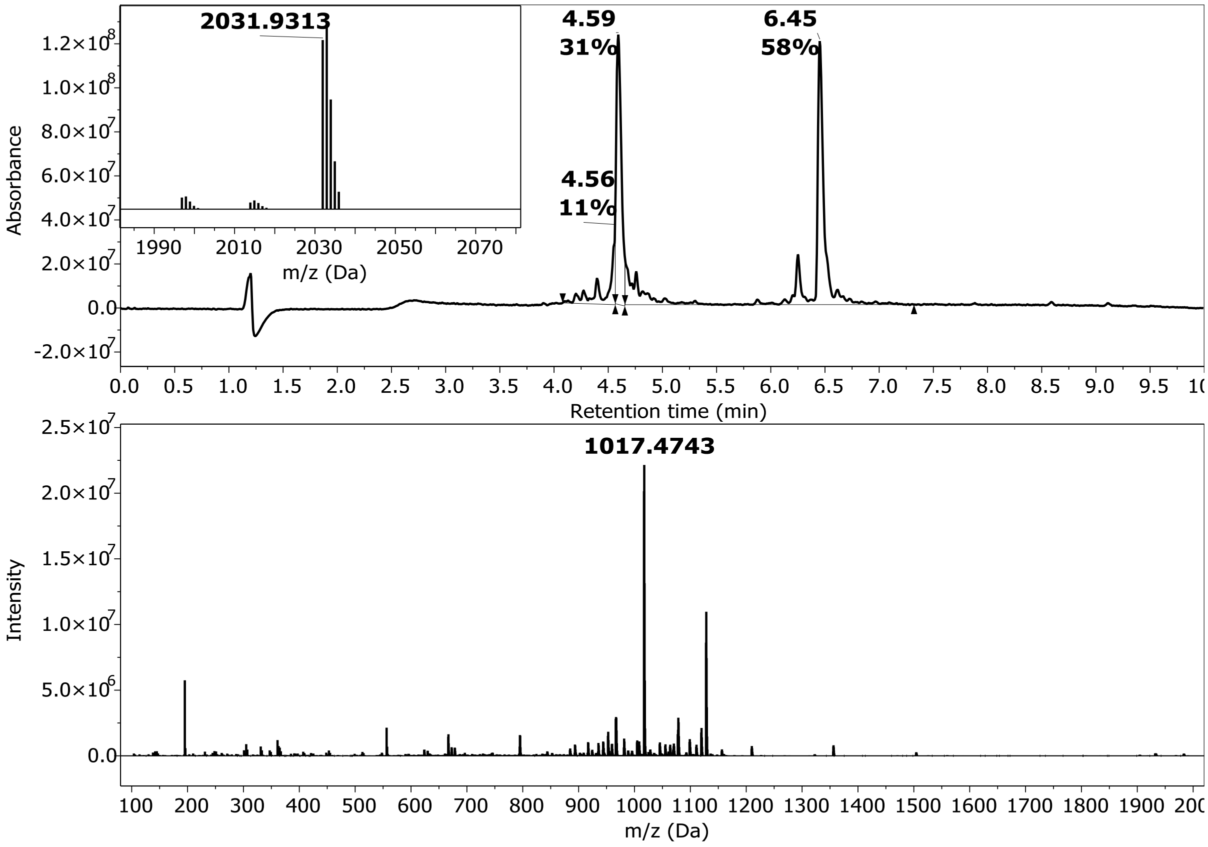

***SI Figure 170**. LCMS Profile of crude Top: Absorbance chromatogram of pseudoproline dimer containing GB1 Rt 4.59 min, 31% purity. Bottom: ESI-TOF spectrum found within Rt 2–8 min (insert: deconvoluted masses). Monoisotopic mass (ESI+) calcd. for $C_{91}H_{133}N_{21}O_{32}$ 2031.9426, found 2031.9313.*

UHPLC

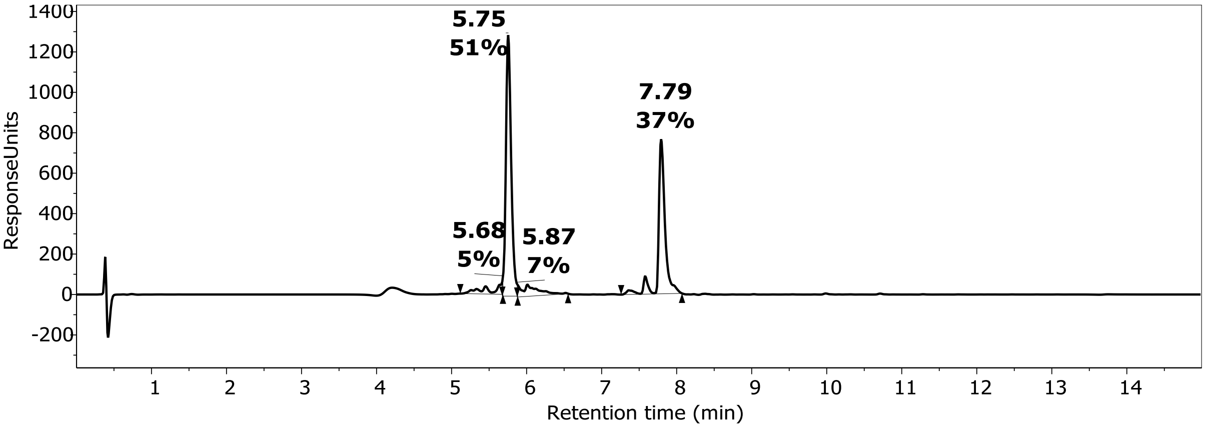

***SI Figure 171**. UHPLC profile of crude Rt 5.75 min, 51% purity based on Area Under Curve (AUC) at λ = 214 nm.*
